# Organosulfur Materials with High Photo- and Photo-Oxidation Stability: 10-Anthryl Sulfoxides and Sulfones and Their Photophysical Properties Dependent on the Sulfur Oxidation State

**DOI:** 10.3390/ma14133506

**Published:** 2021-06-23

**Authors:** Piotr Bałczewski, Emilia Kowalska, Ewa Różycka-Sokołowska, Paweł Uznański, Joanna Wilk, Marek Koprowski, Krzysztof Owsianik, Bernard Marciniak

**Affiliations:** 1 Functional Materials Synthesis Group, Division of Organic Chemistry, Centre of Molecular and Macromolecular Studies, Polish Academy of Sciences, Sienkiewicza 112, 90-363 Łódź, Poland; kowala88@vp.pl (E.K.); jskalik@cbmm.lodz.pl (J.W.); mkopr@cbmm.lodz.pl (M.K.); owsianik@cbmm.lodz.pl (K.O.); 2 Structural & Material Chemistry Group, Faculty of Science and Technology, Institute of Chemistry, Jan Dlugosz University in Częstochowa, Armii Krajowej 13/15, 42-200 Częstochowa, Poland; e.sokolowska@ujd.edu.pl (E.R.-S.); b.marciniak@ujd.edu.pl (B.M.); 3 Centre of Molecular and Macromolecular Studies, Division of Polymers, Polish Academy of Sciences, Sienkiewicza 112, 90-363 Łódź, Poland; puznansk@cbmm.lodz.pl

**Keywords:** aromatic sulfides, aromatic sulfoxides, aromatic sulfones, optoelectronic materials, photostability, photooxidation, light emitters, photoluminescence lifetimes

## Abstract

While few studies show only symmetrical and poorly mono-SO_n_ (n = 0–2) substituted acenes, in this study, we present a synthesis of a new group of unsymmetrical, significantly substituted derivatives, which revealed unique photophysical properties. Both sulfides (S), sulfoxides (SO) and sulfones (SO_2_) showed very high photochemical stabilities, unusual for these groups, during UV-irradiation at 254/365 nm (air O_2_ and Ar), which was higher than any found in the literature. For the (S)/(SO) series (254 nm), the stabilities of 80–519 min. (air O_2_ and Ar) were found. At 365 nm, stabilities of 124—812 min./(air O_2_) for (S)/(SO) and higher for (SO_2_) were observed. Photoluminescence lifetimes of (SO_n_) of the lower anthryl symmetry remained in the following order: (SO_2_) < (S) < (SO); those with full symmetry were in the following order: (S) < (SO) < (SO_2_). The enhanced photostability was explained with DFT/MS/Hammett’s constants, which showed the leading role of the SO_n_ groups in stabilization of HOMO/LUMO frontier orbitals. The SO_n_ (n = 0–2) substituted acenes turned out to be tunable violet/blue/green emitters by oxidation of S atoms and the introduction of rich substitution.

## 1. Introduction

(Hetero)aromatic sulfoxides (SO) and sulfones (SO_2_), the oxidized analogs of the corresponding sulfides (S), have recently been investigated as low molecular weight and polymeric materials in optoelectronic devices, such as: organic light-emitting diodes [1,2,3,4,5,6,7,8], solar cells [9,10], chemosensors [11,12,13], photosensitizers [14], photoreactive inks [15], redox switches [16] and as compounds exhibiting other interesting electronic and photophysical properties [17,18,19,20,21]. They have also been reported as useful pharmaceuticals [22,23] and agrochemicals [24].

Due to the high electronegativity of oxygen, the sulfoxide S(O) and sulfone S(O)_2_ moieties are powerful electron-withdrawing groups in comparison to the sulfide precursors. Electron character of the latter deserves, however, a comment. The sulfide groups become quite strong electron donors when attached to electron-poor aromatic systems, while, when attached to electron-rich aromatics, they turn into weak acceptors [21]. The sulfur bridges between two (hetero)aromatic moieties exhibit tetrahedral geometry that can reduce the p-conjugation length to effectively develop blue emitters [25]. In polymeric materials, the limited conjugation endows the polymers with a weak optical absorption and a high transparency in the visible region [9]. The S(O) or S(O)_2_ bridges, between two (hetero)aromatic moieties, may deepen the frontier molecular orbital energy levels, and hence improve the electron collection and the hole blocking abilities of the resulting polymer solar cells [9]. A successive oxidation of the bridging sulfur atom in the SO_n_ (n = 0, 1) bridges causes a large increase in photoluminescence yield (Φ) of the resulting chromophores [26]. Some studies conclude that the rapid intersystem crossing (ISC) to the triplet state is the main deactivation process limiting the photoluminescence (PL) quantum yield Φ, as in terthiophene dimers [27], but other investigations show that differently to the latter, the SO_n_ bridged chromophores do not require competing ISC, as in binaphthyl systems, to limit the PL emission [28]. It is believed that these conclusions can be extrapolated to other sulfur-bridged conjugated chromophores [25]. In the above investigations, the different PL efficiencies upon oxidation of the sulfur bridge have been rationalized by the existence of energetically available non-radiative decays in the (S) and S(O) bridges. Both studies confirmed the generality of the electron lone-pair(s) screening concept, which assumed that electron lone-pair(s) on the sulfur atom in (S) and (SO) screened the electronic interactions between the two (hetero)aromatic chromophores. According to the concept, the Φ limiting ISC efficiency is reduced in the presence of the intramolecular charge transfer (CT), which can be tuned by the oxidation state of the SO_n_ bridge. This causes a decrease in the CT and allows the efficient ISC in symmetrical (S) and (SO), which results in lower PL efficiencies in these systems and higher PL efficiencies in (SO_2_), with no electron lone pairs on the bridging sulfur atom. In other words, the formation of CT states in (SO_2_) can suppress ISC and lead to higher Φ values. Hence, the studies do not maintain the expectation that a greater electron density on the sulfur atom should facilitate electronic communication between bridged chromophores [27]. The origin of the anomalously low emission of aromatic (S)/ (SO) and high emission of (SO_2_) has also been rationalized by Finney et al. [29] on two sets of unsymmetrical 1-pyrenyl (S)/(SO)/(SO_2_); their study established that the excited states of benzylic aryl sulfoxides (SO) were deactivated not by the photoinduced electron transfer from the sulfur lone electron pair, but by the non-radiative, reversible α-cleavage of the C-S bond [29,30]. Further studies on the origin of the non-radiative excited state relaxation in aryl sulfoxides, including excited state pyramidal inversion and alternate formation of a twisted intramolecular charge transfer state (TICT), in which the radical cation/radical anion pair is twisted fully out of conjugation, have recently been described in the literature [13,28,31].

The materials with the S(O)_2_ group lower both the LUMO and HOMO energy levels. The electronic devices with the sulfone-based materials can be expected to have: (1) an efficient electron-injection and transport, an improved balance of holes and electrons allowing their effective recombination in emissive layers; (2) a high triplet energy for the efficient blue phosphorescent organic light-emitting diodes; (3) wide band gaps; and (4) good electrochemical and thermal stabilities [2,5,31]. To date, the sulfone-based materials have also been used as host or emitting materials in phosphorescent organic light-emitting diodes (PhOLEDs) and efficient, thermally activated delayed fluorescence (TADF) emitting materials [1,2,3,4,5,6,7,8].

In one of the few papers on photostability and photooxidation stability of the SO_n_ bridged chromophores, Wolf et al. [31] reported that unsubstituted, symmetrical 9,9′-bianthryl sulfide (S) was stable in solution to irradiation at 365 nm for several hours, the sulfoxide (SO) underwent a rapid (seconds) loss of the bridging SO group followed by the quantitative formation of 9,9′-bianthryl, while the corresponding sulfone (SO_2_) underwent chemical reactions to give anthracene dimer containing a three-membered episulfone ring which, like the starting sulfone, could be converted to anthraquinone in the presence of oxygen upon overnight irradiation. There is a lack of studies on the photostability and photooxidation stability of these compounds at 254 nm.

In addition to the mentioned examples, a very limited number of other SO_n_ bridged chromophores have been reported to date [29,30,32]. Moreover, the lack of studies on the synthesis and photochemical behavior of bulky, SO_n_ (n = 0, 1, 2) bridged, non-symmetric chromophores, based on anthracene and larger acenes, is particularly noticeable in the literature. The present study fills this gap and investigates highly substituted (S), i.e., 10-RS-anthracenes, obtained in the *thio*-variant of the Friedel–Crafts-Bradsher cyclization [33,34], and the corresponding sulfoxides (SO) and sulfones (SO_2_).

## 2. Materials and Methods

### 2.1. Synthetic Procedures and Spectroscopic Techniques

*m*-Chloroperbenzoic acid (m-CPBA, Sigma-Aldrich, St. Louis, MO, USA) was used at ≥ 77%. Dry and oxygen free dichloromethane (DCM, JT Baker Chemicals, Deventer, The Netherlands) was taken from MB SPS-800 (MBRAUN Solvent Purification System, Garching, Germany). The ^1^H NMR and ^13^C NMR spectra (Figure S1) were measured with a Bruker AV 200 or AV 500 spectrometer, (Billerica, MA, USA) in CDCl_3_ with chemical shifts (δ) given in ppm relative to TMS as an internal standard. High-resolution mass spectrometry (HRMS) measurements were performed using Synapt G2-Si mass spectrometer (Waters, Milford, MA, USA) equipped with an APCI source and quadrupole-Time-of-Flight mass analyzer. The mass spectrometer was operated in the positive ion detection mode with discharge current set at 4.0 μA. The heated capillary temperature was 350 °C. The results of the measurements were processed using the MassLynx 4.1 software (Waters, Milford, MA, USA) incorporated with the instrument. Accurate mass measurements were performed by a peak matching technique using perfluorokerosene as an internal standard at a resolving power of 10,000 (10% valley definition). The HR(MS_MS)-(+)-APCI spectra were recorded at 15 eV, 25 eV and 35 eV. Melting points were measured using Boetius apparatus. Thin layer chromatography (TLC) was performed on precoated Merck 60 (F254 60, Darmstad, Germany) silica gel plates with fluorescent indicator, with detection by means of UV light at 254 and 360 nm. Column chromatography was done on Merck silica gel (Kieselgel 60, Darmstad, Germany, 230–400 mesh). The UV-Vis absorption spectra were recorded in 1 cm cuvettes on a Shimadzu UV-2700 spectrophotometer (Kioto, Japan) using two types of the light source: a D2 64604 deuterium lamp and a Wl L6380 halogen lamp (Kioto, Japan, 220—600 nm). Room temperature, steady-state emission spectra were obtained with the Horiba Jobin Yvon, Fluorolog-3 spectrofluorimeter (Glasgow, UK) using a xenon lamp as a light source. The fluorescence quantum yields Φ of the obtained compounds were determined in EtOH and toluene on excitation at their absorption maximum using an integrating sphere (Horiba, Jobin Yvon, Quanta-φ F-3029 Integrating sphere). Fluorescence lifetime values were measured by the Time Correlated Single Photon Counting (TCSPC, Glasgow, UK) analysis. The fluorescence decay curves were obtained by exciting the molecules at their emission maximum using a NanoLED laser at 374 nm ± 10 nm (<200 ps) as a light source. The photodegradation measurements were investigated by monitoring the absorbance decay of 10^−5^ ethanolic solutions in a quartz cuvette stored in the dark at room temperature, under ambient atmosphere (O_2_) and inert atmosphere (Ar), and then exposed to a UVP-Hg-Pen-ray lamp (254 nm, 16.33 mW/cm^2^ at distance 1 cm) and a VL-6.LC fluorescent lamp (6W) (365 nm, 27.4 mW/cm^2^ at distance 1 cm).

### 2.2. Photostability and Photooxidation Stability in Ethanol Solutions

The photodegradation was investigated by monitoring the absorbance decay of 10^−5^ M ethanolic solutions in quartz cuvettes stored in the dark at room temperature, under ambient atmosphere and Ar atmosphere, and exposed to a UVP-Hg-Pen-ray lamp (254 nm, 16.33 mW/cm^2^ at distance 1 cm) and a VL-6.LC fluorescent lamp (6W) (365 nm, 27.4 mW/cm^2^ at distance 1 cm).

### 2.3. HRMS-(+)-APCI Spectra

The photodegradation was investigated by monitoring the MS spectra of 10^−5^ M ethanolic solutions in quartz cuvettes at room temperature, under ambient atmosphere, after exposure to UVP-Hg-Pen-ray lamp (254 nm, 16.33 mW/cm^2^ at distance 1 cm) for 3 h (irradiation dose: 176 J/cm^2^) and 9 h (irradiation dose: 528 J/cm^2^).

## 3. Results and Discussion 

### 3.1. Chemical Synthesis

**Scheme 1:**
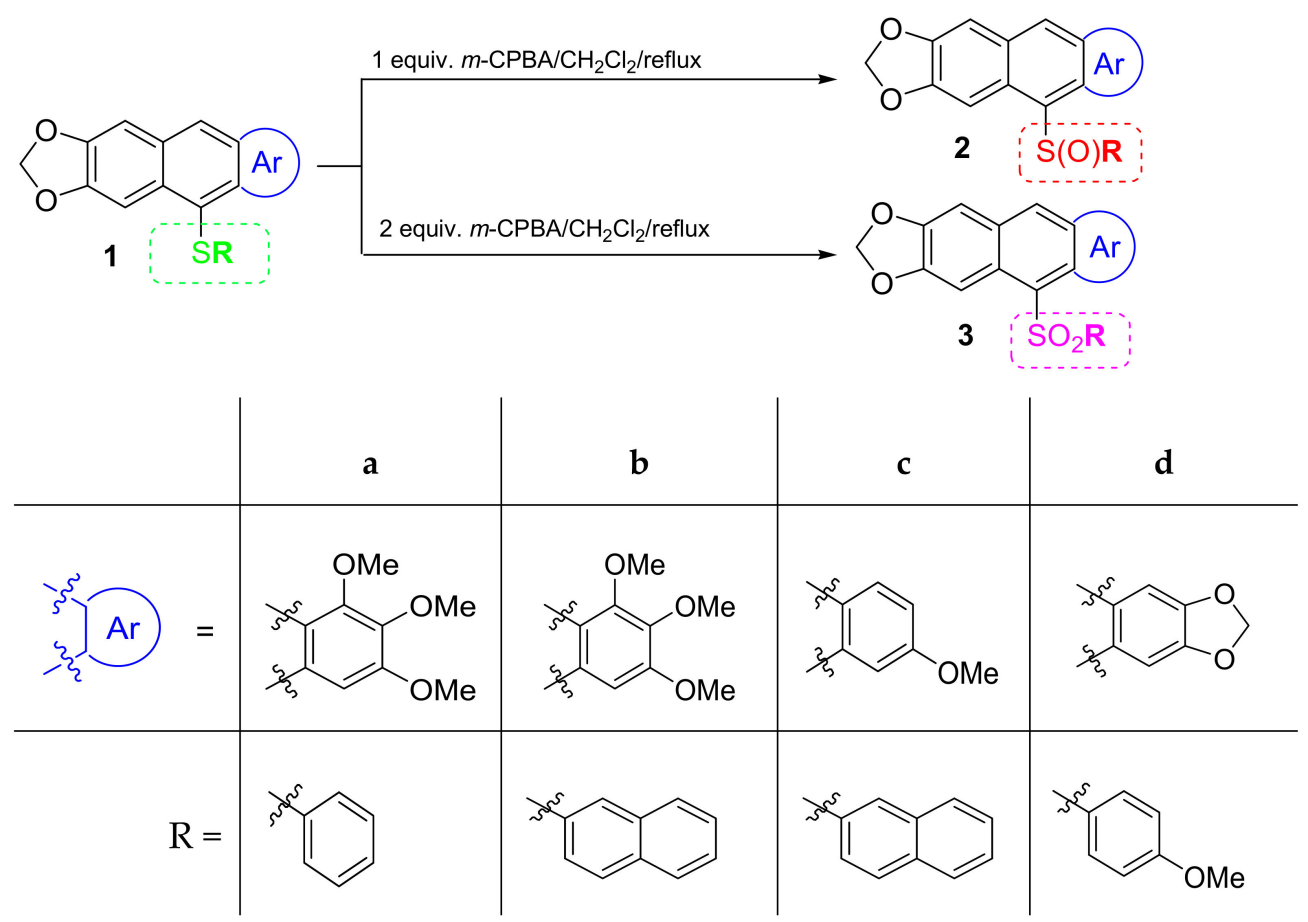
Oxidation reaction of (S), i.e., 10-RS-anthracenes **1** to the corresponding (SO)-**2** and (SO_2_)-**3**.

The (S) substrates, mono-RS-substituted anthracenes **1**, used for the synthesis of two series of mono-RS(O)- and mono-RS(O)_2_-substituted anthracenes ((SO) **2** and (SO_2_) **3**, respectively), were obtained from cyclisation of (*o*-*S,S*-dithioacetalaryl)arylmethyl thioethers in the presence of FeCl_3_/KI in boiling ethanol, according to the *thio-*Friedel–Crafts–Bradsher reaction, which has recently been elaborated in our lab [33,34]. Next, RS groups in (S)-**1a–d** were oxidized using one or two equivalents of *m*-chloroperbenzoic acid (*m*-CPBA) to give the corresponding (SO)-**2a–d** and (SO_2_)-**3c**, **3d**, respectively (Scheme 1 and Table 1). The oxidation reaction of **1a** and **1b** gave **2a** and **2b** in 55% and 42% yields, respectively. In this series, the corresponding sulfones **3a** and **3b**, obtained directly from the reaction of **1a** and **1b** with 2 equiv. of *m*-CPBA, although detected in small quantities in the crude reaction mixture (mass spectra), could not be isolated. An attempted synthesis of **3a** from the sulfoxide **2a** with 1 equiv. of *m*-CPBA, carefully monitored by ^1^H NMR for 5 days, resulted in the same outcome. The anthracene core in these two series has the highest electron donor character due to the presence of five alkoxy substituents and, most probably due to this reason, is unstable during oxidation. The oxidation of other sulfides **1c** and **1d** with 1 equiv. of *m*-CPBA gave sulfoxides **2c** and **2d** in 36% and 32% yields, respectively. In this series, synthesis of the corresponding sulfones **3c** and **3d** was successful and they were obtained from **2c** and **2d** with 2 equiv. of *m*-CPBA in 26% and 17% yields, respectively. 

### 3.2. Photophysical Properties

**Figure 1:**
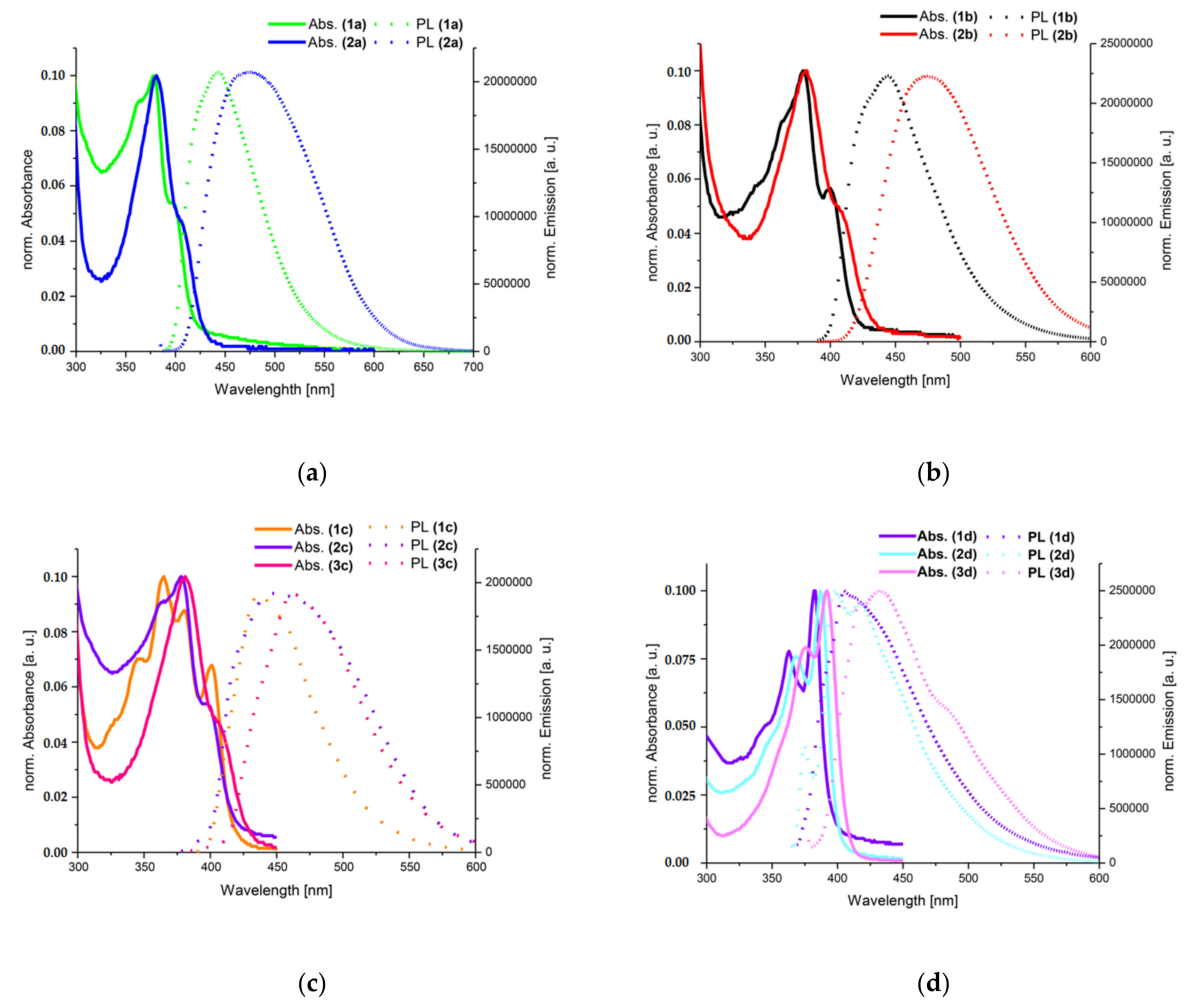
Absorption (Abs, continuous line) and photoluminescence (PL, dotted line) spectra of (**a**): (S)-**1a**, (SO)-**2a**, (**b**): (S)-**1b**, (SO)-**2b**, (**c**): (S)-**1c**, (SO)-**2c**, (SO_2_)-**3c**, (**d**): (S)-**1d**, (SO)-**2d**, (SO_2_)-**3d** in ethanol solutions (10^−5^ mol/L).

Photophysical properties (absorption and PL spectra, PL quantum yields (Φ) as well as PL lifetimes (τ) of (S)-**1a–d**, (SO)-**2a–d** and (SO_2_)-**3c** and **3d,** were measured in two solvents: polar ethanol (Table 2, Figure 1, Figures S2–S4) and non-polar toluene (Table 2, Figures S2, S5, and S6 in Supplementary Materials). The obtained results in the series of **2** and **3** were compared with the results obtained for **1**-RS. The absorption spectra in the series of **1a**-SPh/**2a**-S(O)Ph and **1b**-S-2-naphthyl/**2b**-S(O)-2-naphthyl, having identical configurations of alkoxy substituents at the anthracene core and different oxidation states of their sulfur atoms, showed the same absorption pattern in the range of 310–450 nm with only two absorption maxima for **1a** and for **1b** at $\lambda_{\max}^{\mathrm{abs}}$ = 380 and 400 nm, respectively, and one absorption maximum for **2a** and for **2b** at $\lambda_{\max}^{\mathrm{abs}}$ = 380 nm with a shoulder at 405 nm for **2a** and **2b**. In the two **a** and **b** series of (S) and (SO), with five alkoxy substituents on the anthracene core, the higher oxidation state of sulfur atom ((S) versus SO)) had, astonishingly, no effect on the position of the absorption maxima (**1a**/**2a** and **1b**/**2b**, $\Delta_{\max}^{\mathrm{abs}}$ = 0 nm), unlike in the **d** series with four alkoxy groups (**1d**/**2d**, $\Delta_{\max}^{\mathrm{abs}}$ = 3 nm) and in the **c** series with three alkoxy groups (**1c**/**2c**, $\Delta_{\max}^{\mathrm{abs}}$ = 19 nm) (Table 2, Figure 1). In the **c** and **d** series, the comparison could be more comprehensive due to the available data for (SO_2_)-**3c** and **3d**. Thus, in the case of **1c**-S-2-naphthyl, absorption spectra in ethanol solution showed five maxima at $\lambda_{\max}^{\mathrm{abs}}$ = 330, 348, 367, 383 and 403 nm, while in case of **2c**-S(O)-2-naphthyl, only three maxima at $\lambda_{\max}^{\mathrm{abs}}$ = 368, 386 and 407 nm, and in case of the **3c**- S(O_2_)-2-naphthyl, four maxima at $\lambda_{\max}^{\mathrm{abs}}$ = 328, 378, 395 and 416 nm in the range of 310–450 nm, were observed, respectively. Small bathochromic shifts for the absorption maxima $\lambda_{\max}^{\mathrm{abs}}$ were observed in the series of **1c**-S-2-naphthyl, **2c**-S(O)-2-naphthyl and **3c**- S(O_2_)-2-naphthyl, in ethanol solutions ($\lambda_{\max}^{\mathrm{abs}}$ = 365, 368 and 376), as in the series of **1d**-S-4-(MeO-C_6_H_4_), **2d**-S(O)-4(MeO-C_6_H_4_) and **3d**-S(O_2_)-4-(MeO-C_6_H_4_) ($\lambda_{\max}^{\mathrm{abs}}$ = 382, 387 and 392).

In contrast to the absorption spectra, where maxima positions were very similar both in the ethanol and toluene solutions, the PL spectra, in the series of RS-, RS(O)- and RS(O_2_)-substituted anthracenes, revealed emission red shifts both in ethanol (Figure 1) and toluene (SM, Figure S5). The spectra also showed relatively stronger dependence on the sulfur oxidation state, going from (S) to (SO) and from the latter to (SO_2_).

**Figure 2:**
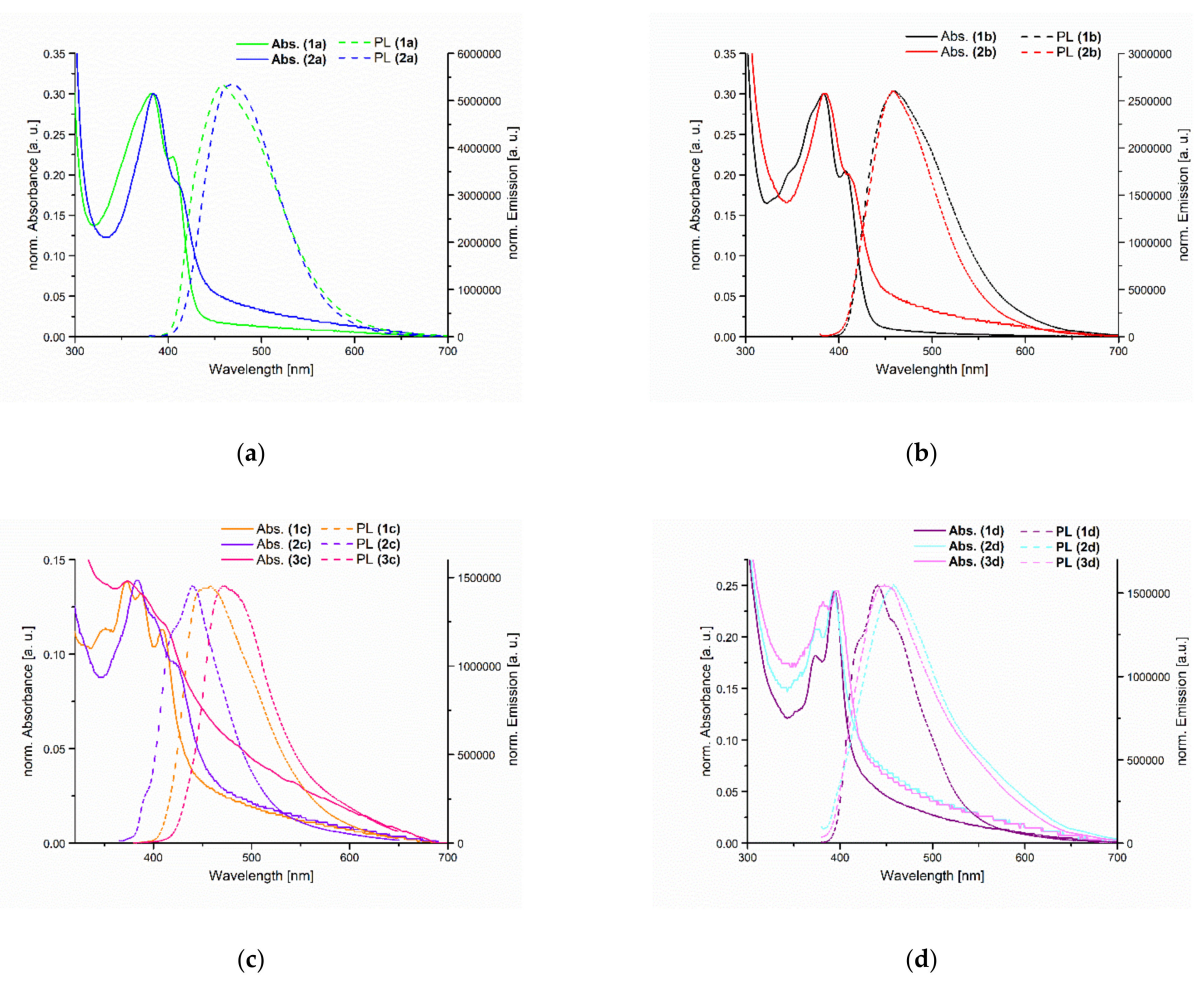
Absorption (Abs, continuous line) and photoluminescence (PL, dashed line) spectra for (**a**): (S)-**1a**, (SO)-**2a**, (**b**): (S)-**1b**, (SO)-**2b**, (**c**): (S)-**1c**, (SO)-**2c**, (SO_2_)-**3c**, (**d**): (S)-**1d**, (SO)-**2d**, (SO_2_)-**3d** in thin film cast from toluene solutions.

Fluorescence spectra of compounds **2a** and **2b** with 3-methoxy groups on the athracene moiety, both in toluene and EtOH, show large redshifts in relation to the spectra of **1a** and **1b** (Figure 1a,b and Figure S5). In contrast, in solid thin films, where conformational motions are limited relative to solutions, the spectra of **2a** and **2b** do not show such a shift and almost overlap (Figure 2, Figure S7). A similar increase can be seen for quantum yields and lifetimes, which are larger for sulfoxides than for sulfides (Table 2). These data provide arguments for the involvement of intramolecular CT processes in the excited state. Indeed, charge-transfer (CT) states have been observed previously for symmetrically substituted arenes bridged by sulfide at different oxidation levels [26].

For compounds **1c, 2c** and **3c**, with only one electron-donating methoxy substituent (EDG) on the methylene-1,3-dioxo-anthracene backbone (Figure 1c), a similar tendency for the fluorescence spectra to redshift with the degree of oxidation state is observed, although the absorption spectra reveal the vibronic structure of anthracene to a greater extent than in the case of **1a,b** and **2a,b**. The greatest shift shows the spectrum of **3c**, while the spectrum of **2c**, being broader, covers both the **1c** and **3c** fluorescence spectra, i.e., it contains localised emission from the anthracene molecule and long-wavelength emission from the CT state.

The observed quantum yields and fluorescence lifetimes of **1c** and **2c** show a similar increasing trend as for pair **1a**-**2a** and **1b**-**2b**, i.e., they are higher for sulfoxide analogues than for the sulfide ones. The sulfone derivative does not show the largest increase in φ and τ as in the case of symmetric chromophores [26]. On the contrary, a significant decrease in these parameters is observed for both studied solvents. Additionally, **3c** in EtOH has a bi-exponential fluorescence decay.

For the symmetric bis(methylene-1,3-dioxo)-anthracene moiety, the decreases in quantum yields and lifetimes are more drastic (Table 2). There is no clear redshift of the fluorescence bands associated with expected increasing contribution of CT transitions in the fluorescence process due to oxidation of the sulfide bridge. The emission decays are 2- or 3-exponential with the slow components below 5.7 ns (for EtOH) and 9.5 ns (for toluene). This may indicate the presence of two or three chromophore populations in the excited state due to, among other factors, conformational strain of the five-membered methylene-1,3-dioxolane ring. Then, the non-radiative processes start to play an essential role in the deactivation of the excited state, as well as significantly reducing quantum efficiency.

In conclusion of this section, it should be stated that: (1) with the degree of oxidation of the sulfide bridge, the vibronic character of the anthracene absorption band disappears; (2) no increase in quantum yields as a function of bridge oxidation was observed as for symmetric arenes [26], but a clear increase was observed for sulfoxide derivatives; and (3) EDG substituents on the anthracene backbone have a significant influence on the photophysical processes. Their amount and size facilitate the participation of intramolecular CT processes in both polar and non-polar media.

A similar trend concerning a stronger dependence on the sulfur bridge oxidation was observed with Stokes shifts in both investigated solvents (toluene/ethanol: **1a**-3840/3760 cm^−1^; **2a**-4900/5240 cm^−1^ nm), (toluene/ethanol: **1b**-3940/3860 cm^−1^; **2b**-4480/5350 cm^−1^) and (toluene/ethanol: **1c**-4720/4510 cm^−1^; **3c**-4720/5140 cm^−1^), which illustrates the increasing interactions of excited polar molecules (from (S) to (SO_2_)) with solvents of the increasing polarity (from toluene to ethanol). Indeed, in these series, the highest Stokes shifts values (**2a**, **2b** and **3c**) were higher in ethanol (5240, 5350, 5140 cm^−1^) than in toluene (4900, 4480, 4720 cm^−1^), respectively (Table 2). They are generally recognized as moderate. Inconsistent with this trend, very low Stokes shifts were found in series **c** and **d**: in (SO)-**2c** (3280 cm^−1^) and **2d** (650 cm^−1^) in toluene, and in the (SO)-**2d** (710 cm^−1^) in ethanol. In addition, these values were unexpectedly lower than in the corresponding (S) (4520 for **1c** in toluene, 2460 for **1d** in toluene and 1490 cm^−1^ for **1d** in ethanol, respectively). In the solid samples **1**, **2** and **3**, cast as thin layers (Table 3), the Stokes shifts slightly increased in comparison to the toluene solutions of (S)-**1a–d** by 240–480 cm^−1^, of (SO)-**2c** and **2d** by 750–2960 cm^−1^ and of (SO_2_)-**3c** by 250 cm^−1^ with exception of (SO_2_)-**3d** for which the Stokes shift decreased. The increase in the Stokes shift values is due to greater intermolecular interactions in solids which is a common phenomenon. The results of optical measurements in solutions (ethanol and toluene) and in thin film casts from toluene are summarized in Figure 1 and Figure 2, Table 2 and Table 3, and in SM (Figures S2–S4). 

In Table 2, we present absorption and photoluminescence maxima (λ_max_), Stokes shifts, photoluminescence quantum yields (Φ) and lifetimes (τ) in solution (toluene and ethanol) of **1a–d**, **2a–d**, **3c** and **3d**. Thus, in toluene solutions, the following quantum yields were measured: 0.63–31.5% for (S)-**1a–d**, 1.9–55.6% for (SO)-**2a–d** and only 2.0–10.0% for (SO_2_)-**3c**, **3d**, in contrast to higher efficiencies recorded in the literature for symmetrical (SO_2_) [29]. The highest values of 58.2% (in ethanol) and 55.6% (in toluene) were measured for **2a**-S(O)Ph and **2b**-S(O)-2-naphthyl, respectively. In contrast to our results concerning unsymmetrical chromophores, Wolf et al. [26] found in symmetrically bridged chromophores that Φ was significantly enhanced by the successive oxidizing the sulfur bridge from (S) to (SO), and further to (SO_2_). They showed that previously studied unsymmetrical (SO) and (SO_2_) exhibited different photophysical behavior and in some cases, (SO) were less emissive than the corresponding (SO_2_), which was attributed to non-radiative pathways including α-cleavage/recombination and/or pyramidal inversion in sulfur. In other cases, the (SO_2_) were less emissive, such as in our studies [33,34]. 

The room-temperature PL lifetimes (τ) of compounds in series of **1–3** were dependent on the oxidation state of the bridging sulfur. The lifetimes in the (S), (SO) and (SO_2_) series were measured in ethanol and toluene. The lifetimes on the level of several nanoseconds in the series **a–d** were longer in polar ethanol than in non-polar toluene. In general, the lifetimes of unsymmetrical (SO)-**2a–d** (toluene/ethanol: τ = 4.06–8.29 ns/5.47–15.28 ns) were longer than the corresponding (S)-**1a–d** (toluene/ethanol: τ = 1.94–5.43 ns/2.10–6.08 ns). The (SO_2_)-**3c** (toluene/ethanol: τ = 0.98 ns/3.80 ns) possessed the shortest lifetimes in both solvents. Moreover, in the series **c** and **d** (**1c**, **1d**, **2c**, **2d** and **3c**, **3d**), more than one lifetime was measured. For (SO_2_)-**3d**, three τ-components were recorded, of which two were very short (toluene/ethanol: τ = 0.47, 3.98 ns/0.16, 0.77 ns) and one relatively very long (toluene/ethanol: τ = 9.46/5.64 ns). For the symmetrically bridged (S), (SO) and (SO_2_) chromophores, Wolf et al. [31] showed that all of the compounds exhibited prompt lifetimes (<1 ns), while (SO) and (SO_2_) compounds had multi-exponential lifetimes with a substantially longer (about 30 ns) component, which was two-fold greater than the longest lifetimes, reported for **2a** and **2b** in the whole **a–c** series of unsymmetrically bridged chromophores (Table 2). It has been found in the literature that the longest-lived component for symmetrical SO_n_ bridges increases with increasing oxi- dation state of the bridging sulfur atom, in the following order: (S) < (SO) < (SO_2_); this occurs under both air and argon [31]. The same order was observed in our case for the **d** series of unsymmetrical compounds, however with a much greater dispersion in the lifetime values. A comparison of the longest-lived components in the **c** series, showed a different order: (SO_2_) < (S) < (SO); this may be due to a lower symmetry of the unsymmetrically substituted anthryl core in comparison to the fully symmetrical one in the **d** series. 

The absorption spectra of (S)-**1a–d**, (SO)-**2a–d** and (SO_2_)-**3c**, **3d** in thin films are presented in Figure 2 and their optical properties are summarized in Table 3. In thin films, lower-energy absorption bands are redshifted and broadened, compared with absorption bands in solution, which is attributed to the stronger intermolecular interactions in solid state. The positions of the absorption maxima for (S)-**1** and (SO)-**2** were practically identical, while the maxima for (SO_2_) were redshifted by 4–10 nm in comparison to **1** and **2**. In PL spectra, slightly higher redshifts of 10–17 nm were observed for pairs of (S)/(SO)-**1a**/**2a** and **1d**/**2d**, respectively, while for the pairs of **1b**/**2b** and **1c**/**2c**, a blueshift of 2–19 nm was found.

### 3.3. Structural Determinants of the CIE 1931 Chromaticity Coordinates

**Figure 3:**
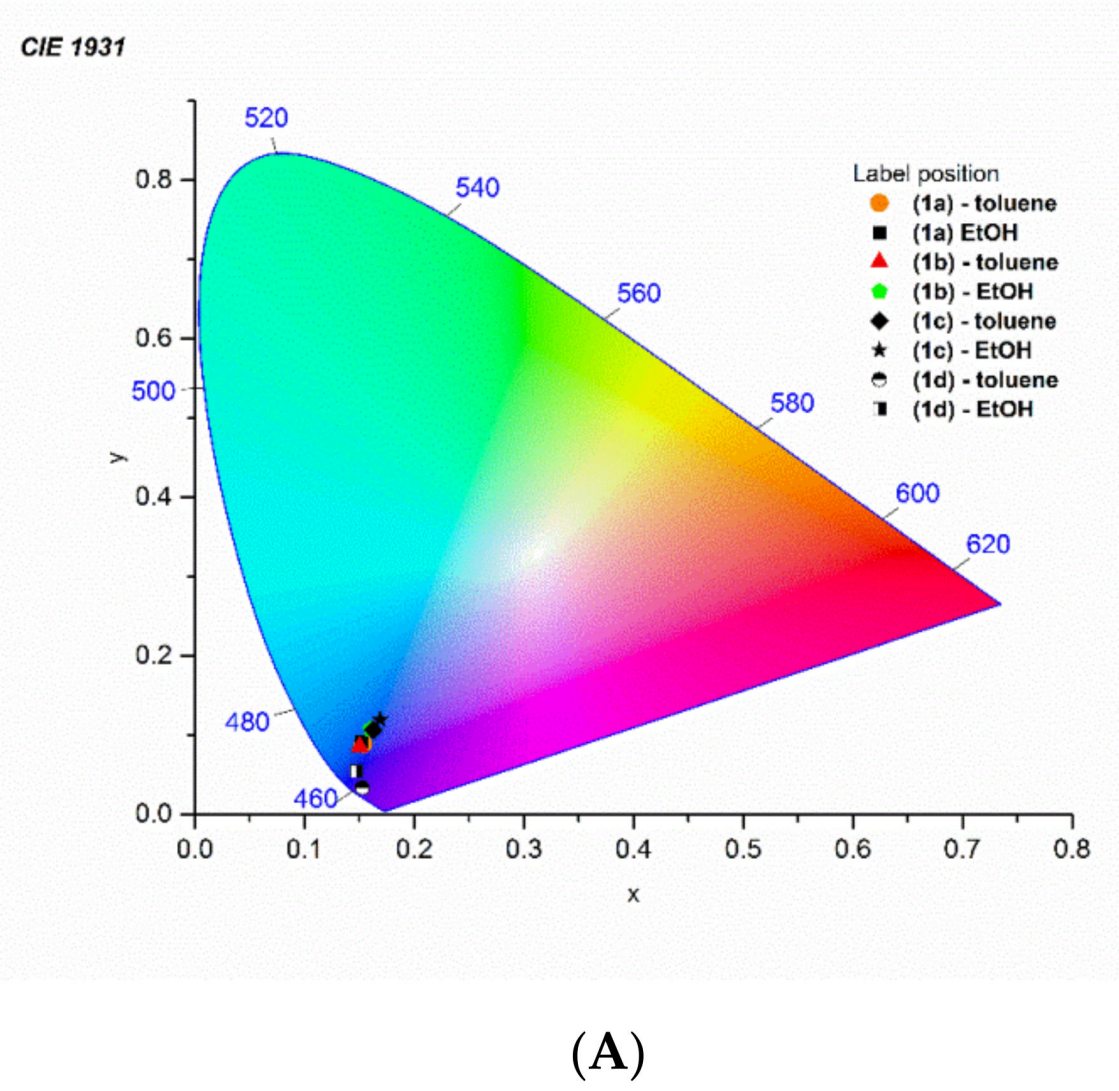

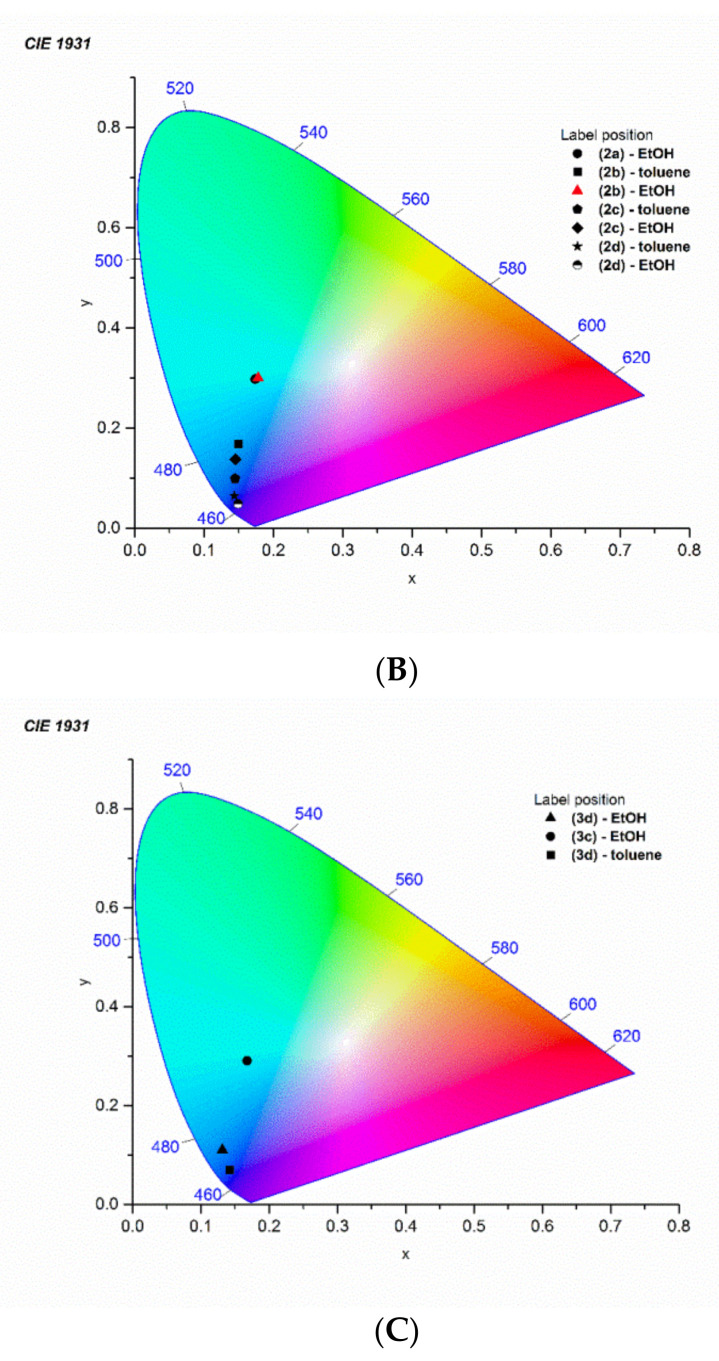
(**A**) The CIE 1931 chromaticity coordinates for (S)-**1a–d** in ethanol and toluene solutions; (**B**) The CIE 1931 chromaticity coordinates for (SO)-**2a–d** in ethanol and toluene solutions; (**C**) The CIE 1931 chromaticity coordinates for (SO_2_)-**3c**, **3d** in ethanol and toluene solutions.

Table 4 shows the CIE 1931 chromaticity coordinates for (S)-**1a-1d**, (SO)-**2a-2d**, (SO_2_)-**3c** and **3d** in toluene and ethanol solutions. The CIE 1931 coordinates for the luminescent materials on the plane of color spaces define the relationship between emitted wavelengths of light and physiologically recognized by humans. The (S)-**1a–d** emitted blue light both in toluene and ethanol solutions. Only coordinates of the light emitted by the symmetrical (S)-**1d**, possessing four alkoxy substituents and the 4-methoxy-phenylsulfenyl group on the anthracene core, were slightly shifted towards blue-violet part of the CIE 1931 color space chromaticity diagram (Table 2, Figure 3A). 

Oxidation of (S)-**1a–d** caused a significant, sulfur oxidation state dependent effect, and chromaticity coordinates dispersion from deep blue and blue through blue-green to green color was observed in the resulting (SO)-**2a–d** (Table 2, Figure 3B). The sulfoxide group S(O), which may form a hydrogen bond with ethanol, caused a further dispersion in chromaticity coordinates for **2a–c**, in relation to non-polar toluene. Only in the case of (SO)-**2d**, a weaker effect of the solvent polarity on wavelength of the emitted blue light was observed. In the series of (SO_2_)-**3c**, **3d**, the distribution of the coordinates was similar as in the group of (SO), and again in **3d**, with electron-donating 4-methoxyphenyl and four alkoxy-anthryl groups around the SO_2_ bridge, a weaker effect of the solvent polarity was observed (Table 2, Figure 3C). This confirms previous observations made for the series of (S)-**1** and (SO)-**2** that the increasing number of electron-rich groups at the anthracene core is the main factor responsible for color shifting of the emitted light towards the blue or blue-violet part of the spectrum.

### 3.4. Photochemical Stability

**Figure 4:**
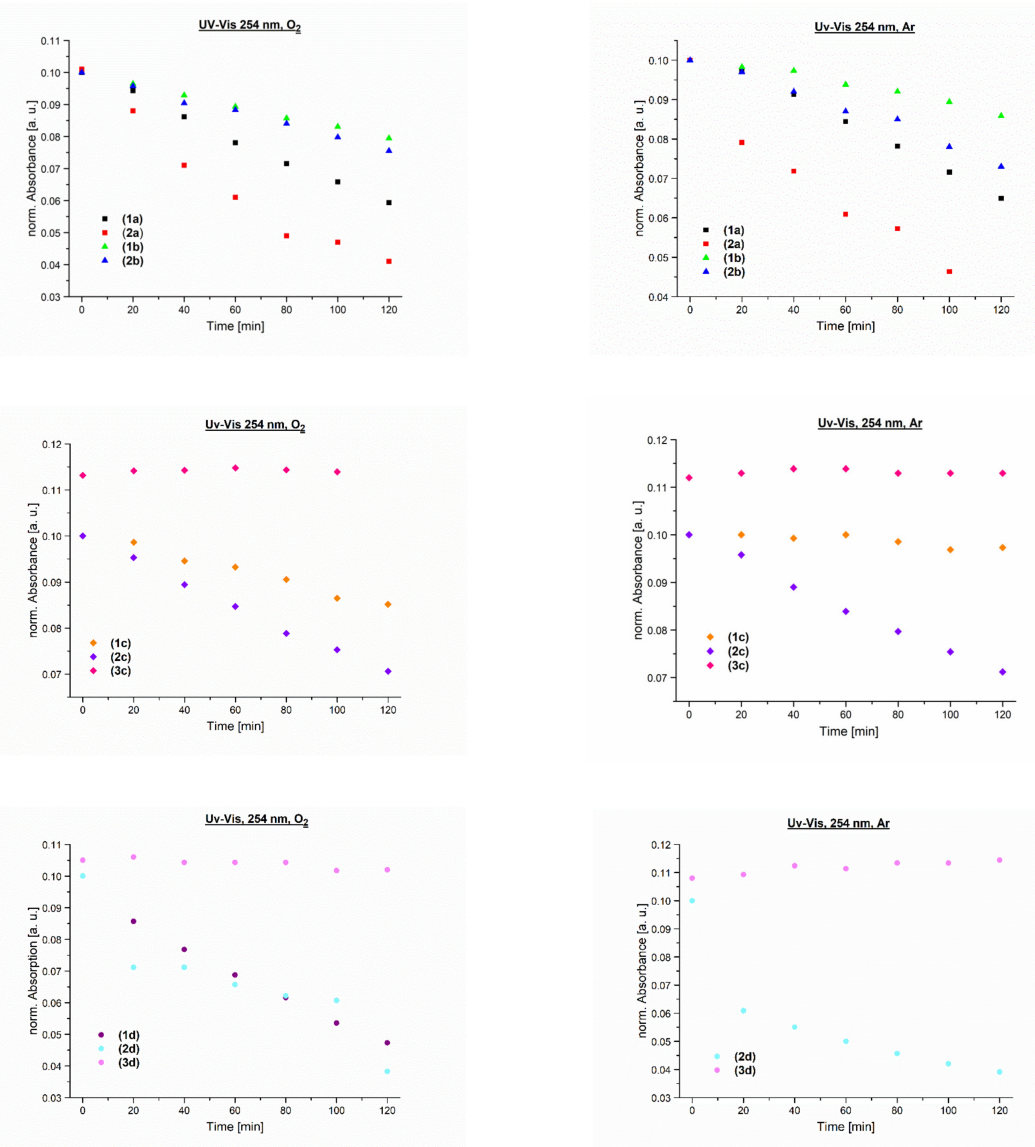
Evolution of the lower energy band absorptions of (S)-**1a–d**, (SO)-**2a–d** and (SO_2_)-**3c**, **3d** (in 10^−5^ M concentrations) in EtOH, under UV exposure (254 nm) at room temperature under ambient (O_2_) atmosphere (figures on the left) and under inert (Ar) atmosphere (figures on the right).

Optical materials should have a high photochemical stability; therefore, all three series of (S)-**1a–d**, (SO)-**2a–d** and (SO_2_)-**3c**, **3d** were exposed to light under anaerobic and aerobic conditions, in ethanol solutions, using two different UV wavelengths of 254 and 365 nm. Photostability under anaerobic conditions and photooxidation stability under aerobic (ambient) conditions were examined by monitoring the 50% loss of absorbance intensity of 10^−5^ M ethanol solutions of samples **1–3**, first stored in the dark at room temperature, under inert (Ar) or ambient (O_2_) atmosphere, and next exposed to the UV light of 254 nm (UVP-Hg-Pen-ray lamp, 16.33 mW/cm^2^ at a distance 1 cm) and 365 nm (fluorescent lamp VL-6.LC (6W), 27.4 mW/cm^2^ at a distance 1 cm). The actual irradiation dose (J/cm^2^) may be calculated by multiplication of the latter (W/cm^2^) by exposure time (sec.). Both (S)-**1**, (SO)-**2** and (SO_2_)-**3** revealed enhanced photochemical stability in solution during UV-irradiation at both 254 and 365 nm. In the series of significantly substituted chromophores **1** and **2**, the photooxidation stability in the range of 80–249 min. was measured at 254 nm under ambient atmosphere (O_2_), and even higher photostability in the range of 88–519 min. was found at 254 nm under inert atmosphere (Ar), which turned out to be the highest stability values reported for the π-extended (SO) and (SO_2_) (Table 5, Figure 4). Surprisingly, there were no significant differences in the 50% loss of absorbance between aerobic and anaerobic conditions at 254 nm for the tested series of (S)-**1a–d** and (SO)-**2a–d** (e.g., **1a**: 254 nm-166 (O_2_)/175 (Ar) min.; **2a**: 254 nm-80 (O_2_)/88 (Ar) min.), and for **2c** and **2d** at 254 and 365 nm (e.g., **2c**: 254 nm (O_2_)/254 nm (Ar)/365 nm (O_2_) = 247/245/254 min.). Only in case of (S)-**1b**, a significant two-fold increase in photostability was observed at 254 nm under inert versus ambient atmosphere (**1b**: 254 nm-249(O_2_)/519 (Ar) min). Another (S)-**1d** showed the highest photostability at 254 nm (Ar, absorption decay not observed) and photooxidation stability at 365 nm (O_2_, 812 min.) (Table 5).

A further detailed analysis shows that the photostability and photooxidation stability of (SO)-**2a**, **2b** and **2d** are lower than the corresponding (S)-**1a**, **1b** and **1d**, both under ambient and inert atmosphere, but are still much higher than the values, reported in the literature, of unsubstituted, symmetrical 9,9′-bianthryl analogs [31]. Only the (SO)-**2c** was slightly more stable under ambient atmosphere at 254 nm than its (S) precursor **1c** (**2c**/**1c**-247 min./210 min.). The highest stability at 254 nm among the (SO) family presented **2b** and **2c** (251, 245 nm, respectively) with the bulky naphthyl substituent at the S(O) bridge. The compounds (S)-**1a–c** and (SO)-**2a**, **2b** deserve a special mention due to their extreme photooxidation stability at 365 nm under ambient conditions, which has not been reported in the literature so far. A similar, very high stability showed (SO_2_)-**3c** and **3d** at 365 nm under the same conditions, for which the absorbance decay was not observed as well (see SM, Figure S8). 

Analysis of the UV/Vis spectra of (S)-**1a-1d** and (SO)-**2a-2d** (254 nm, air O_2_/Ar) showed a presence of several isosbestic points, which indicated the co-existence in solution of the starting materials and photo-products. For **1b**-**1d** and **2a**, **2b** (365 nm, air O_2_), isosbestic points were not observed, as was also the case for all (SO_2_)-**3** both at 254 and 365 nm (air O_2_/Ar). For **1a**, a weak 50% absorbance decay was observed and only two isosbestic points in a lower part of the UV spectrum could be detected (see SM, Figure S7). 

The HRMS-(+)-APCI analysis of the crude reaction mixtures obtained by irradiation of ethanolic solutions of **1–3** for 3 h (air O_2_, 254 nm, Hg lamp, irradiation dose: 176 J/cm^2^), did indeed confirm the formation of the oxidized, deoxidized or degraded products, which accompanied the starting acenes, as minor components of the mixtures (see SM, Figures S9–S15).

The characteristic feature for all starting acenes **1–3** was the maintenance of the integrity of anthracene rings without the ring C-C bonds’ cleavage. However, in case of (S) **1,** aromatic character of the products was destroyed by the oxidation of one or two peripherial or middle ring carbon atoms to ketone groups, while the aromatic character of (SO)-**2** and (SO_2_)-**3** was preserved. Thus, in the HRMS-(+)-APCI spectra of (S)-**1a**, **1b** and **1d**, two main groups of photo-products were found in the mixtures, which originated from: (1) the addition of oxygen to anthracene rings to initially form endoperoxides or hydroperoxides (not detected), which were subsequently cleaved to phenolic derivatives and further oxidized to 10-ketones or 1,4-anthraquinones; (2) the oxidation of sulfur atoms to sulfoxide [33]. Contrary to (S)-**1**, (SO)-**2a**, **2d** and (SO_2_)-**3c**, **3d** preserved aromatic character of photo-products after the 3h irradiation at 254 nm under air O_2_ in EtOH. Thus, (SO)-**2a** was photo-deoxygenated to the corresponding (S)-**1a**, as the main product. This unexpected reduction that occurred under photochemical, aerobic conditions, has earlier been observed for aromatic sulfides by Posner and Gurria under nitrogen atmosphere [35]. 

According to the authors’ suggestions, the reaction most probably occurred via the triplet excited state. Interestingly, reoxidation of (S)-**1a** back to (SO)-**2a** and a cleavage of the Ar-S bond were observed to be the main pathways after prolonged contact with air O_2_ and irradiation of the sample for 9 h under the same reaction conditions (254 nm, EtOH, irradiationdose of 528 J/cm^2^). On the other hand, (SO)-**2d** underwent a cleavage of both An-S and Ar-S bonds to a minor extent. After prolonged irradiation for 9 h, the fragmentation of **2d** slightly deepened; however, the parent peak remained as the main one in the spectrum, which meant that there was still a good stability of this compound after a three-fold increase in the irradiation time. Sulfones (SO_2_)-**3c**, **3d**, for which isosbestic points were not observed in UV spectra, were stable under the irradiation conditions (3 h, 254/365 nm, air O_2_/Ar, EtOH). The HRMS-(+)-APCI spectra of (SO_2_)-**3c**, **3d** were almost identical before and after irradiation, with the main fragmentation leading to desulfonylated species derived from **3c**, **3d** due to the An-S cleavage. The predominant fragmentation of An-S bonds over Ar-S ones in **3c** and **3d** may be explained by a relatively large bond lengths and a large An-S /Ar-S difference in DFT calculated bond lengths of the optimized molecules (An-S/Ar-S: **3c**-1.82954/1.81225 Å; **3d**-1.83695/1.80460 Å). For a comparison, the corresponding bonds in **1** are shorter and stronger, and the An-S /Ar-S difference is negligible (**1c**-1.79759/1.79689 Å; **1d**-1.79941/1.79978 Å). After prolonged exposure to the UV light at 254 nm for 9 h, both sulfones **3c** and **3d** showed still a good photooxidation stability with the increased cleavage of the Ar-S bond. In order to distinguish fragmentation peaks from the peaks due to the photo-products in the irradiated mixture, a HR(MS_MS)-(+)-APCI fragmentation of single peaks, in the whole spectra, was carried out to conclude that the detected peaks for all investigated compounds were due to the real photoproducts in the mixture.

The stability to photooxidation of the tested compounds **1–3** upon exposure to the UV light at 365 nm (air O_2_) was obviously much higher than the stability upon exposure to the UV light at 254 nm and no isosbestic points were detected for these compounds in UV spectra.

Due to the lack of broader investigations on photostability and photooxidation stability of SO_n_ (n = 0, 1, 2) bridged chromophores, the stability of the investigated compounds in this study may be compared with photooxidation results obtained at 365 nm for the unsubstituted 9,9′-bianthryl (S), (SO) and (SO_2_) [31], which are structurally the most similar compounds to the series of significantly substituted mono-anthryl derivatives **1**, **2** and **3**. While the high photooxidation stability (hours) at 365 nm of the 9,9′-bianthryl (S) was comparable with the high stability of **1**, the stability of **2** was many times greater than the stability (seconds) of the 9,9′-bianthryl (SO) [31]. The (SO_2_)-**3** were similarly very stable, while the 9,9′-bianthryl (SO_2_) underwent chemical reactions (first the episulfone and then the anthraquinone formation) [31]. To our knowledge, unsymmetrical, significantly substituted **1**-, **2**- and **3**-SO_n_ bridged chromophores, obtained by electron-donating alkoxy substituents, are the most stable sulfur compounds of this kind reported to date. At the same time, a lack of photostability and photooxidation stability tests of such compounds at 254 nm should be noted.

### 3.5. DFT Calculations of Molecular and Electronic Structures of 1, 2 and 3. Influence of Sulfur Oxidation State on Stabilization of Frontier Orbitals

**Figure 5:**
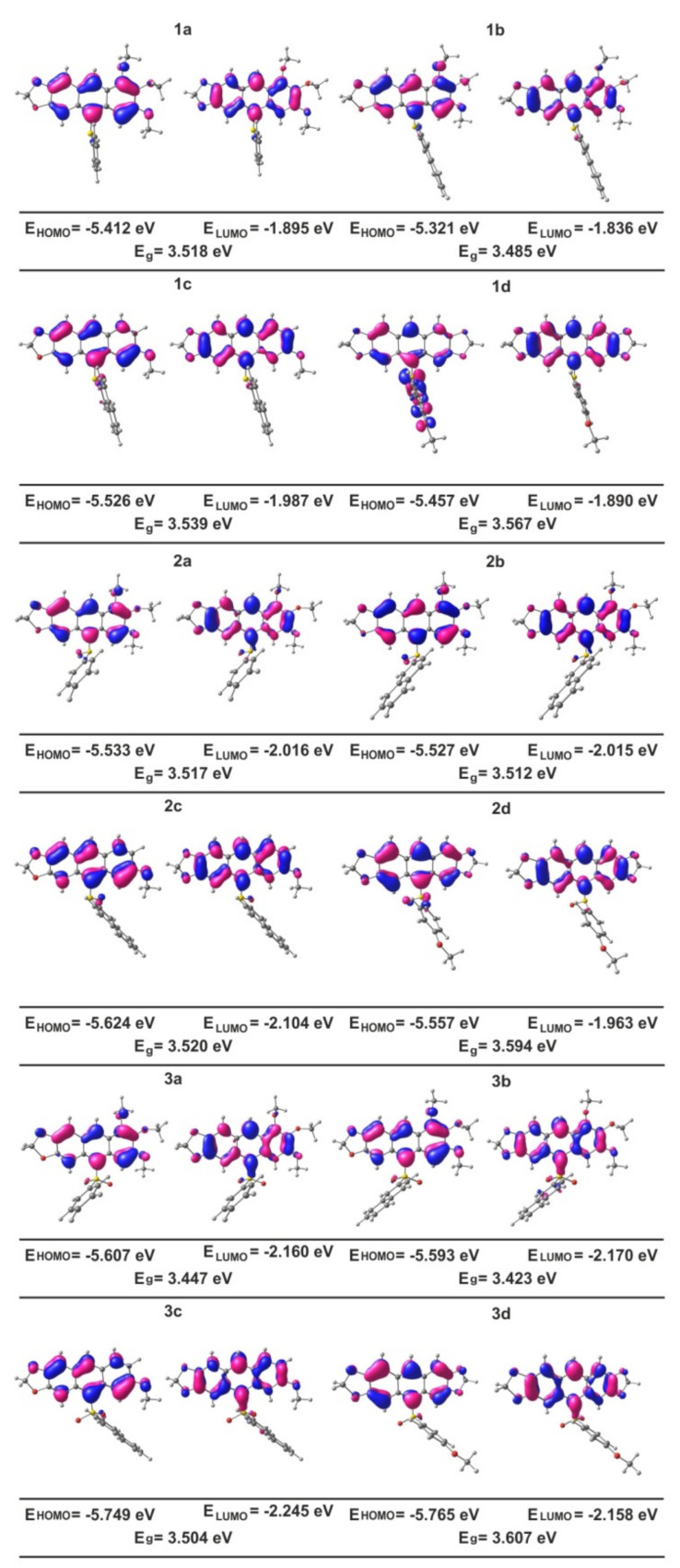
Plots of the HOMO and LUMO orbitals for molecules **1a–d**, **2a–d** and **3a–d** optimized according to the DFT theory B3LYP/6-311++G(d,p).

The above studies were supported by DFT calculations of molecular and electronic structures of (S)-**1a–d**, (SO) -**2a–d** and (SO_2_)-**3a–d** in the gas phase, and at the ground state, using the gradient corrected three-parameter hybrid function (B3LYP) with the 6-311++G(d,p) basis set. Full geometry optimizations of these compounds were performed using the GAUSSIAN 09 quantum chemistry package (coordinates of atoms for all optimized geometries are given in Tables S1–S12). Analysis of shape and values of the HOMO-LUMO energy was carried out for twelve RS-, RS(O)- and RS(O_2_)-acene derivatives, organized in three **a–c** series. (Figure 5). 

**Figure 6:**
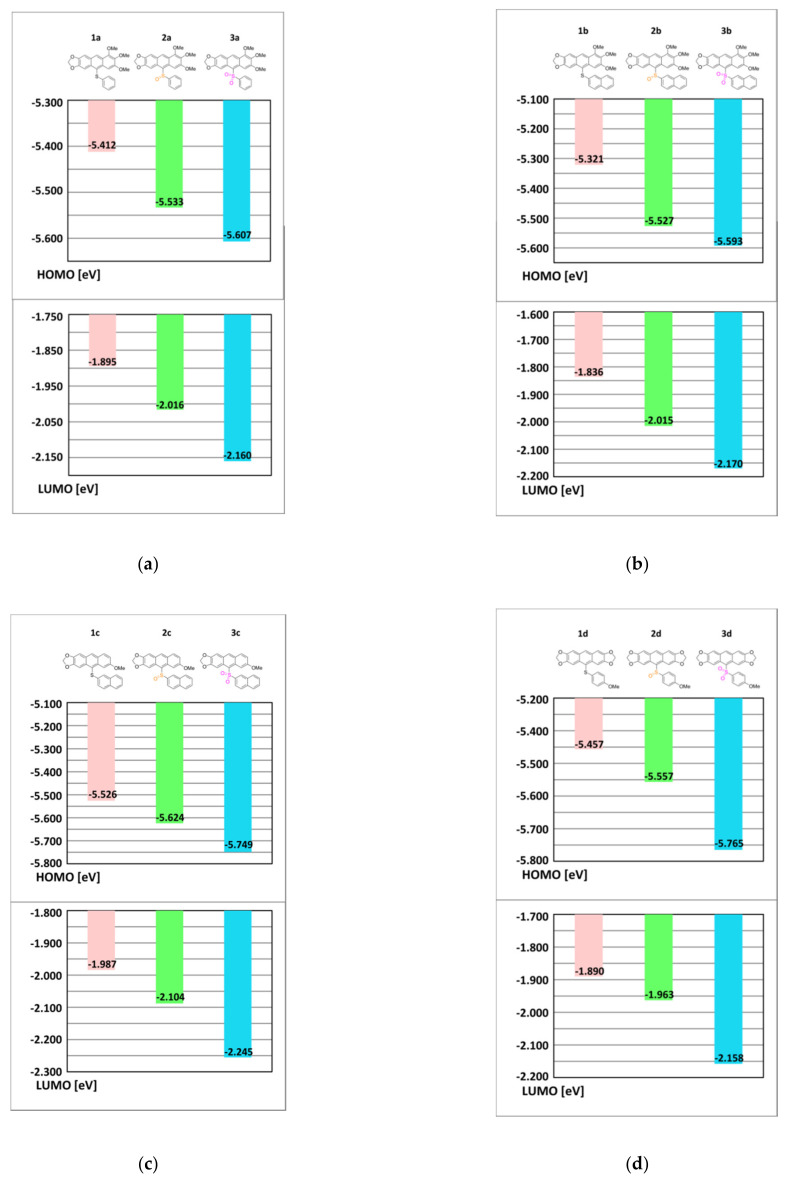
Graphs illustrating the changes in the HOMO and LUMO energy values for the series of molecules **1a**, **2a** and **3a** (**a**), **1b**, **2b** and **3b** (**b**), **1c**, **2c** and **3c** (**c**) **1d**, **2d** and **3d** (**d**) optimized according to the DFT theory B3LYP/6-311++G(d,p).

The analysis showed that HOMO and LUMO orbitals, for each molecule, were mainly located on the system of fused aromatic rings with exception of **1d**, for which the HOMO orbital was located also on the 4-methoxyphenylthio group. A comparison of the HOMO and LUMO energy values (Figure 6a), calculated for **1a**-SPh, **2a**-S(O)Ph and **3a**-S(O_2_)Ph, indicated that oxidation of the PhS-group to PhS(O) (in **2a**) resulted in a significant decrease and stabilization in both HOMO and LUMO energy values from −5.412 eV to −5.533 eV, and from −1.895 eV to −2.016 eV, respectively. A replacement of the PhS(O) for the PhS(O_2_) group (in **3a**) caused a further orbital stabilization and decrease in the HOMO and LUMO energies to −5.607 eV and −2.160 eV, respectively. The same trend was observed in all three **a–c** series of compounds (Figure 6a–d). The decrease and stabilization in the HOMO and LUMO energies are correlated well with the increase in the sulfur oxidation degree in the following order: (S) < (SO) < (SO_2_); in addition, the electron-acceptor character of the SO_n_ bridge increased in the same order. The measure of the latter effect is the Hammett σ_p_ constant. For instance, the σ_p_ values calculated using ACD/Percepta [36] were 0.07, 0.44 and 0.68 for PhS, PhS(O) and PhS(O_2_) substituents, respectively, and 0.13, 0.39 and 0.77 for 4-MeOC_6_H_4_-S, 4-MeOC_6_H_4_-S(O) and 4-MeOC_6_H_4_-S(O_2_) substituents, respectively. The small but positive values of σ_p_ constants confirm a weak electron-acceptor character of the RS groups in all cases, when attached to electron-rich anthracenes substituted by five (**a**, **b** series), four (**d** series) and three (**c** series) electron-donating alkoxy groups [16]. Analysis of the σ_ind_ and σ_res_ components shows that a mostly negative inductive effect (-I) is responsible for the electron-withdrawing character of the RS-groups in the electron-rich aromatics (e.g., SPh: σ_ind_/σ_res_ = 0.31/−0.24; S-Naphth: σ_ind_/σ_res_ = 0.3/−0.15). The (-I) effect dominates, in all cases (SO_n_), over the resonance effect (+M), which gradually decreases in the oxidized sulfur chromophores and finally changes to the (-M) effect in (SO_2_) (e.g., S(O)Ph: σ_ind_/σ_res_ = 0.51/−0.07; S(O_2_)Ph: σ_ind_/σ_res_ = 0.56/0.12). For a comparison, for RO groups, the (+M) effect dominates over the (-I) effect (PhO: σ_ind_/σ_res_ = 0.4/−0.48 and MeO: σ_ind_/σ_res_ = 0.30)/−0.58). Thus, all three **a–c** series are donor–acceptor chromophores, in which the electron-accepting properties are associated with the operation of the inductive effect (-I) caused by RS(O)_n_ groups (weaker for RS and stronger for RS(O) and RSO_2_), and the electron-donating properties are connected with the operation of the positive resonance effect (+M) of alkoxy substituents. The final conclusion is that the observed high photochemical stability of **1–3** comes from the lowering of the LUMO energy level. The lower LUMO makes the compounds more resistant to photooxidation, which usually occurs via electron transfer (Type-I) or/and energy transfer (Type-II) mechanisms [37,38].

## 4. Conclusions

The most striking result obtained in this study is related to the very high, hitherto unrecorded photostability and photooxidation stability of the significantly ring substituted acenes, (S), (SO) and (SO_2_), under both anaerobic and aerobic conditions, at 254 and 365 nm. These stabilities are many times higher than stabilities under similar conditions of less sterically hindered or unsubstituted analogs that are known in the literature, which makes them promising materials for optoelectronics, photocatalysis and other areas requiring photostable compounds. The high photochemical stability is associated with the effect of the sulfur oxidation state and a significant substitution at the aromatic core by electron-donating groups. This leads to the formation of stable donor–acceptor systems, in which a negative, inductive effect (-I) from the RS(O_n_) groups is responsible for electron-accepting properties, and the (+M) effect of alkoxy substituents is due to electron-donating properties (Hammett’s constants, *vide supra*). The enhanced photooxidation stability (hours) of the obtained, unsymmetrical, significantly substituted (SO) was particularly striking, compared to the literature photooxidation stability results (seconds) in symmetrical, unsubstituted bianthryl (SO) at 365 nm [31]. The high stabilities in the obtained (S), (SO) and (SO_2_) series was supported by DFT calculations, which revealed the leading role of the SO_n_ groups in lowering and stabilization of HOMO and LUMO frontier orbitals, making the mechanisms responsible for the anthracene oxidation more difficult [37,38].

This study also shows other photophysical/photochemical properties of the new groups of bridged, unsymmetrical, donor–acceptor SO_n_ (n = 0, 1, 2) chromophores of a dominant electron-rich character, based on a significantly substituted anthracene core. The study complements the literature investigations on symmetrically SO_n_ bridged, unsubstituted analogs. The (S) substrates were synthesized via the *thio*-Friedel–Crafts–Bradsher (*thio*-F–C–B) cyclization [33] as an extension of the *oxo*-variant of this reaction [39,40,41,42,43,44,45,46,47], while (SO) and (SO_2_) were obtained by a successive oxidizing of the sulfide bridge. The *thio*-F–C–B reaction is also a tool to introduce a significant substitution on the mono-RS-acene (anthracene) core, which also remains in the (SO) and (SO_2_) products after oxidation. This makes all three groups of compounds easily tunable optoelectronic materials that are of high photostability and high photooxidation stability. For instance, the colour of the emitted light can be tuned from blue-violet to green depending on the sulfur oxidation state, the number of electron-donating groups on anthracene, and the kind of solvent.

We believe that the findings herein will spur further interest and development in the unexplored area of photochemical behaviour of significantly substituted, SOn (n = 0, 1, 2)-bridged chromophores, both symmetrical and unsymmetrical, based on larger acenes than anthracene.

## 5. Patents

P.Bałczewski, E.Kowalska, J. Skalik, M.Koprowski, K.Owsianik, E.Różycka- Sokołowska, Aromatyczne sulfotlenki i sulfony, sposób ich wytwarzania i zastosowanie”, Patent Application, P.429272, 14.03.2019.

## Figures and Tables

**Table 1:** (S)-**1** obtained via the *thio*-Friedel–Crafts–Bradsher cyclization, (SO)-**2** and (SO_2_)-**3** synthesized via oxidation of (S)-**1**.

Compound.	Chemical Structure	Yield (%)
**1a**	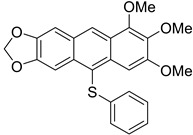	62
**1b**	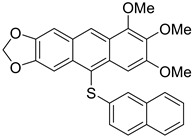	51
**1c**	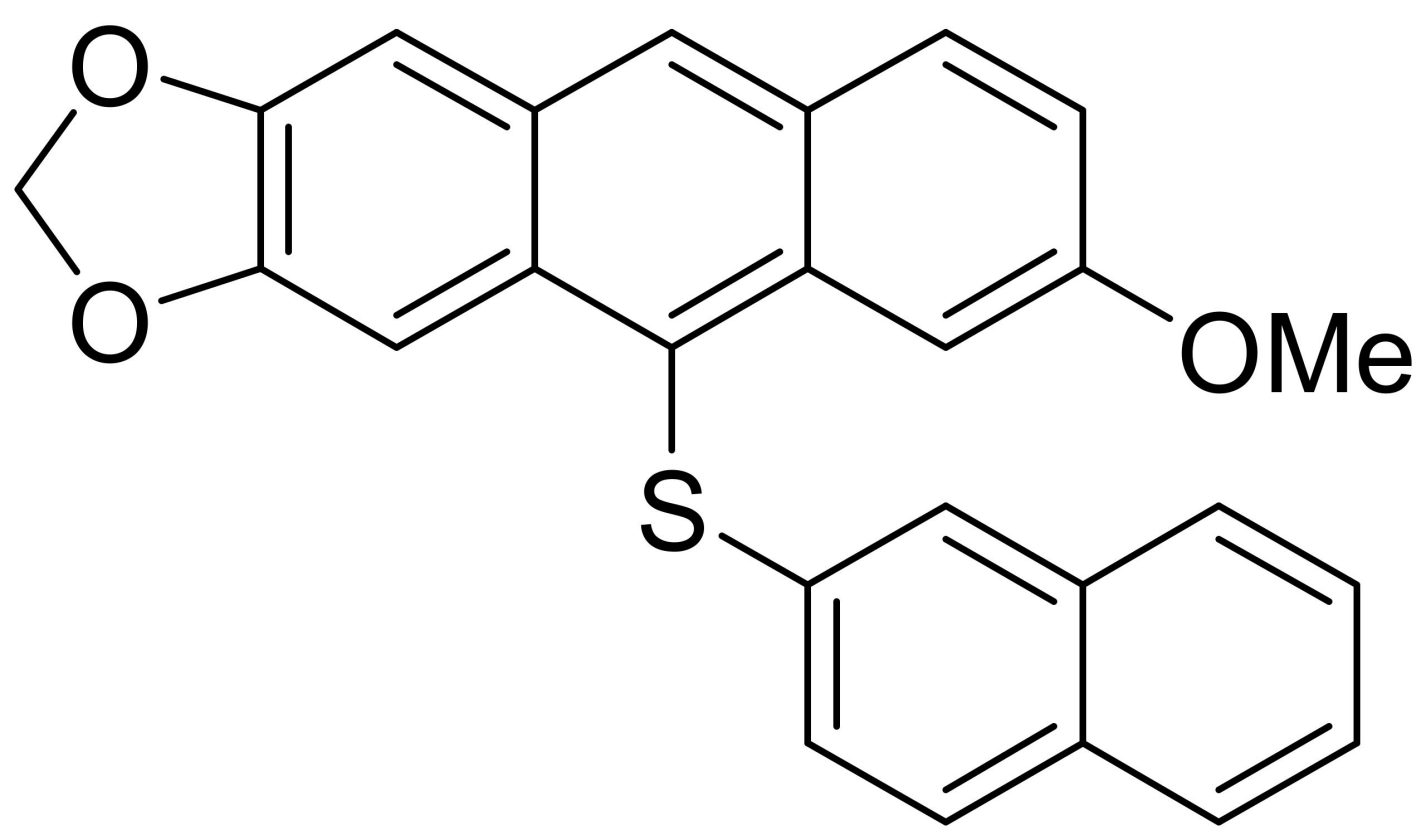	50
**1d**	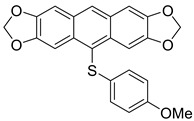	52
**2a**	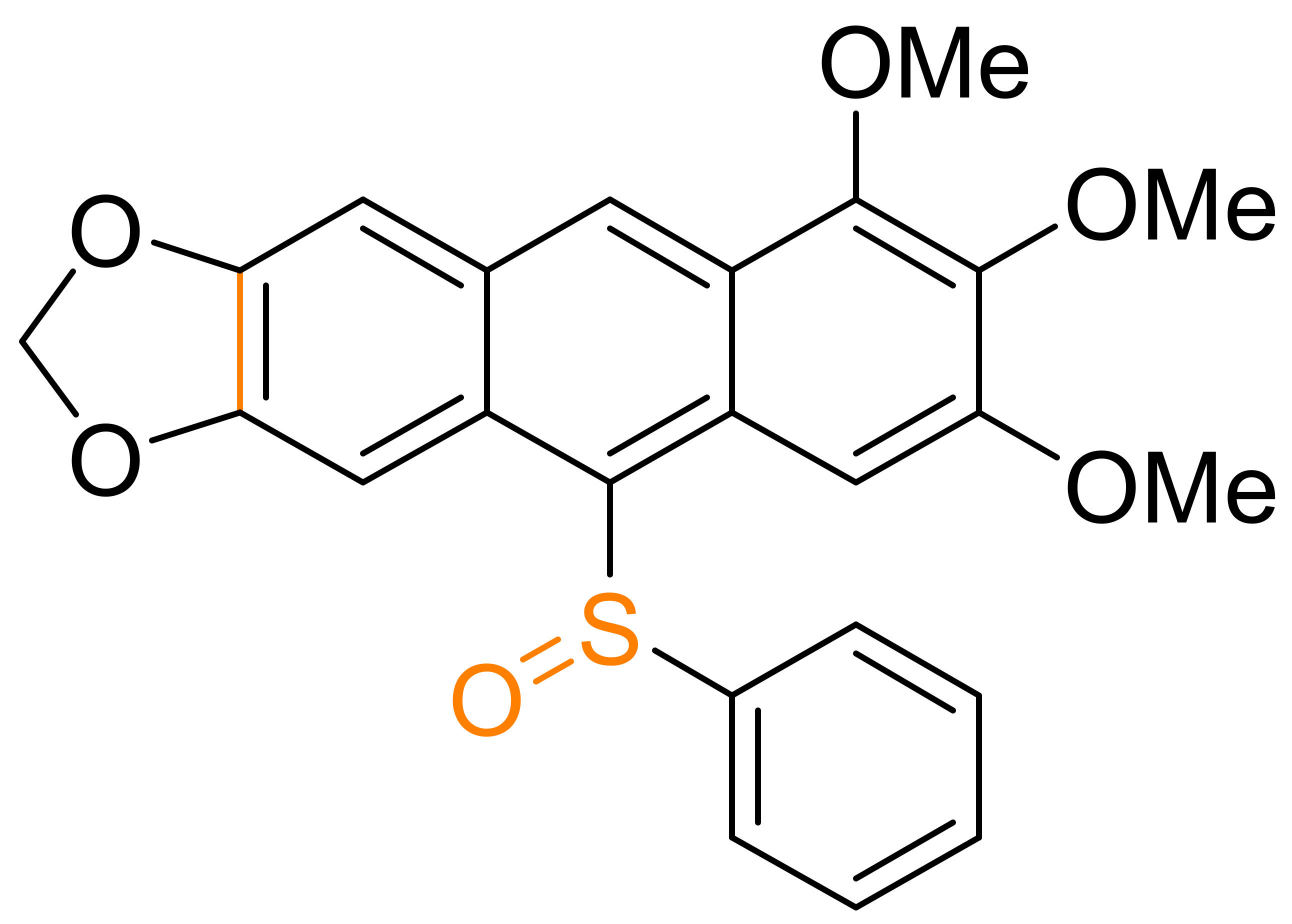	55
**2b**	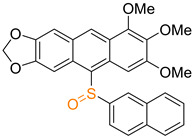	42
**2c**	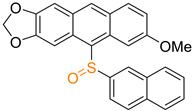	36
**2d**	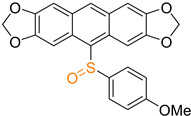	32
**3c**	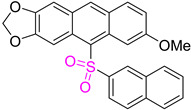	26
**3d**	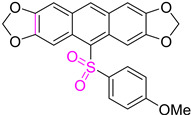	17

**Table 2:** Absorption and photoluminescence maxima (λ_max_), Stokes shifts, photoluminescence quantum yields (Φ) and lifetimes (τ) in solution (toluene and ethanol) for (S)-**1a–d**, (SO)-**2a–d** and (SO_2_)-**3c**, **3d** (underlined values are highest absorption maxima in a lower part of the spectra).

Compound	Absorption λ_max_ (nm)	Photoluminescence λ_max_ (nm)	Stokes Shift (cm^−1^)	τ ^(a)^ (ns)	χ^2 (b)^	Φ ^(c)^ (%)
**In toluene**						
**1a**	380, 401	445	3840	5.43	1.03	31.5
**2a**	380, 405	467	4900	8.29	1.20	40.6
**1b**	380, 403	447	3940	2.23	1.17	18.3
**2b**	380, 405	458	4480	8.70	1.17	55.6
**1c**	330, 348, 367, 383, 403	440	4520	1.94	1.10	15.0
**2c**	368, 386, 407	442	3280	4.06	1.01	21.6
**3c**	416, 395, 378, 328	460	4720	0.98	1.13	10.0
**1d**	364, 384	424	2460	0.23; 2.17 (0.95)	1.12	0.63
**2d**	367, 387	397	650	0.34; 4.75 (3.90)	1.09	1.91
**3d**	375, 392	433	3840	0.47; 3.98; 9.46 (7.50)	1.15	2.02
**In ethanol**						
**1a**	379, 399	442	3760	6.08	1.14	33.3
**2a**	379	473	5240	15.28	1.23	58.2
**1b**	379, 400	444	3860	2.24	1.01	16.6
**2b**	380	477	5350	14.23	1.20	54.2
**1c**	345, 365, 380, 401	437	4510	2.10	1.06	12.9
**2c**	368, 370, 386, 406	449	4900	1.91; 8.36 (5.15)	1.12	32.4
**3c**	376, 391, 413	466	5140	0.41; 3.80 (1.55)	1.04	1.9
**1d**	362, 382	405	1490	0.47; 2.81 (1.15)	1.24	0.1
**2d**	368, 387	398	710	0.28; 5.47 (4.20)	1.09	2.5
**3d**	376, 392	433	2420	0.16; 0.77; 5.64 (4.90)	1.45	0.53

^(a)^ In parentheses, the average lifetime calculated, taking into account the amplitudes for the each lifetime component. ^(b)^ χ^2^–CHISQ (chi square). ^(c)^ Quantum yields Φ were measured with an integrating sphere in the solvent indicated (1 × 10^−5^ M). Average lifetime should be used for comparison.

**Table 3:** Absorption and photoluminescence maxima (λ_max_) and Stokes shifts in solid state (thin film cast from toluene) for (S)-**1a–d**, S(O)-**2a–d** and (SO_2_)-**3c**, **3d**.

Compound	Absorption λ_max_ (nm)	Photoluminescence λ_max_ (nm)	Stokes Shift (cm^−1^)
**1a**	383	459	4320
**2a**	384	469	4720
**1b**	383	460	4370
**2b**	385	458	4140
**1c**	373	458	4980
**2c**	373	439	4030
**3c**	383	473	4970
**1d**	394	441	2700
**2d**	393	458	3610
**3d**	397	448	2870

**Table 4:** The Commission Internationale de L’Eclairage (CIE) 1931 chromaticity coordinates for (S)-**1a–d**, (SO)-**2a–d** and (SO_2_)-**3c**, **3d** in ethanol and toluene solutions.

The CIE 1931 Color Spaces-Luminosity Function		
Compound	Toluene (x, y)	Ethanol (x, y)
**1a**	x = 0.1534, y = 0.0891	x = 0.15148, y = 0.09148
**2a**	^(a)^	x = 0.17369, y = 0.29827
**1b**	x = 0.15012, y = 0.0855	x = 0.16057, y = 0.10734
**2b**	x = 0.14996, y = 0.16765	x = 0.17792, y = 0.3006
**1c**	x = 0.1627, y = 0.10657	x = 0.16889, y = 0.1198
**2c**	x = 0.1447, y = 0.09946	x = 0.14522, y = 0.13796
**3c**	^(a)^	x = 0.16769, y = 0.29084
**1d**	x = 0.1526, y = 0.03397	x = 0.14725, y = 0.05423
**2d**	x = 0.14357, y = 0.06482	x = 0.14886, y = 0.04898
**3d**	x = 0.14217, y = 0.06996	x = 0.13137, y = 0.11087

^(a)^ not measured due to poor solubility in toluene.

**Table 5:** The time of 50% loss of absorbance decay measured for the lowest energy absorption bands of (S)-**1a–d** and (SO)-**2a–d** in ethanol solutions under UV light (254/365 nm) exposure.

Compound	The Time of 50% Loss of Absorbance Measured for the Lowest Energy Absorption Band (min)		
	UV-Vis 254 nm/O_2_	UV-Vis 254 nm/Ar	UV-Vis 365 nm/O_2_
**1a**	166	175	n/o ^(a)^
**2a**	80	88	n/o ^(a)^
**1b**	249	519	n/o ^(a)^
**2b**	249	251	n/o ^(a)^
**1c**	210	276	n/o ^(a)^
**2c**	247	245	254
**1d**	115	n/o	812
**2d**	111	93	124

^(a)^ absorbance decay not observed.

## Data Availability

The datasets generated and analyzed during the current study are available from the corresponding author on reasonable request.

## Supplementary Materials

The following are available online at https://www.mdpi.com/article/10.3390/ma14133506/s1, Pages S1–S4: Synthetic procedures and spectroscopic data; Figure S1: ^1^H NMR and ^13^C NMR spectra of the obtained compounds; Figure S2: Chemical structures of sulfides **1a–d**, sulfoxides **2a–d** and sulfones **3c**,**d** used in measurements; Figure S3: Absorption spectra for sulfides **1a–d**, sulfoxides **2a–d** and sulfones **3c,d** in ethanol solutions; Figure S4: Time-correlated single-photon counting (TCSPC) measurements of photoluminescence lifetimes in ethanol solutions; Figure S5: UV/Vis absorption and photoluminescence spectra for sulfides **1a–d**, sulfoxides **2a–d** and sulfones **3c,d** in toluene solutions; Figure S6: Time-correlated single-photon counting (TCSPC) measurements of photoluminescence lifetimes in toluene solutions; Figure S7: Absorption spectra recorded for sulfides **1a–d**, sulfoxides **2a–d** and sulfones **3c**,**d** in solid state (thin-films); Figure S8: Photostability and photooxidation stability of compounds: **1a**–**d**, **2a**-**d** and **3c**,**d** in ethanol solutions; Figure S9: HRMS-(+)-APCI spectra of **1a**; Figure S10: HRMS-(+)-APCI spectra of **1b**; Figure S11: HRMS-(+)-APCI spectra of **1d**; Figure S12: HRMS-(+)-APCI spectra of **2a**; Figure S13: HRMS-(+)-APCI spectra of **2d**; Figure S14: HRMS-(+)-APCI spectra of **3c**; Figure S15: HRMS-(+)-APCI spectra of **3d**; Tables S1–S12: Atom coordinates (Å), total energy (Hartree) and the number of imaginary vibrational frequencies for the geometries of **1a–d**, **2a–d**, and **3a–d** optimized according to the B3LYP/6-311++(d,p) level in the gas phase using Gaussian 09.

## References

[1] Xu Y., Xu P., Hu D., Ma Y. (2021). Recent progress in hot exciton materials for organic light-emitting diodes. Chem. Soc. Rev..

[2] Sasabe H., Seino Y., Kimura M., Kido J. (2012). A m-Terphenyl-Modifed Sulfone Derivative as a Host Material for High-Efficiency Blue and Green Phosphorescent OLEDs. Chem. Mater..

[3] Zhang Q., Li J., Shizu K., Huang S., Hirata S., Miyazaki H., Adachi C. (2012). Design of Efficient Thermally Activated Delayed Fluorescence Materials for Pure Blue Organic Light Emitting Diodes. J. Am. Chem. Soc..

[4] Yang S.-Y., Tian Q.-S., Yu Y.-J., Zou S.-N., Li H.-C., Khan A., Wu Q.-H., Jiang Z.-Q., Liao L.-S. (2020). Sky-Blue Thermally Activated Delayed Fluorescence with Intramolecular Spatial Charge Transfer Based on a Dibenzothiophene Sulfone Emitter. J. Org. Chem..

[5] Jeon S.O., Earmme T., Jenekhe S.A. (2014). New sulfone-based electron-transport materials with high triplet energy for highly efficient blue phosphorescent organic light-emitting diodes. J. Mater. Chem. C.

[6] Di Z., Tingting Y., Zhengqin W., Huixia X., Hua W., Junsheng Y., Bingshe X. (2020). Photoelectric properties of host materials based on diphenyl sulfone as acceptor and the performances in green phosphorescent OLEDs. Opt. Mater..

[7] Jürgensen N., Kretzschmar A., Höfle S., Freudenberg J., Bunz U.H.F., Hernandez-Sosa G. (2017). Sulfone-Based Deep Blue Thermally Activated Delayed Fluorescence Emitters: Solution-Processed Organic Light-Emitting Diodes with High Efficiency and Brightness. Chem. Mater..

[8] Tonge C.M., Zeng J., Zhao Z., Tang B.Z., Hudson Z.M. (2020). Bis(hexamethylazatriangulene)sulfone: A high-stability deep blue-violet fluorophore with 100% quantum yield and CIE *y* <0.07. J. Mater. Chem. C.

[9] Liu X., Xu R., Duan C., Huang F., Cao Y. (2016). Non-conjugated water/alcohol soluble polymers with different oxidation states of sulfide as cathode interlayers for high-performance polymer solar cells. J. Mater. Chem. C.

[10] Li S., Ye L., Wang Q., Zhang S., Zhao W., Hou J. (2016). Improving the open-circuit voltage of alkylthio-substituted photovoltaic polymers via post-oxidation. Org. Electron..

[11] Hirose A., Tanaka K., Yoshii R., Chujo Y. (2015). Film-type chemosensors based on boron diiminate polymers having oxidation-induced emission properties. Polym. Chem..

[12] Lamberth C. (2004). Sulfur chemistry in crop protection. J. Sulfur Chemistry.

[13] Kathayat R.S., Yang L., Sattasathuchana T., Zoppi L., Baldridge K.K., Linden A., Finney N.S. (2016). On the Origins of Nonradiative Excited State Relaxation in Aryl Sulfoxides Relevant to Fluorescent Chemosensing. J. Am. Chem. Soc..

[14] Wang M., Ma X., Yu J., Jia X., Han D., Zhou T., Yang J., Nie J., Wang T. (2015). Aromatic amine–sulfone/sulfoxide conjugated D–π-A–π-D-type dyes in photopolymerization under 405 nm and 455 nm laser beams. Polym. Chem..

[15] Christensen P.R., Wolf M.O. (2016). Photopatterned Multidimensional Fluorescent Images. Adv. Funct. Mater..

[16] Mísek J., Jentzsch A.V., Sakurai S., Emery D., Mareda J., Matile S. (2010). A Chiral and Colorful Redox Switch: Enhanced π-Acidity in Action. Angew. Chem. Int. Ed..

[17] Monçalves M., da Silveira Rampon D., Schneider P.H., Rodembusch F.S., da Cruz Silveira C. (2014). Divinyl sulfides/sulfones-based D–π–A–π–D dyes as efficient non-aromatic bridges for π-conjugated compounds. Dyes. Pigm..

[18] Benz S., Mareda J., Besnard C., Sakai N., Matile S. (2017). Catalysis with chalcogen bonds: Neutral benzodiselenazole scaffolds with high-precision selenium donors of variable strength. Chem. Sci..

[19] Caron E., Wolf M.O. (2017). Soluble Oligo- and Polythienyl Sulfides and Sulfones: Synthesis and Photophysics. Macromolecules.

[20] Monçalves M., Zanotto G.M., Toldo J.M., Rampon D.S., Schneider P.H., Gonçalves P.F.B., Rodembusch F.S., Silveira C.C. (2017). Dipolar vinyl sulfur fluorescent dyes. Synthesis and photophysics of sulfide, sulfoxide and sulfone based D–π–A compounds. RSC Adv..

[21] Verolet Q., Rosspeintner A., Soleimanpour S., Sakai N., Vauthey E., Matile S. (2015). Turn-On Sulfide π Donors: An Ultrafast Push for Twisted Mechanophores. J. Am. Chem. Soc..

[22] Piazza G.A., Rahm A.L.K., Krutzsch M., Sperl G., Paranka N.S., Gross P.H., Brendel K., Burt R.W., Alberts D.S., Pamukcu R. (1995). Antineoplastic Drugs Sulindac Sulfide and Sulfone Inhibit Cell Growth by Inducing Apoptosis. Cancer Res..

[23] Liu L., Stelmach J.E., Natarajan S.R., Chen M.H., Singh S.B., Schwatrz C.D., Fitzgerald C.E., O’Keefe S.J., Zaller D.M., Schmatz D.B. (2003). SAR of 3,4-Dihydropyrido [3,2-*d*] pyrimidone p38 inhibitors. Bioorg. Med. Chem. Lett..

[24] Feng M., Tang B., Liang S.H., Jiang X. (2016). Sulfur Containing Scaffolds in Drugs: Synthesis and Application in Medicinal Chemistry. Curr. Top. Med. Chem..

[25] Godumala M., Choi S., Cho M.J., Choi D.H. (2016). Thermally activated delayed fluorescence blue dopants and hosts: From the design strategy to organic light-emitting diode applications. J. Mater. Chem. C.

[26] Christensen P.R., Nagle J.K., Bhatti A., Wolf M.O. (2013). Enhanced Photoluminescence of Sulfur-Bridged Organic Chromophores. J. Am. Chem. Soc..

[27] Cruz C.D., Christensen P.R., Chronister E.L., Casanova D., Wolf M.O., Bardeen C.J. (2015). Sulfur-Bridged Terthiophene Dimers: How Sulfur Oxidation State Controls Interchromophore Electronic Coupling. J. Am. Chem. Soc..

[28] Climent C., Barbatti M., Wolf M.O., Bardeend C.J., Casanova D. (2017). The photophysics of naphthalene dimers controlled by sulfur bridge oxidation. Chem. Sci..

[29] Malashikhin S., Finney N.S. (2008). Fluorescent Signaling Based on Sulfoxide Profluorophores: Application to the Visual Detection of the Explosive TATP. J. Am. Chem. Soc..

[30] Malashikhin S. (2010). Efficient Discovery of Fluorescent Chemosensors Based on the Biarylpyridine Scaffold: Visual Detection of the Explosive TATP the Origin of Sulfoxides “Non Emission”. Ph.D. Thesis.

[31] Christensen P.R., Patrick B.O., Caron E., Wolf M.O. (2013). Oxidation-State-Dependent Photochemistry of Sulfur-Bridged Anthracenes. Angew. Chem. Int. Ed..

[32] Zhao C., Wang X., Cao J., Feng P., Zhang J., Zhang Y., Yang Y., Yang Z. (2013). BODIPY-based sulfoxide: Synthesis, photophysical characterization and response to benzenethiols. Dye. Pigm..

[33] Bałczewski P., Kowalska E., Różycka-Sokołowska E., Skalik J., Owsianik K., Koprowski M., Marciniak B., Guziejewski D., Ciesielski W. (2019). Mono-Aryl/Alkylthio-Substituted (Hetero)acenes of Exceptional Thermal and Photochemical Stability by the Thio-Friedel–Crafts/Bradsher Cyclization Reaction. Chem. Eur. J..

[34] Bałczewski P., Kowalska E., Skalik J. (2017). 10-Thiosubstituted Pentahydroxyanthracene Derivatives, a Method of Their Preparation and Intermediate Compounds. EPO appl., No. EP-14460003.8, 06.02.2014. EPO patent.

[35] Gurria G.M., Posner G.H. (1973). Photochemical deoxygenation of aryl sulfoxides. J. Org. Chem..

[36] (2015). ACD/Percepta, Version 14.0.0.

[37] Zhang J., Sarrafpour S., Haas T.E., Müller P., Thomas S.W. (2012). Structure, photophysics and photooxidation of crowded diethynyltetracenes. J. Mater. Chem..

[38] Maliakal A., Raghavachari K., Katz H., Chandross E., Siegrist T. (2004). Photochemical Stability of Pentacene and a Substituted Pentacene in Solution and in Thin Films. Chem. Mater..

[39] Bałczewski P., Koprowski M., Bodzioch A., Marciniak B., Różycka-Sokołowska E. (2006). Unusual Transformation of the Diarylmethanol Derivative into an Unknown 1,2,3,6,7,10-Hexahydroxylated Anthracene System. J. Org. Chem..

[40] Bałczewski P., Bodzioch A., Różycka-Sokołowska E., Marciniak B., Uznański P. (2010). First Approach to Nitrogen-Containing Fused Aromatic Hydrocarbons as Targets for Organoelectronics Utilizing a New Transformation of O-Protected Diaryl Methanols. Chem. Eur. J..

[41] Bodzioch A., Marciniak B., Różycka-Sokołowska E., Jeszka J.K., Uznański P., Kania S., Kuliński J., Bałczewski P. (2012). Synthesis and Optoelectronic Properties of Hexahydroxylated 10-RO-Substituted Anthracenes via a New Modification of the Friedel–Crafts Reaction Using O-Protected ortho-Acetal Diarylmethanols. Chem. Eur. J..

[42] (2014). 10-Thiosubstituted Pentahydroxyanthracene Derivatives, a Method of Their Preparation and Intermediate Compounds; EP 288539.

[43] (2019). Tio-Sfunkcjonalizowane aceny i ich Zastosowanie.

[44] Bałczewski P., Skalik J., Uznański P., Guziejewski D., Ciesielski W. (2015). Use of isomeric, aromatic dialdehydes in the synthesis of photoactive, positional isomers of higher analogs of o-bromo (hetero) acenaldehydes. RSC Adv..

[45] Kowalska E., Bałczewski P. (2017). Ultrasound assisted Bradsher reaction in aqueous and non-aqueous media: First use of ultrasounds in electrophilic aromatic cyclisation leading to polyacenes. Ultrason. Sonochem..

[46] Bałczewski P., Bodzioch A., Koprowski M. (2014). New Condensed Polyaromatic and Polyheteroaromatic Hydrocarbons, Method of Their Manufacturing and the Intermediate Compounds. UPRP.

[47] thioBodzioch A., Kowalska E., Skalik J., Bałczewski P. (2017). Synthesis of Polycyclic (Hetero)Aromatic Hydrocarbons via the Friedel–Crafts/Bradsher Cyclization. Chem. Heterocycl. Compd..

