# Supplementary material for: Organosulfur Materials with High Photo- and Photo-Oxidation Stability: 10-Anthryl Sulfoxides and Sulfones and Their Photophysical Properties Dependent on the Sulfur Oxidation State
Source: Materials (Basel). 2021 Jun 23;14(13):3506. doi: 10.3390/ma14133506 (PMC8269563; doi:10.3390/ma14133506)
Supplement: Supplementary file 1 [file materials-14-03506-s001.zip › materials-1225246-supplementary.pdf]

# Supplementary Materials (SM)

**Organosulfur Materials with High Photo- and Photo-oxidation Stability: 10-Anthryl Sulfoxides and Sulfones and Their Photophysical Properties Dependent on the Sulfur Oxidation State**

**Piotr Bałczewski [1,2,*], Emilia Kowalska [1], Ewa Różycka-Sokołowska [2], Paweł Uznański [3], Joanna Wilk [1], Marek Koprowski [1], Krzysztof Owsianik [1] and Bernard Marciniak [2]**

1 Centre of Molecular and Macromolecular Studies, Functional Materials Synthesis Group, Division of Organic Chemistry, Polish Academy of Sciences, Sienkiewicza 112, 90-363 Łódź, Poland; kowala88@vp.pl (E.K.); jskalik@cbmm.lodz.pl (J.W.); mkopr@cbmm.lodz.pl (M.K.); owsianik@cbmm.lodz.pl (K.O.)
2 Structural & Material Chemistry Group, Faculty of Science and Technology, Institute of Chemistry, Jan Dlugosz University in Częstochowa, Armii Krajowej 13/15, 42-200 Częstochowa, Poland; e.sokolowska@ujd.edu.pl (E.R.-S.); b.marciniak@ujd.edu.pl (B.M.)
3 Centre of Molecular and Macromolecular Studies, Division of Polymers, Polish Academy of Sciences, Sienkiewicza 112, 90-363 Łódź, Poland; puznansk@cbmm.lodz.pl
* Correspondence: pbalczew@cbmm.lodz.pl

# Table of contents

Synthetic procedures and spectroscopic data S1-S4

$^{1}$H NMR and $^{13}$C NMR spectra S5-S26

Optical properties S27-S32

Photostability and photo-oxidation stability S33-S53

HRMS APCI spectra S54-S80

Cartesian coordinates and total energies for the geometries of **1a**, **1b**,**1c**, **1d**, **2a**, **2b**, **2c**, **2d**, **3a**, **3b**, **3c** and **3d** optimized using Gaussian 09 S81-S94

References S94

# Synthetic procedures and spectroscopic data

**General:** *m*-Chloroperbenzoic acid (m-CPBA, ≥77%) was purchased from Aldrich. Dry and oxygen free dichloromethane (DCM, JT Baker Chemicals) was taken from MB SPS-800 (MBRAUN Solvent Purification System). The $^{1}$H NMR and $^{13}$C NMR spectra were measured with a Bruker AV 200 or AV 500 spectrometers in $CDCl_3$ with chemical shifts (δ) given in ppm relative to TMS as an internal standard. High-resolution mass spectrometry (HRMS) measurements were performed using Synapt G2-Si mass spectrometer (Waters) equipped with an APCI source and quadrupole-Time-of-Flight mass analyzer. The mass spectrometer was operated in the positive ion detection mode with discharge current set at 4.0 μA. The heated capillary temperature was 350 °C. The results of the measurements were processed using the MassLynx 4.1 software (Waters) incorporated with the instrument. Accurate mass measurements were performed by a peak matching technique using perfluorokerosene as an internal standard at a resolving power of 10.000 (10% valley definition). The HR(MS_MS)-(+)-APCI spectra were recorded at 15 eV, 25 eV and 35 eV. Melting points were measured using Boetius apparatus. Thin layer chromatography (TLC) was performed on precoated Merck 60 ($F_{254}$ 60) silica gel plates with fluorescent indicator, with detection by means of UV light at 254 and 360 nm. Column chromatography was done on Merck silica gel

(Kieselgel 60, 230-400 mesh). IR spectra were carried out with a FT-IR spectrometer ATI Mattson model Infinity AR60 in KBr pellets and reported in $cm^{-1}$. The UV-Vis absorption spectra were recorded in 1 cm cuvettes on a Shimadzu UV-2700 spectrophotometer using two types of the light source: deuterium lamp D2 64604 and halogen lamp Wl L6380 (220 - 600 nm). Room temperature, steady-state emission spectra were obtained with the Horiba Jobin Yvon, Fluorolog-3 spectrofluorimeter using xenon lamp as a light source. The fluorescence quantum yields Φ of the obtained compounds were determined in EtOH and toluene on excitation at their absorption maximum using an integrating sphere (Horiba, Jobin Yvon, Quanta-φ F-3029 Integrating sphere). Fluorescence lifetime values were measured by the Time Correlated Single Photon Counting (TCSPC) analysis. The fluorescence decay curves were obtained by exciting the molecules at their emission maximum using NanoLED laser at 374 nm ± 10 nm (< 200 ps) as a light source. The photodegradation measurements were investigated by monitoring the absorbance decay of $10^{-5}$ ethanolic solutions in a quartz cuvette stored in the dark at room temperature, under ambient atmosphere ($O_2$) and inert atmosphere (Ar), and then exposed to UVP-Hg-Pen-ray lamp (254 nm, 16.33 mW/$cm^2$ at distance 1 cm) and fluorescent lamp VL-6.LC (6W), 365 nm, 27.4 mW/$cm^2$ at distance 1 cm).

## Synthesis of sulfides 1

To a solution of appropriate diarylmethyl thioethers (1.1 mmol) in dry EtOH (10 mL), $FeCl_3$ (1.21 mmol, 1.1 equiv.) and KI (1.21 mmol, 1.1 equiv.) were added. The mixture was refluxed until disappearance of the starting material (monitoring by TLC). After completion of the reaction, the solvent was removed. To the crude product, ethyl acetate (10 mL) was added and the resulting mixture was poured onto saturated solution of $Na_2S_2O_{3aq}$ (10 mL). The organic layer was dried over anhydrous $MgSO_4$. The solvent was evaporated to give products (**1a-d**), which were purified using a gradient column chromatography (petroleum: acetone, 10:1 v/v).[1]

## Synthesis of sulfoxides 2

*meta*-Chloroperbenzoic acid (33 mg, 0.196 mmol, 1 equiv.) was added to a solution of **1** (0.196 mmol, 1 equiv.) in $CH_2Cl_2$ (20 mL) at 0 °C and the resulting solution was heated to reflux under argon atmosphere overnight in the dark. Next, the reaction mixture was stirred overnight in the dark, then poured onto saturated aqueous solution of sodium bicarbonate/ice, and finally extracted with $CH_2Cl_2$ (2x20 mL). The combined organic layers were dried over anhydrous $MgSO_4$, filtered, and concentrated to afford orange solids. Purification was carried out with column chromatography (hexanes/$Et_2O$, 1:1) to afford yellow solids of **2**.

## Synthesis of sulfones 3

*m*-Chloroperbenzoic acid (66 mg, 0.392 mmol, 2 equiv.) was added to a solution of **1** (0.196 mmol, 1 equiv.) in $CH_2Cl_2$ (20 mL) at 0 °C and the resulting mixture was heated to reflux overnight in the dark under argon atmosphere. After this time, the reaction mixture was poured onto saturated aqueous solution of sodium bicarbonate/ice, and then extracted with $CH_2Cl_2$ (2x20 mL). The organic layers were combined, dried over anhydrous $MgSO_4$, filtered, and concentrated to afford orange solids. Purification by column chromatography (hexanes/$Et_2O$, 1:1) afforded yellow solids of **3**.

***7,8,9-Trimethoxy-5-(phenylthio)anthra[2,3-d][1,3]dioxole (1a)***

Yield: 62% as an yellow solid, m.p. 140°C; $^1$H NMR (200 MHz, $CDCl_3$): δ = 3.89 (s, 3H, $OCH_3$), 3.99 (s, 3H, $OCH_3$), 4.13 (s, 3H, $OCH_3$), 6.01 (s, 2H, $OCH_2O$), 6.89-7.16 (5H, m, 5x$H_{Ar}$, $C_6H_5$), 7.23 (s, 1H, $H_{Ar}$), 7.76 (s, 1H, $H_{Ar}$), 7.98 (s, 1H, $H_{Ar}$), 8.53 (s, 1H, $H_{Ar}$); $^{13}$C NMR (50 MHz, $CDCl_3$): δ = 54.54, 59.90, 60.23, 98.86, 99.92, 100.52, 102.21, 119.97, 121.09, 122.05, 123.65, 125.00, 126.99, 127.59, 130.76, 132.38, 136.93, 145.69, 145.93, 148.31, 152.49; MS (EI, 70 eV): *m/z* (%): 420 (100, $M^+$), 374 (30, $M^+$, -$OCH_2O$); MS (CI, isobutane): *m/z* (%) 420 (100, $M^+$) 374 (10, $M^+$, -$OCH_2O$); HRMS (EI, 70 eV): *m/z* Calcd. for $C_{24}H_{20}O_5S$: 420.1032; Found: 420.1030; Elemental analysis: Found: C, 68.32; H, 4.95; S, 7.59. Calc. for $C_{24}H_{20}O_5S$: C, 68.55; H, 4.79; S, 7.63 %.

***7,8,9-Trimethoxy-5-(naphth-2-ylthio)anthra[2,3-d][1,3]dioxole (1b)***

Yield: 51% as an yellow solid; m.p. 92°C; $^1$H NMR (500 MHz, $C_6D_6$): δ = 3.37 (s, 3H, $OCH_3$), 3.80 (s, 3H, $OCH_3$), 3.95 (s, 3H, $OCH_3$), 5.13 (s, 2H, $OCH_2O$), 6.98-7.06 (m, 2H, 2x$H_{Ar}$, 6,7-naphth), 7.09-7.11 (m, 2H, 2x$H_{Ar}$), 7.22 ($d_{AB}$d, $^3J_{HHAB}$ = 8.5, $^4J_{HH}$ = 2.0 Hz, 1H, $H_{Ar}$), 7.29 ($d_{AB}$, $^3J_{HHAB}$ = 8.5 Hz, 1H, 2x$H_{Ar}$), 7.37 (d, $^3J_{HH}$ = 8.5 Hz, 1H, $H_{Ar}$), 7.51 (d, $^4J_{HH}$ = 1.0 Hz, 1H, $H_{Ar}$), 8.12 (s, 1H, $H_{Ar}$), 8.47 (s, 1H, $H_{Ar}$), 8.83 (s, 1H, $H_{Ar}$); $^{13}$C NMR (50 MHz, $C_6D_6$): δ = 54.97, 60.62, 60.98, 100.30, 100.90, 101.91, 103.73, 121.31, 123.19, 124.00, 124.93,

125.11, 126.40, 127.02, 127.68, 128.85, 131.64, 132.76, 134.21, 136.25, 147.58, 150.12, 154.76; MS (EI, 70 eV) *m/z* (%): 470 (100, $M^+$), 423 (32, $M^+$, -$OCH_2O$); MS (CI, isobutane): *m/z* (%) 470 (80, $M^+$); HRMS (EI, 70 eV): *m/z* Calcd for $C_{28}H_{22}O_5S$: 470.1188; Found: 470.1184; Elemental analysis: Found: C, 71.54; H, 4.97; S, 6.75. Calc. for $C_{28}H_{22}O_5S$: C, 71.47; H, 4.71; S, 6.81 %.

***7-Methoxy-5-(naphth-2-ylthio)anthra[2,3-d][1,3]dioxole (1c)***

Yield: 50% as an yellow solid; m.p. >200°C; $^1$H NMR (500 MHz, $CDCl_3$): δ = 3.84 (s, 3H, $OCH_3$), 6.02 (s, 2H, $OCH_2O$), 7.14 (dd, $^3J$ = 9.0, $^4J_{HH}$ = 2.5 Hz, 2H, Hz, 2x$H_{Ar}$), 7.23 (s, 1H, $H_{Ar}$), 7.32 (s, 1H, $H_{Ar}$), 7.33-7.35 (m, 2H, 2x$H_{Ar}$, 6,7-naphth), 7.47-7.49 (m, 1H, $H_{Ar}$, 5-naphth), 7.60 (d, $^3J$ = 8.5 Hz, 1H, $H_{Ar}$), 7.68-7.70 (m, 1H, $H_{Ar}$, 8-naphth), 7.86 (d, $^3J$ = 9.0 Hz, 1H, $H_{Ar}$), 7.99 (d, $^4J_{HH}$ = 2.0Hz, 1H, $H_{Ar}$), 8.07 (s, 1H, $H_{Ar}$), 8.30 (s, 1H, $H_{Ar}$); $^{13}$C NMR (125 MHz, $CDCl_3$): δ = 55.43, 101.38, 101.96, 103.02, 103.33, 119.85, 121.11, 129.98, 125.13, 125.25, 126.49, 127.01, 127.64, 127.78, 128.35, 128.59, 128.82, 130.23, 131.43, 133.92, 134.43, 135.72, 135.99, 147.35, 149.98, 158.55; MS (EI, 70 eV) *m/z* (%): 410 (100, $M^+$); HRMS (EI, 70 eV): *m/z* Calcd. for $C_{26}H_{18}O_3S$: 410.0977; Found: 410.0984; Elemental analysis: Found: C, 75.81; H, 4.72; S 8.05. Calc. for $C_{26}H_{18}O_3S$: C, 76.08; H, 4.42; S, 7.81 %.

***5-(4-Methoxyphenylthio)anthra[2,3-d:6,7-d']bis[1,3]dioxole (1d)***

Yield: 52% as an yellow solid; m.p. >200°C; $^1$H NMR (200 MHz, $CDCl_3$): δ = 3.69 (s, 3H, $OCH_3$), 6.03 (s, 4H, 2x$OCH_2O$), 6.68 ($d_{AB}$, $^3J_{HHAB}$ = 9.0 Hz, 2H, 2x *o*-$C_6H_4$-OMe), 6.91 ($d_{AB}$, $^3J_{HHAB}$ = 9.0 Hz, 2H, 2x *m*-$C_6H_4$-OMe), 7.15 (s, 2H, 2x$H_{Ar}$), 8.07 (s, 2H, 2x$H_{Ar}$), 8.08 (s, 1H, $H_{Ar}$); $^{13}$C NMR (50 MHz, $CDCl_3$): δ = 55.27, 101.16, 102.11, 102.68, 114.66, 123.40, 127.03, 128.04, 128.83, 132.51, 147.34, 149.01, 157.61; MS (EI, 70 eV) *m/z* (%): 404 (100, $M^+$), 389 (23, $M^+$, -Me), 372 (12, $M^+$, -MeOH); HRMS (EI, 70 eV): *m/z* Calcd for $C_{23}H_{16}O_5S$: 404.0719; Found: 404.0720; Elemental analysis: Found: C, 68.19; H, 3.86; S, 8.02. Calc. for $C_{23}H_{16}O_5S$: C, 68.30; H, 3.99; S, 7.93 %.

***7,8,9-Trimethoxy-5-(phenylsulfinyl)anthra[2,3-d][1,3]dioxole (2a)***

Yield: 55%, yellow solid, m.p. 194-196°C; $^1$H NMR ($CDCl_3$, 200 MHz): δ = 3.86 (s, 3H, $OCH_3$), 3.94 (s, 3H, $OCH_3$), 4.08 (s, 3H, $OCH_3$), 6.05 (s, 2H, $OCH_2O$), 7.22 (s, 1H, $H_{Ar}$), 7.31-7.40 (m, 3H, 3x$H_{Ar}$, *m*-$C_6H_5$, *p*-$C_6H_5$), 7.46-7.55 (m, 2H, 2x$H_{Ar}$), 7.81 (s, 1H, $H_{Ar}$), 8.08 (s, 1H, $H_{Ar}$), 8.56 (s, 1H, $H_{Ar}$) ppm; $^{13}$C NMR ($CDCl_3$, 125 MHz): δ = 56.00, 61.23, 61.60, 97.39, 98.56, 101.52, 103.90 (s, 2x$CH_{Ar}$), 123.49, 124.43 (s, 2x$CH_{Ar}$), 125.32 (s, 2x$C_{Ar}$), 127.91, 128.15, 128.52, 128.95 (s, 2x$CH_{Ar}$), 129.69, 139.93, 144.79, 147.17 (s, 2x$C_{Ar}$), 150.03, 154.28 ppm; MS (EI, 70 eV) *m/z* (%): 436 [$M^+$, 100], 420 [$M^+$,-O, 52], 388 [$M^+$, -$HOCH_2OH$, 30], 359 [$M^+$, -$C_6H_5$, 51]; HRMS (EI, 70 eV): *m/z* $[M]^+$ Calcd for $C_{24}H_{20}O_6S$: 436.0981; Found: 436.0981; IR (KBr) $cm^{-1}$: 3443, 3062, 2935, 2836, 1625, 1542, 1479, 1464, 1415, 1338, 1250, 1223, 1109, 1038, 1004, 955, 896, 830, 749, 694.

***7,8,9-Trimethoxy-5-(naphth-2-yl-sulfinyl)anthra[2,3-d][1,3]dioxole (2b)***

Yield: 42%, yellow solid, m.p. 186-188°C; $^1$H NMR ($CDCl_3$, 200 MHz): δ = 3.84 (s, 3H, $OCH_3$), 3.92 (s, 3H, $OCH_3$), 4.07 (s, 3H, $OCH_3$), 6.00-6.08 (m, 2H, $OCH_2O$), 7.17 (dd, $^3J_{HH}$ = 10.0, $^4J_{HH}$ = 2.0 Hz, 1H, $H_{Ar}$), 7.24 (d, $^4J_{HH}$ = 2.0 Hz, 1H, $H_{Ar}$), 7.44-7.53 (m, 2H, 2x$H_{Ar}$, 6,7-naphth), 7.54 (s, 1H, $H_{Ar}$), 7.68-7.81 (m, 1H, $H_{Ar}$), 7.88-7.93 (m, 1H, $H_{Ar}$), 7.91 (s, 1H, $H_{Ar}$), 8.17 (s, 1H, $H_{Ar}$), 8.34 (s, 1H, $H_{Ar}$), 8.57 (s, 1H, $H_{Ar}$) ppm; $^{13}$C NMR ($CDCl_3$, 125 MHz): δ = 55.95, 61.21, 61.61, 97.39, 98.58, 101.53, 103.94 (2x$CH_{Ar}$), 120.74, 123.52, 124.51, 125.42, 127.26, 127.41 (2x$CH_{Ar}$), 127.98, 128.13 (2x$CH_{Ar}$), 128.32, 129.09, 132.84, 133.62, 139.93, 141.86, 147.20 (2x$C_{Ar}$), 150.76,09, 154.22 ppm; MS (EI, 70 eV) *m/z* (%): 486 [$M^+$, 31], 470 [$M^+$, - O, 100], 424 [$M^+$, -$HOCH_2OH$, 48]; HRMS (EI, 70 eV): *m/z* $[M]^+$ Calcd for $C_{28}H_{22}O_6$: 486.1137; Found:486.1139; IR (KBr) $cm^{-1}$: 3435, 3055, 2933, 1622, 1542, 1480, 1463, 1415, 1339, 1247, 1222, 1110, 1069, 1037, 1003, 954, 898, 808, 746, 699.

***7-Methoxy-5-(naphth-2-yl-sulfinyl)anthra[2,3-d][1,3]dioxole (2c)***

Yield: 36%, yellow solid, m.p. 174-176°C; $^1$H NMR ($CDCl_3$, 500 MHz): δ = 3.83 (s, 3H, $OCH_3$), 6.03 (s, 1H, $OCH_2O$), 6.06 (s, 1H, $OCH_2O$), 7.08 (d, $^3J_{HH}$ = 8.4 Hz, 1H, $H_{Ar}$), 7.18 (dd, $^3J_{HH}$ = 8.7, $^4J_{HH}$ = 1.8 Hz, 1H, $H_{Ar}$), 7.20 (s, 1H, $H_{Ar}$), 7.49-7.56 (m, 2H, 6,7-Naphth), 7.69 (d, 1H, $^3J_{HH}$ = 8.7 Hz, 1H, $H_{Ar}$), 7.78 (dd, $^3J_{HH}$ = 7.7, $^4J_{HH}$ = 1.3 Hz, 1H, $H_{Ar}$), 7.81 (d, $^3J_{HH}$ = 9.3 Hz, 1H, $H_{Ar}$), 7.90 (dd, $^3J_{HH}$ = 7.6, $^4J_{HH}$ = 1.2 Hz, 1H, $H_{Ar}$), 8.10 (d, $^4J_{HH}$ = 2.0 Hz, 1H, $H_{Ar}$), 8.22 (s, 1H, $H_{Ar}$), 8.28 (s, 1H, $H_{Ar}$), 8.38 (d, $^4J_{HH}$ = 1.0 Hz, 1H, $H_{Ar}$) ppm; $^{13}$C NMR ($CDCl_3$, 125 MHz): δ = 55.42, 98.72, 100.04, 101.55, 103.58 (2x$CH_{Ar}$), 119.98, 120.74, 124.57, 127.05, 127.22, 127.38, 127.81, 127.90, 127.96, 128.13, 128.34, 129.07, 130.31, 131.47, 132.85, 133.61, 141.86, 147.13, 150.25, 158.52; MS (EI, 70 eV) *m/z* (%): 426 [$M^+$, 42], 378 [$M^+$, -$OCH_2O$, 100], 299 [$M^+$, -$C_{10}H_7$, 15], 268 [$M^+$,-$C_{10}H_7$, -$OCH_3$, 25]; HRMS (EI, 70 eV): *m/z* $[M]^+$ Calcd for $C_{26}H_{18}O_4S$: 426.0926; Found: 426.0919; IR (KBr) $cm^{-1}$: 3437, 3051, 2921, 1626, 1460, 1378, 1281, 1255, 1221, 1194, 1122, 1066, 1033, 953, 896, 817, 747, 654.

***5-(4-Methoxyphenylsulfinyl)-anthra[2,3-d:6,7-d′]bis[1,3]dioxole (2d)***

Yield: 32%, yellow solid, m.p. 176-178°C (decomp.); $^1$H NMR ($CDCl_3$, 200 MHz): δ = 3.75 (s, 3H, $OCH_3$), 6.02 (s, 4H, 2x$OCH_2O$), 6.86 (d, $^3J_{HH}$ = 9.0 Hz, 2H, 2x$H_{Ar}$, 2x *o*-$C_6H_4$-OMe), 7.12 (s, 2H, 2x$H_{Ar}$), 7.24 (s, 1H, $H_{Ar}$), 7.38 (d, $^3J_{HH}$ = 9.0 Hz, 2H, 2x$H_{Ar}$, 2x *m*-$C_6H_4$-OMe), 8.09 (s, 2H, 2x$H_{Ar}$) ppm; $^{13}$C NMR ($CDCl_3$, 125 MHz): δ = 55.43, 98.90, 101.44 (2x$CH_2$), 103.10 (2x$CH_{Ar}$), 114.60 (2x$CH_{Ar}$), 126.07 (2x$CH_{Ar}$), 128.83, 129.69, 129.76 (2x$CH_{Ar}$),135.57 (2x$CH_{Ar}$), 147.35 (4x$C_{Ar}$), 149.27, 160.86 ppm; MS (EI, 70 eV) *m/z* (%): 420 [$M^+$, 16], 404 [$M^+$, -O, 100], 372 [$M^+$, -MeOH, 70]; HRMS (EI, 70 eV): *m/z* $[M]^+$ Calcd for $C_{23}H_{16}O_6S$: 420.0668; Found: 420.0668; IR (KBr) $cm^{-1}$: 3432, 2917, 1593, 1494, 1460, 1300, 1229, 1085, 1038, 952, 893, 827, 733.

***7-Methoxy-5-(naphth-2-yl-sulfonyl)anthra[2,3-d][1,3]dioxole (3c)***

Yield: 26%, yellow solid, m.p. 172-174°C (decomp.); $^1$H NMR ($CDCl_3$, 500 MHz): δ = 3.92 (s, 3H, $OCH_3$), 6.07 (s, 2H, $OCH_2O$), 7.11 (dd, 3JHH = 9.1, 4JHH = 2.3 Hz, 1H, $H_{Ar}$), 7.18 (s, 1H, $H_{Ar}$), 7.54-7.61 (m, 2H, 2x$H_{Ar}$), 7.72 ($d_{AB}$d, $^3J_{HHAB}$ = 8.7, $^4J_{HH}$ = 1.8 Hz, 1H, $H_{Ar}$), 7.79 (d, $^5J_{HH}$ = 0.3 Hz, 1H,$H_{Ar}$), 7.80 (d, $^5J_{HH}$ = 0.5 Hz, 1H, $H_{Ar}$), 7.81 ($d_{AB}$dd, $^3J_{HHAB}$ = 8.7, $^4J_{HH}$ = 2.5, $^4J_{HH}$ = 0.5 Hz, 1H, HAr), 7.92 (dd, $^3J_{HH}$ = 7.0, $^4J_{HH}$ = 2.0 Hz, 1H, $H_{Ar}$), 8.36 (s, 1H, $H_{Ar}$), 8.56-8.57 (m, 1H, $H_{Ar}$), 8.65 (d, $^4J_{HH}$ = 2.1 Hz, 1H, $H_{Ar}$), 8.86 (dd, $^5J_{HH}$ = 0.4, $^5J_{HH}$ = 0.7 Hz, 1H, $H_{Ar}$) ppm; $^{13}$C NMR ($CDCl_3$, 125 MHz): δ = 55.42, 100.78, 101.73, 102.02, 103.63, 119.64, 121.47, 124.69, 126.63, 126.92, 127.59, 127.95, 127.99, 128.77, 129.31, 129.43, 130.55, 130.73, 131.88, 131.96, 134.73, 134.84, 141.14, 146.70, 151.14, 159.16 ppm; MS (EI, 70 eV) *m/z* (%): 442 [$M^+$, 68], 377 [$M^+$, -$SO_2$, 34], 347 [$M^+$, -$SO_2$, -$OCH_3$, 100]; HRMS (EI, 70 eV): *m/z* $[M]^+$ Calcd for $C_{26}H_{18}O_5S$: 442.0875; Found: 442.0864; IR (KBr) $cm^{-1}$: 3437, 3057, 2909, 1626, 1479, 1462, 1378, 1350, 1303, 1280, 1245, 1222, 1195, 1146, 1127, 1070, 1040, 953, 902, 864, 818, 749, 668, 650, 573.

***5-(4-Methoxyphenylsulfonyl)-anthra[2,3-d:6,7-d′]bis[1,3]dioxole (3d)***

Yield: 17%, yellow solid, m.p. 195-198°C (decomp.); $^1$H NMR ($CDCl_3$, 200 MHz): δ = 3.77 (s, 3H, $OCH_3$). 6.05 (s, 4H, $OCH_2O$), 6.86 (d, $^3J_{HH}$ = 8.0Hz, 2H, 2x$H_{Ar}$, 2x *o*-$C_6H_4$-OMe), 7.09 (s, 2H, 2x$H_{Ar}$), 7.82 (d, $^3J_{HH}$ = 8.0 Hz, 2H, 2x$H_{Ar}$, 2x *m*-$C_6H_4$-OMe), 8.16 (s, 1H, $H_{Ar}$), 8.70 (s, 2H, 2x $H_{Ar}$) ppm; $^{13}$C NMR ($CDCl_3$, 125 MHz): δ = 55.58, 101.04 (2x$CH_{Ar}$), 101.61 (2x$CH_{Ar}$), 103.08 (2x$CH_{Ar}$), 114.23 (2x$CH_{Ar}$), 127.35, 127.99 (2x$CH_{Ar}$), 128.52, 128.75 (2x$C_{Ar}$), 132.83 (2x$C_{Ar}$), 135.88, 146.88 (2x$C_{Ar}$), 150.16 (2x$C_{Ar}$), 162.82 ppm; MS (EI, 70 eV) *m/z* (%): 436 [$M^+$, 100], 372 [$M^+$, -$SO_2$, 43], 342 [$M^+$, -2x$OCH_2OH$ 78]; HRMS (EI, 70 eV): *m/z* $[M]^+$ Calcd for $C_{23}H_{16}O_7S$: 436.0617; Found: 436.0610; IR (KBr) $cm^{-1}$: 3434, 2905, 1620, 1592, 1500, 1460, 1378, 1303, 1265, 1232, 1149, 1087, 1035, 952, 877, 837, 735, 676, 574.

# $^{1}$H NMR and $^{13}$C NMR spectra

**Figure S1.** $^1$H NMR and $^{13}$C NMR spectra of the obtained compounds.

## $^{1}$H NMR of 1a

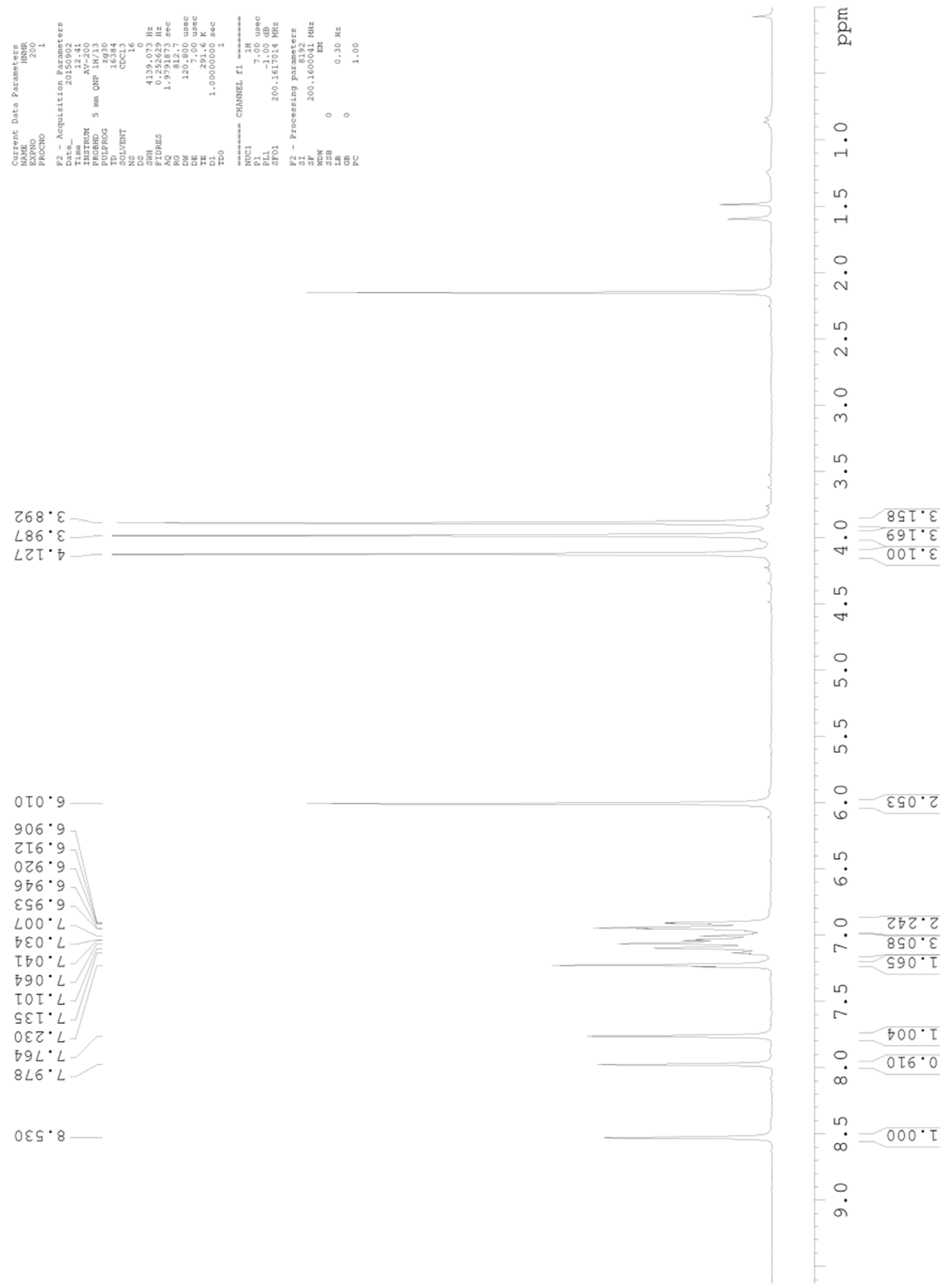

## $^{13}$C NMR of 1a

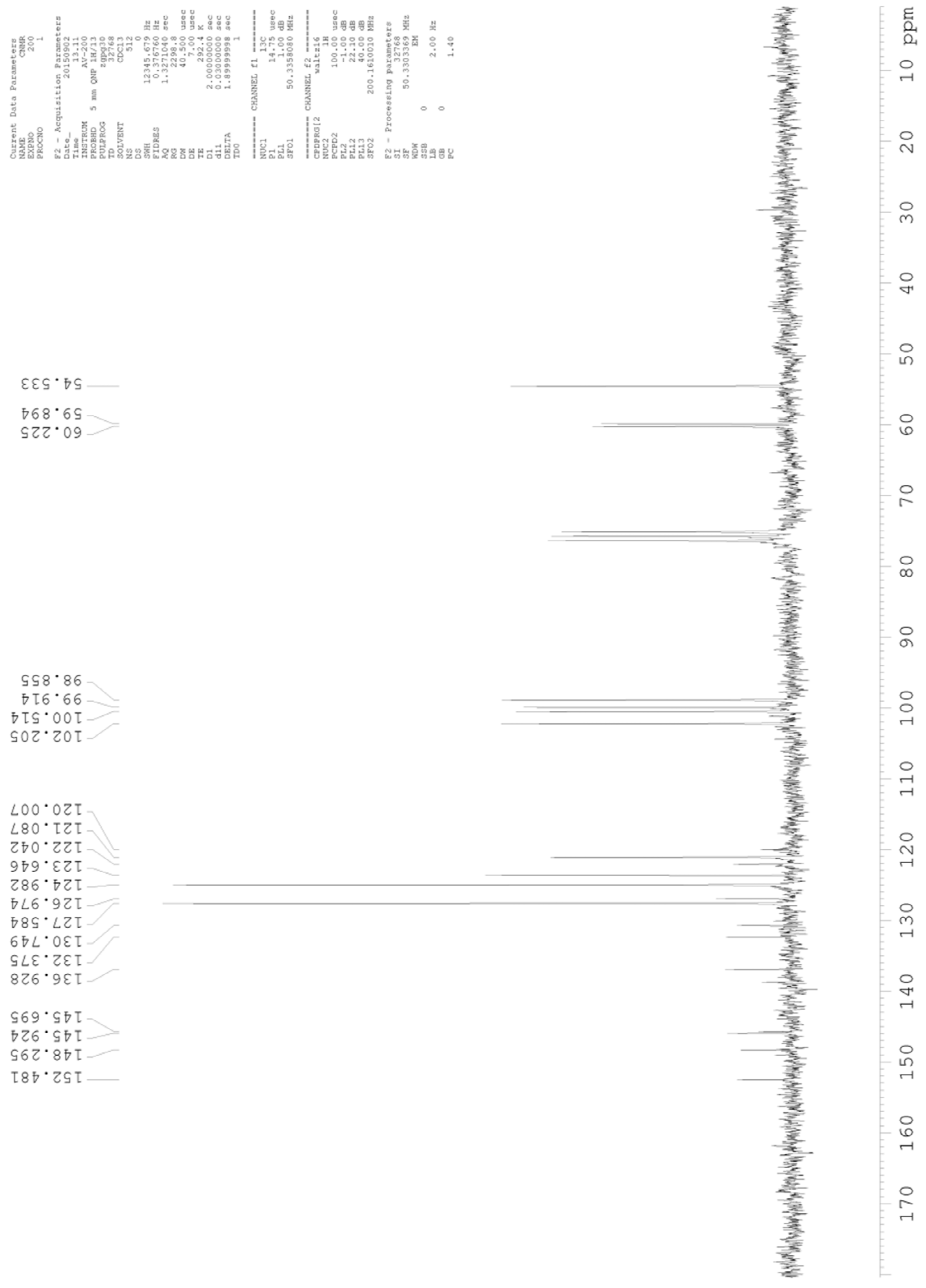

54.533
59.894
60.225
98.855
99.914
100.514
102.205
120.007
121.087
122.042
123.646
124.982
126.974
127.584
130.749
132.375
136.928
145.695
145.924
148.295
152.481
ppm

## $^1$H NMR of 1b

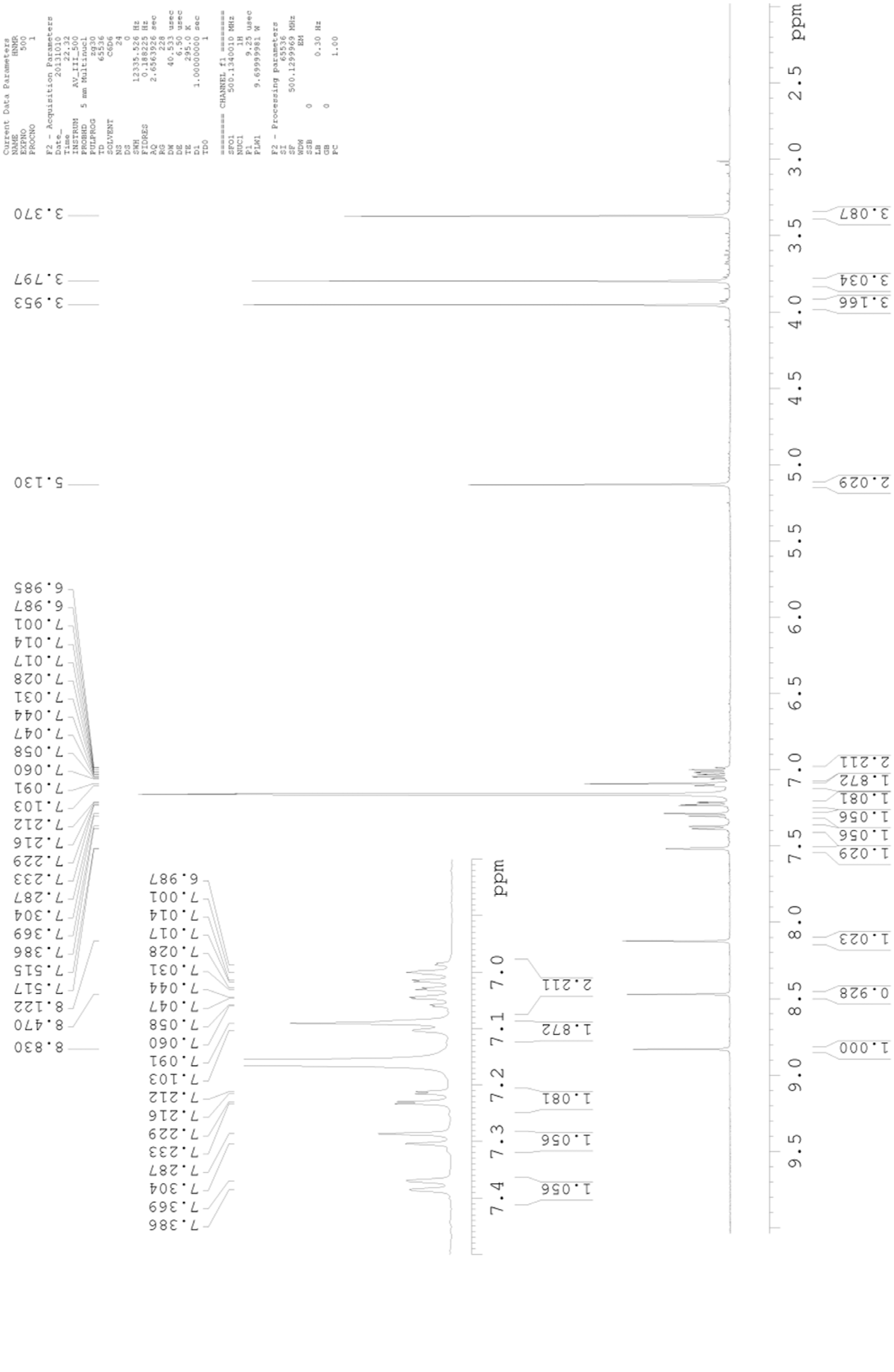

## $^{13}$C NMR of 1b

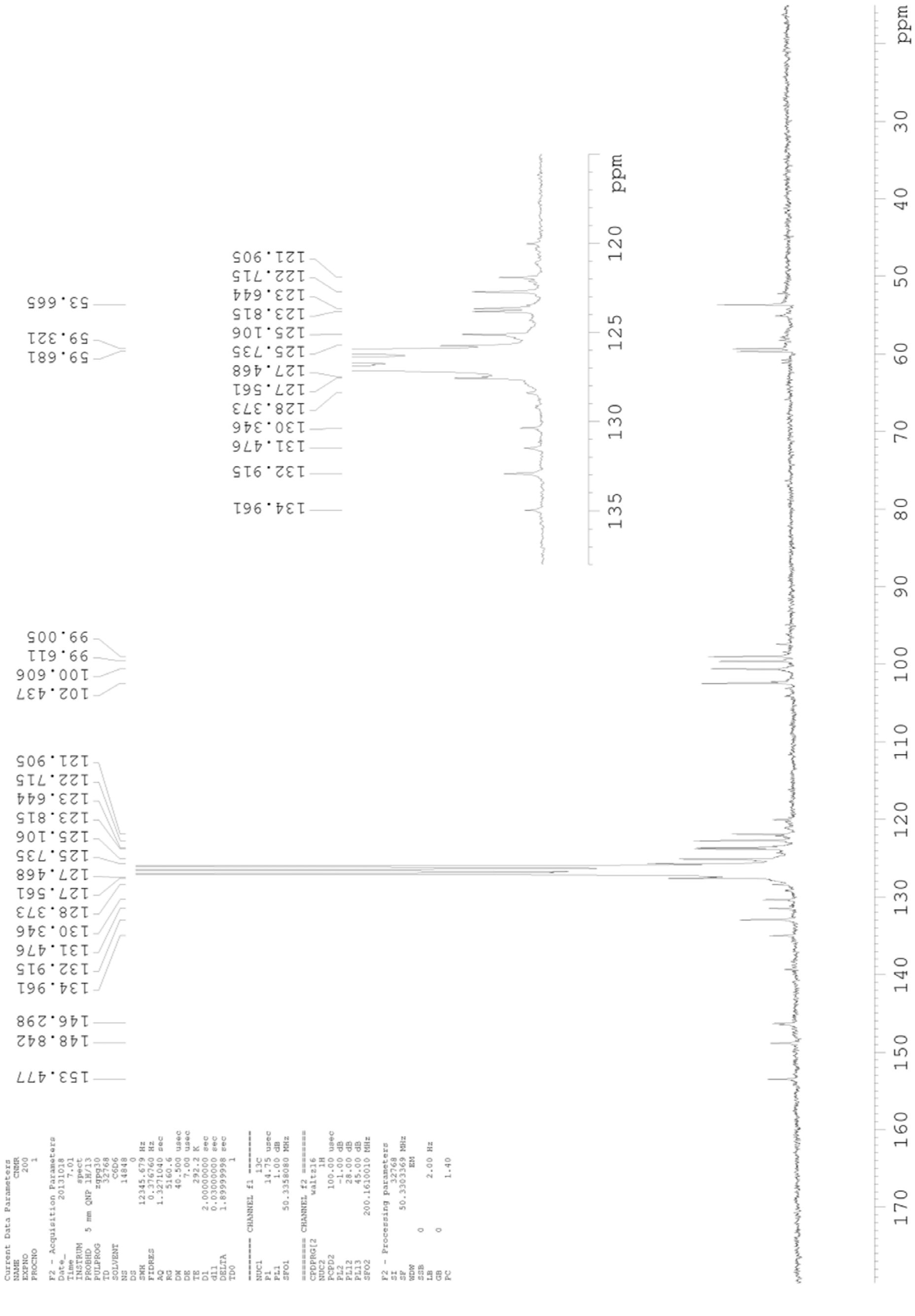

# $^1$H NMR of 1c

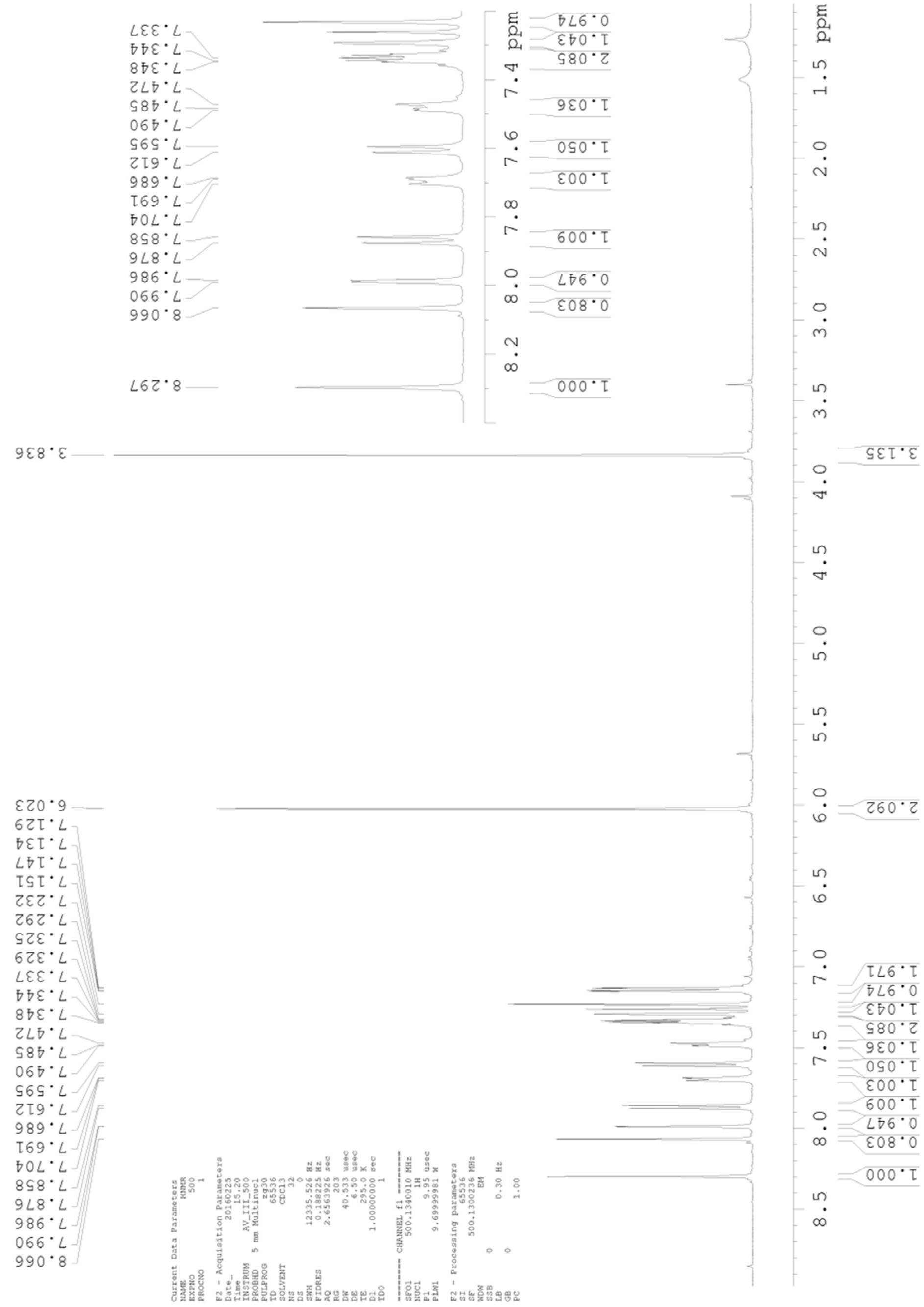

8.297
8.066
7.990
7.986
7.876
7.858
7.704
7.691
7.686
7.612
7.595
7.490
7.485
7.472
7.348
7.344
7.337
6.023
3.836
7.129
7.134
7.147
7.151
7.232
7.292
7.325
7.329
1.000
0.803
0.947
1.009
1.003
1.050
1.036
2.085
1.043
0.974
1.971
2.092
3.135
ppm

**$^{13}$C NMR of 1c**

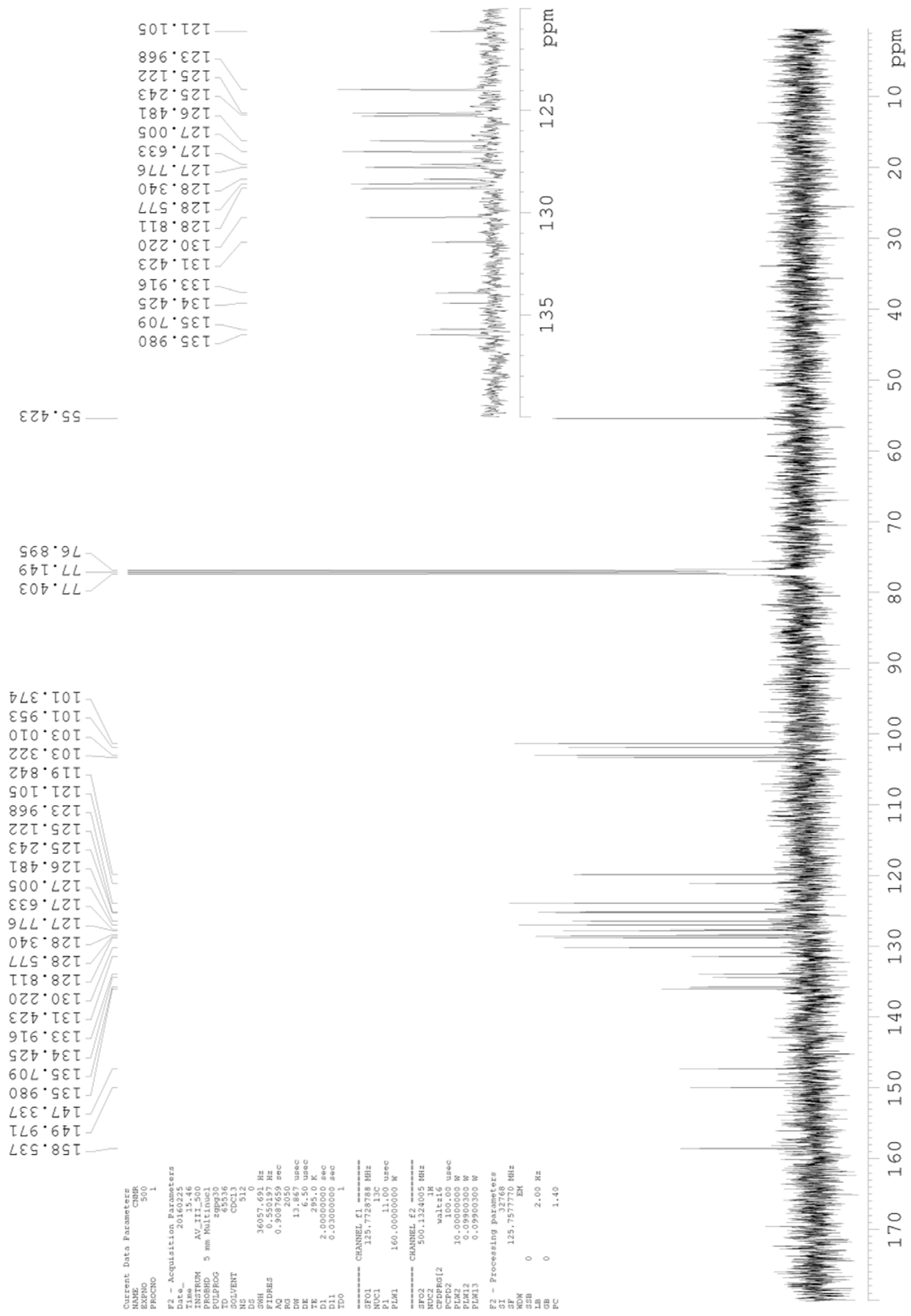
158.537
149.971
147.337
135.980
135.709
134.425
133.916
131.423
130.220
128.811
128.577
128.340
127.776
127.633
127.005
126.481
125.243
125.122
123.968
121.105
119.842
103.322
103.010
101.953
101.374
77.403
77.149
76.895
55.423
ppm

**$^1$H NMR of 1d**

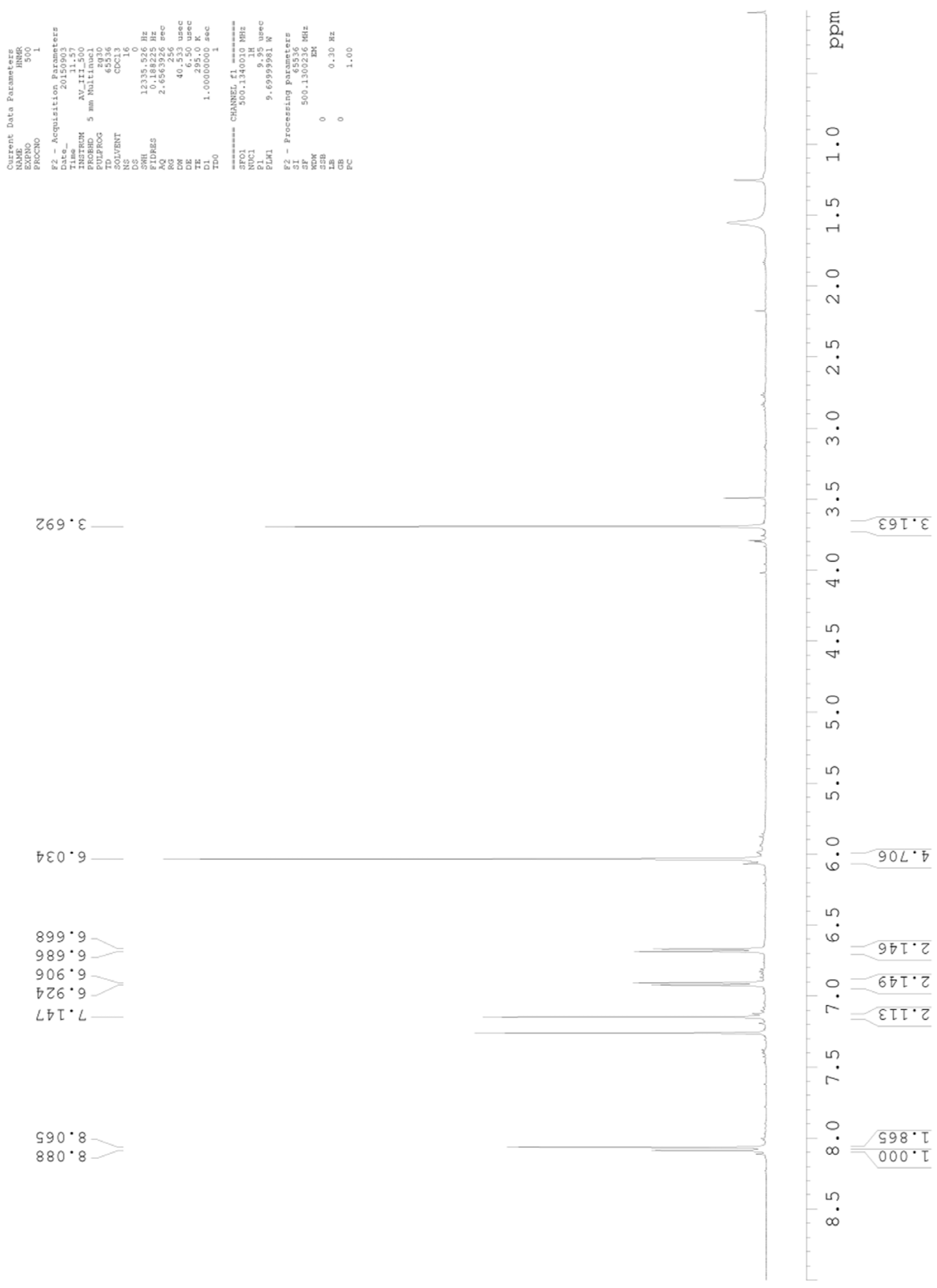
3.692
6.034
6.668
6.686
6.906
6.924
7.147
8.065
8.088
3.163
4.706
2.146
2.149
2.113
1.865
1.000
ppm

**$^{13}$C NMR of 1d**

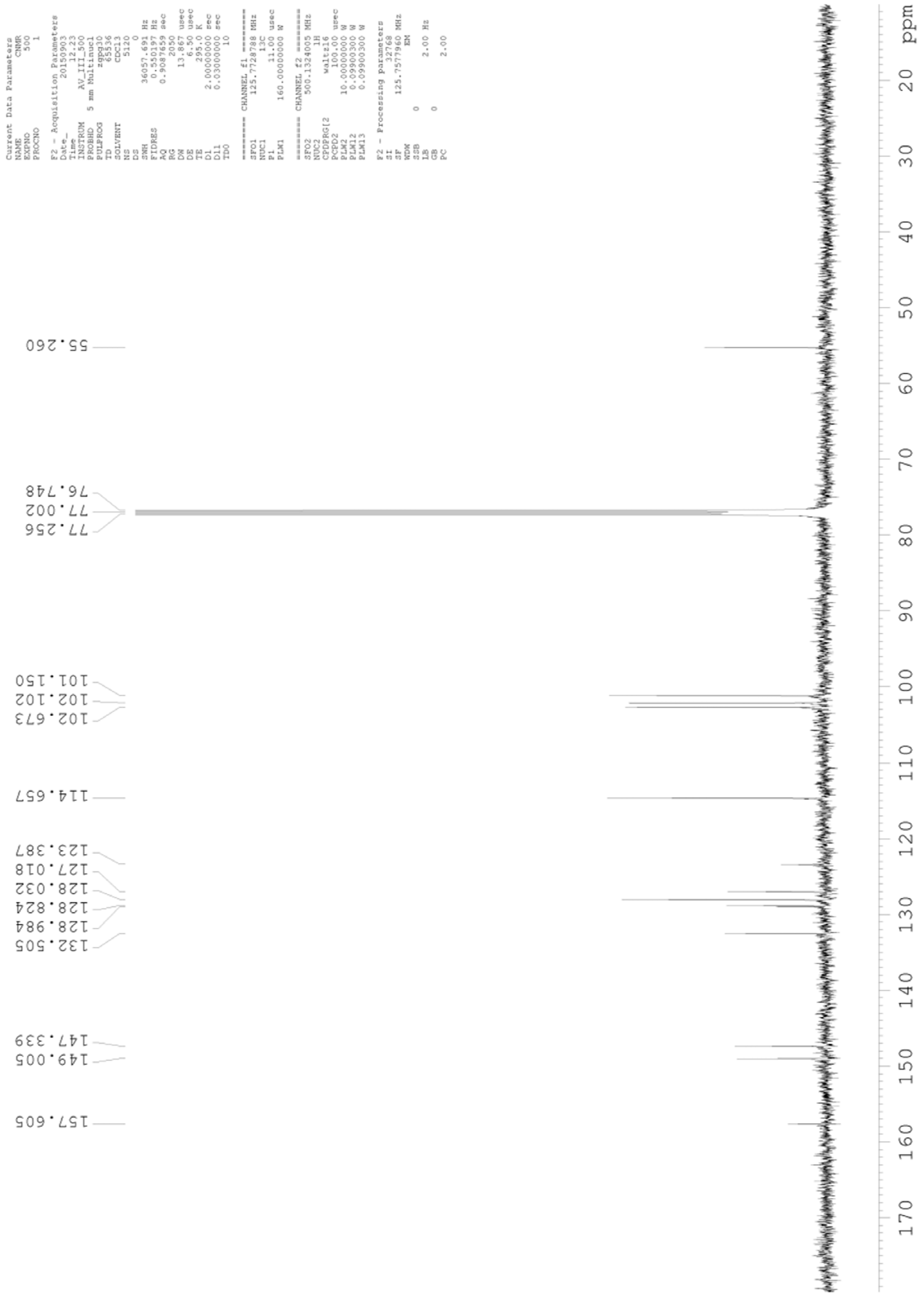
55.260
76.748
77.002
77.256
101.150
102.102
102.673
114.657
123.387
127.018
128.032
128.824
128.984
132.505
147.339
149.005
157.605
ppm

**$^{1}$H NMR of 2a**

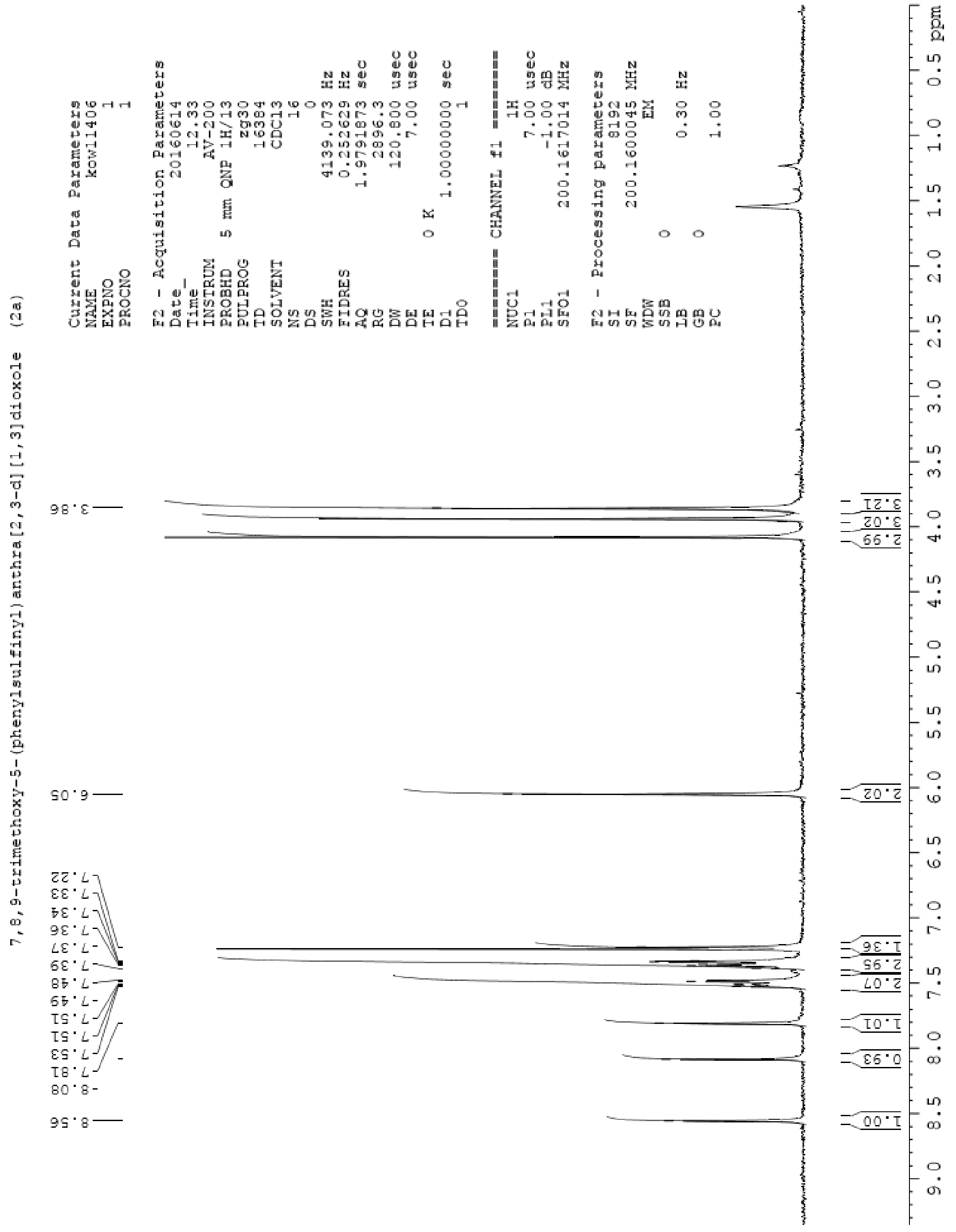
7,8,9-trimethoxy-5-(phenylsulfinyl)anthra[2,3-d][1,3]dioxole (2a)
Current Data Parameters
NAME kowl1406
EXPNO 1
PROCNO 1
F2 - Acquisition Parameters
Date_ 20160614
Time_ 12.33
INSTRUM AV-200
PROBHD 5 mm QNP 1H/13
PULPROG zg30
TD 16384
SOLVENT CDCl3
NS 16
DS 0
SWH 4139.073 Hz
FIDRES 0.252629 Hz
AQ 1.9791873 sec
RG 2896.3
DW 120.800 usec
DE 7.00 usec
TE 0 K
D1 1.00000000 sec
TD0 1
======== CHANNEL f1 ========
NUC1 1H
P1 7.00 usec
PL1 -1.00 dB
SFO1 200.1617014 MHz
F2 - Processing parameters
SI 8192
SF 200.1600045 MHz
WDW EM
SSB 0
LB 0.30 Hz
GB 0
PC 1.00
8.56
8.08
7.81
7.53
7.51
7.51
7.49
7.48
7.39
7.37
7.36
7.34
7.33
7.22
6.05
3.86
1.00
0.93
1.01
2.07
2.95
1.36
2.02
2.99
3.02
3.21
9.0 8.5 8.0 7.5 7.0 6.5 6.0 5.5 5.0 4.5 4.0 3.5 3.0 2.5 2.0 1.5 1.0 0.5 ppm

**$^{13}$C NMR of 2a**

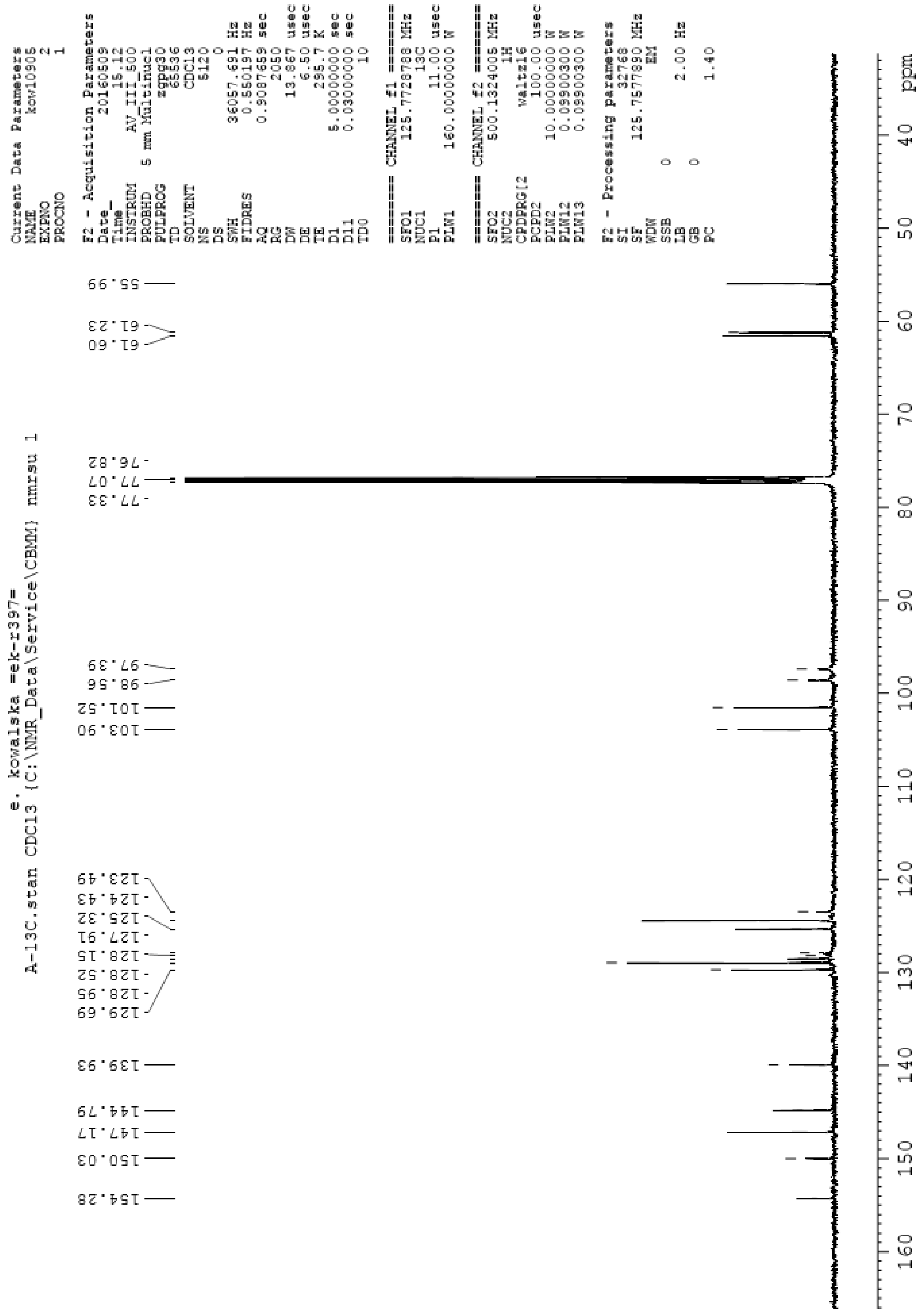
e. kowalska =ek-r397=
A-13C.stan CDCl3 {C:\NMR_Data\Service\CBMM} nmrsu 1
55.99
61.23
61.60
76.82
77.07
77.33
97.39
98.56
101.52
103.90
123.49
124.43
125.32
127.91
128.15
128.52
128.95
129.69
139.93
144.79
147.17
150.03
154.28
Current Data Parameters
NAME kow10905
EXPNO 2
PROCNO 1
F2 - Acquisition Parameters
Date_ 20160509
Time 15.12
INSTRUM AV_III_500
PROBHD 5 mm Multinucl
PULPROG zgpg30
TD 65536
SOLVENT CDCl3
NS 5120
DS 0
SWH 36057.691 Hz
FIDRES 0.550197 Hz
AQ 0.9087659 sec
RG 2050
DW 13.867 usec
DE 6.50 usec
TE 295.7 K
D1 5.00000000 sec
D11 0.03000000 sec
TD0 10
======== CHANNEL f1 ========
SFO1 125.7728788 MHz
NUC1 13C
P1 11.00 usec
PLW1 160.00000000 W
======== CHANNEL f2 ========
SFO2 500.1324005 MHz
NUC2 1H
CPDPRG[2 waltz16
PCPD2 100.00 usec
PLW2 10.00000000 W
PLW12 0.09900300 W
PLW13 0.09900300 W
F2 - Processing parameters
SI 32768
SF 125.7577890 MHz
WDW EM
SSB 0
LB 2.00 Hz
GB 0
PC 1.40
40
50
60
70
80
90
100
110
120
130
140
150
160
ppm

**¹H NMR of 2b**

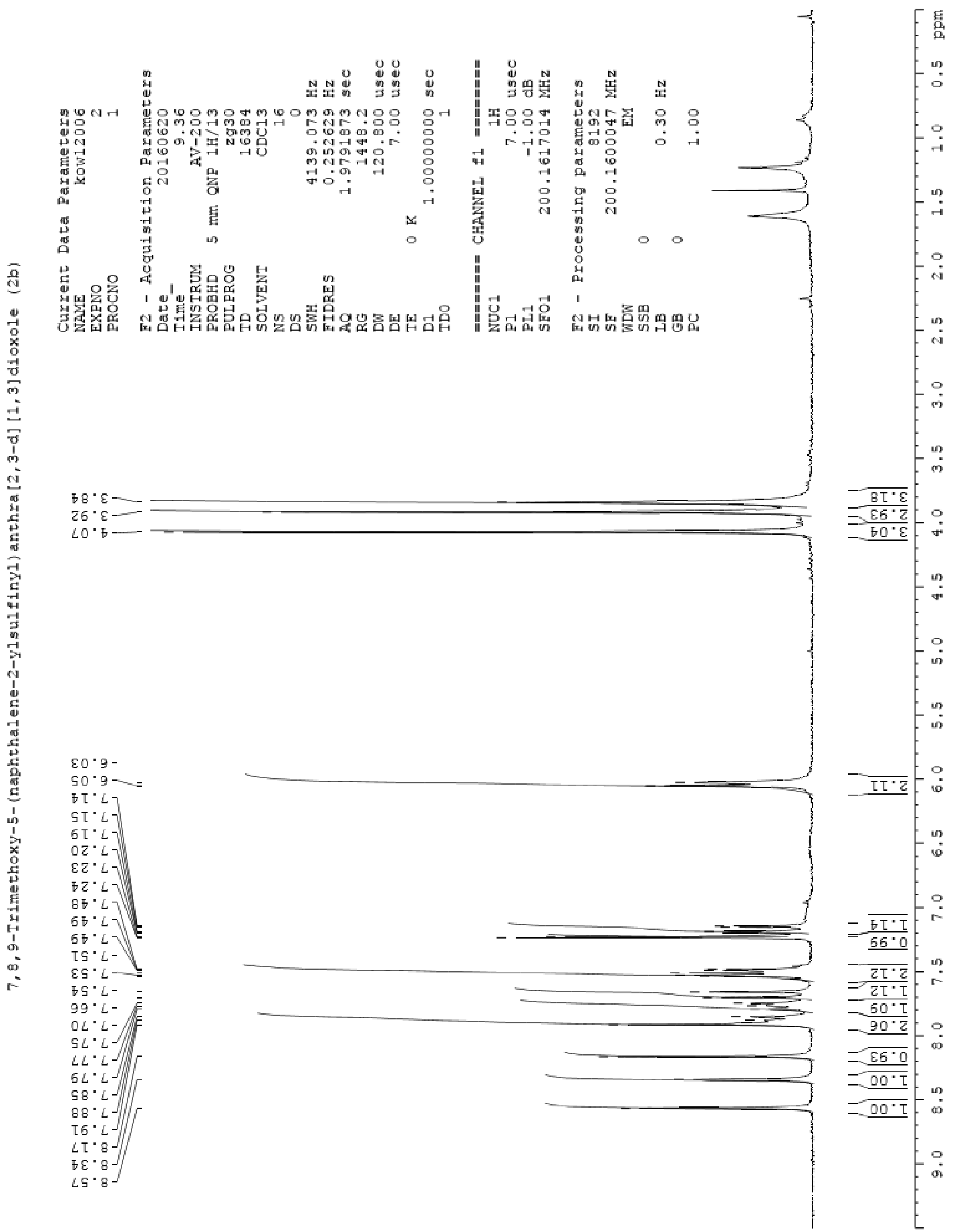
7,8,9-Trimethoxy-5-(naphthalene-2-ylsulfinyl)anthra[2,3-d][1,3]dioxole (2b)
Current Data Parameters
NAME kowl2006
EXPNO 2
PROCNO 1
F2 - Acquisition Parameters
Date_ 20160620
Time_ 9.36
INSTRUM AV-200
PROBHD 5 mm QNP 1H/13
PULPROG zg30
TD 16384
SOLVENT CDCl3
NS 16
DS 0
SWH 4139.073 Hz
FIDRES 0.252629 Hz
AQ 1.9791873 sec
RG 1448.2
DW 120.800 usec
DE 7.00 usec
TE 0 K
D1 1.00000000 sec
TD0 1
======== CHANNEL f1 ========
NUC1 1H
P1 7.00 usec
PL1 -1.00 dB
SFO1 200.1617014 MHz
F2 - Processing parameters
SI 8192
SF 200.1600047 MHz
WDW EM
SSB 0
LB 0.30 Hz
GB 0
PC 1.00
ppm

**$^{13}$C NMR of 2b**

7,8,9-Trimethoxy-5-(naphthalene-2-ylsulfinyl)anthra[2,3-d][1,3]dioxole (2b)

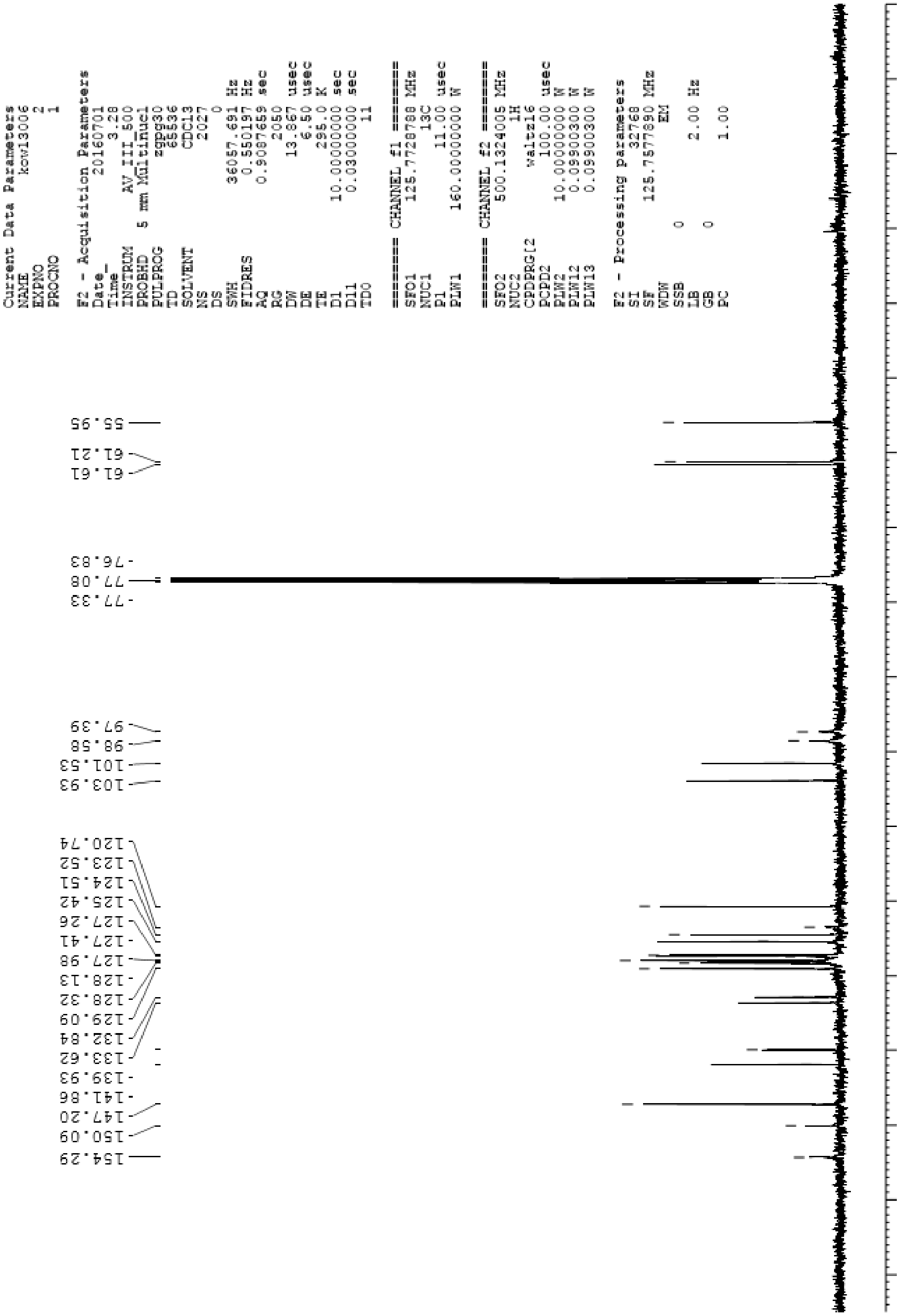

## ¹H NMR of 2c

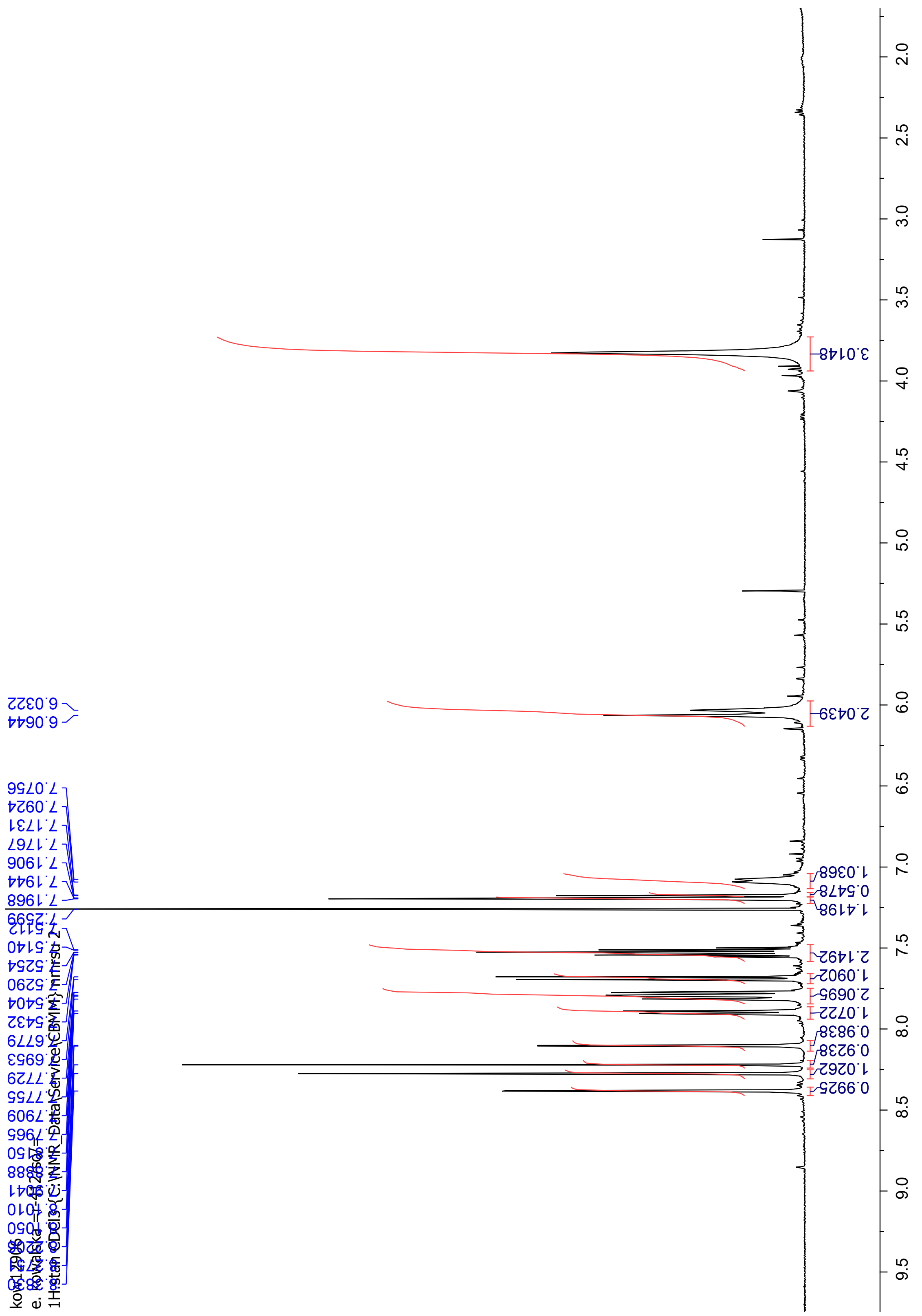

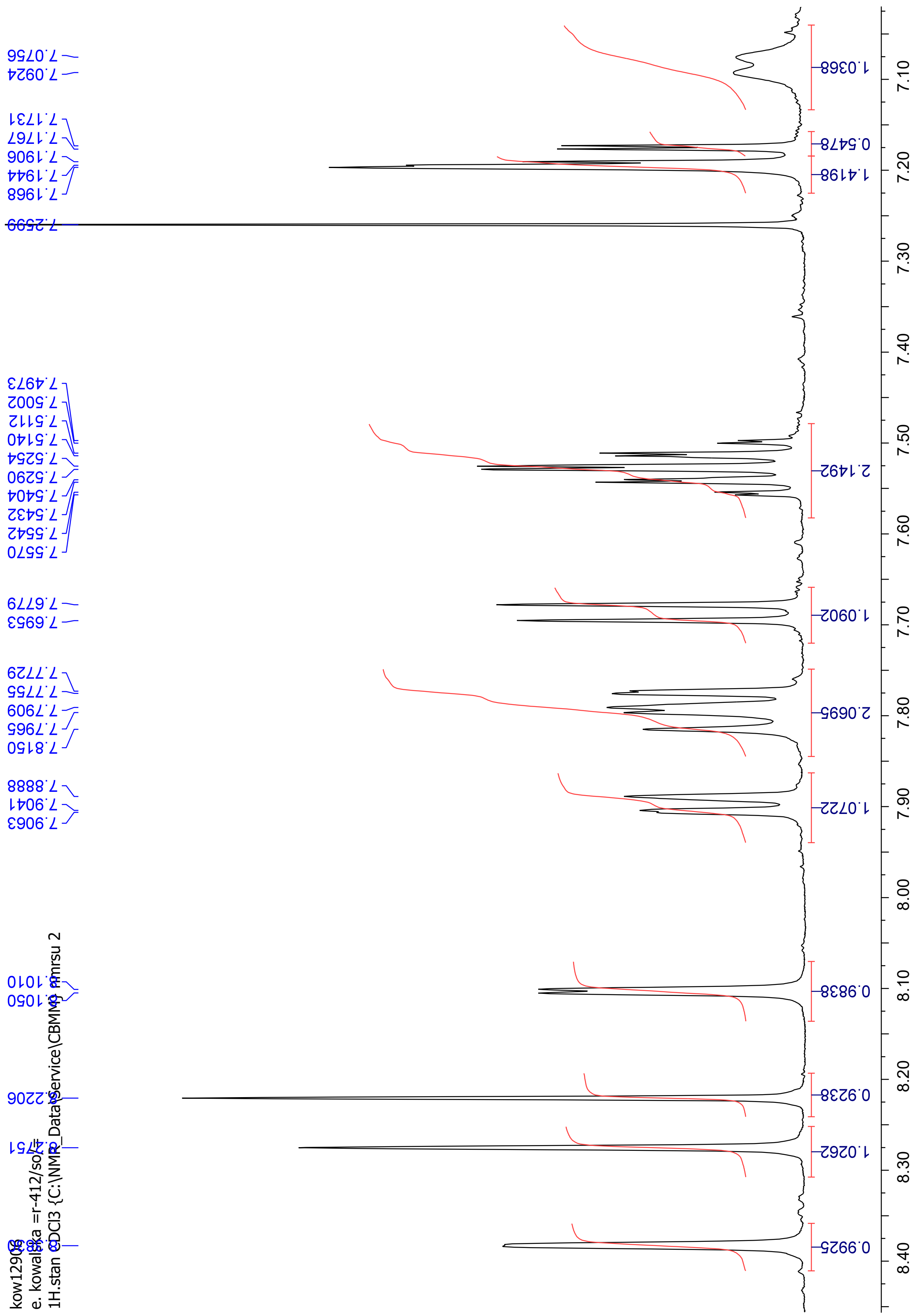
kow12909
e. kowalska =r-412/so/z
1H.stan CDCl3 {C:\NMR_Data\Service\CBMM} nmrsu 2
7.0756
7.0924
7.1731
7.1767
7.1906
7.1944
7.1968
7.2599
7.4973
7.5002
7.5112
7.5140
7.5254
7.5290
7.5404
7.5432
7.5542
7.5570
7.6779
7.6953
7.7729
7.7755
7.7909
7.7965
7.8150
7.8888
7.9041
7.9063
8.1010
8.1050
8.2206
8.2751
1.0368
0.5478
1.4198
2.1492
1.0902
2.0695
1.0722
0.9838
0.9238
1.0262
0.9925
7.10
7.20
7.30
7.40
7.50
7.60
7.70
7.80
7.90
8.00
8.10
8.20
8.30
8.40

## $^{13}$C NMR of 2c

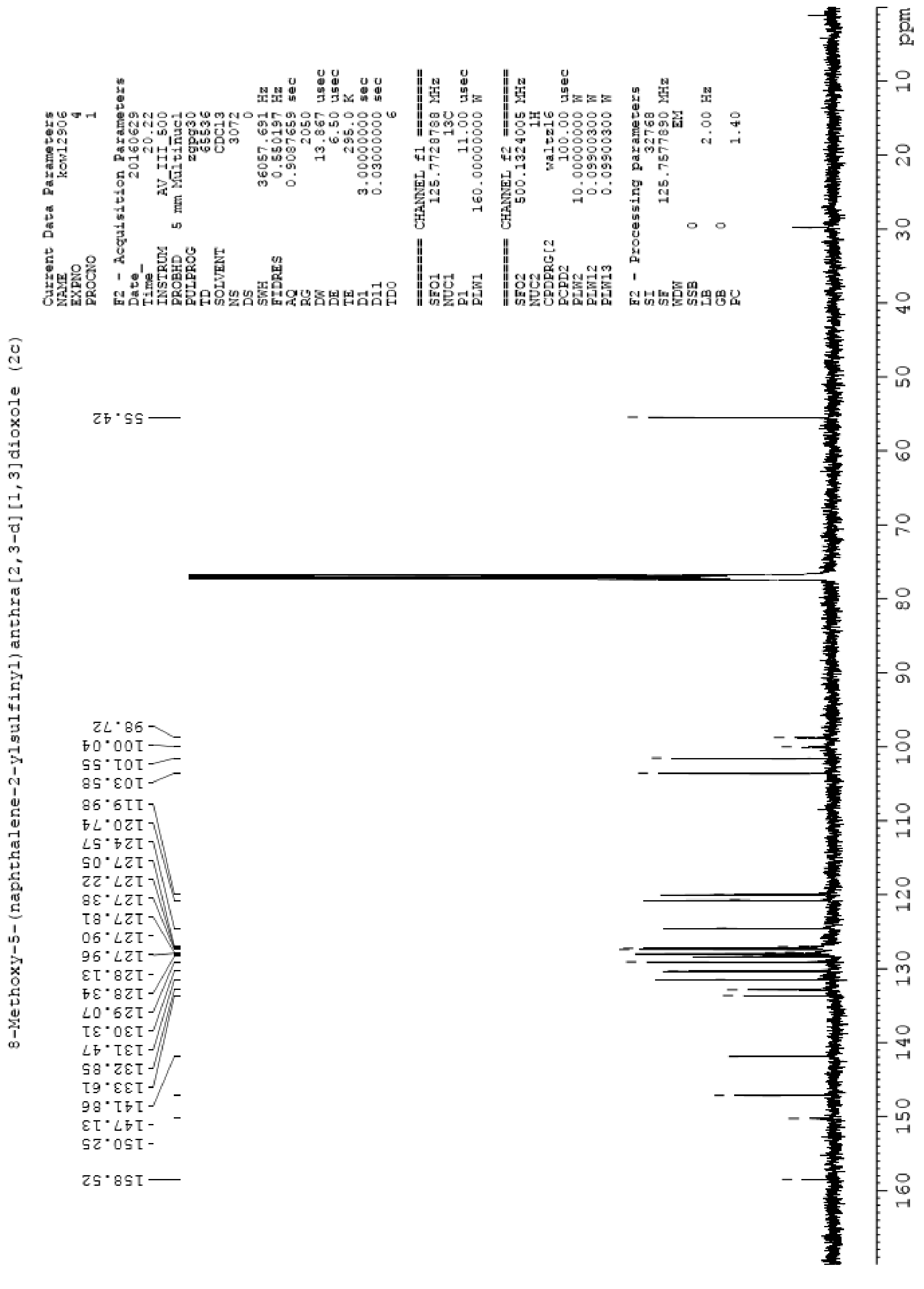
8-Methoxy-5-(naphthalene-2-ylsulfinyl)anthra[2,3-d][1,3]dioxole (2c)
55.42
98.72
100.04
101.55
103.58
119.98
120.74
124.57
127.05
127.22
127.38
127.81
127.90
127.96
128.13
128.34
129.07
130.31
131.47
132.85
133.61
141.86
147.13
150.25
158.52
Current Data Parameters
NAME kcw12906
EXPNO 4
PROCNO 1
F2 - Acquisition Parameters
Date_ 20160629
Time 20.22
INSTRUM AV_III_500
PROBHD 5 mm Multinucl
PULPROG zgpg30
TD 65536
SOLVENT CDCl3
NS 3072
DS 0
SWH 36057.691 Hz
FIDRES 0.550197 Hz
AQ 0.9087659 sec
RG 2050
DW 13.867 usec
DE 6.50 usec
TE 295.0 K
D1 3.00000000 sec
D11 0.03000000 sec
TD0 6
======== CHANNEL f1 ========
SFO1 125.7728788 MHz
NUC1 13C
P1 11.00 usec
PLW1 160.00000000 W
======== CHANNEL f2 ========
SFO2 500.1324005 MHz
NUC2 1H
CPDPRG[2 waltz16
PCPD2 100.00 usec
PLW2 10.00000000 W
PLW12 0.09900300 W
PLW13 0.09900300 W
F2 - Processing parameters
SI 32768
SF 125.7577890 MHz
WDW EM
SSB 0
LB 2.00 Hz
GB 0
PC 1.40
ppm

## $^1H$ NMR of 2d

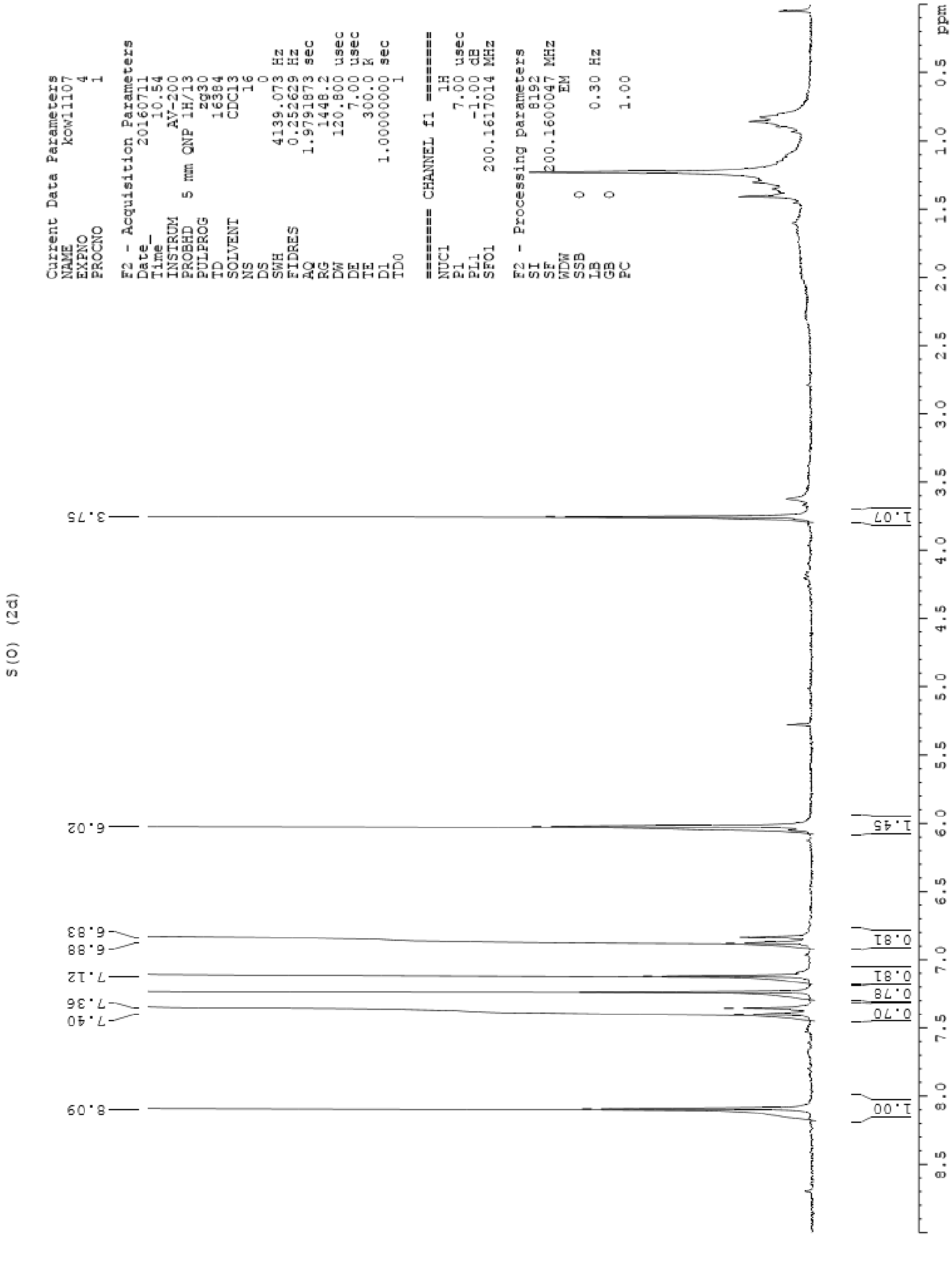
S(O) (2d)
Current Data Parameters
NAME kow11107
EXPNO 4
PROCNO 1
F2 - Acquisition Parameters
Date_ 20160711
Time 10.54
INSTRUM AV-200
PROBHD 5 mm QNP 1H/13
PULPROG zg30
TD 16384
SOLVENT CDCl3
NS 16
DS 0
SWH 4139.073 Hz
FIDRES 0.252629 Hz
AQ 1.9791873 sec
RG 1448.2
DW 120.800 usec
DE 7.00 usec
TE 300.0 K
D1 1.00000000 sec
TD0 1
======== CHANNEL f1 ========
NUC1 1H
P1 7.00 usec
PL1 -1.00 dB
SFO1 200.1617014 MHz
F2 - Processing parameters
SI 8192
SF 200.1600047 MHz
WDW EM
SSB 0
LB 0.30 Hz
GB 0
PC 1.00
8.09
7.40
7.36
7.12
6.88
6.83
6.02
3.75
1.00
0.70
0.78
0.81
0.81
1.45
1.07
ppm

## $^{13}C$ NMR of 2d

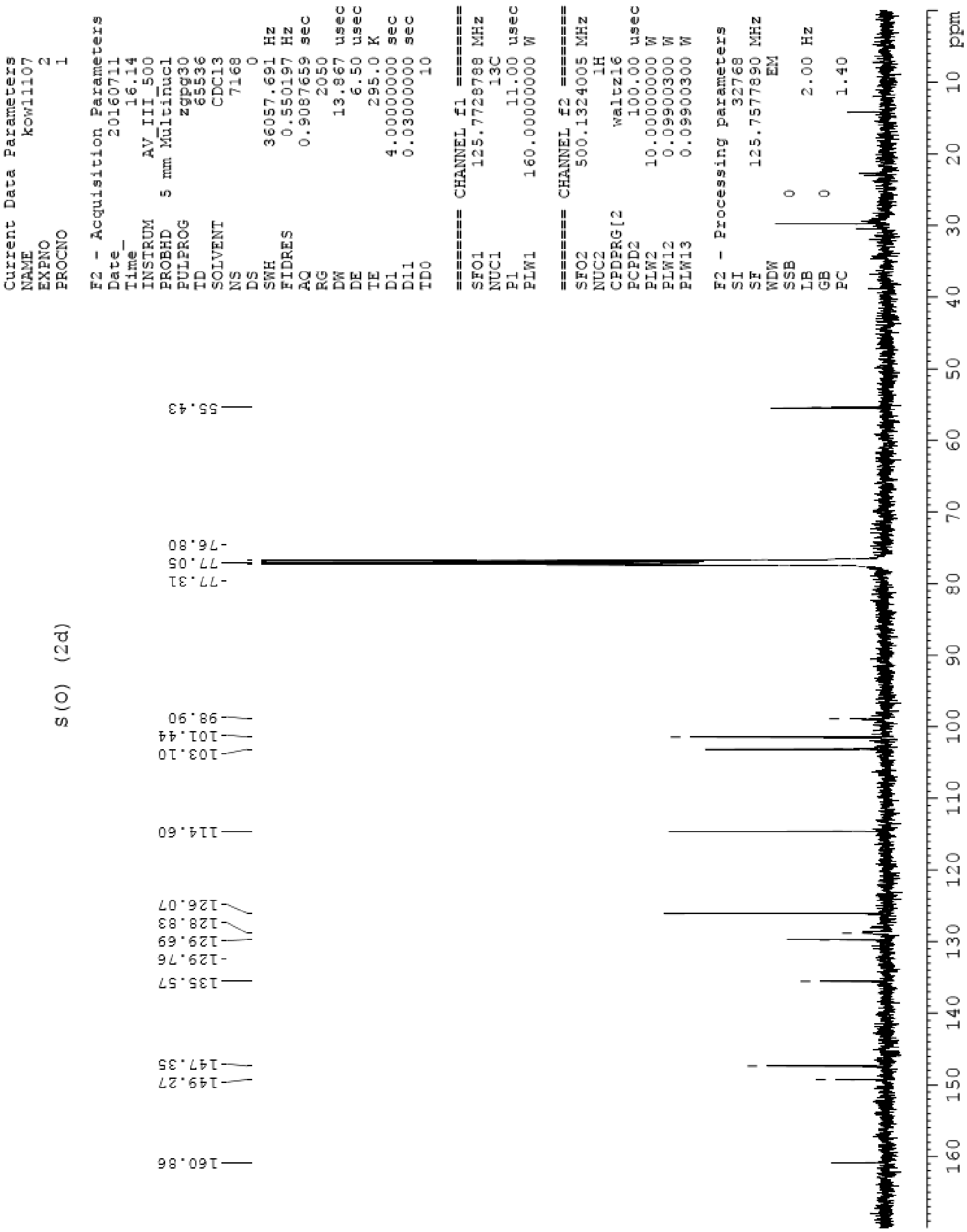
Current Data Parameters
NAME kowl1107
EXPNO 2
PROCNO 1
F2 - Acquisition Parameters
Date_ 20160711
Time 16.14
INSTRUM AV_III_500
PROBHD 5 mm Multinucl
PULPROG zgpg30
TD 65536
SOLVENT CDCl3
NS 7168
DS 0
SWH 36057.691 Hz
FIDRES 0.550197 Hz
AQ 0.9087659 sec
RG 2050
DW 13.867 usec
DE 6.50 usec
TE 295.0 K
D1 4.00000000 sec
D11 0.03000000 sec
TD0 10
======== CHANNEL f1 ========
SFO1 125.7728788 MHz
NUC1 13C
P1 11.00 usec
PLW1 160.00000000 W
======== CHANNEL f2 ========
SFO2 500.1324005 MHz
NUC2 1H
CPDPRG[2 waltz16
PCPD2 100.00 usec
PLW2 10.00000000 W
PLW12 0.09900300 W
PLW13 0.09900300 W
F2 - Processing parameters
SI 32768
SF 125.7577890 MHz
WDW EM
SSB 0
LB 2.00 Hz
GB 0
PC 1.40
S(O) (2d)
160.86
149.27
147.35
135.57
129.76
129.69
128.83
126.07
114.60
103.10
101.44
98.90
77.31
77.05
76.80
55.43
160 150 140 130 120 110 100 90 80 70 60 50 40 30 20 10 ppm

## $^{1}$H NMR of 3c

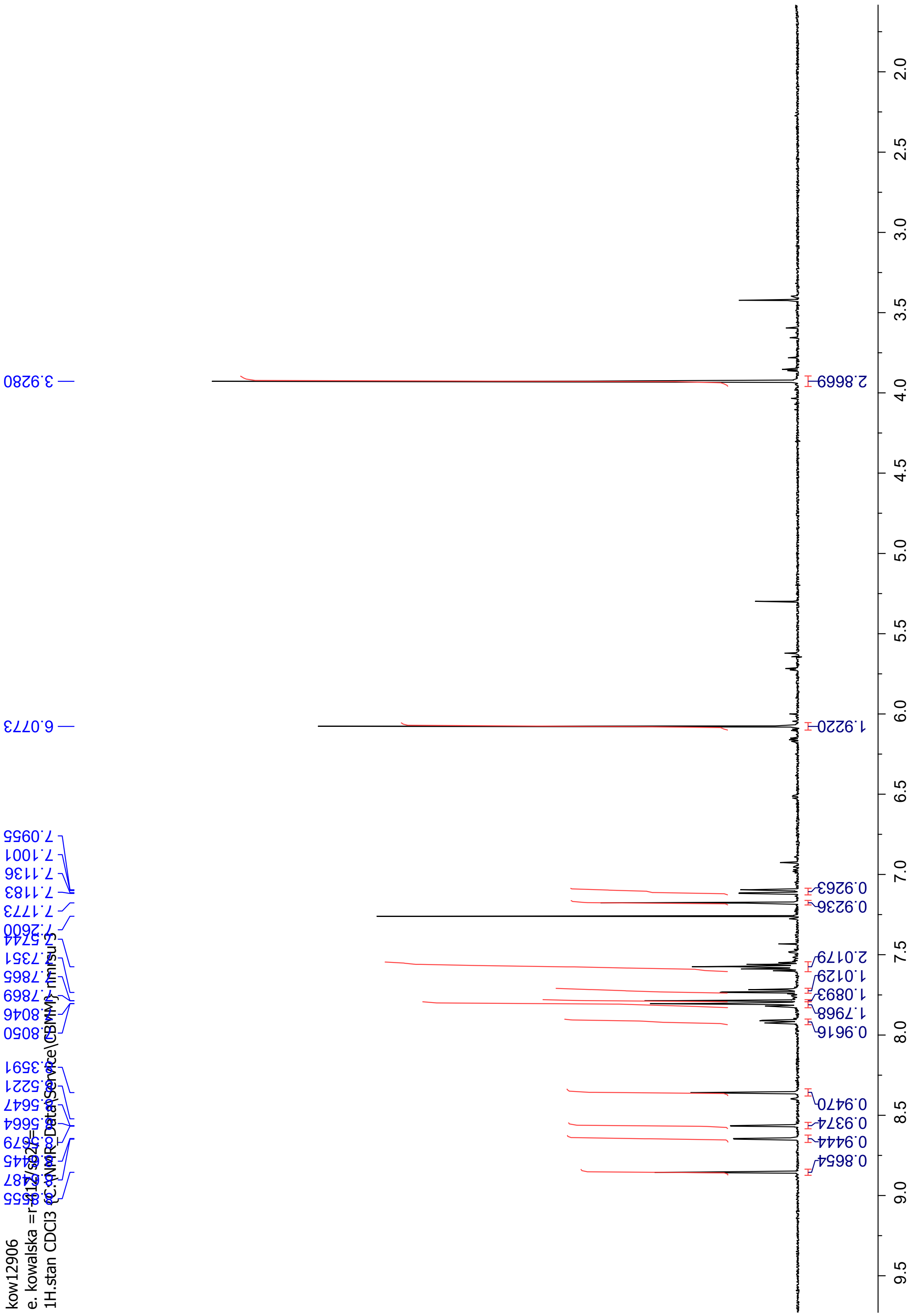
kow12906
1H.stan CDCl3
3.9280
6.0773
2.8669
1.9220
0.9263
0.9236
2.0179
1.0129
1.0893
1.7968
0.9616
0.9470
0.9374
0.9444
0.8654
2.0
2.5
3.0
3.5
4.0
4.5
5.0
5.5
6.0
6.5
7.0
7.5
8.0
8.5
9.0
9.5

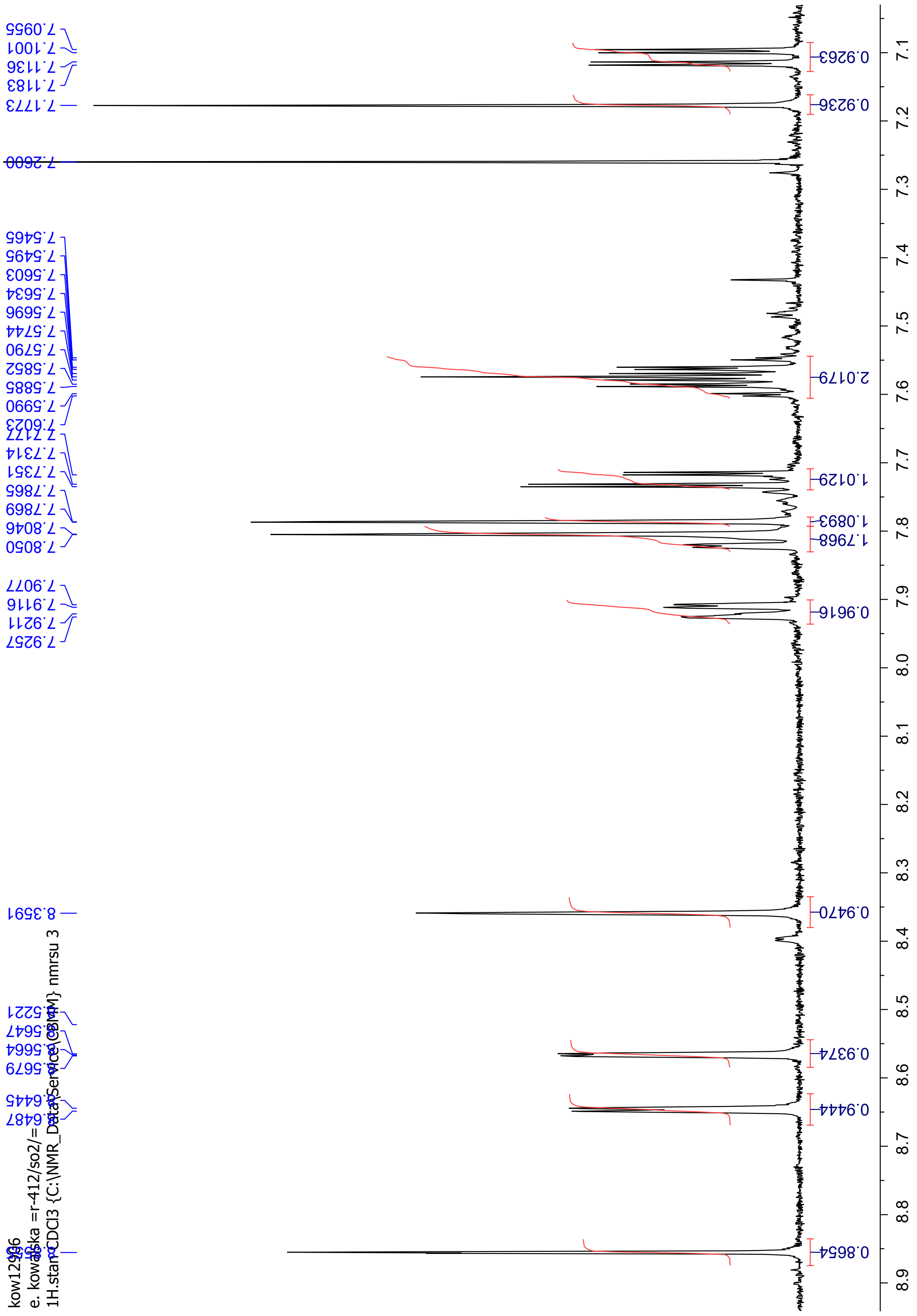
kow12996
7.0955
7.1001
7.1136
7.1183
7.1773
7.2600
7.5465
7.5495
7.5603
7.5634
7.5696
7.5744
7.5790
7.5852
7.5885
7.5990
7.6023
7.7314
7.7351
7.7865
7.7869
7.8046
7.8050
7.9077
7.9116
7.9211
7.9257
8.3591
0.9263
0.9236
2.0179
1.0129
1.0893
1.7968
0.9616
0.9470
0.9374
0.9444
0.8654
7.1
7.2
7.3
7.4
7.5
7.6
7.7
7.8
7.9
8.0
8.1
8.2
8.3
8.4
8.5
8.6
8.7
8.8
8.9

## $^{13}$C NMR of 3c

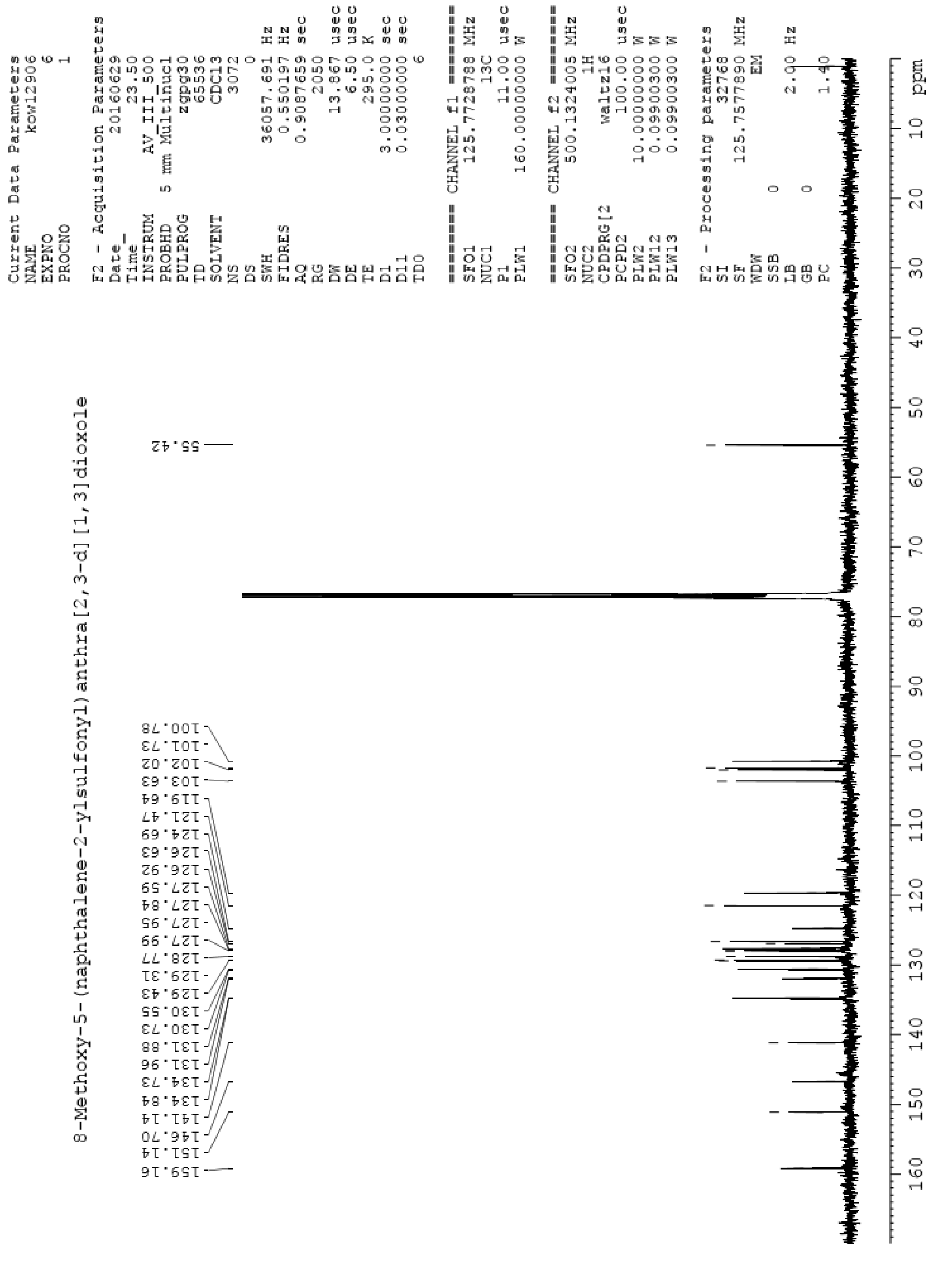
8-Methoxy-5-(naphthalene-2-ylsulfonyl)anthra[2,3-d][1,3]dioxole
55.42
100.78
101.73
102.02
103.63
119.64
121.47
124.69
126.63
126.92
127.59
127.84
127.95
127.99
128.77
129.31
129.43
130.55
130.73
131.88
131.96
134.73
134.84
141.14
146.70
151.14
159.16
Current Data Parameters
NAME kow12906
EXPNO 6
PROCNO 1
F2 - Acquisition Parameters
Date_ 20160629
Time_ 23.50
INSTRUM AV_III_500
PROBHD 5 mm Multinucl
PULPROG zgpg30
TD 65536
SOLVENT CDCl3
NS 3072
DS 0
SWH 36057.691 Hz
FIDRES 0.550197 Hz
AQ 0.9087659 sec
RG 2050
DW 13.867 usec
DE 6.50 usec
TE 295.0 K
D1 3.00000000 sec
D11 0.03000000 sec
TD0 6
======== CHANNEL f1 ========
SFO1 125.7728788 MHz
NUC1 13C
P1 11.00 usec
PLW1 160.00000000 W
======== CHANNEL f2 ========
SFO2 500.1324005 MHz
NUC2 1H
CPDPRG[2 waltz16
PCPD2 100.00 usec
PLW2 10.00000000 W
PLW12 0.09900300 W
PLW13 0.09900300 W
F2 - Processing parameters
SI 32768
SF 125.7577890 MHz
WDW EM
SSB 0
LB 2.00 Hz
GB 0
PC 1.40
160 150 140 130 120 110 100 90 80 70 60 50 40 30 20 10 ppm

# $^1$H NMR of 3d

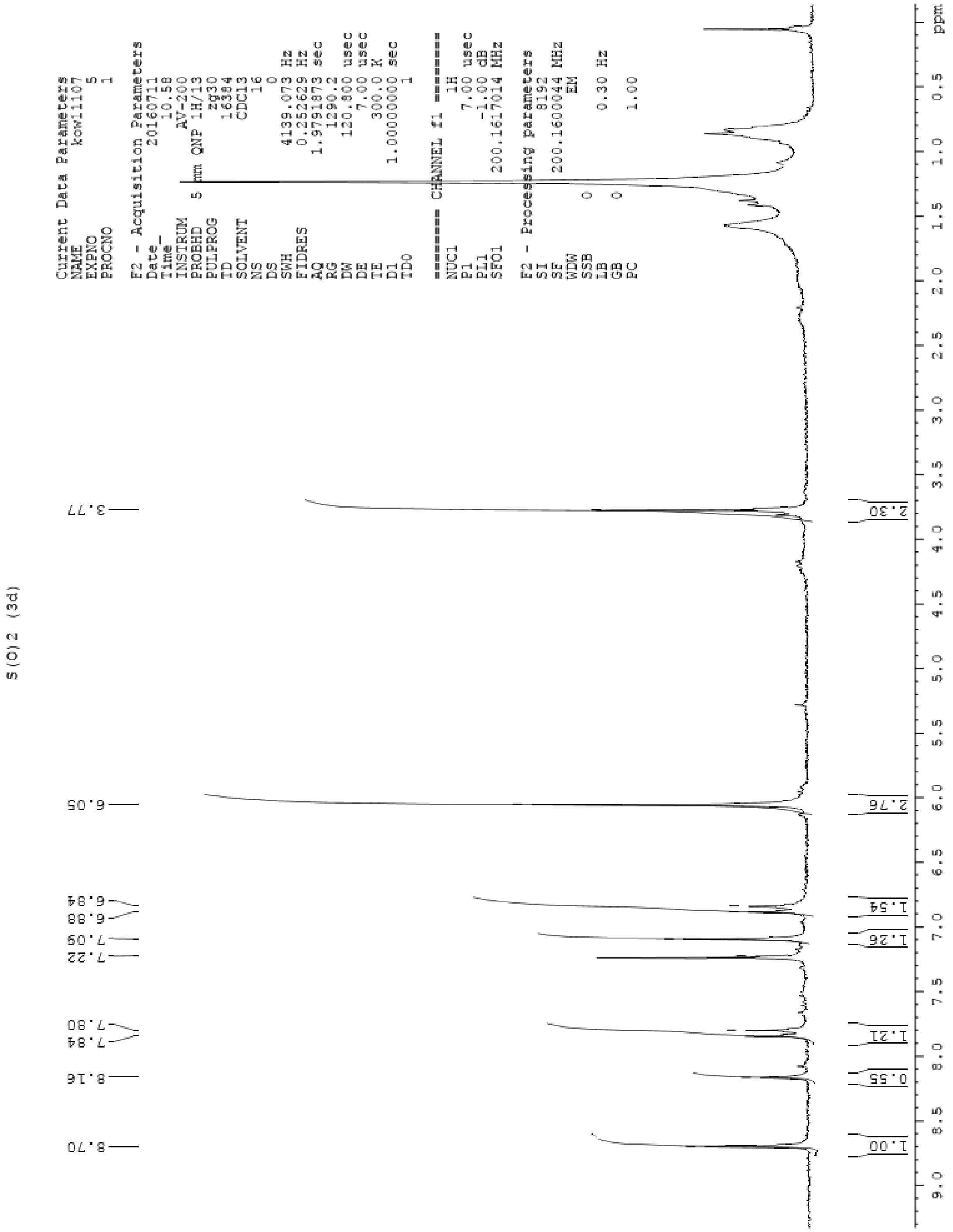
S(O)2 (3d)
Current Data Parameters
NAME kowl1107
EXPNO 5
PROCNO 1
F2 - Acquisition Parameters
Date_ 20160711
Time_ 10.58
INSTRUM AV-200
PROBHD 5 mm QNP 1H/13
PULPROG zg30
TD 16384
SOLVENT CDCl3
NS 16
DS 0
SWH 4139.073 Hz
FIDRES 0.252629 Hz
AQ 1.9791873 sec
RG 1290.2
DW 120.800 usec
DE 7.00 usec
TE 300.0 K
D1 1.00000000 sec
TD0 1
======== CHANNEL f1 ========
NUC1 1H
P1 7.00 usec
PL1 -1.00 dB
SFO1 200.1617014 MHz
F2 - Processing parameters
SI 8192
SF 200.1600044 MHz
WDW EM
SSB 0
LB 0.30 Hz
GB 0
PC 1.00
3.77
6.05
6.84
6.88
7.09
7.22
7.80
7.84
8.16
8.70
2.30
2.76
1.54
1.26
1.21
0.55
1.00
ppm

## $^{13}C$ NMR of 3d

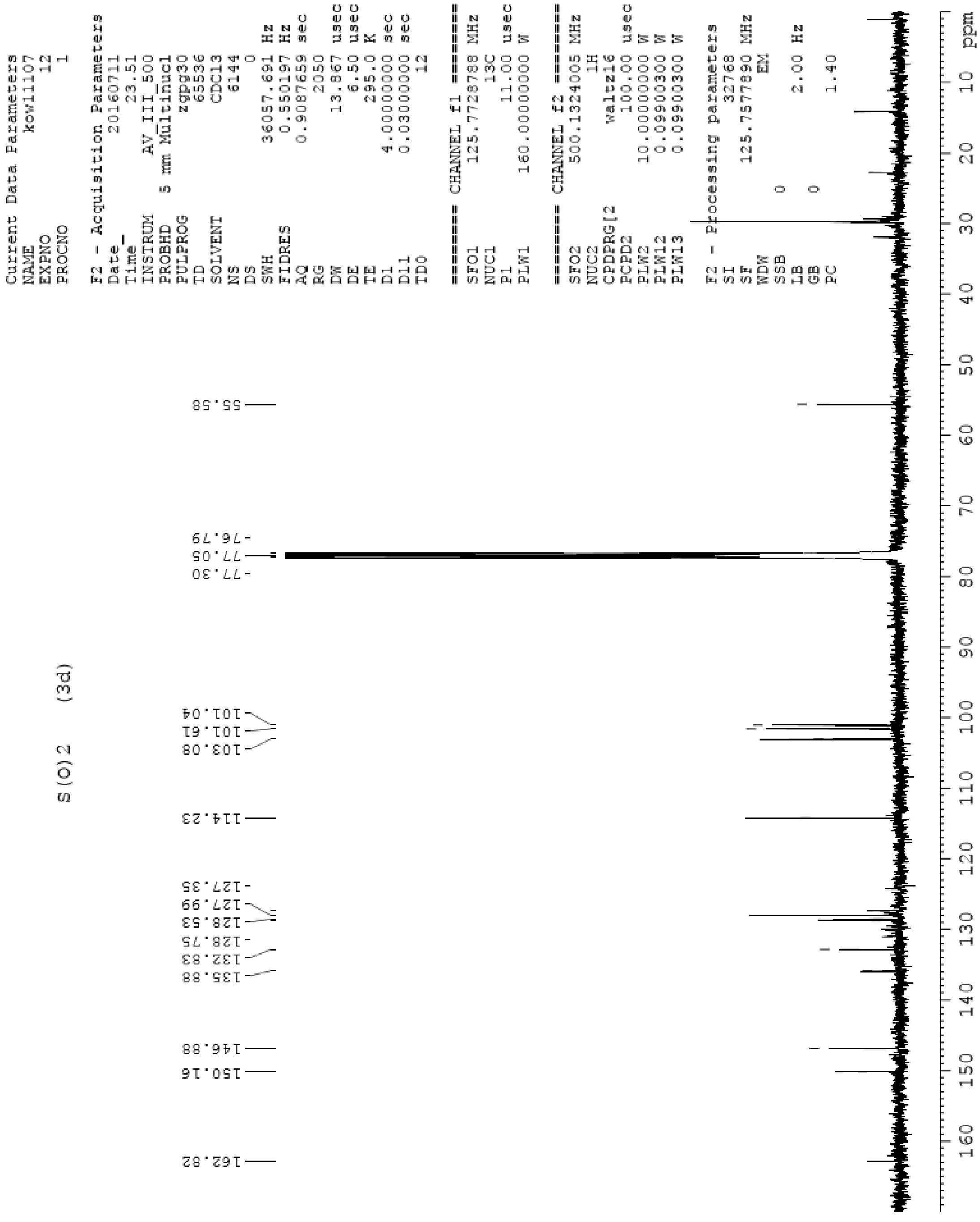

S(O)2 (3d)
162.82
150.16
146.88
135.88
132.83
128.75
128.53
127.99
127.35
114.23
103.08
101.61
101.04
77.30
77.05
76.79
55.58
Current Data Parameters
NAME kow11107
EXPNO 12
PROCNO 1
F2 - Acquisition Parameters
Date_ 20160711
Time_ 23.51
INSTRUM AV_III_500
PROBHD 5 mm Multinucl
PULPROG zgpg30
TD 65536
SOLVENT CDCl3
NS 6144
DS 0
SWH 36057.691 Hz
FIDRES 0.550197 Hz
AQ 0.9087659 sec
RG 2050
DW 13.867 usec
DE 6.50 usec
TE 295.0 K
D1 4.00000000 sec
D11 0.03000000 sec
TD0 12
======== CHANNEL f1 ========
SFO1 125.7728788 MHz
NUC1 13C
P1 11.00 usec
PLW1 160.00000000 W
======== CHANNEL f2 ========
SFO2 500.1324005 MHz
NUC2 1H
CPDPRG[2 waltz16
PCPD2 100.00 usec
PLW2 10.00000000 W
PLW12 0.09900300 W
PLW13 0.09900300 W
F2 - Processing parameters
SI 32768
SF 125.7577890 MHz
WDW EM
SSB 0
LB 2.00 Hz
GB 0
PC 1.40
160 150 140 130 120 110 100 90 80 70 60 50 40 30 20 10 ppm

## Optical properties

**Figure S2.** Chemical structures of sulfides **1a-d**, sulfoxides **2a-d** and sulfones **3c**,**d** used in measurements.

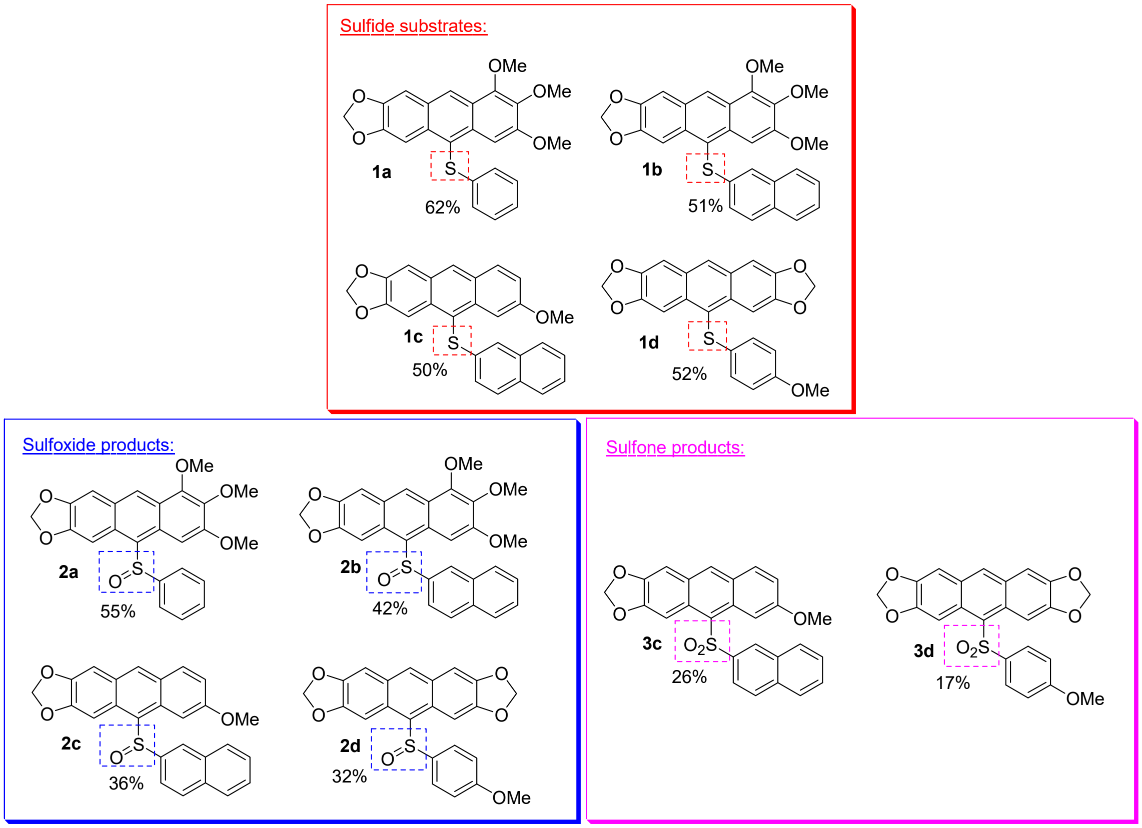

**Figure S3.** Absorption spectra for sulfides **1a-d**, sulfoxides **2a-d** and sulfones **3c**,**d** in ethanol solutions.

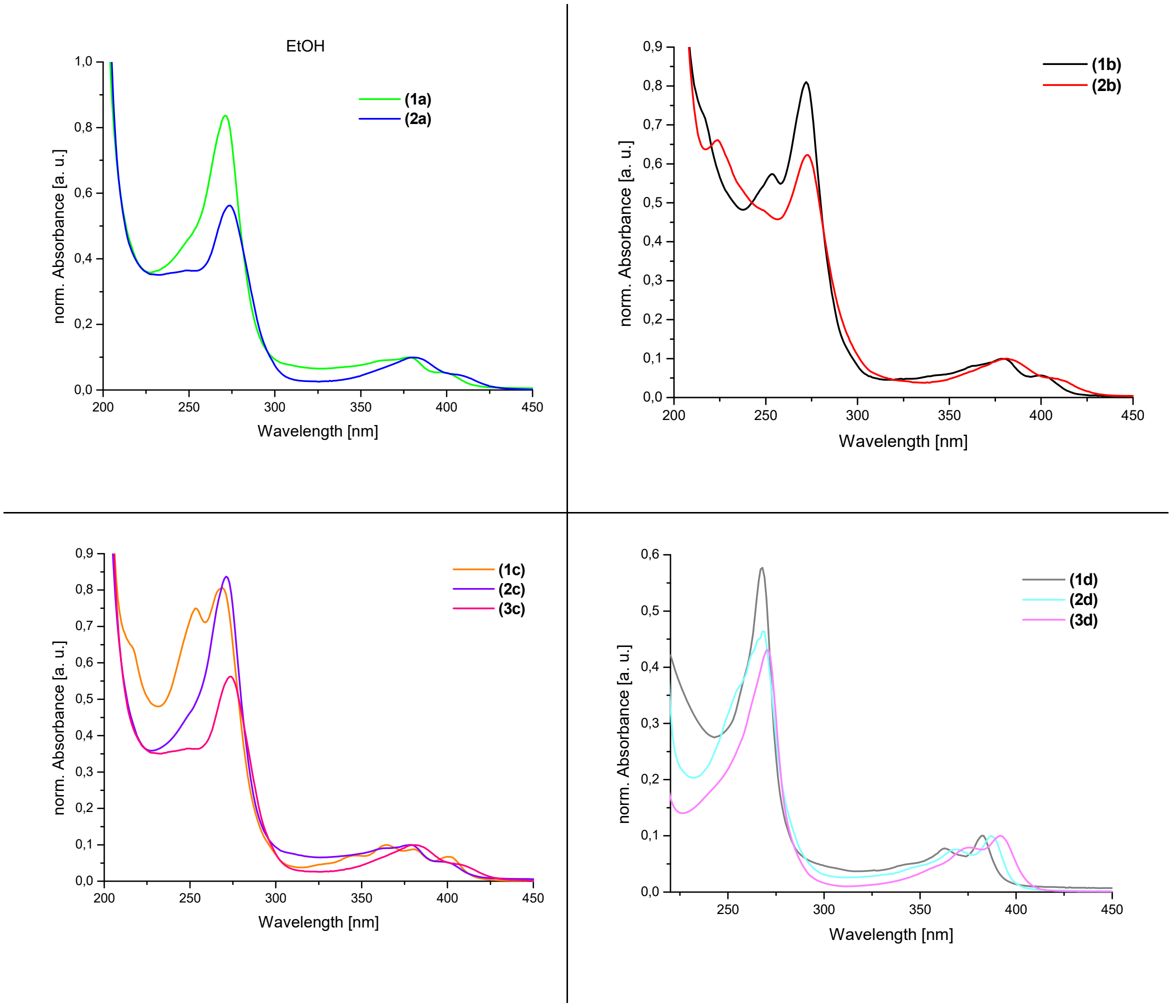

# Time-correlated single-photon counting (TCSPC) measurements

**Figure S4.** Time-correlated single-photon counting (TCSPC) measurements of photoluminescence lifetimes in ethanol solutions.

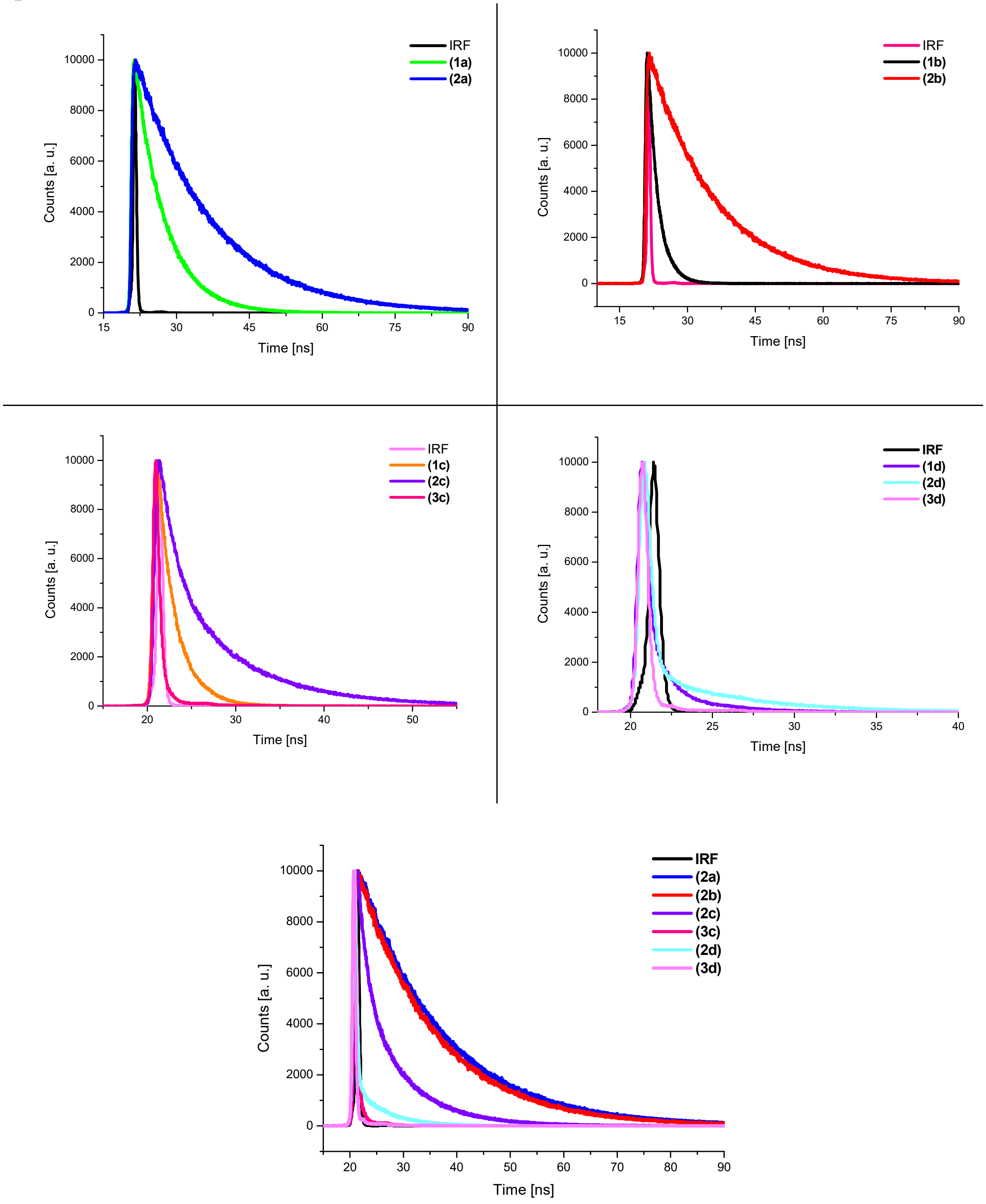

**Figure S5.** UV/Vis absorption and photoluminescence spectra for sulfides **1a-d**, sulfoxides **2a-d** and sulfones **3c**,**d** in toluene solutions.

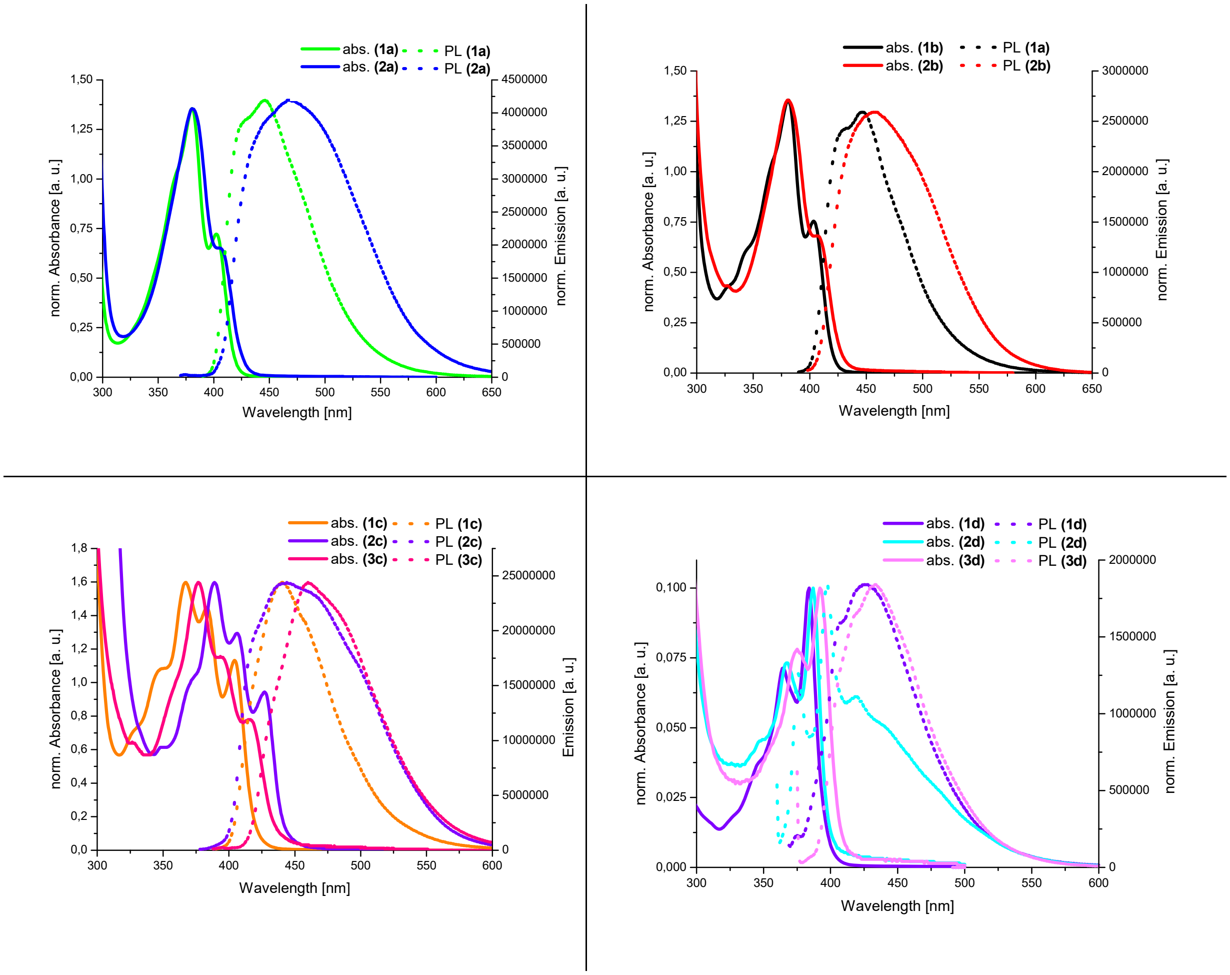

# Time-correlated single-photon counting (TCSPC) measurements

**Figure S6.** Time-correlated single-photon counting (TCSPC) measurements of photoluminescence lifetimes in toluene solutions.

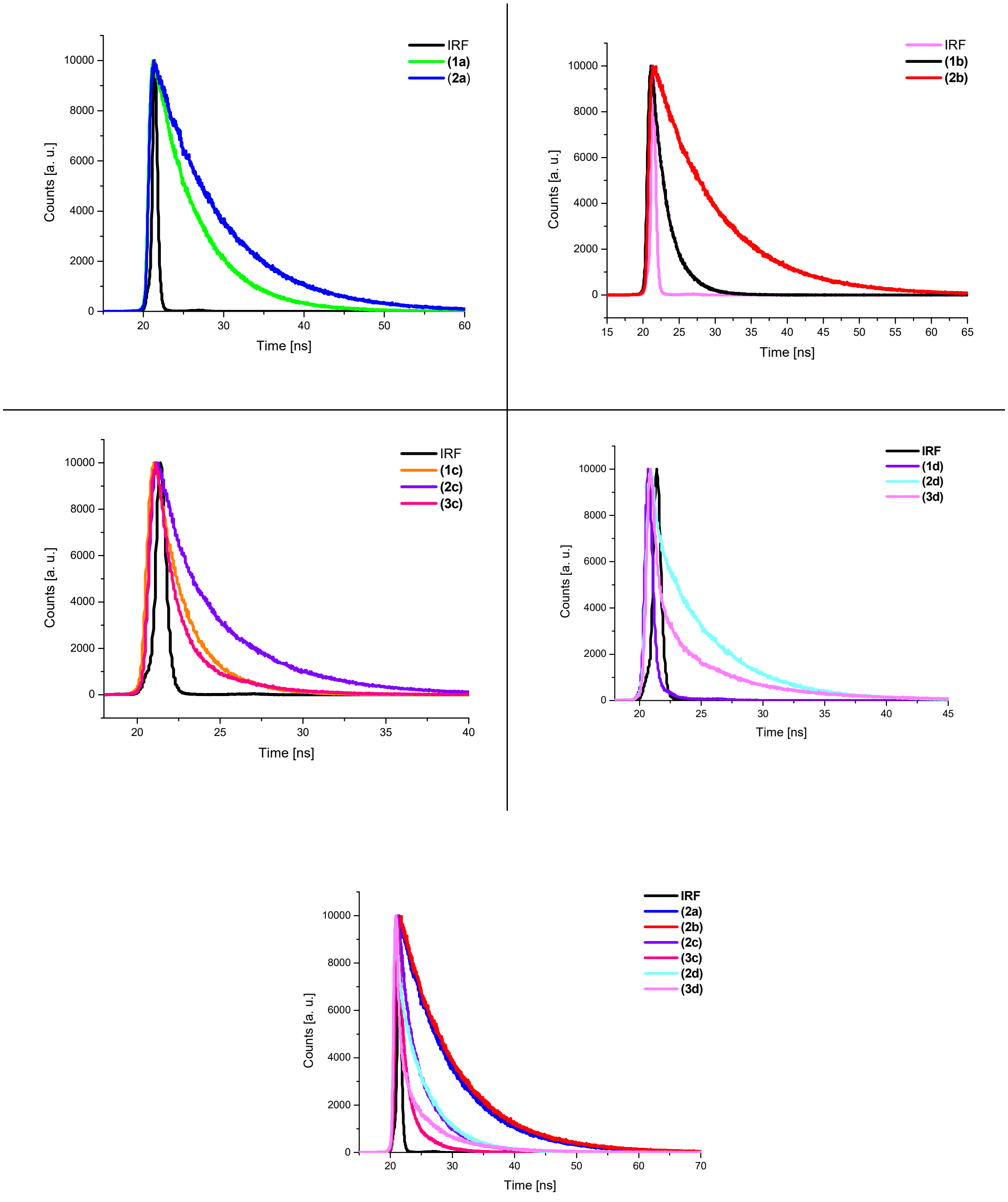

**Figure S7.** Absorption spectra recorded for sulfides **1a-d**, sulfoxides **2a-d** and sulfones **3c**,**d** in solid state (thin-films).

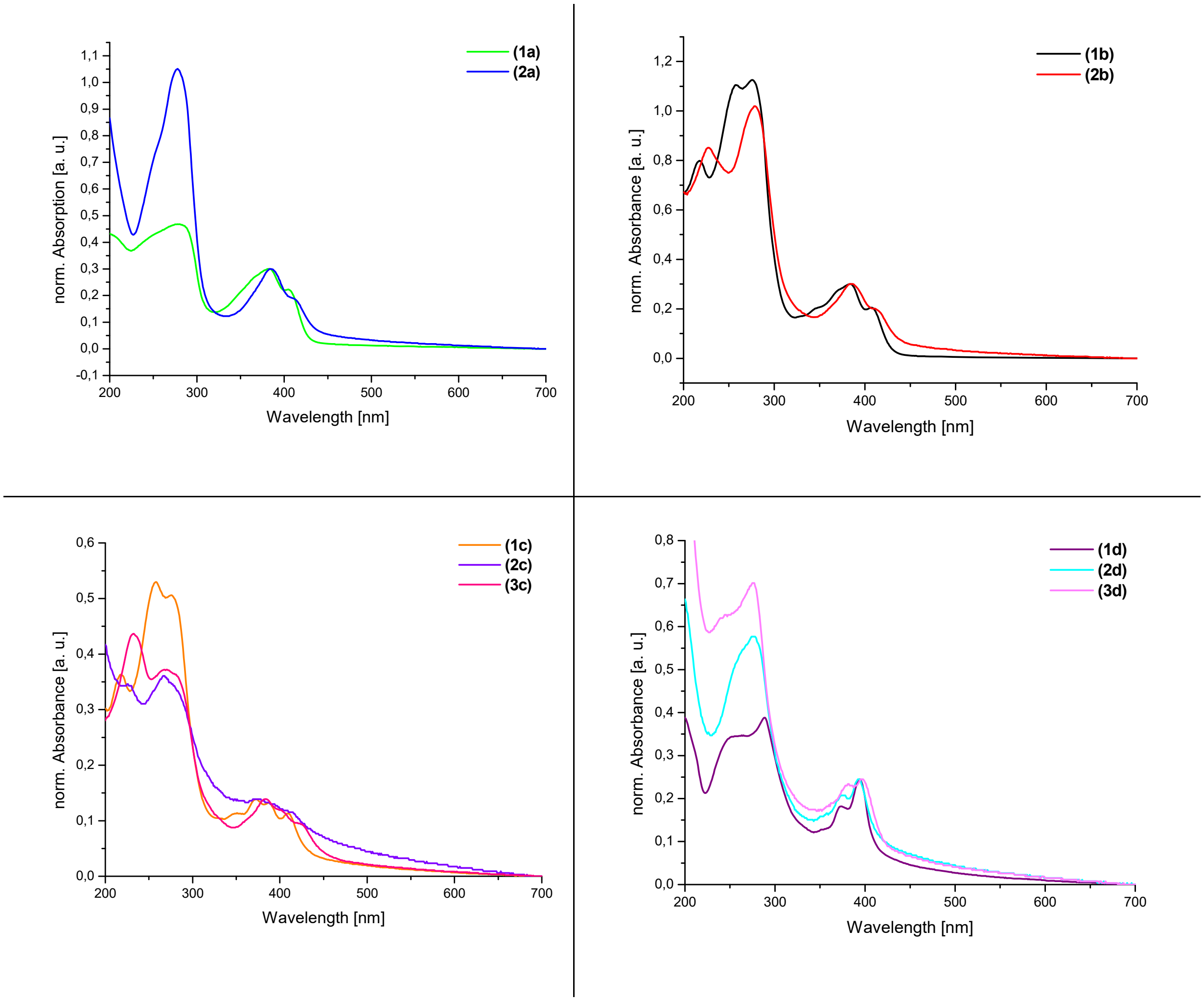

## Photostability and photooxidation stability in ethanol solutions.

The photodegradation was investigated by monitoring the absorbance decay of $10^{-5}$ M ethanolic solutions in quartz cuvettes stored in the dark at room temperature, under ambient atmosphere and Ar atmosphere, and exposed to UVP-Hg-Pen-ray lamp (254 nm, 16.33 mW/cm$^2$ at distance 1 cm) and fluorescent lamp VL-6.LC (6W) (365 nm, 27.4 mW/cm$^2$ at distance 1 cm).

**Figure S8.** Photostability and photooxidation stability of compounds: **1a-d**, **2a-d** and **3c**,**d** in ethanol solutions.

**1a** in ethanol UV-Vis 254 nm, $O_2$, c = $10^{-5}$

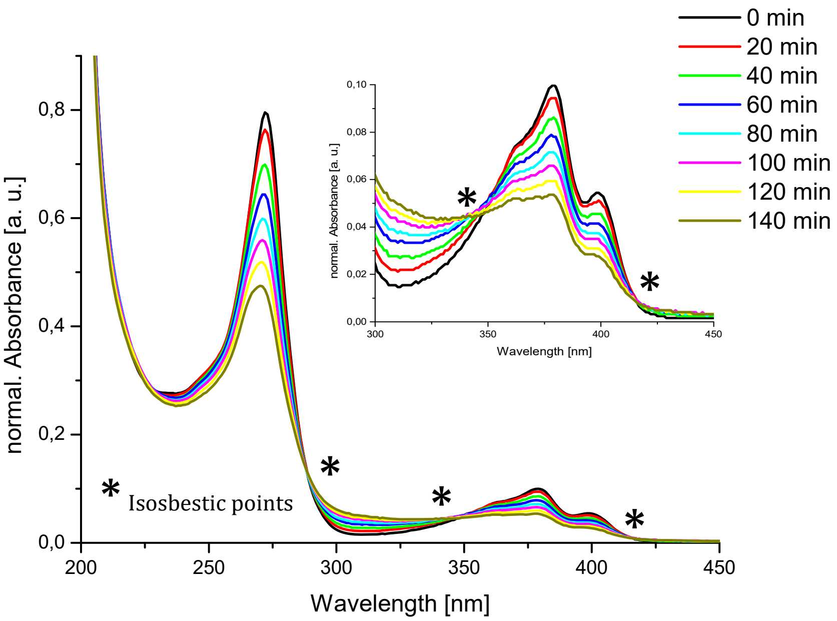

<u>**1a** in ethanol UV-Vis 254 nm, Ar, c = $10^{-5}$</u>

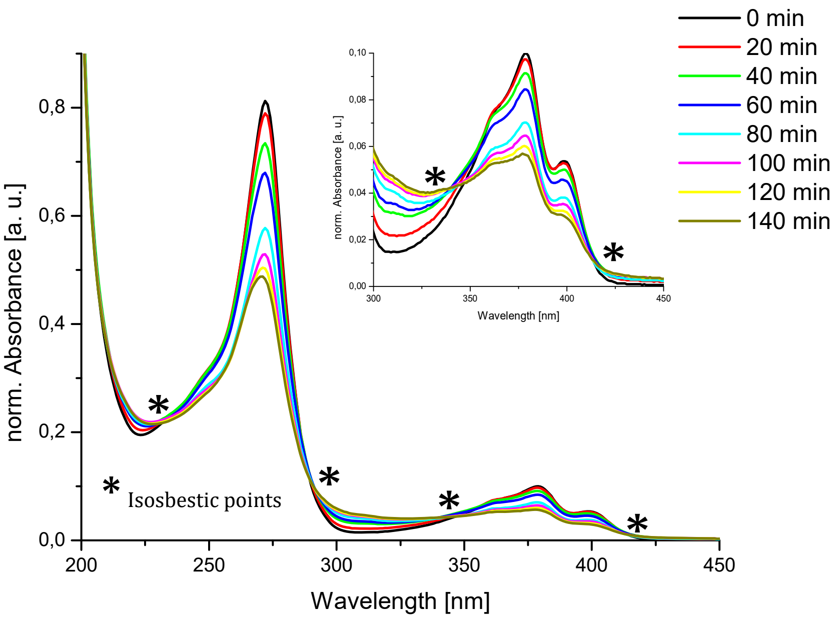

## **1a** in ethanol UV-Vis 365 nm, $O_2$, $c = 10^{-5}$

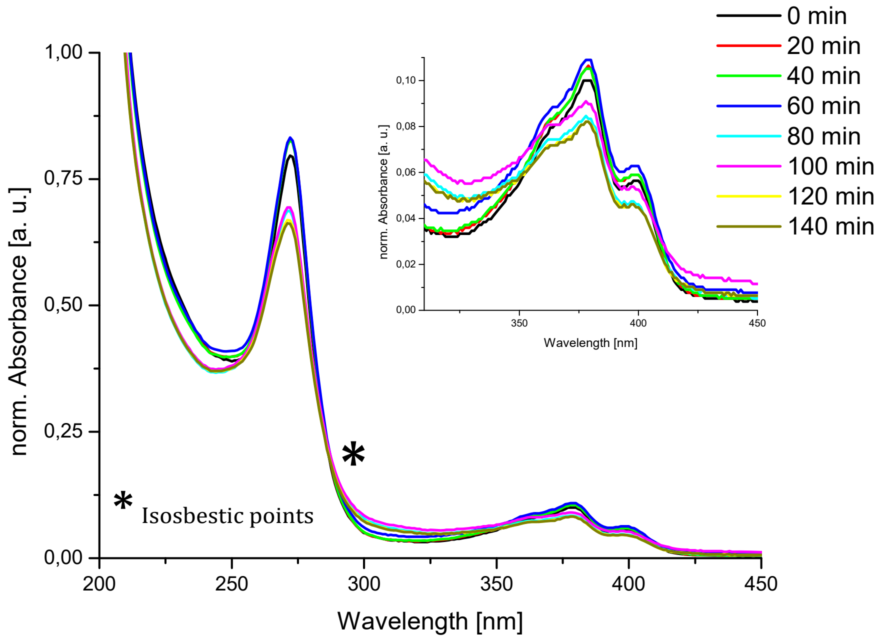


**1a**

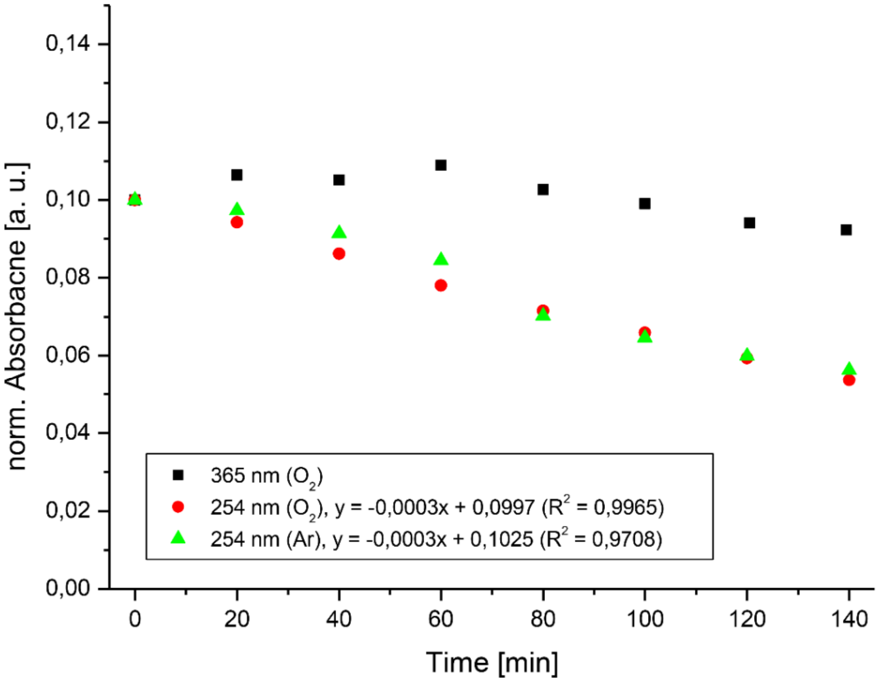

**1b** in ethanol UV-Vis 254 nm, $O_2$, c = $10^{-5}$

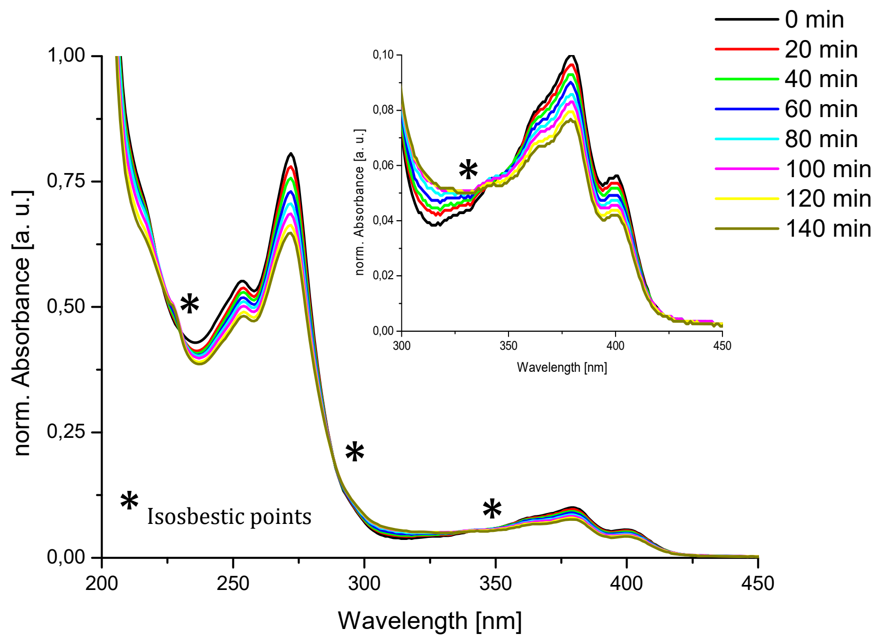


**1b** in ethanol UV-Vis 254 nm, Ar, c = $10^{-5}$

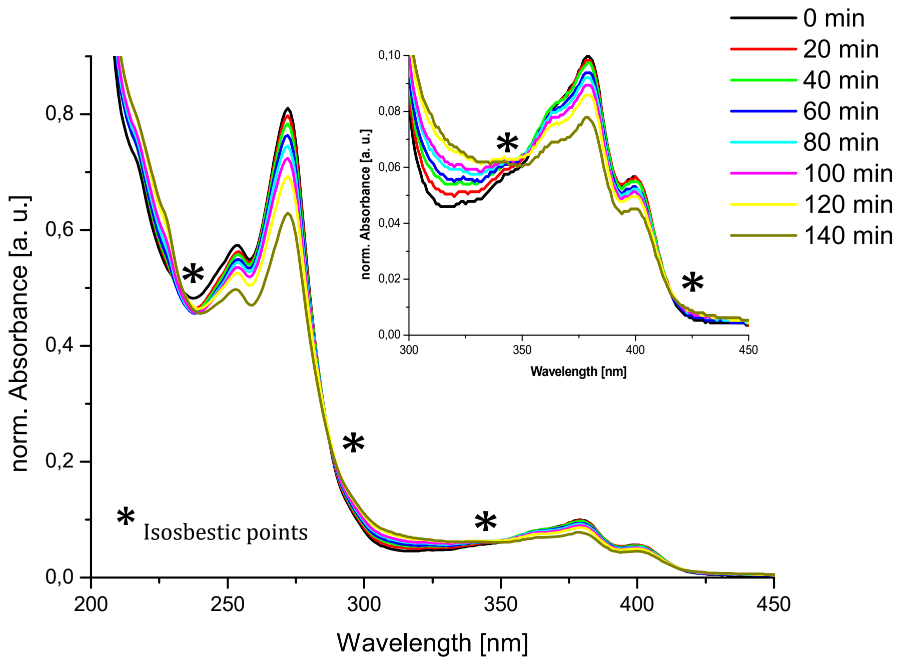

**1b** in ethanol UV-Vis 365 nm, $O_2$, c = $10^{-5}$

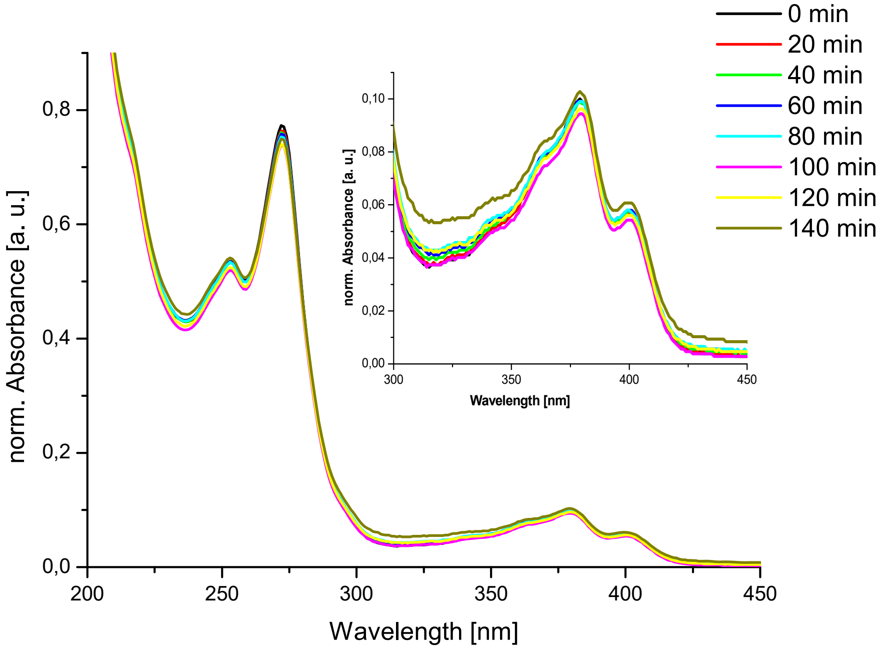


**1b**

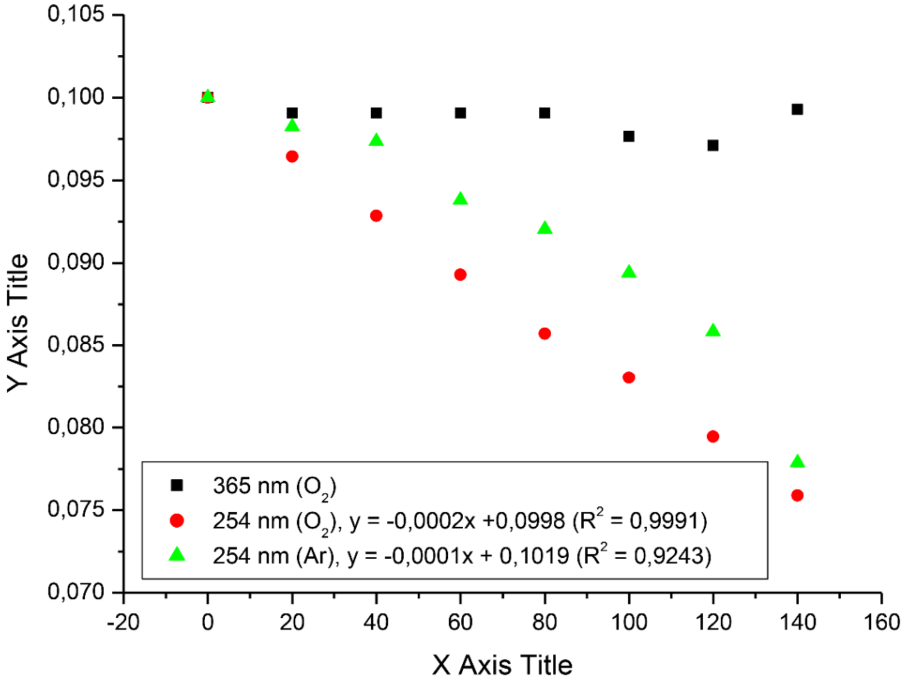

**1c** in ethanol UV-Vis 254 nm, $O_2$, c = $10^{-5}$

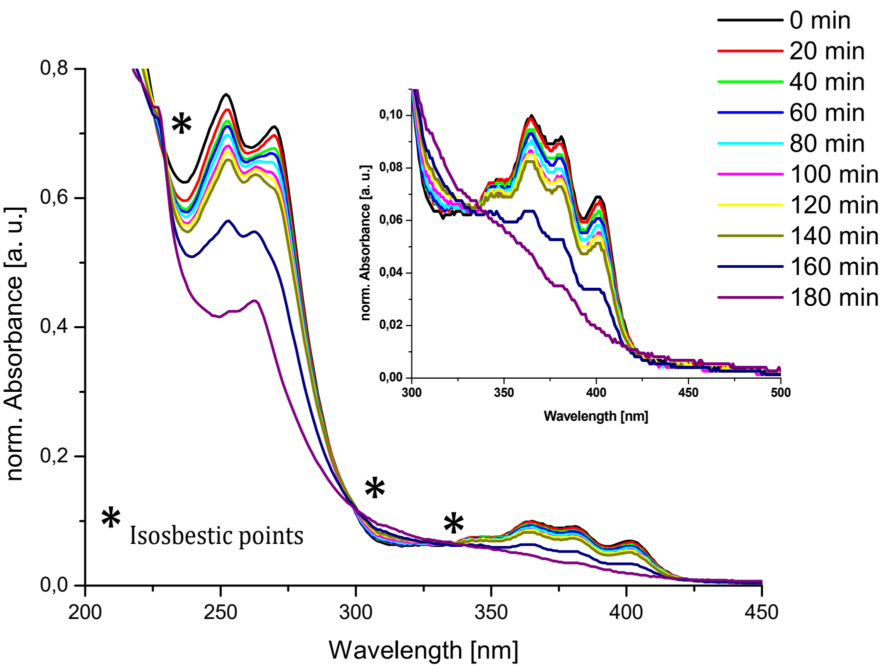


**1c** in ethanol UV-Vis 254 nm, Ar, c = $10^{-5}$

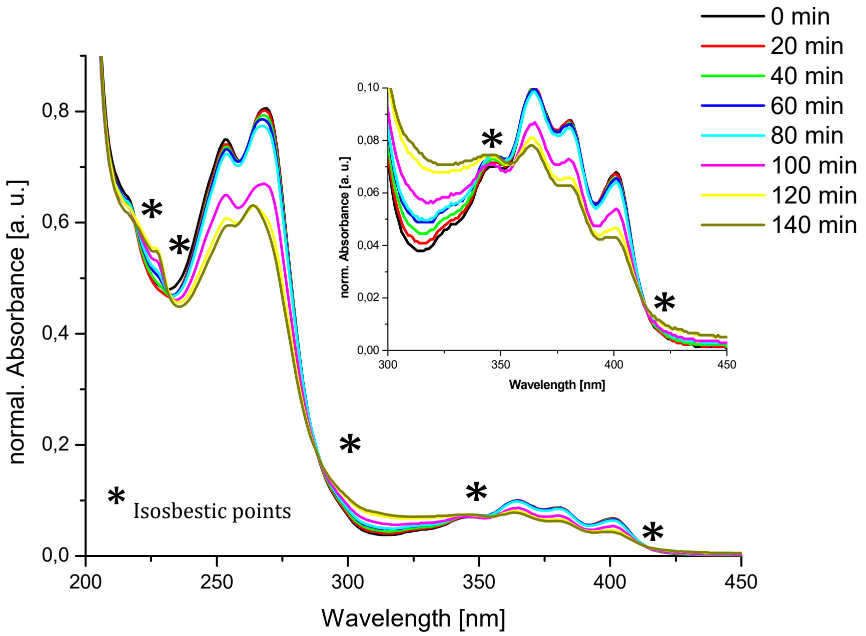

**1c** in ethanol UV-Vis 365 nm, $O_2$, $c = 10^{-5}$

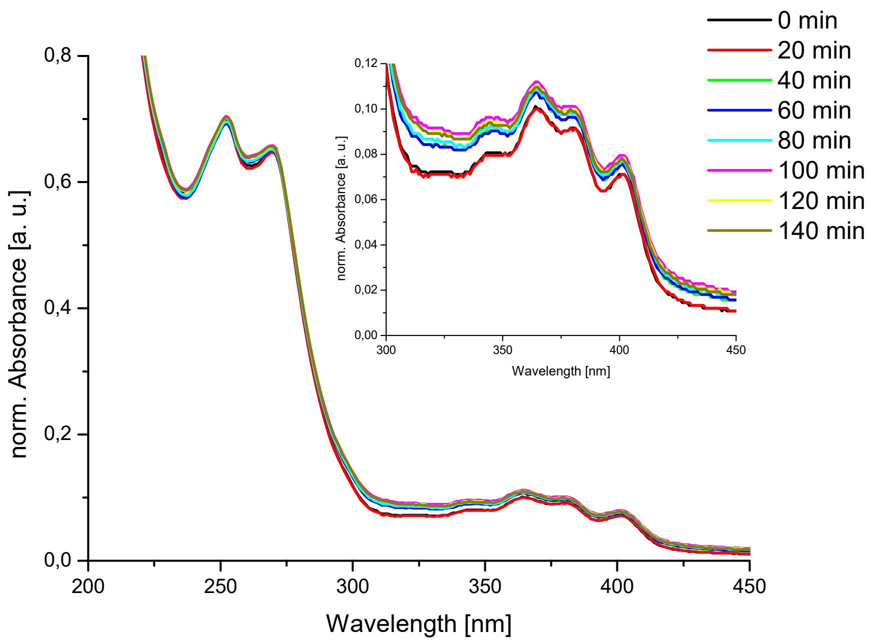


**1c**

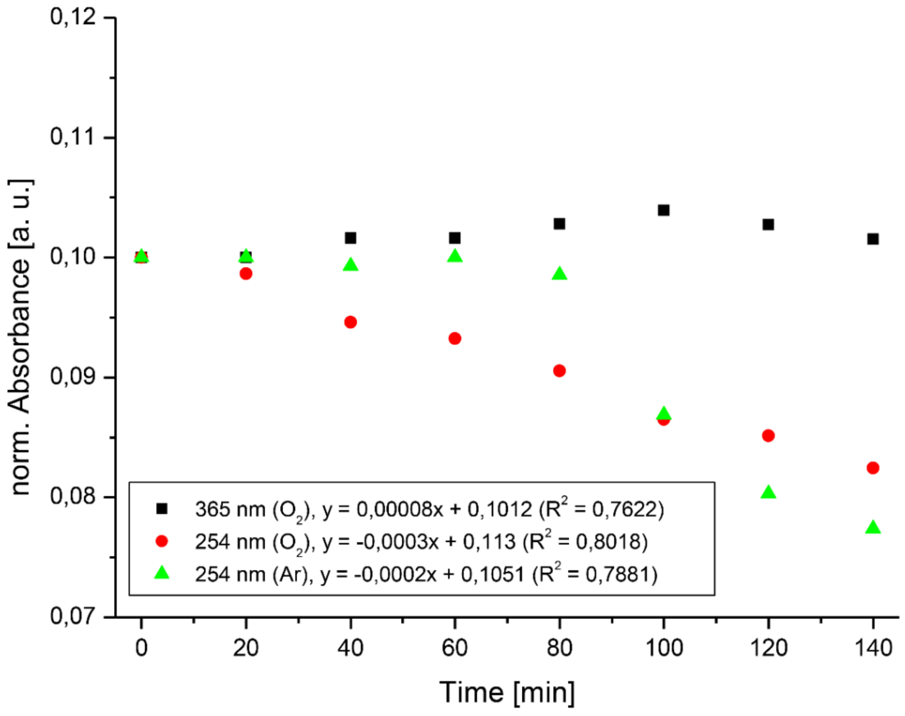

**1d** in ethanol UV-Vis 254 nm, $O_2$, c = $10^{-5}$

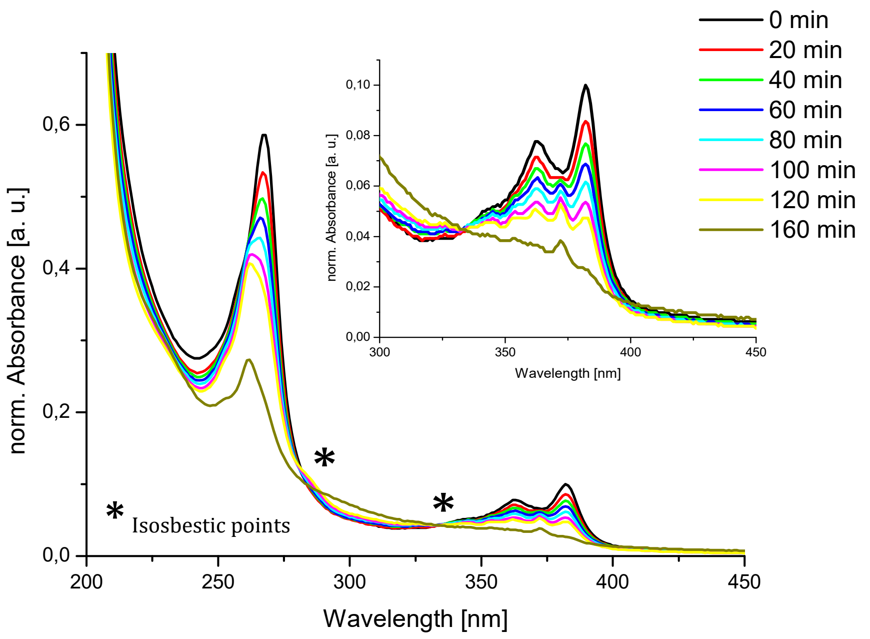


**1d** in ethanol UV-Vis 365 nm, $O_2$, c = $10^{-5}$

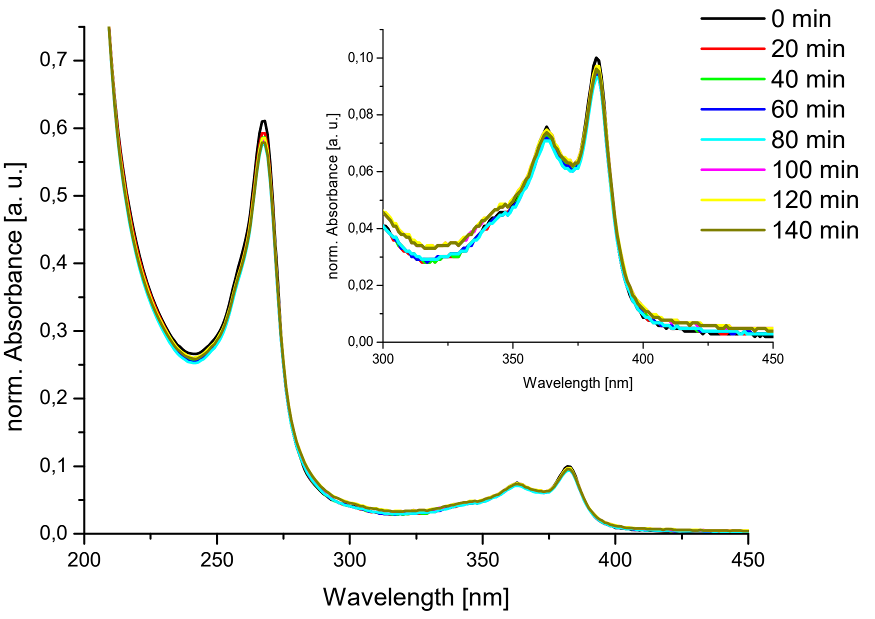

**1d**

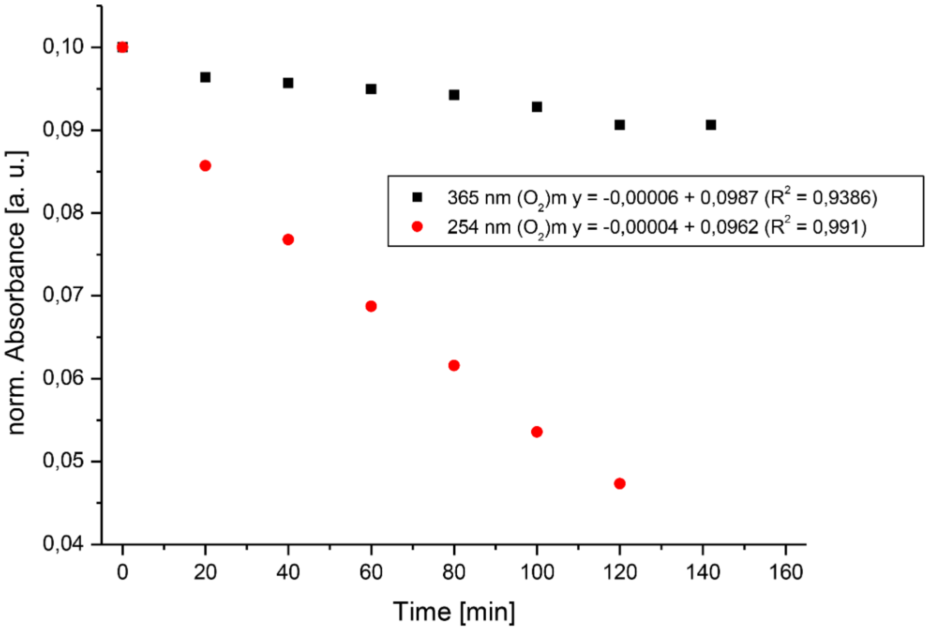

norm. Absorbance [a. u.]
0,10
0,09
0,08
0,07
0,06
0,05
0,04
0
20
40
60
80
100
120
140
160
Time [min]
365 nm (O2)m y = -0,00006 + 0,0987 (R2 = 0,9386)
254 nm (O2)m y = -0,00004 + 0,0962 (R2 = 0,991)

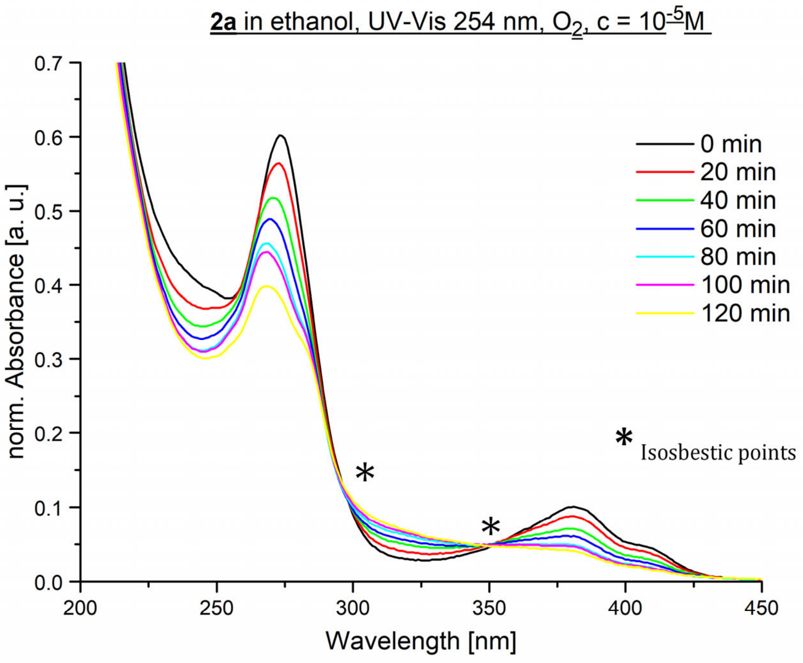
2a in ethanol, UV-Vis 254 nm, $O_2$, c = $10^{-5}$M
norm. Absorbance [a. u.]
Wavelength [nm]
0 min
20 min
40 min
60 min
80 min
100 min
120 min
* Isosbestic points

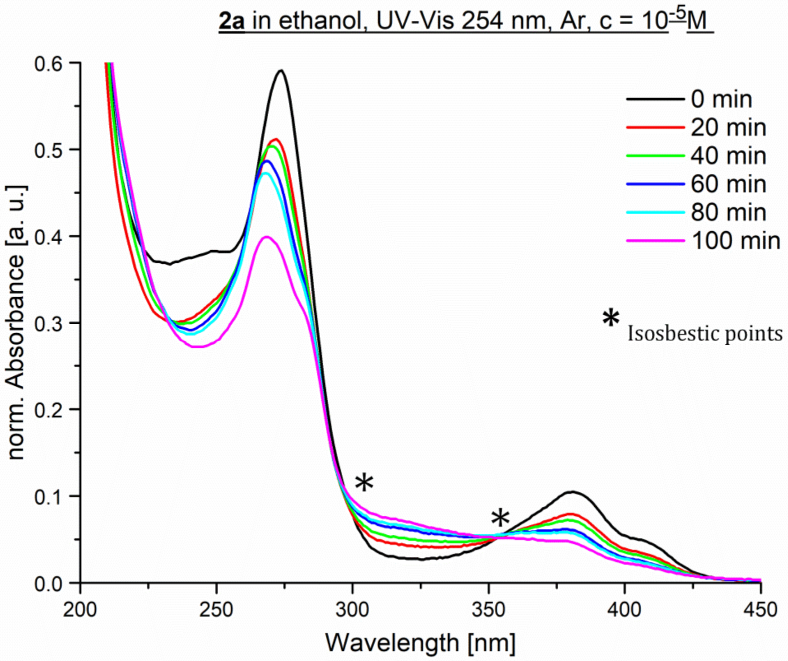
2a in ethanol, UV-Vis 254 nm, Ar, c = $10^{-5}$M
norm. Absorbance [a. u.]
Wavelength [nm]
0 min
20 min
40 min
60 min
80 min
100 min
* Isosbestic points

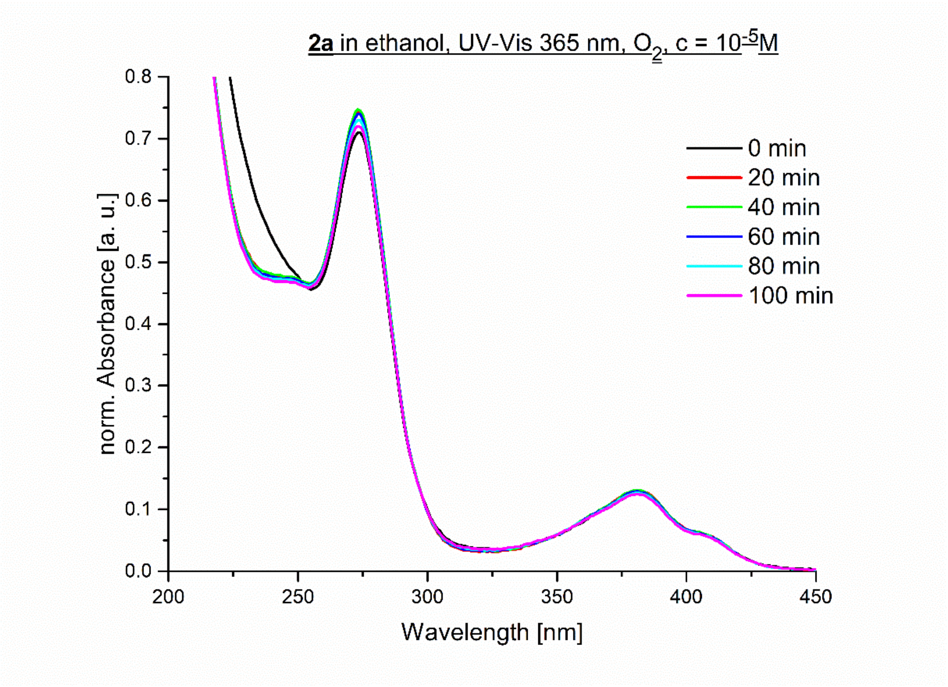

2a in ethanol, UV-Vis 365 nm, O2, c = 10-5M
norm. Absorbance [a. u.]
Wavelength [nm]
0 min
20 min
40 min
60 min
80 min
100 min
0.0
0.1
0.2
0.3
0.4
0.5
0.6
0.7
0.8
200
250
300
350
400
450


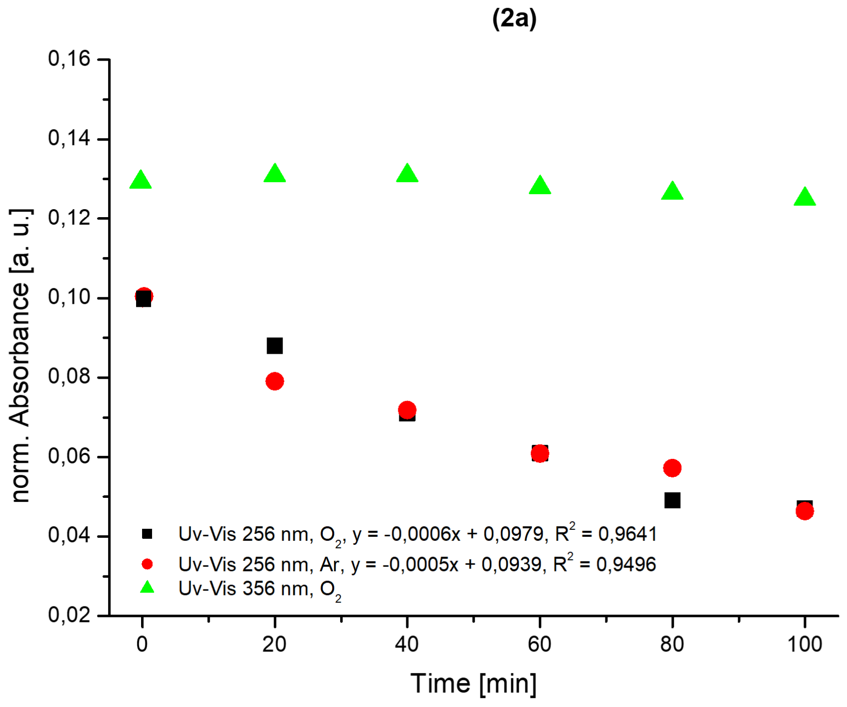

(2a)
norm. Absorbance [a. u.]
Time [min]
Uv-Vis 256 nm, O2, y = -0,0006x + 0,0979, R2 = 0,9641
Uv-Vis 256 nm, Ar, y = -0,0005x + 0,0939, R2 = 0,9496
Uv-Vis 356 nm, O2
0,02
0,04
0,06
0,08
0,10
0,12
0,14
0,16
0
20
40
60
80
100

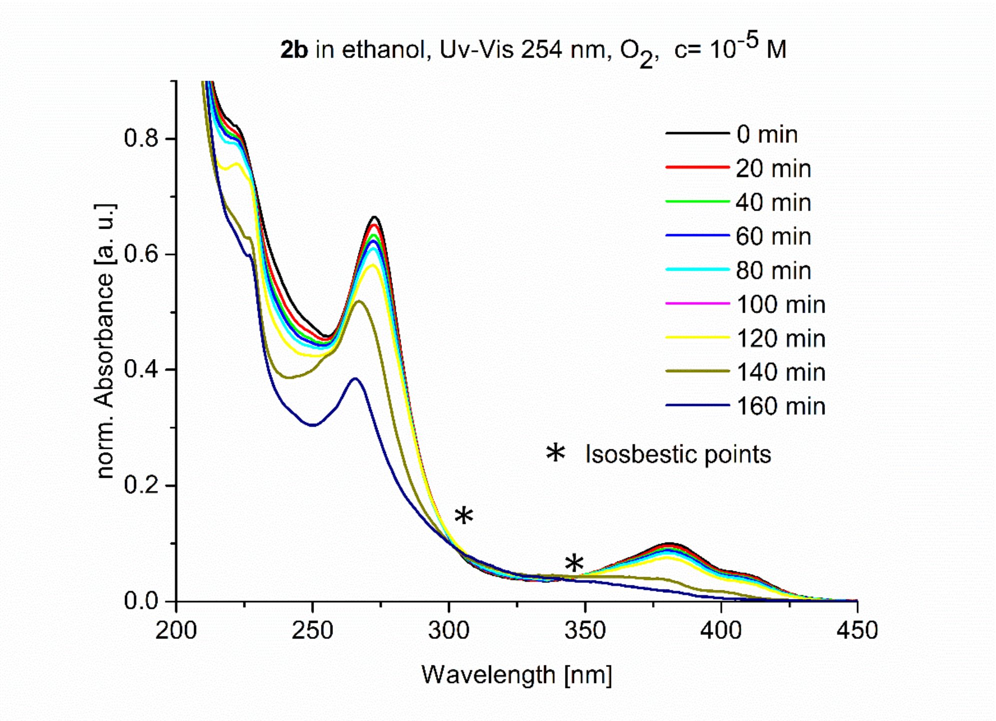
**2b** in ethanol, Uv-Vis 254 nm, O2, c= 10-5 M
norm. Absorbance [a. u.]
Wavelength [nm]
0 min
20 min
40 min
60 min
80 min
100 min
120 min
140 min
160 min
* Isosbestic points

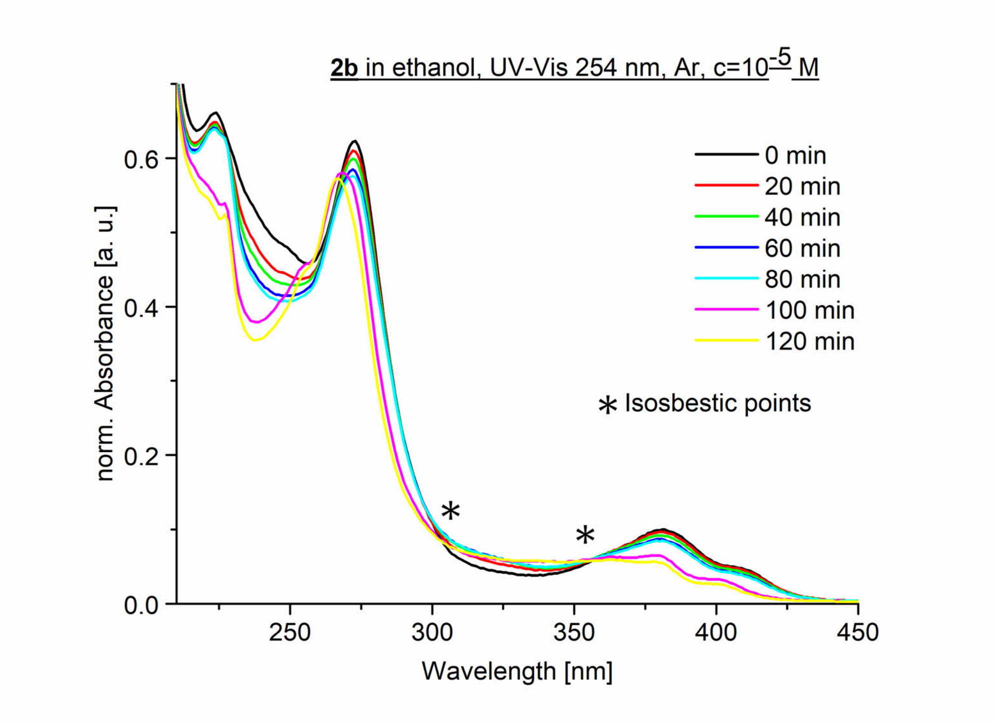
2b in ethanol, UV-Vis 254 nm, Ar, c=10-5 M
norm. Absorbance [a. u.]
Wavelength [nm]
0 min
20 min
40 min
60 min
80 min
100 min
120 min
* Isosbestic points

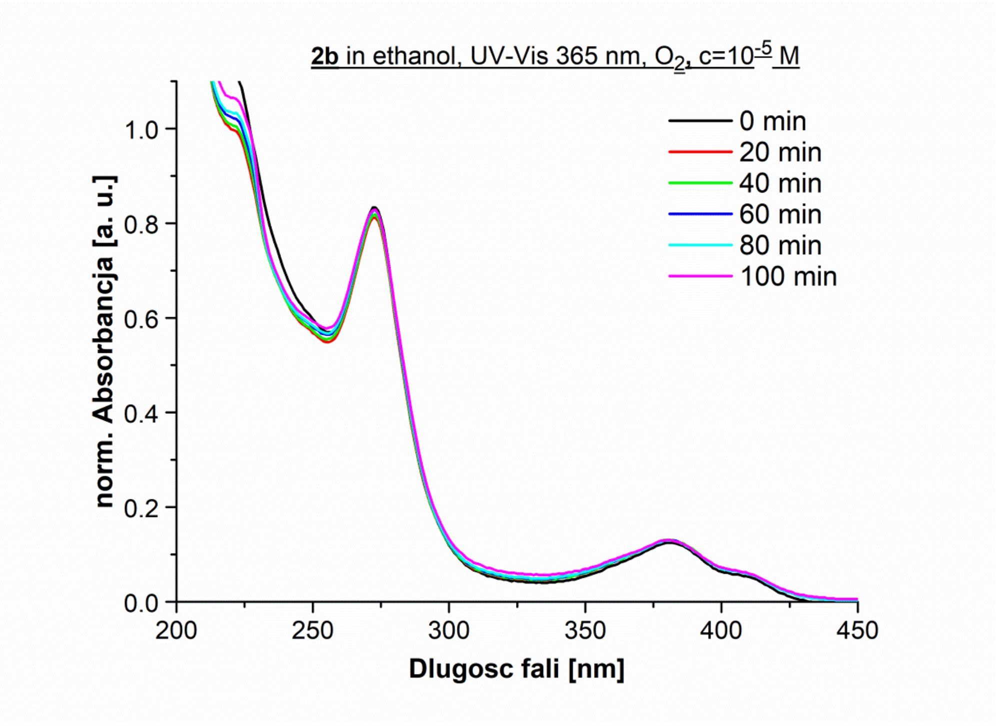
2b in ethanol, UV-Vis 365 nm, O2, c=10-5 M
norm. Absorbancja [a. u.]
Dlugosc fali [nm]
0 min
20 min
40 min
60 min
80 min
100 min
0.0
0.2
0.4
0.6
0.8
1.0
200
250
300
350
400
450

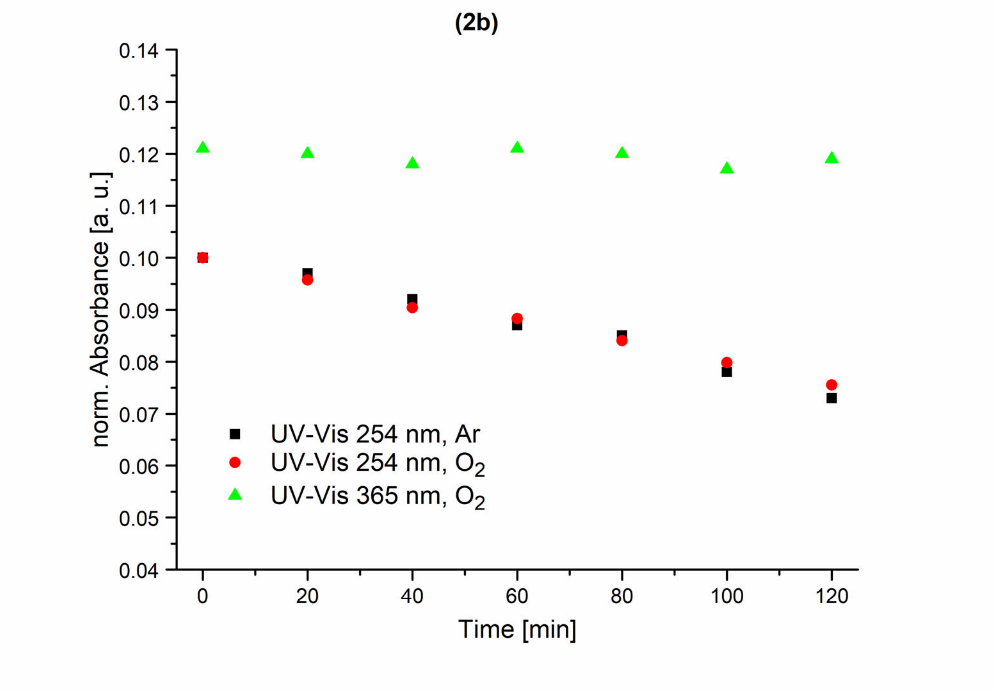
(2b)
norm. Absorbance [a. u.]
Time [min]
UV-Vis 254 nm, Ar
UV-Vis 254 nm, O2
UV-Vis 365 nm, O2
0.04
0.05
0.06
0.07
0.08
0.09
0.10
0.11
0.12
0.13
0.14
0
20
40
60
80
100
120

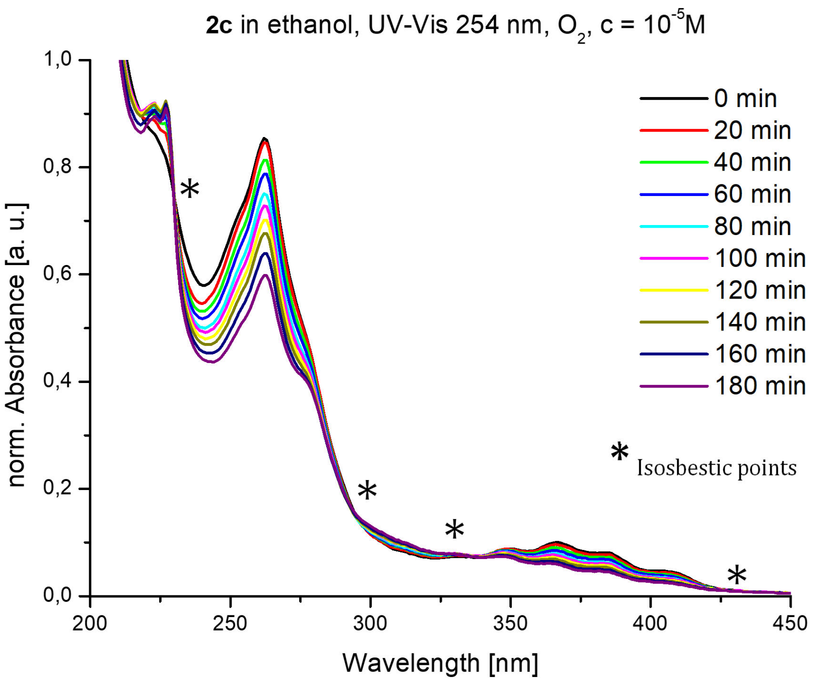
2c in ethanol, UV-Vis 254 nm, O2, c = 10-5M
norm. Absorbance [a. u.]
Wavelength [nm]
0 min
20 min
40 min
60 min
80 min
100 min
120 min
140 min
160 min
180 min
* Isosbestic points

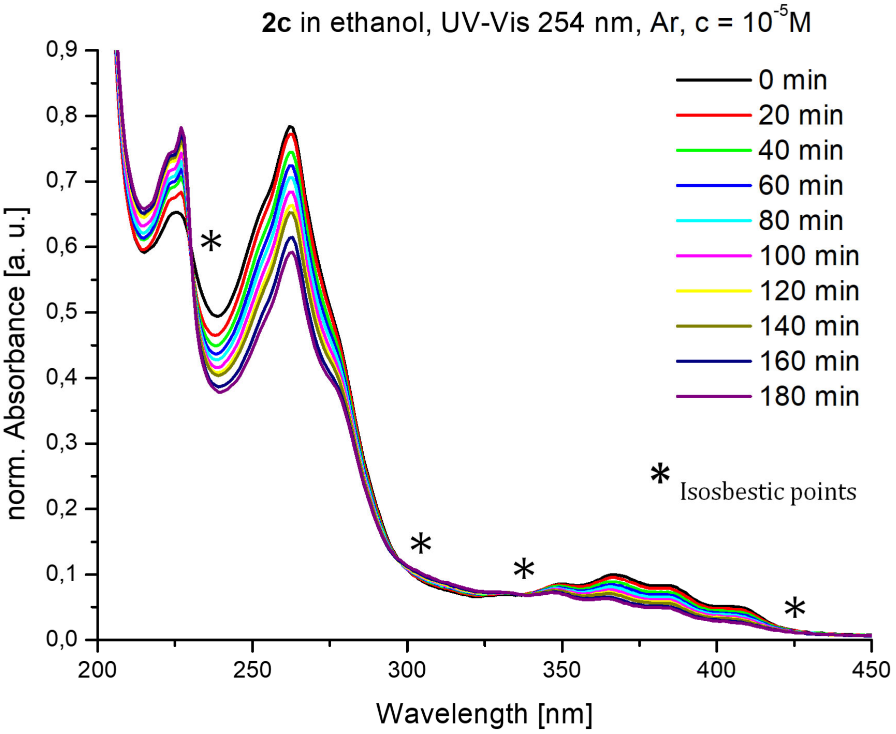
2c in ethanol, UV-Vis 254 nm, Ar, c = 10-5M
norm. Absorbance [a. u.]
Wavelength [nm]
0 min
20 min
40 min
60 min
80 min
100 min
120 min
140 min
160 min
180 min
* Isosbestic points

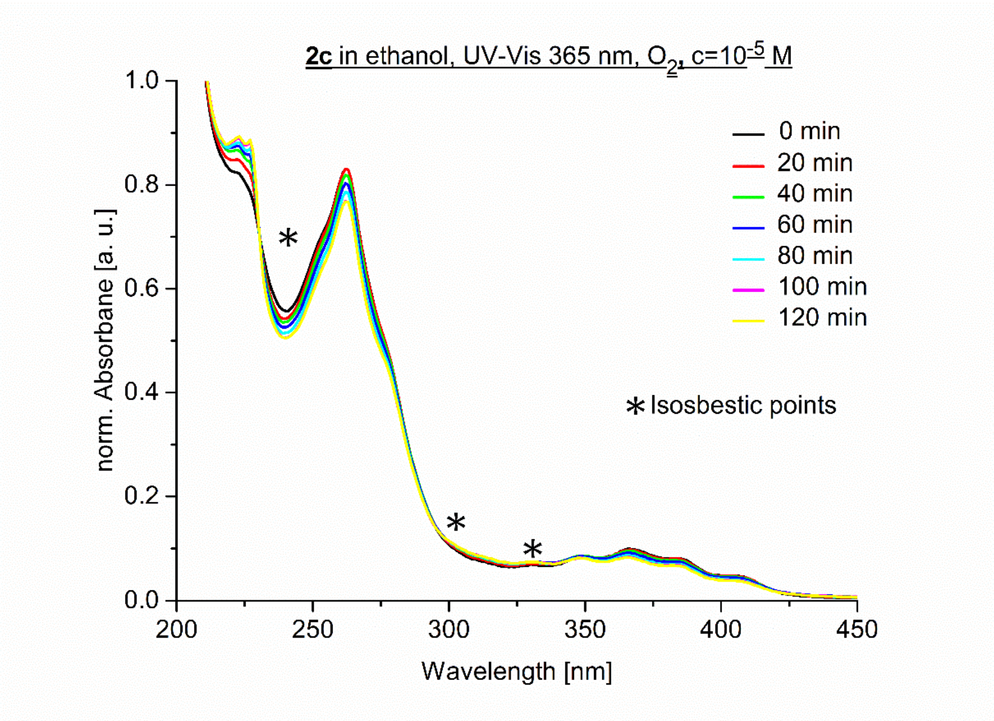
2c in ethanol, UV-Vis 365 nm, O2, c=10-5 M
0 min
20 min
40 min
60 min
80 min
100 min
120 min
* Isosbestic points
norm. Absorbane [a. u.]
Wavelength [nm]
1.0
0.8
0.6
0.4
0.2
0.0
200
250
300
350
400
450

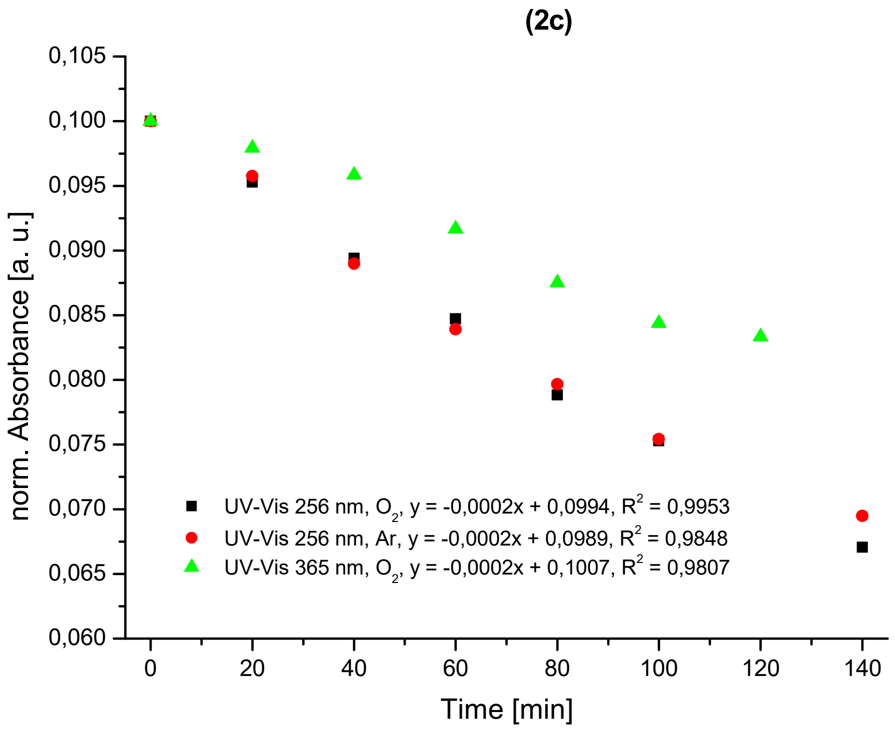
(2c)
UV-Vis 256 nm, O2, y = -0,0002x + 0,0994, R2 = 0,9953
UV-Vis 256 nm, Ar, y = -0,0002x + 0,0989, R2 = 0,9848
UV-Vis 365 nm, O2, y = -0,0002x + 0,1007, R2 = 0,9807
norm. Absorbance [a. u.]
Time [min]
0,105
0,100
0,095
0,090
0,085
0,080
0,075
0,070
0,065
0,060
0
20
40
60
80
100
120
140

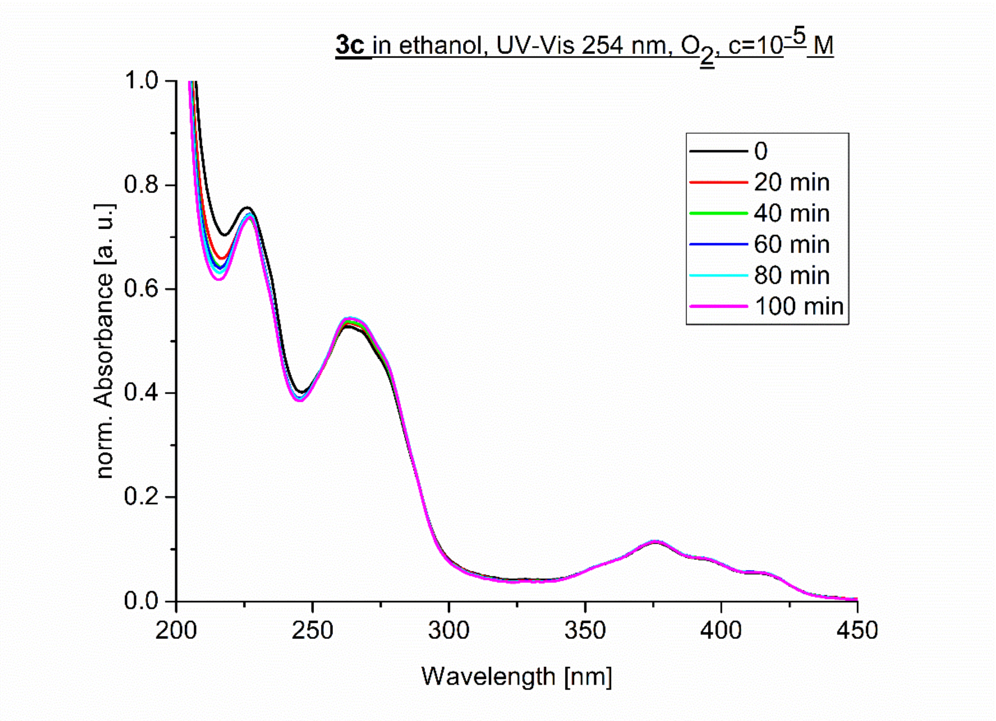
3c in ethanol, UV-Vis 254 nm, O2, c=10-5 M
norm. Absorbance [a. u.]
Wavelength [nm]
0
20 min
40 min
60 min
80 min
100 min
1.0
0.8
0.6
0.4
0.2
0.0
200
250
300
350
400
450

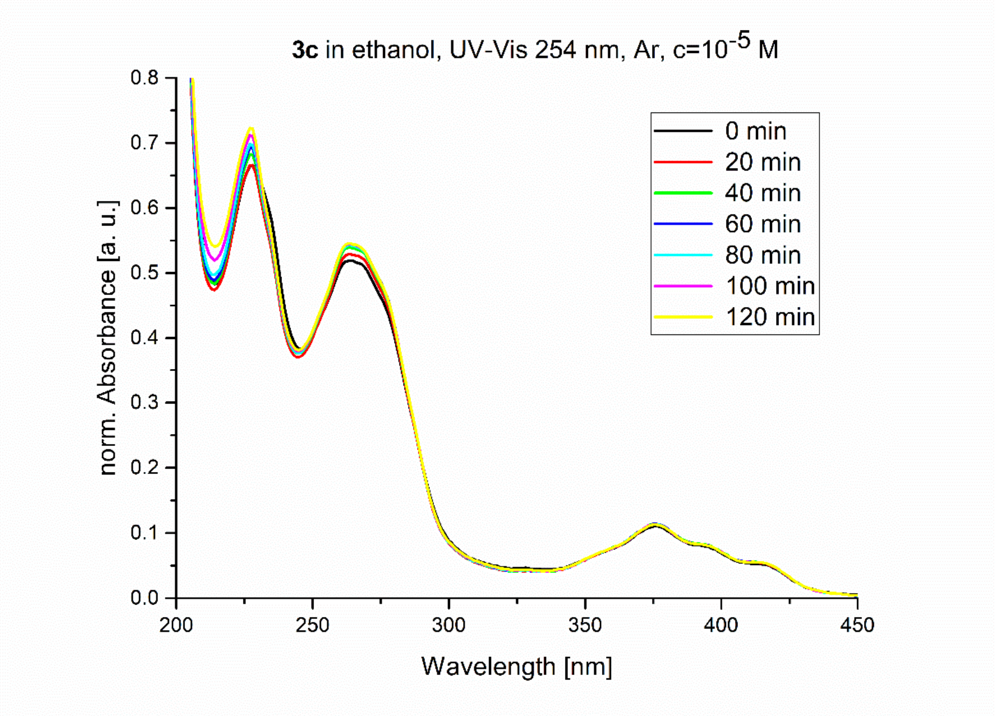
3c in ethanol, UV-Vis 254 nm, Ar, c=10-5 M
norm. Absorbance [a. u.]
Wavelength [nm]
0 min
20 min
40 min
60 min
80 min
100 min
120 min
0.8
0.7
0.6
0.5
0.4
0.3
0.2
0.1
0.0
200
250
300
350
400
450

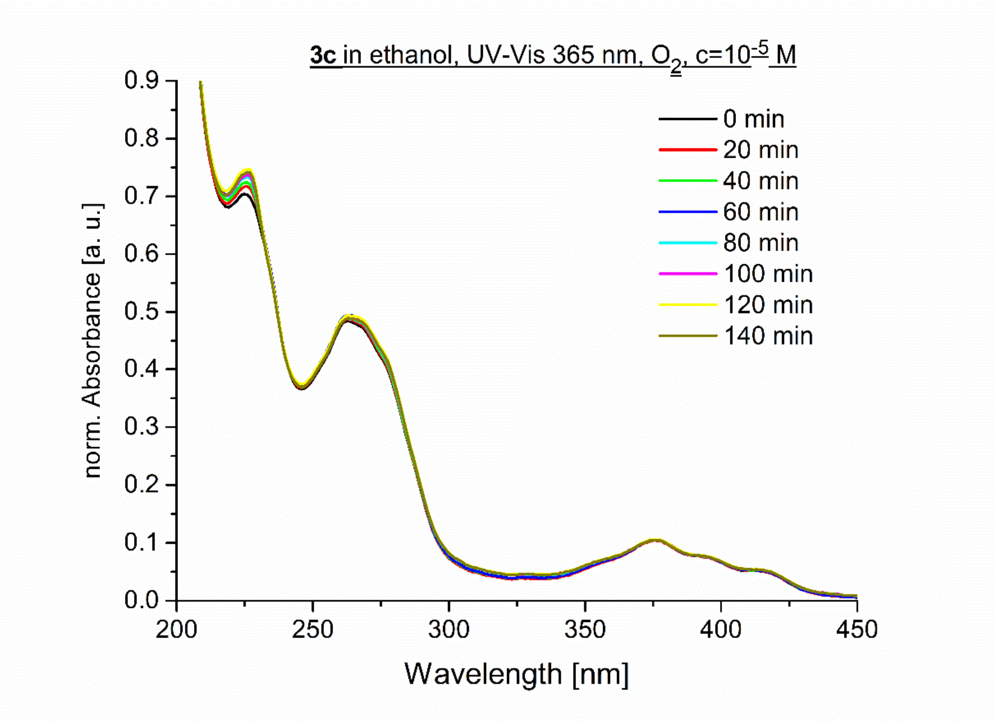
3c in ethanol, UV-Vis 365 nm, O2, c=10-5 M
norm. Absorbance [a. u.]
Wavelength [nm]
0 min
20 min
40 min
60 min
80 min
100 min
120 min
140 min
0.0
0.1
0.2
0.3
0.4
0.5
0.6
0.7
0.8
0.9
200
250
300
350
400
450

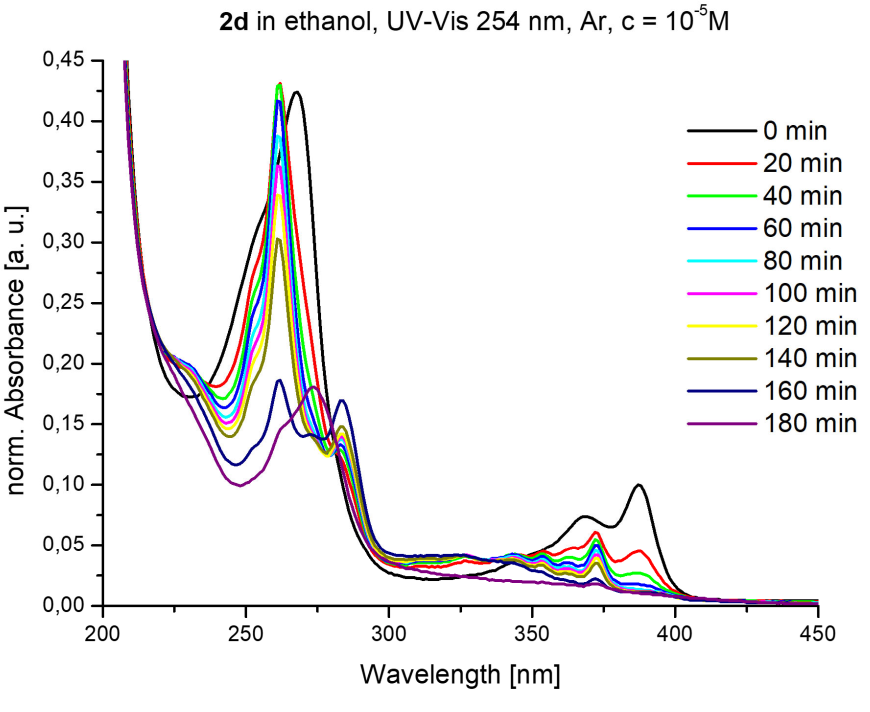
2d in ethanol, UV-Vis 254 nm, Ar, c = 10-5M
norm. Absorbance [a. u.]
Wavelength [nm]
0 min
20 min
40 min
60 min
80 min
100 min
120 min
140 min
160 min
180 min
0,00
0,05
0,10
0,15
0,20
0,25
0,30
0,35
0,40
0,45
200
250
300
350
400
450

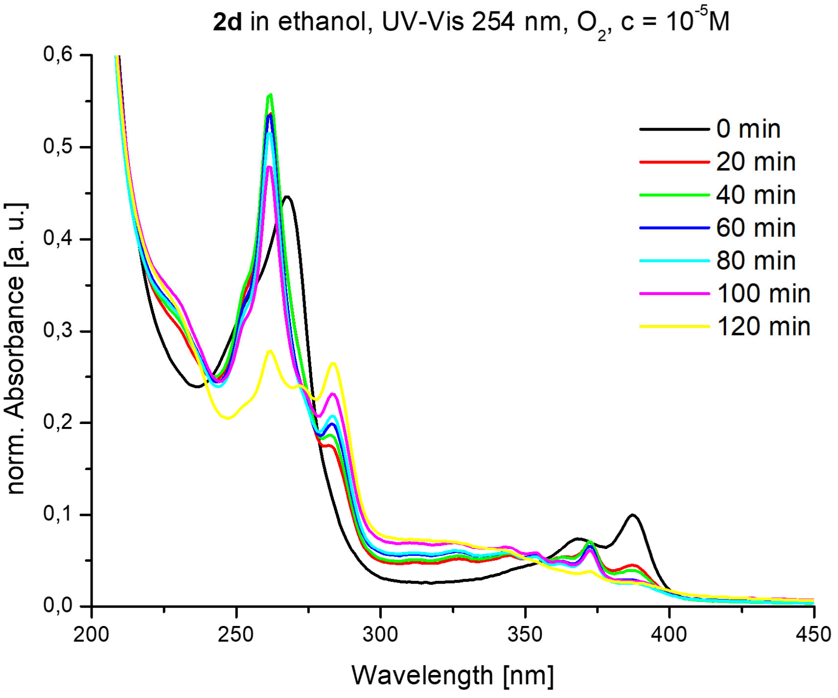
2d in ethanol, UV-Vis 254 nm, O2, c = 10-5M
norm. Absorbance [a. u.]
Wavelength [nm]
0 min
20 min
40 min
60 min
80 min
100 min
120 min
0,0
0,1
0,2
0,3
0,4
0,5
0,6
200
250
300
350
400
450

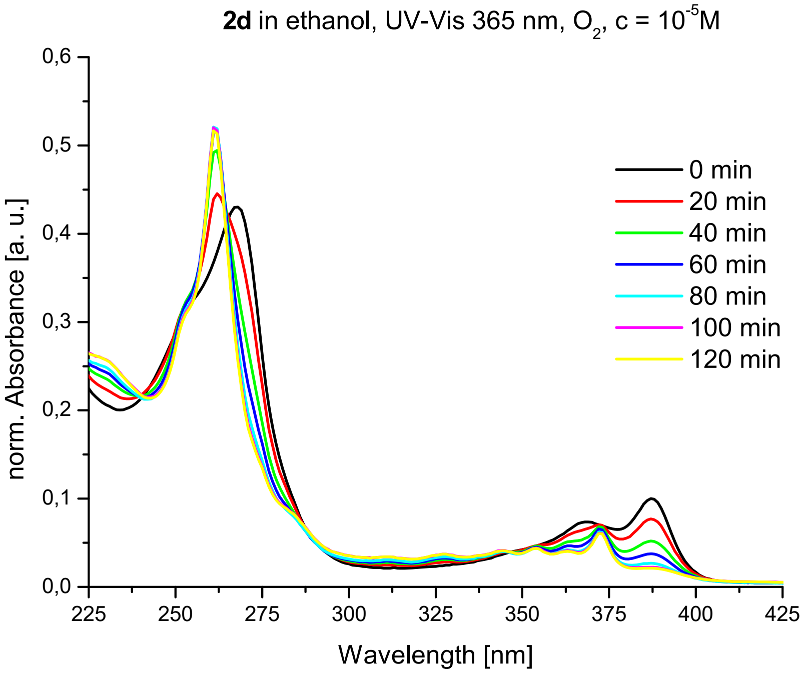
2d in ethanol, UV-Vis 365 nm, O2, c = 10-5M
norm. Absorbance [a. u.]
Wavelength [nm]
0 min
20 min
40 min
60 min
80 min
100 min
120 min
0,0
0,1
0,2
0,3
0,4
0,5
0,6
225
250
275
300
325
350
375
400
425

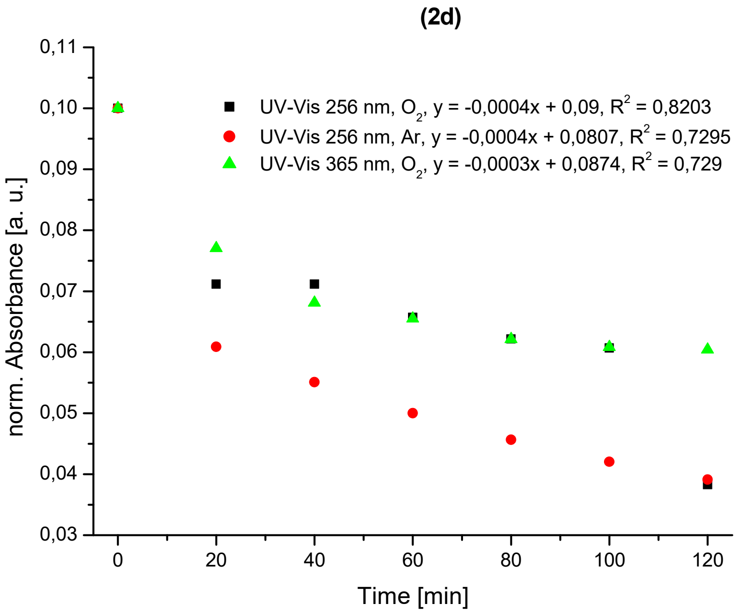
(2d)
UV-Vis 256 nm, O2, y = -0,0004x + 0,09, R2 = 0,8203
UV-Vis 256 nm, Ar, y = -0,0004x + 0,0807, R2 = 0,7295
UV-Vis 365 nm, O2, y = -0,0003x + 0,0874, R2 = 0,729
norm. Absorbance [a. u.]
Time [min]
0,11
0,10
0,09
0,08
0,07
0,06
0,05
0,04
0,03
0
20
40
60
80
100
120

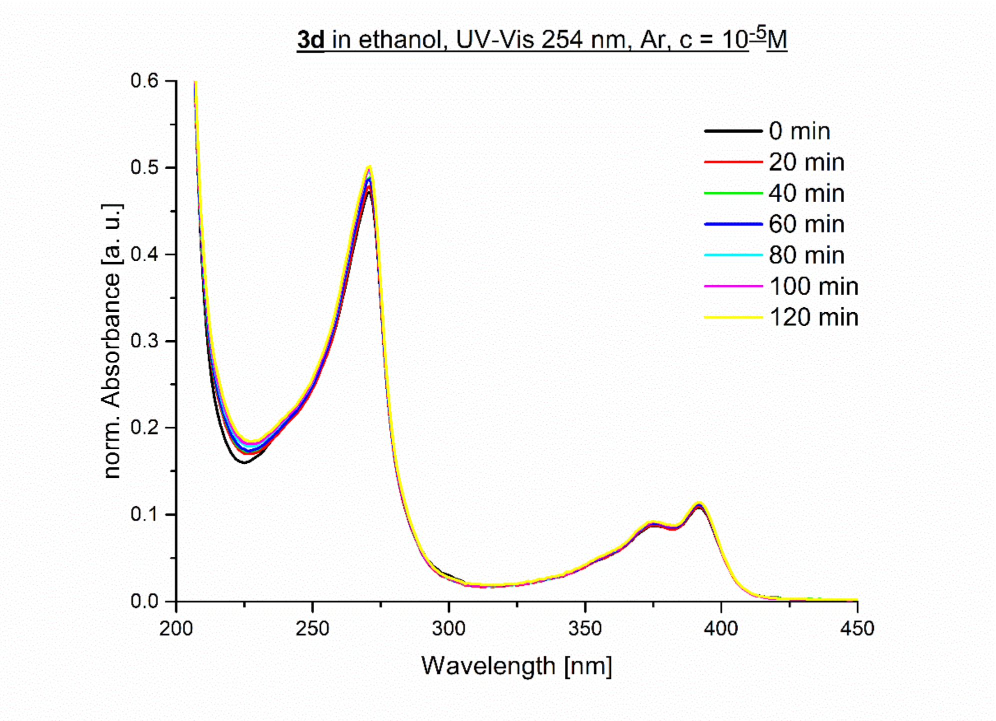
3d in ethanol, UV-Vis 254 nm, Ar, c = 10-5M
norm. Absorbance [a. u.]
Wavelength [nm]
0 min
20 min
40 min
60 min
80 min
100 min
120 min
0.0
0.1
0.2
0.3
0.4
0.5
0.6
200
250
300
350
400
450

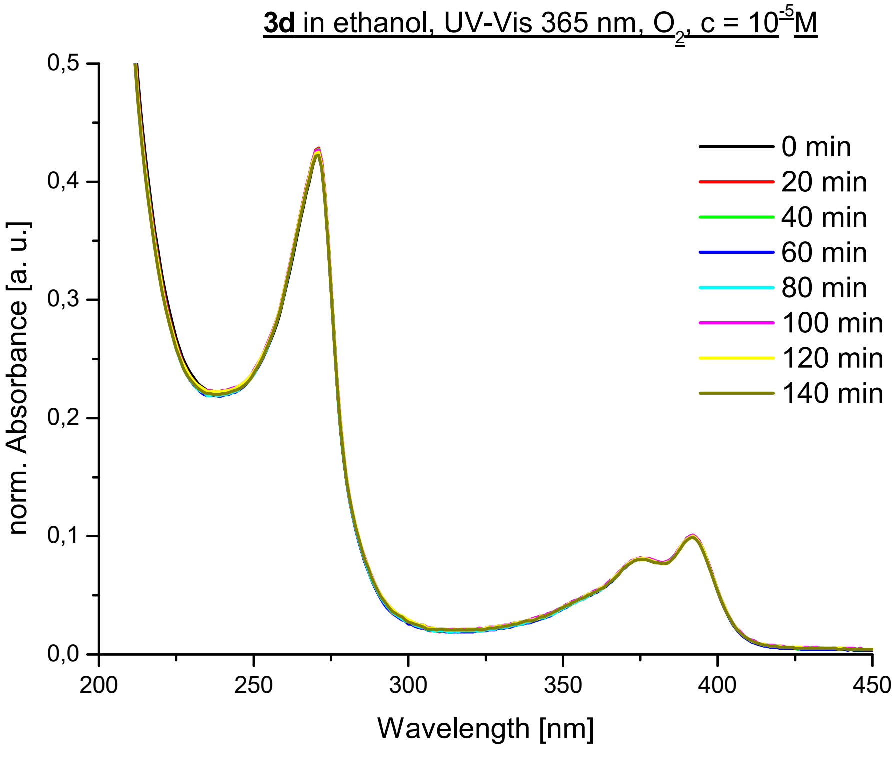
3d in ethanol, UV-Vis 365 nm, O2, c = 10-5M
norm. Absorbance [a. u.]
Wavelength [nm]
0 min
20 min
40 min
60 min
80 min
100 min
120 min
140 min
0,0
0,1
0,2
0,3
0,4
0,5
200
250
300
350
400
450

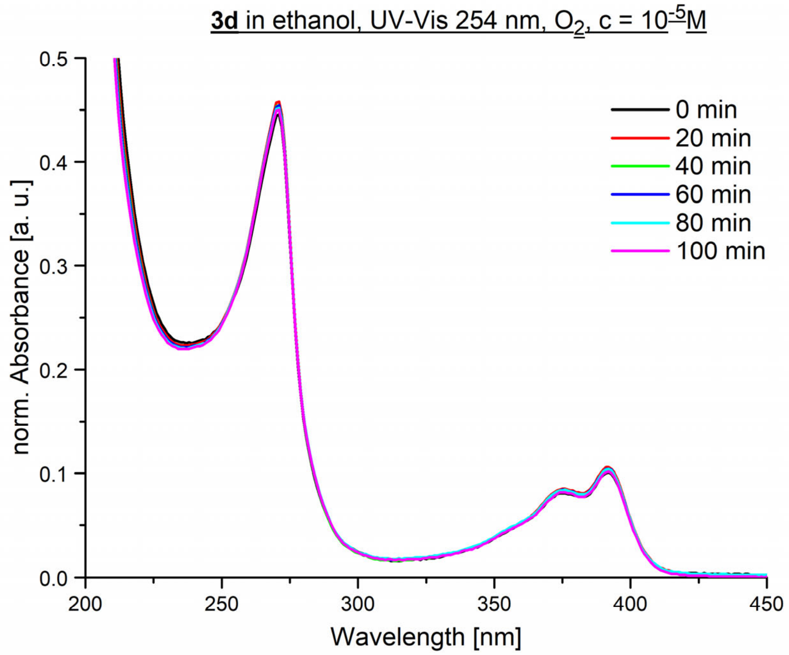
3d in ethanol, UV-Vis 254 nm, O2, c = 10-5M
0.5
0.4
0.3
0.2
0.1
0.0
norm. Absorbance [a. u.]
200
250
300
350
400
450
Wavelength [nm]
0 min
20 min
40 min
60 min
80 min
100 min

**HRMS-(+)-APCI spectra**

The photodegradation was investigated by monitoring the MS spectra of $10^{-5}$ M ethanolic solutions in quartz cuvettes at room temperature, under ambient atmosphere before and after exposure to UVP-Hg-Pen-ray lamp (254 nm, 16.33 mW/cm$^2$ at distance 1 cm) for 3 hours (irradiation dose 176 J/cm$^2$) and 9 hours (irradiation dose 528 J/cm$^2$). In order to distinguish fragmentation peaks from the peaks due to the photo-products in the irradiated mixture, a HR(MS_MS)-(+)-APCI fragmentation of single peaks in whole spectra was carried out to conclude that the detected peaks for all investigated compounds were due to the real photoproducts in the mixture.The HR(MS_MS)-(+)-APCI spectra were recorded at 15 eV, 25 eV and 35 eV.

**Figure S9.** HRMS-(+)-APCI spectra of **1a**.
(The right spectrum shows fragmentation of **1a** after irradiation for 3 hours at 254 nm under air $O_2$ in EtOH and the left spectrum shows fragmentation of the starting acene **1a**)

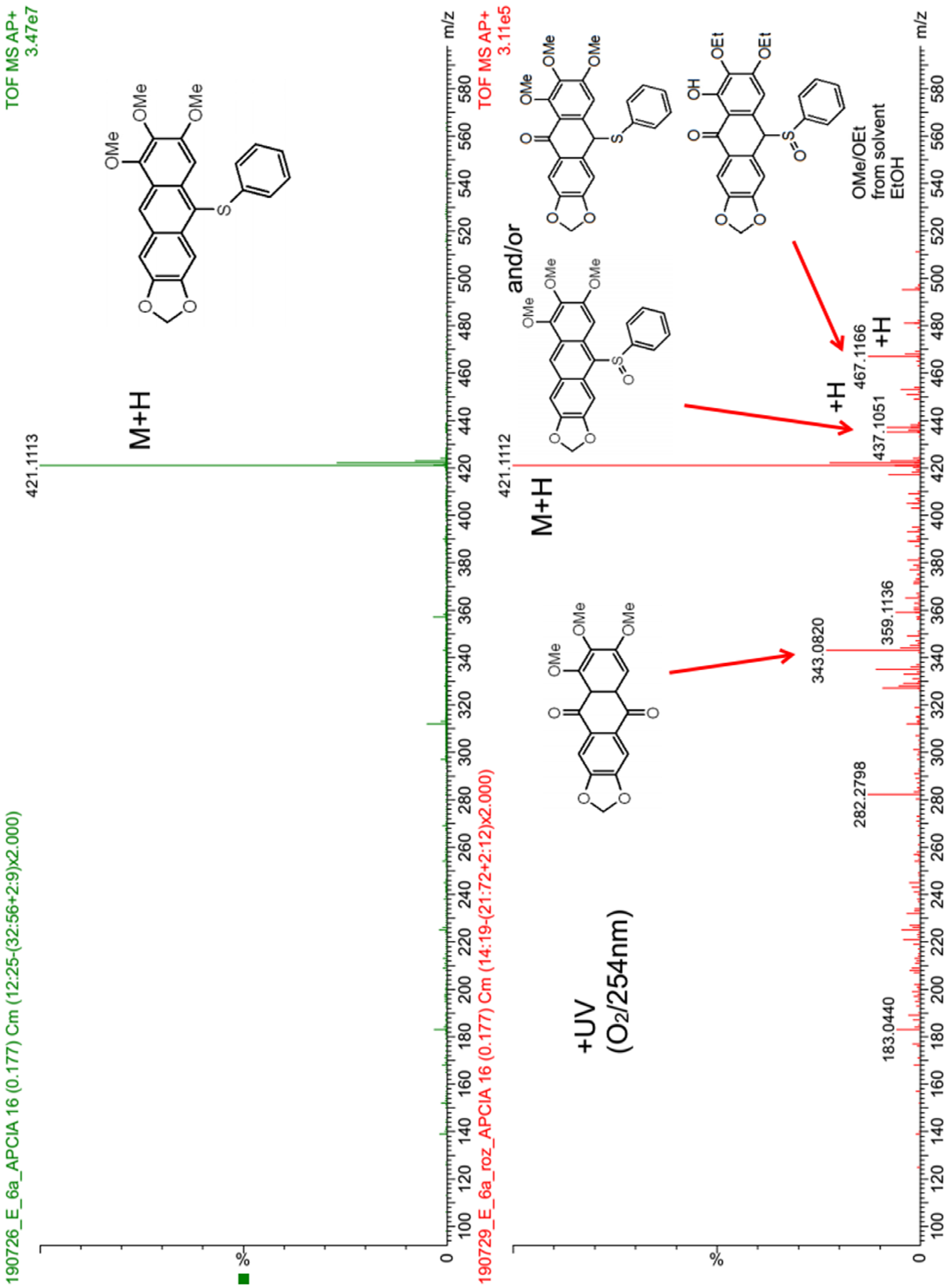

**Figure S10.** HRMS-(+)-APCI spectra of **1b**.
(The right spectrum shows fragmentation of **1b** after irradiation for 3 hours at 254 nm under air $O_2$ in EtOH and the left spectrum shows fragmentation of the starting acene **1b**)

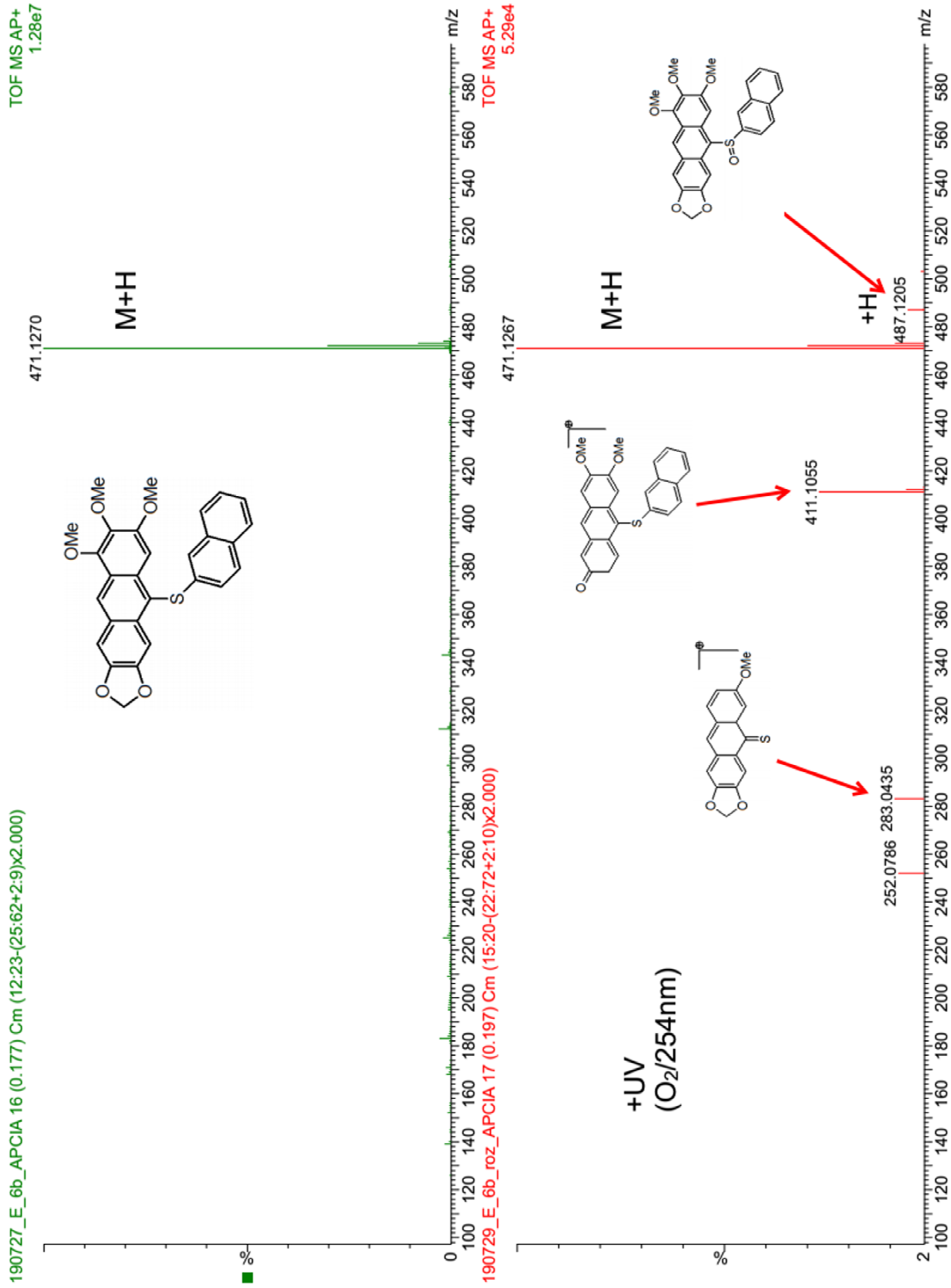

**Figure S11.** HRMS-(+)-APCI spectra of **1d**.
(The right spectrum shows fragmentation of **1d** after irradiation for 3 hours at 254 nm under air $O_2$ in EtOH and the left spectrum shows fragmentation of the starting acene **1d**)

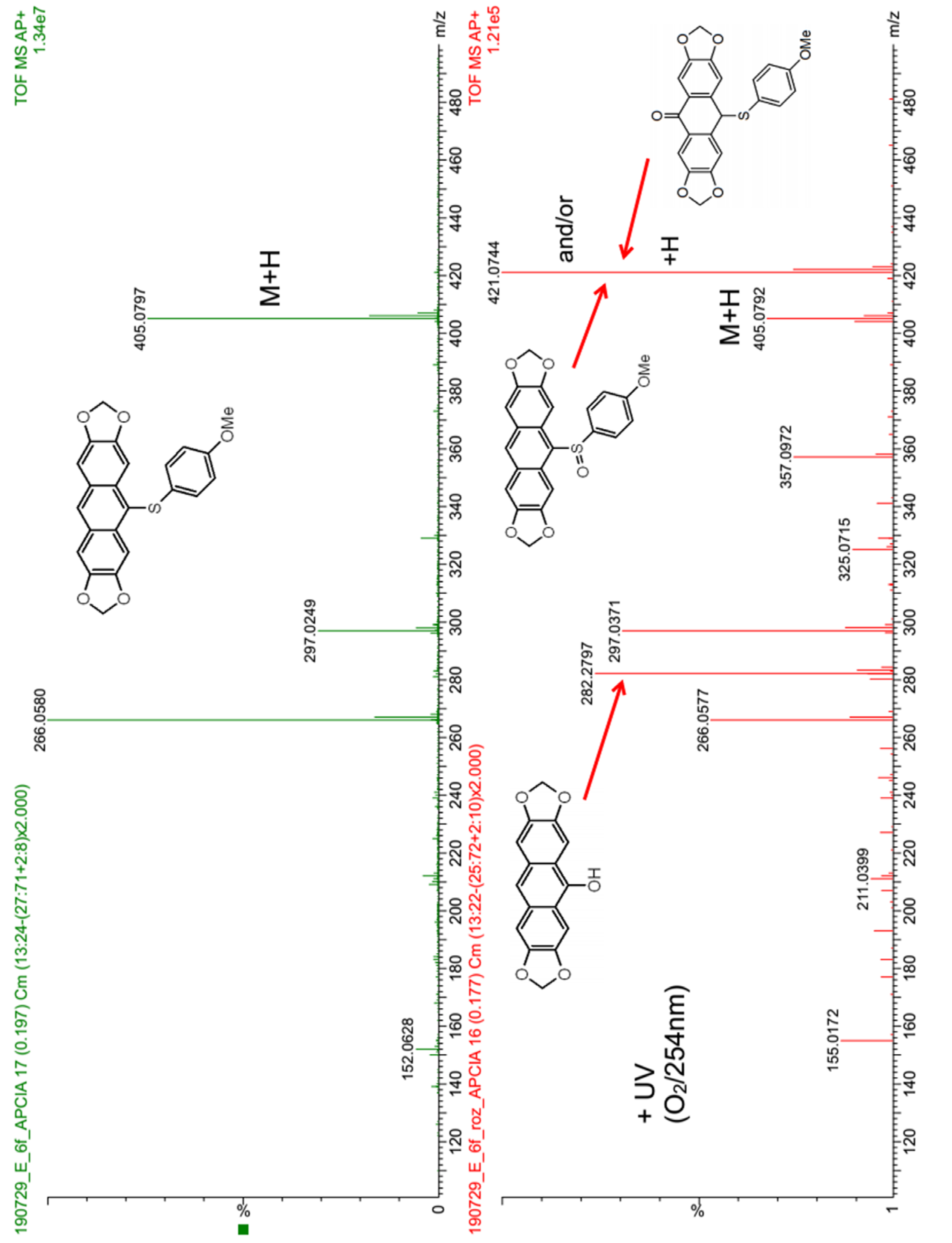

**Figure S12.** HRMS-(+)-APCI spectra of **2a**.
(The left spectrum shows fragmentation of **2a** after irradiation for 3 hours at 254 nm under air $O_2$ in EtOH and the right spectrum shows fragmentation of the starting acene **2a**)

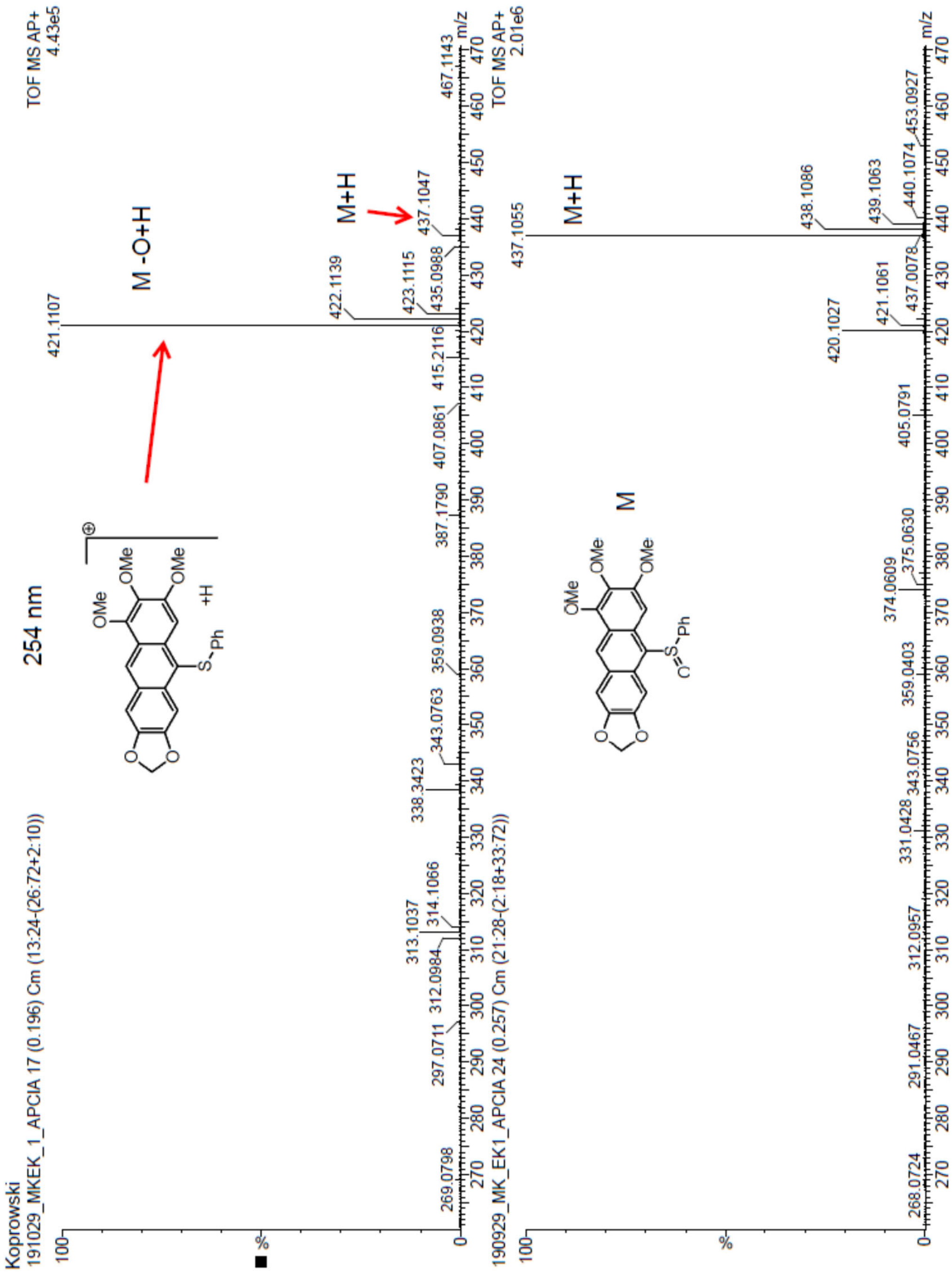

The HRMS-(+)-APCI spectrum of ethanolic solution of **2a** after irradiation for 9 hours at 254 nm

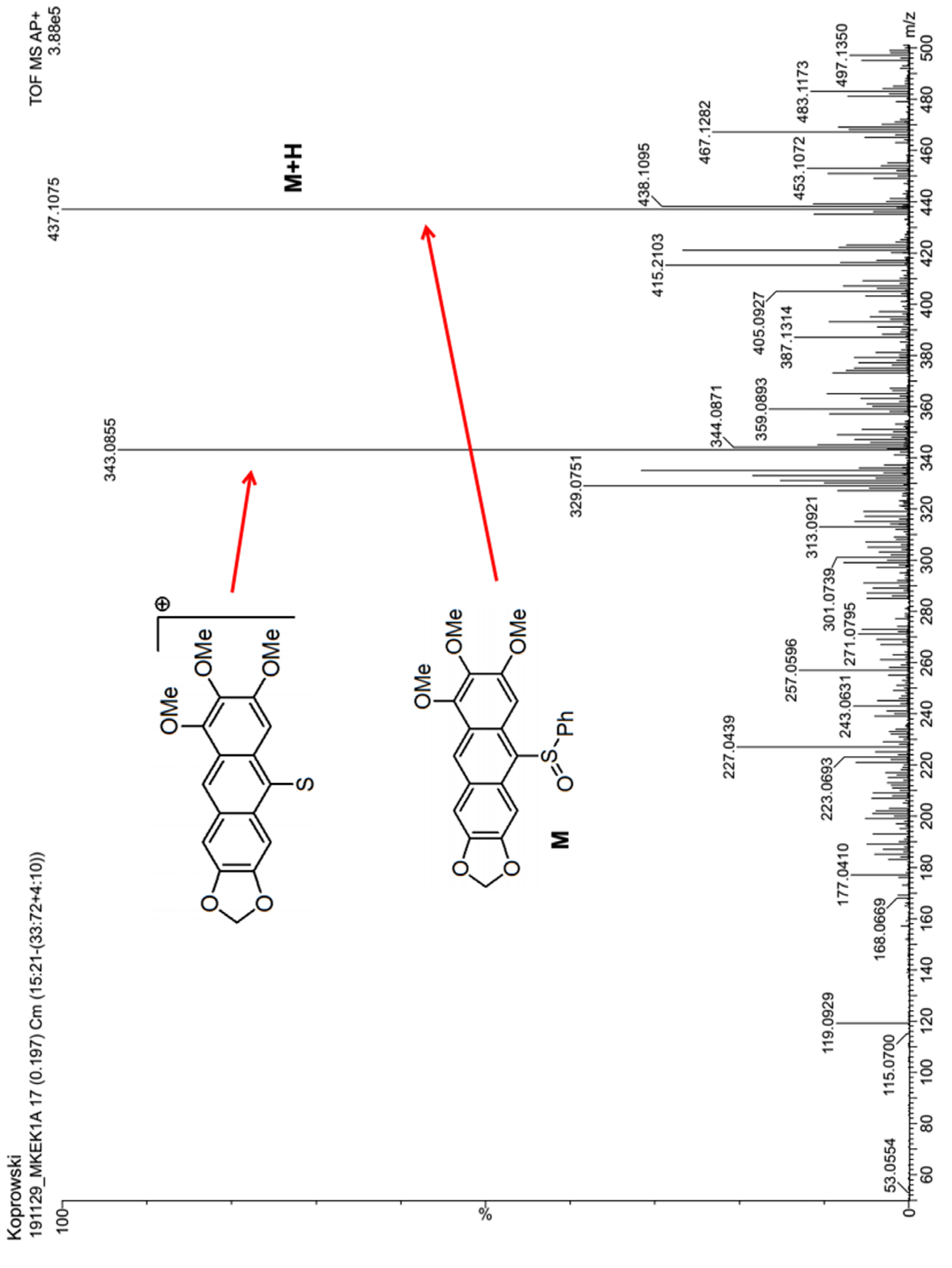

The HR(MS_MS)-(+)-APCI fragmentation spectrum of the peak at $m/z$ = 421 (15eV) after irradiation of ethanolic solution of **2a** for 9 hours at 254 nm

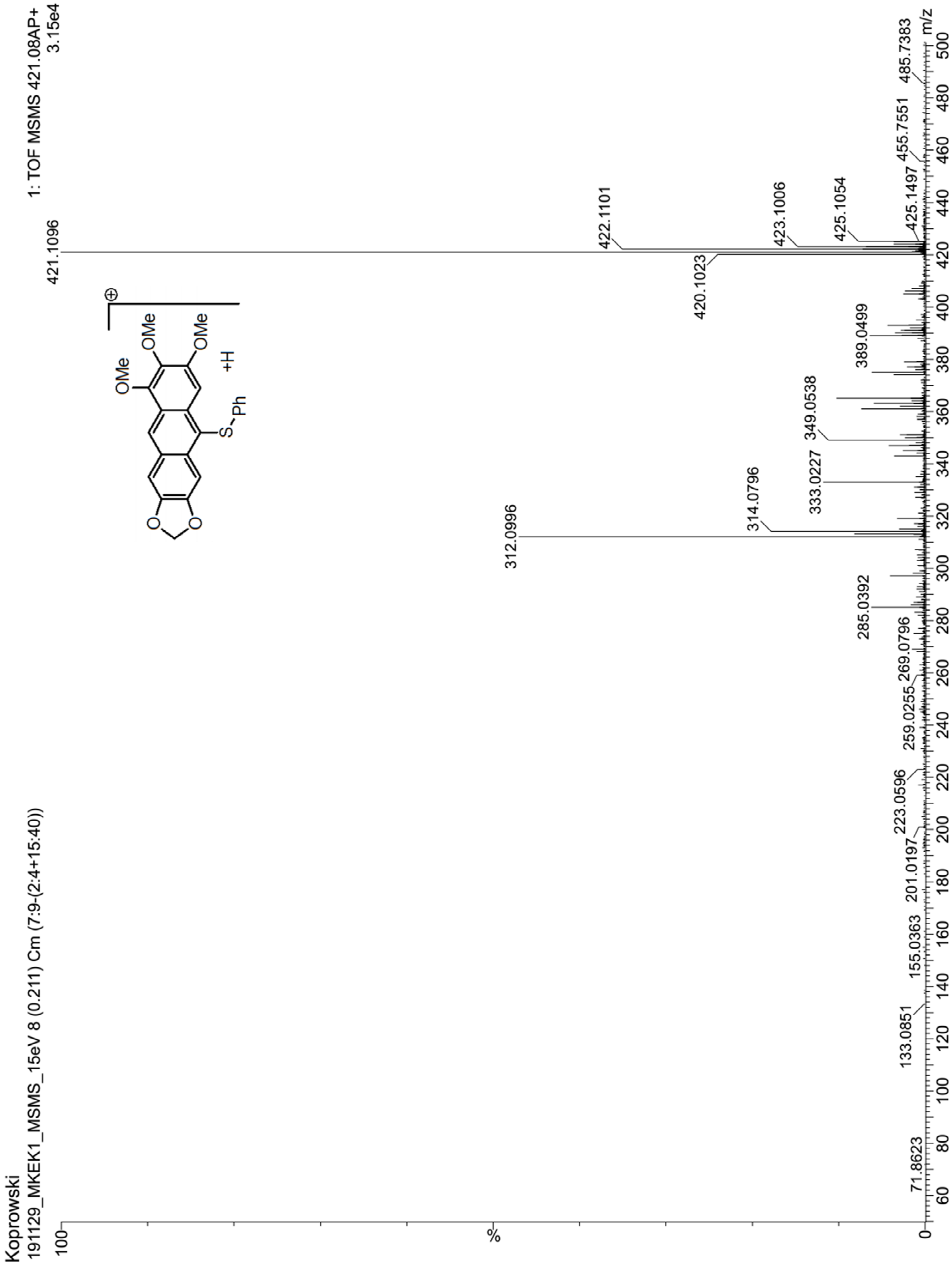

The HR(MS_MS)-(+)-APCI fragmentation spectrum of the peak at *m*/*z* = 421 (25eV) after irradiation of ethanolic solution of **2a** for 9 hours at 254 nm

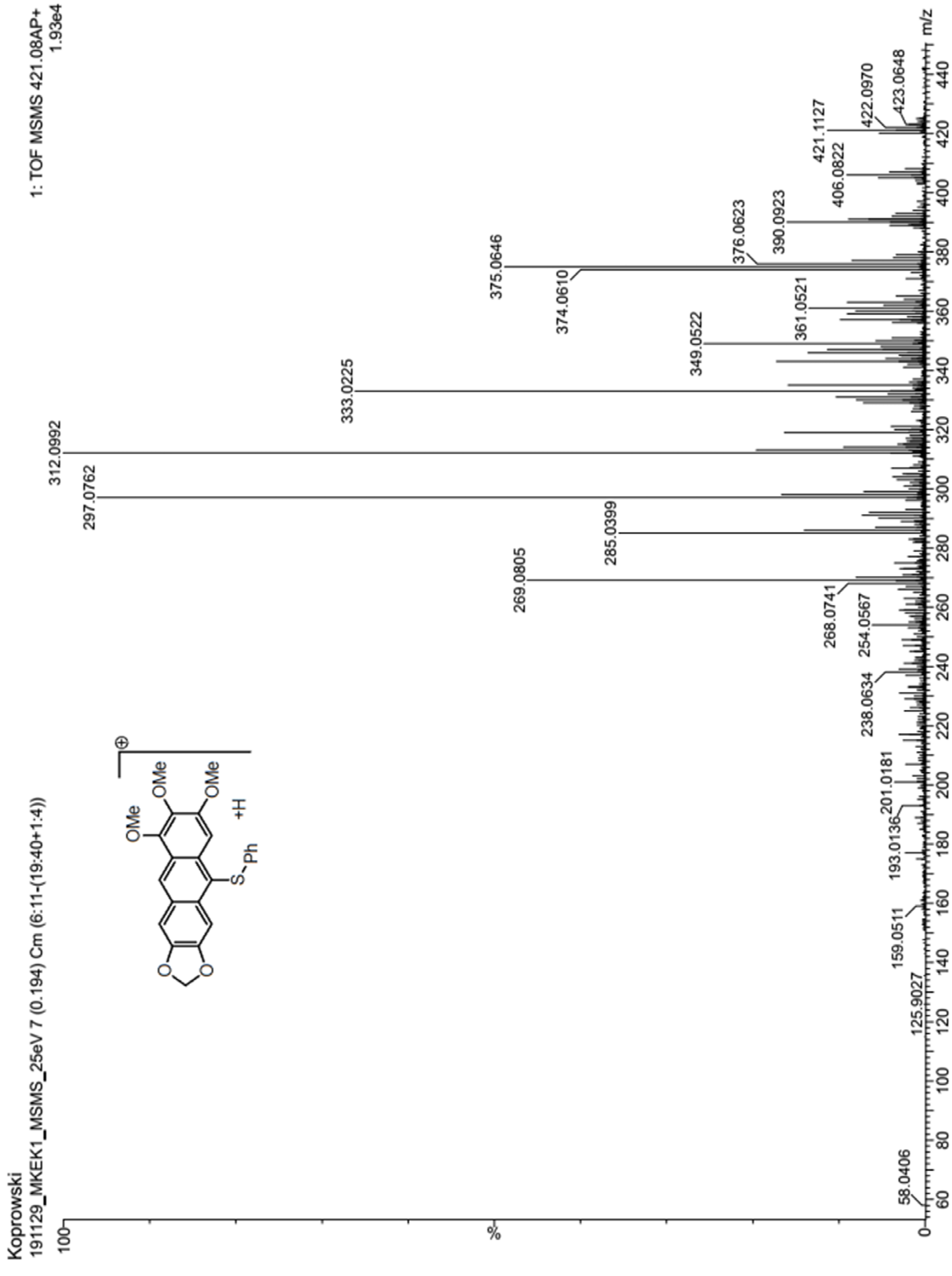

**Figure S13.** HRMS-(+)-APCI spectra of **2d**.
(The left spectrum shows fragmentation of **2d** after irradiation for 3 hours at 254 nm under air $O_2$ in EtOH and the right spectrum shows fragmentation of the starting acene **2d**)

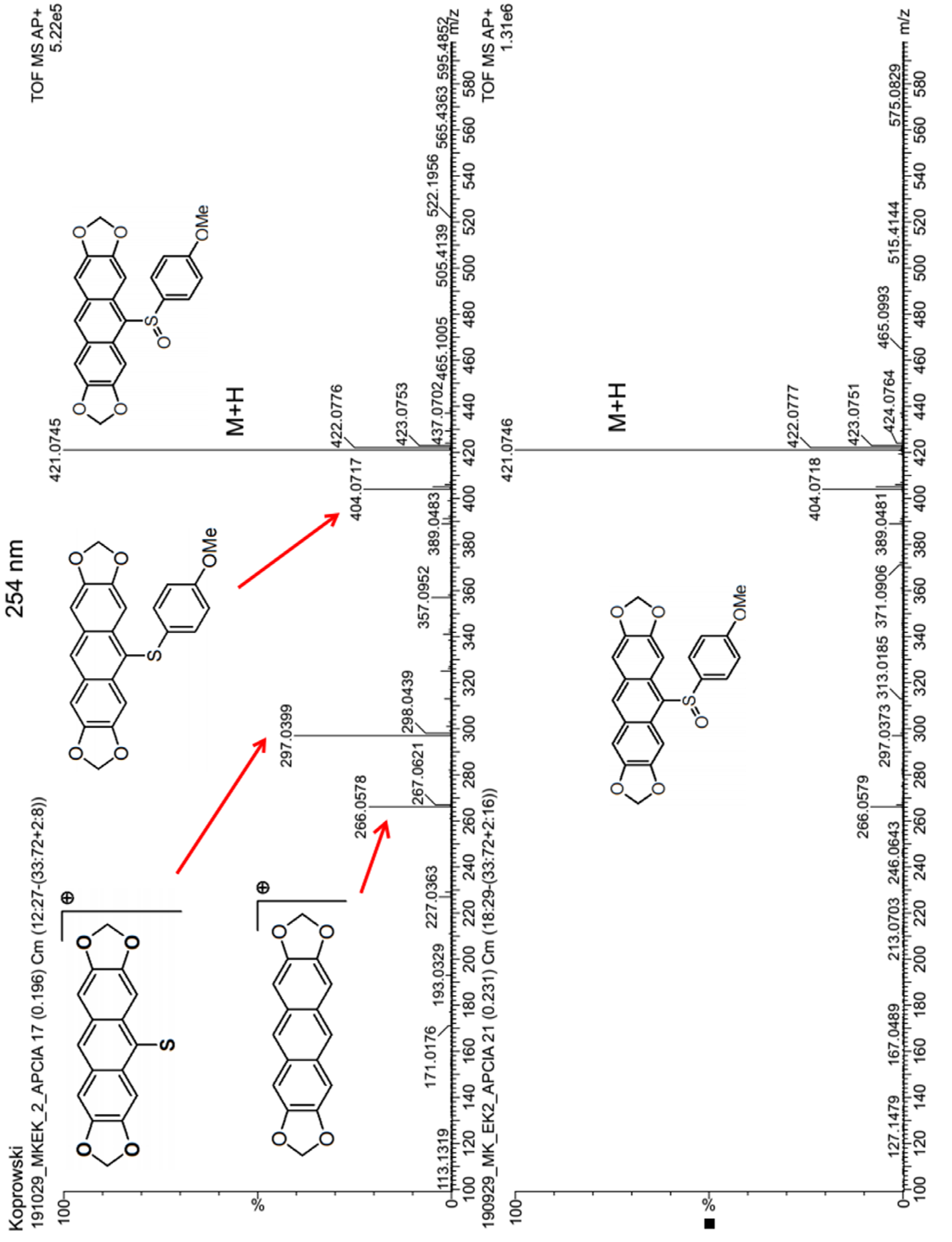


The HRMS-(+)-APCI spectrum of ethanolic solution of **2d** after irradiation for 9 hours at 254 nm

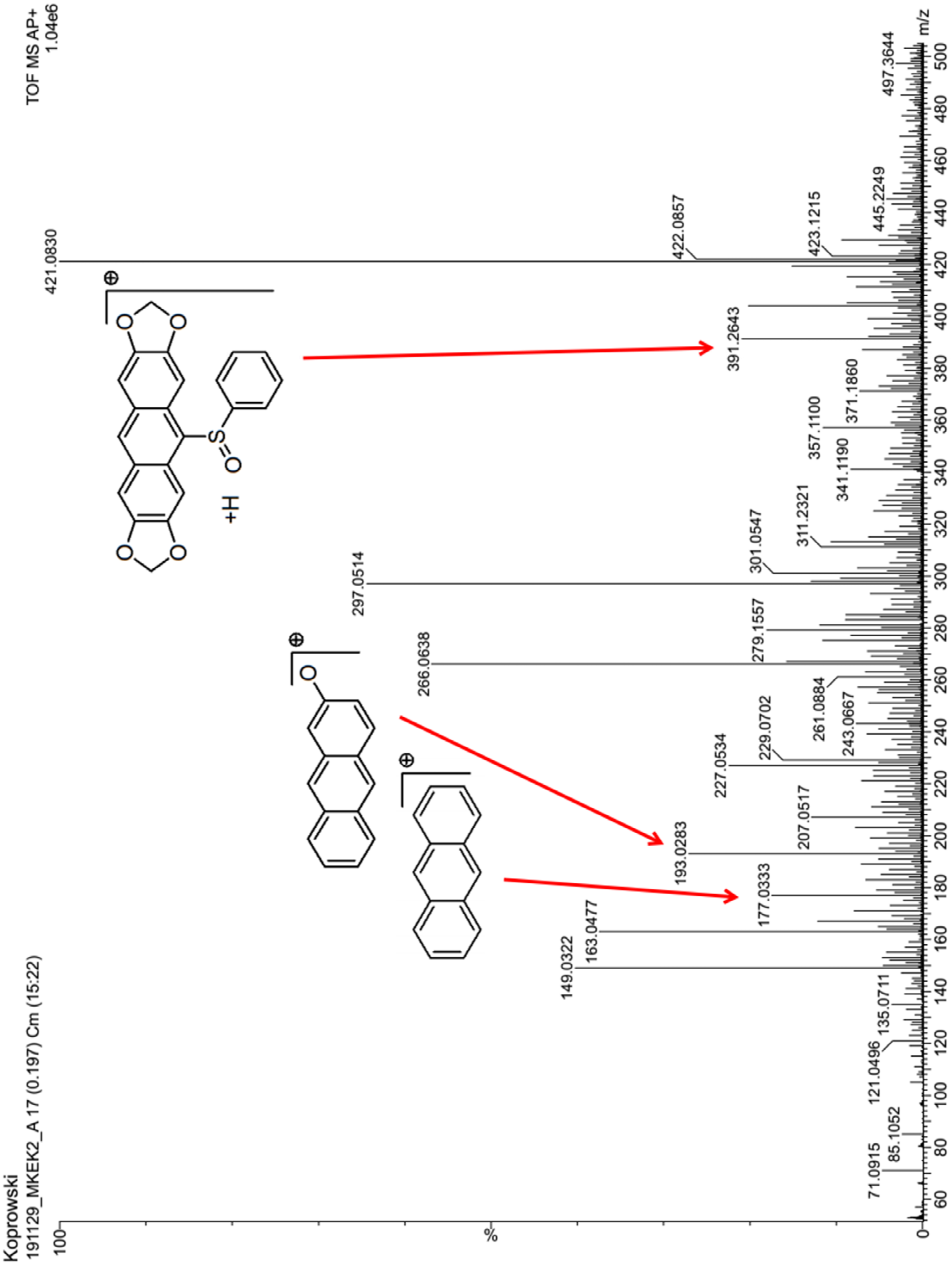


The HR(MS_MS)-(+)-APCI fragmentation spectrum of the peak at $m/z$ = 266 (25eV) after irradiation of ethanolic solution of **2d** for 9 hours at 254 nm

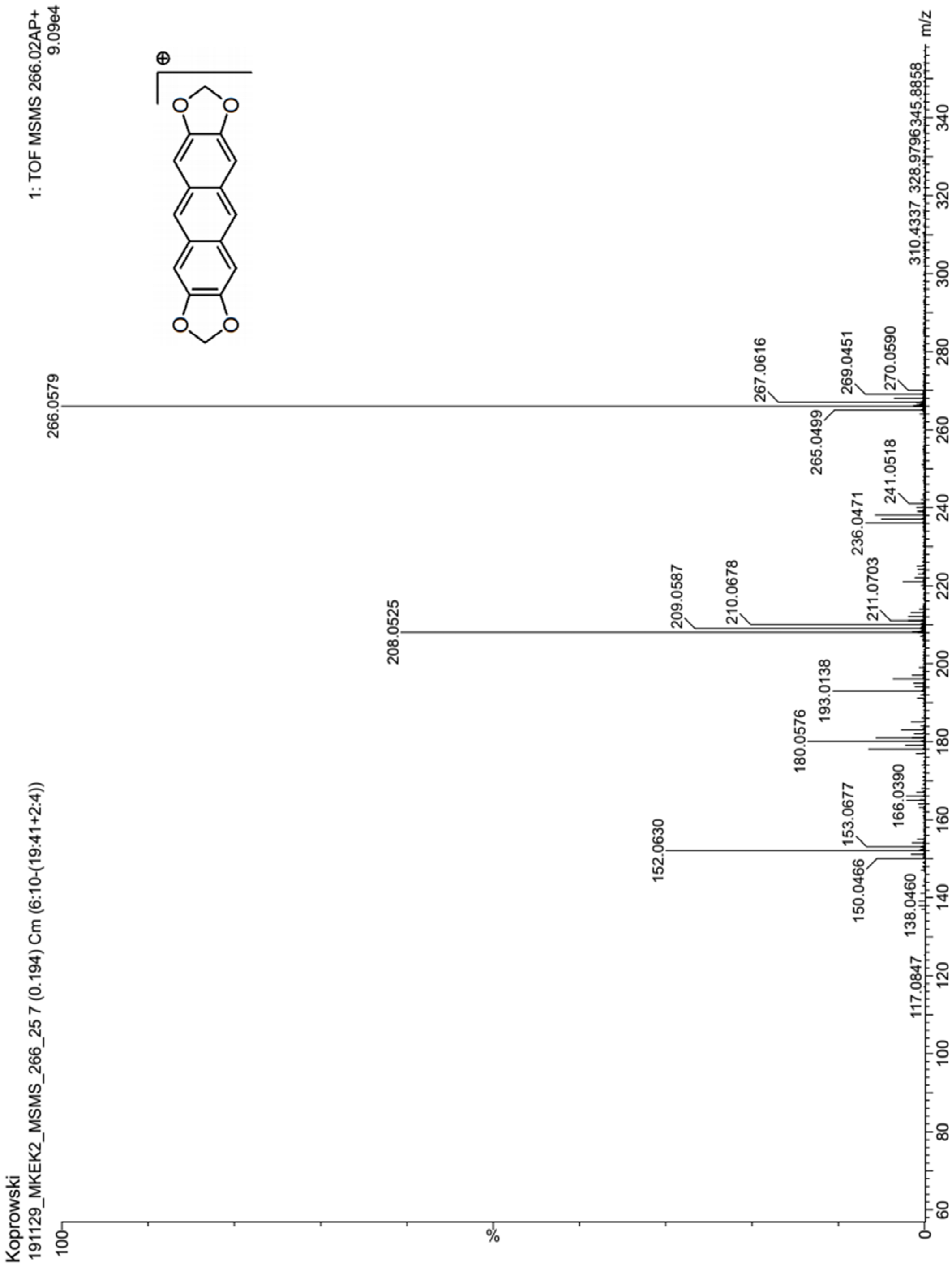


The HR(MS_MS)-(+)-APCI fragmentation spectrum of the peak at $m/z$ = 266 (35eV) after irradiation of ethanolic solution of **2d** for 9 hours at 254 nm

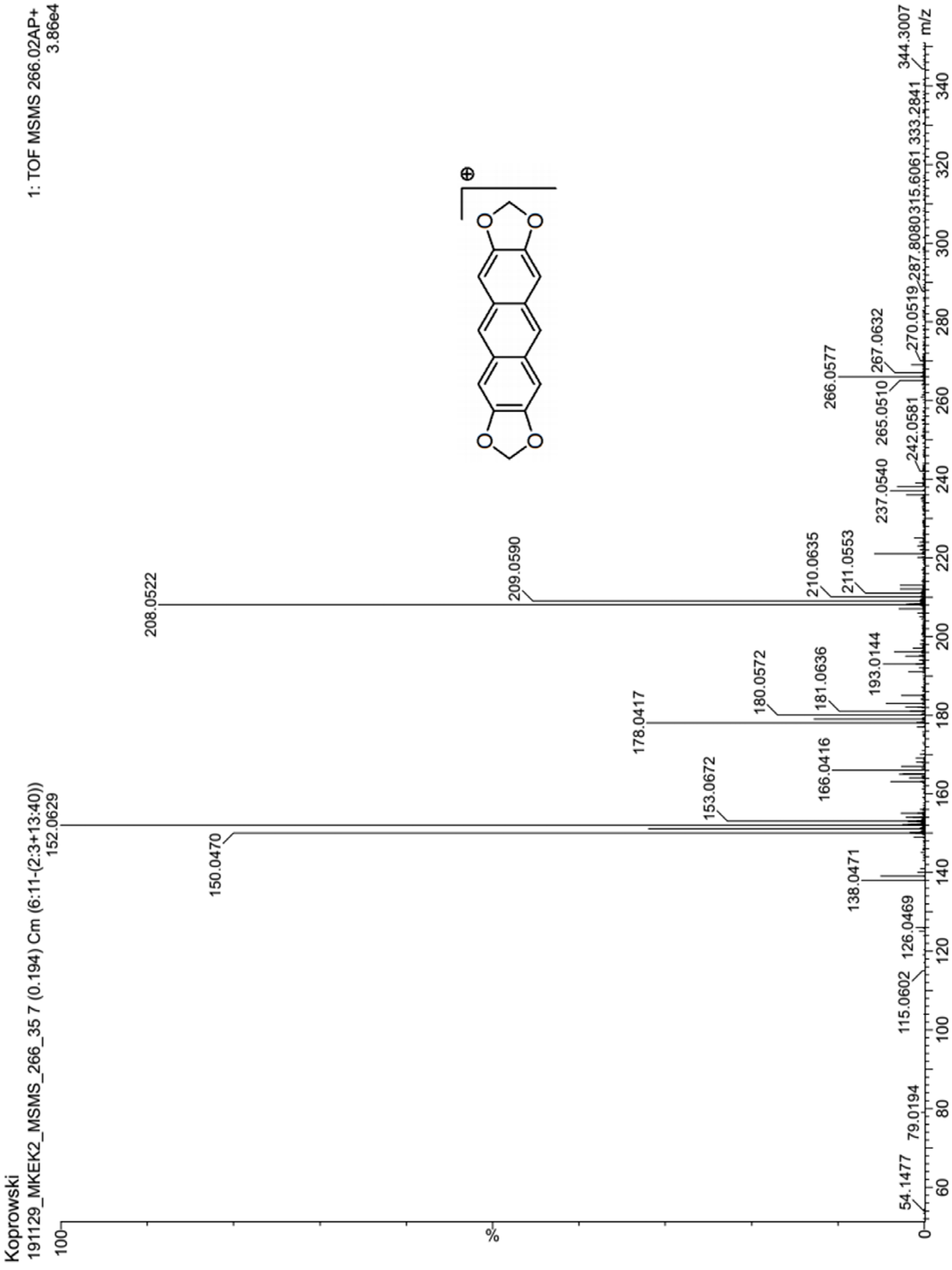


The HR(MS_MS)-(+)-APCI fragmentation spectrum of the peak at $m/z$ = 297 (25eV) after irradiation of ethanolic solution of **2d** for 9 hours at 254 nm

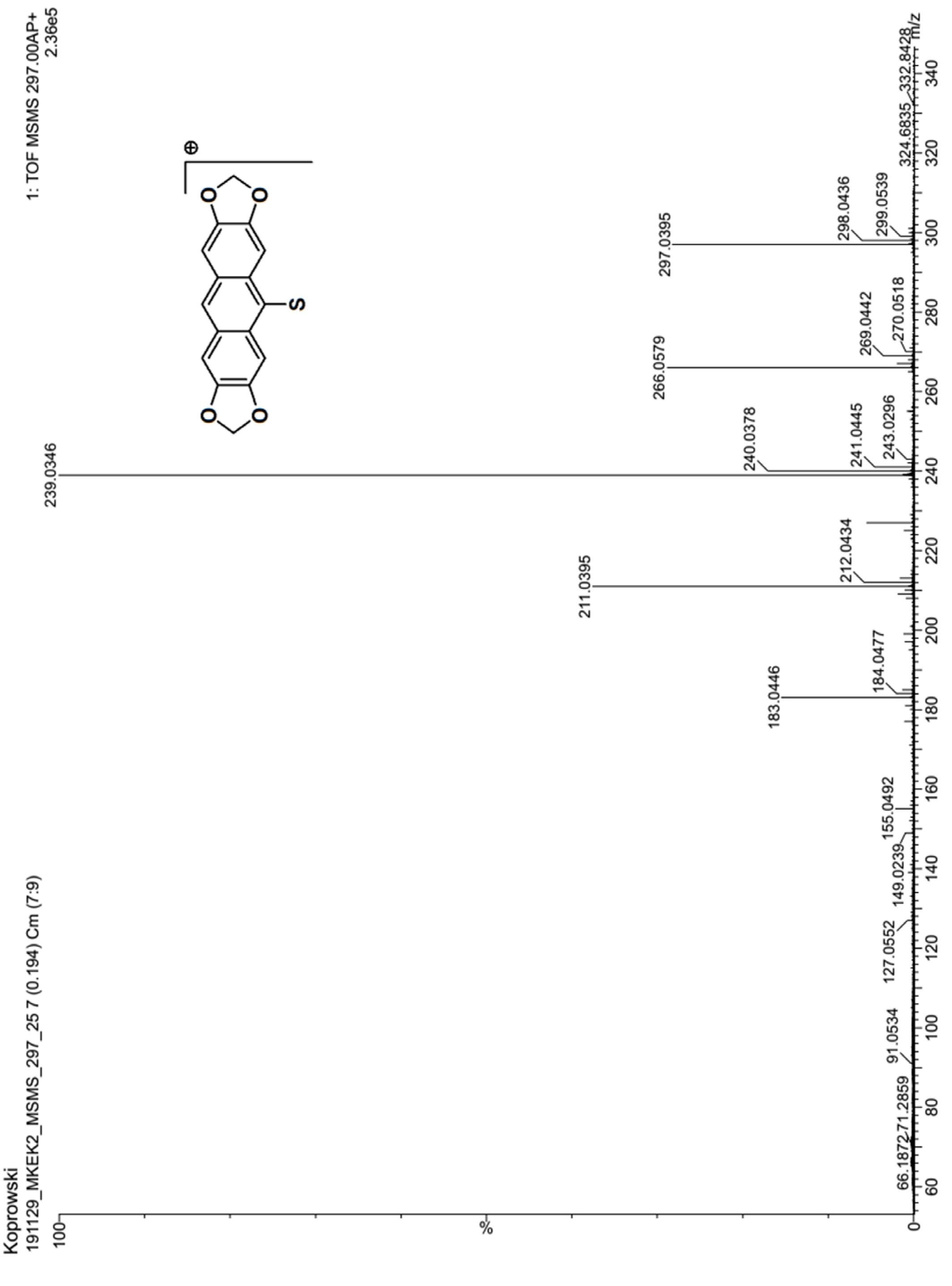


The HR(MS_MS)-(+)-APCI fragmentation spectrum of the peak at $m/z$ = 297 (35eV) after irradiation of ethanolic solution of **2d** for 9 hours at 254 nm

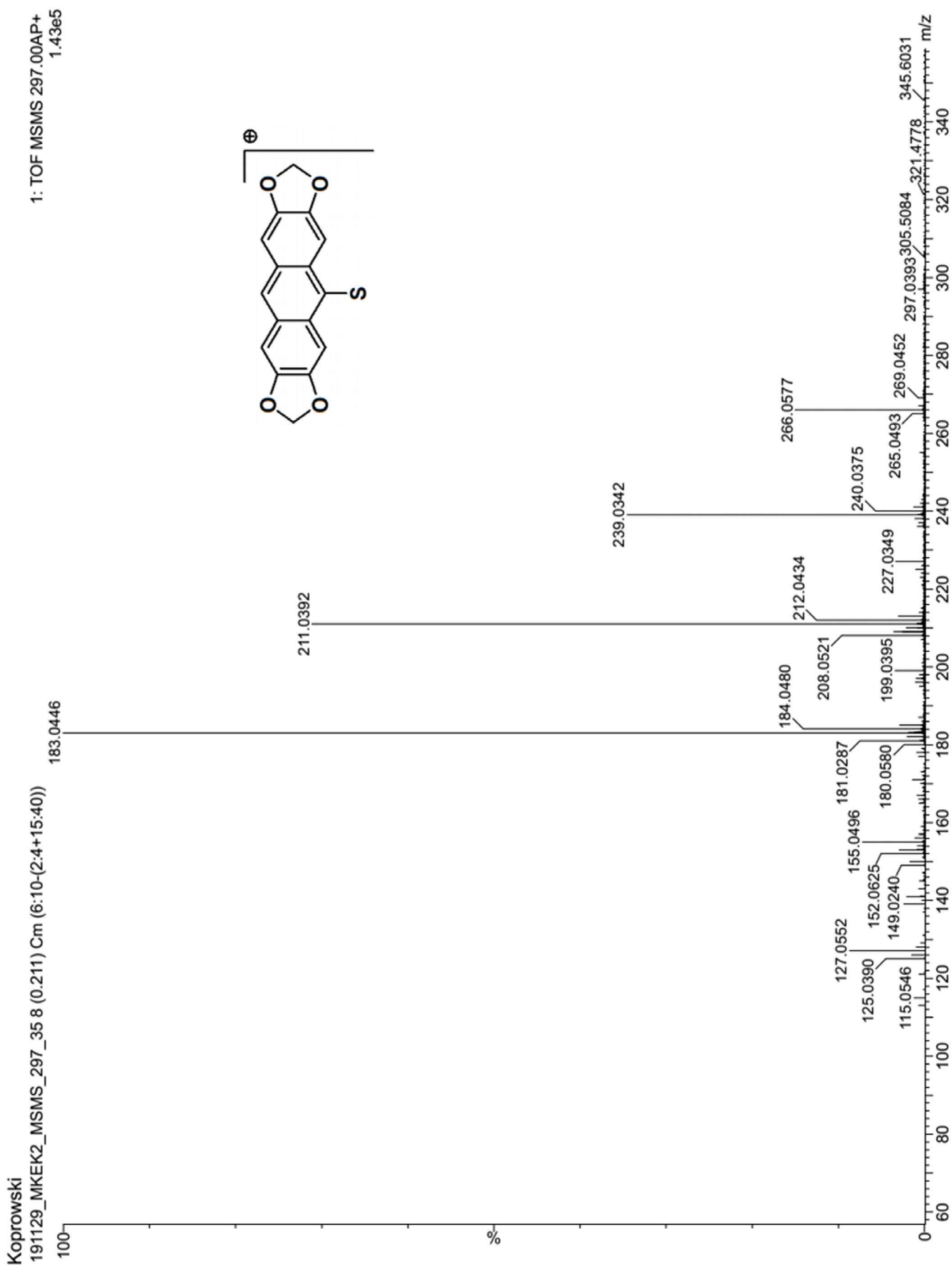


The HR(MS_MS)-(+)-APCI fragmentation spectrum of the peak at $m/z$ = 404 (25eV) after irradiation of ethanolic solution of **2d** for 9 hours at 254 nm

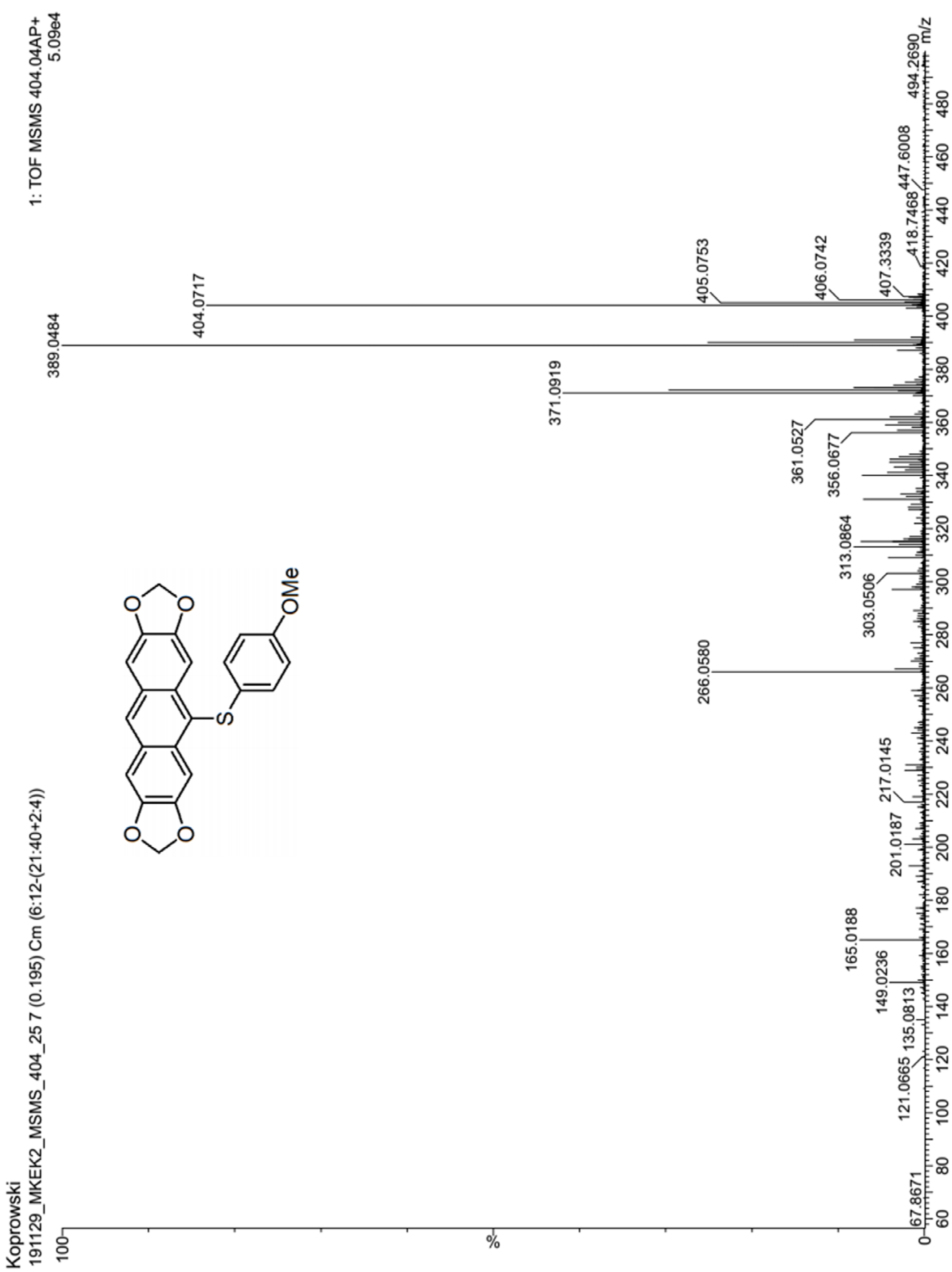


The HR(MS_MS)-(+)-APCI fragmentation spectrum of the peak at $m/z$ = 404 (35eV) after irradiation of ethanolic solution of **2d** for 9 hours at 254 nm

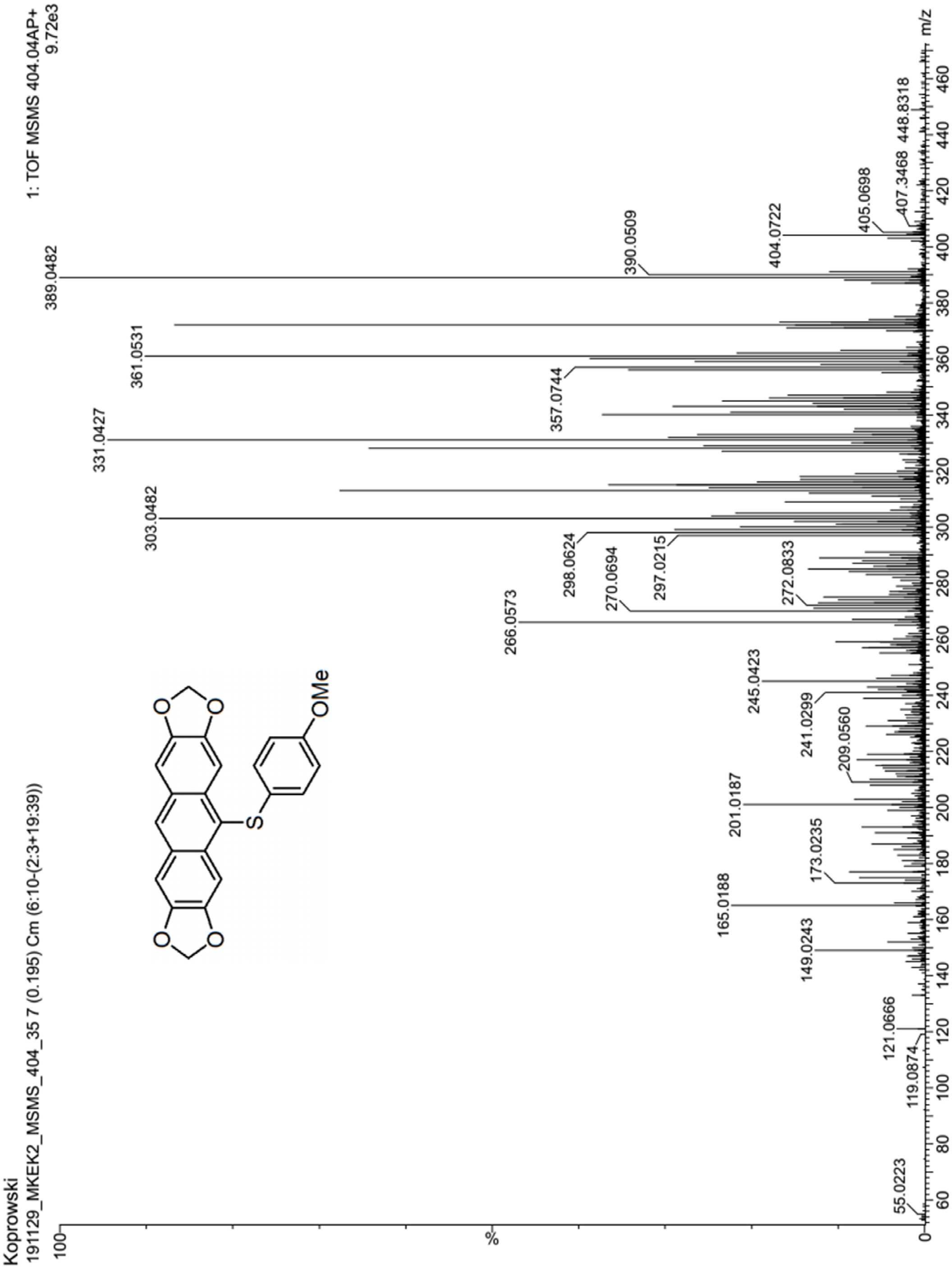
Koprowski
191129_MKEK2_MSMS_404_35 7 (0.195) Cm (6:10-(2:3+19:39))
1: TOF MSMS 404.04AP+
9.72e3
OMe
S
389.0482
390.0509
404.0722
405.0698
407.3468
448.8318
361.0531
357.0744
331.0427
303.0482
298.0624
297.0215
270.0694
272.0833
266.0573
245.0423
241.0299
209.0560
201.0187
173.0235
165.0188
149.0243
121.0666
119.0874
55.0223
100
%
0
m/z

**Figure S14.** HRMS-(+)-APCI spectra of **3c**.
(The left spectrum shows fragmentation of **3c** after irradiation for 3 hours at 254 nm under air $O_2$ in EtOH and the right spectrum shows fragmentation of the starting acene **3c**)

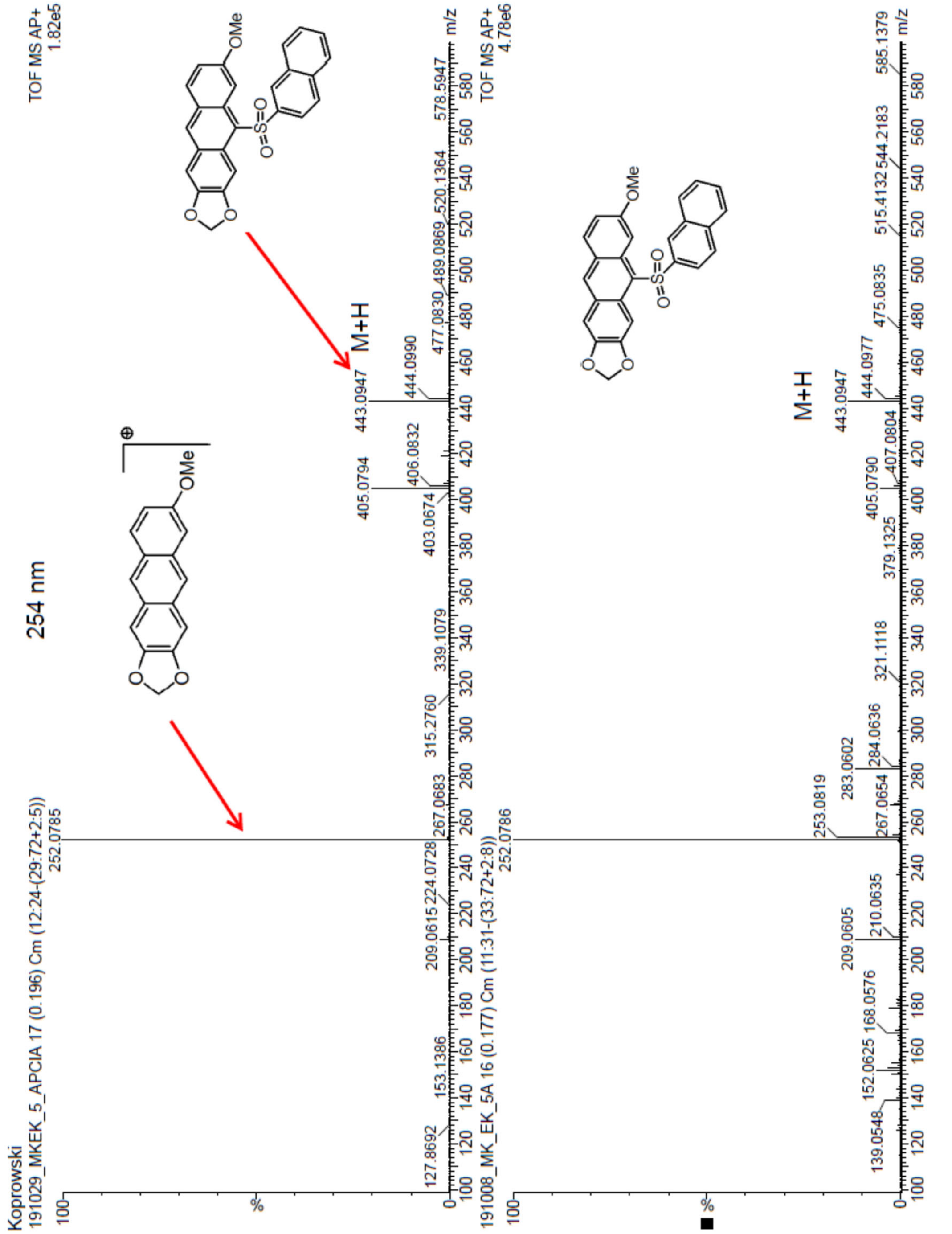

The HRMS-(+)-APCI spectrum of ethanolic solution of **3c** after irradiation for 9 hours at 254 nm

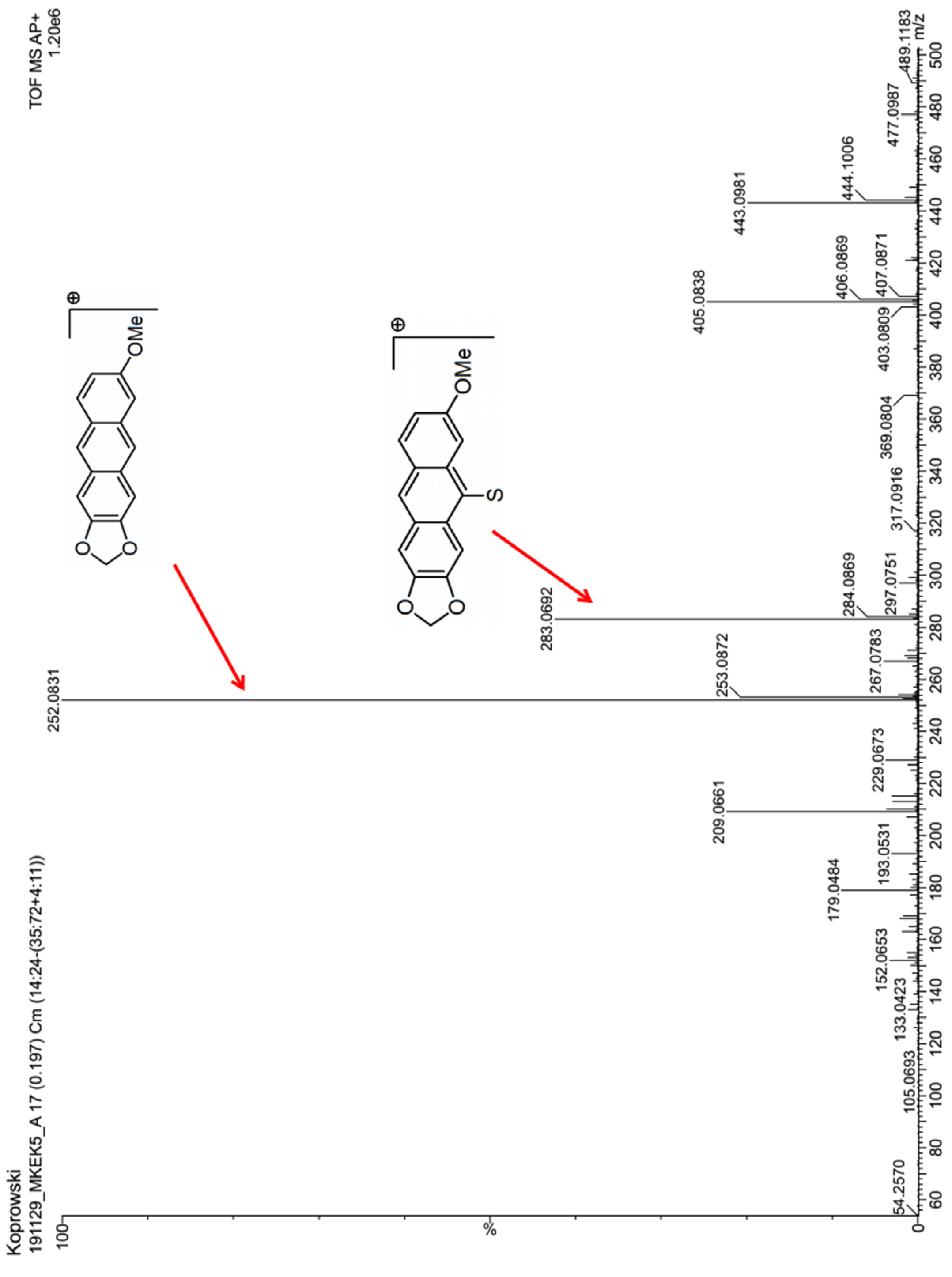

The HR(MS_MS)-(+)-APCI fragmentation spectrum of the peak at $m/z$ = 252 (15eV) after irradiation of ethanolic solution of **3c** for 9 hours at 254 nm

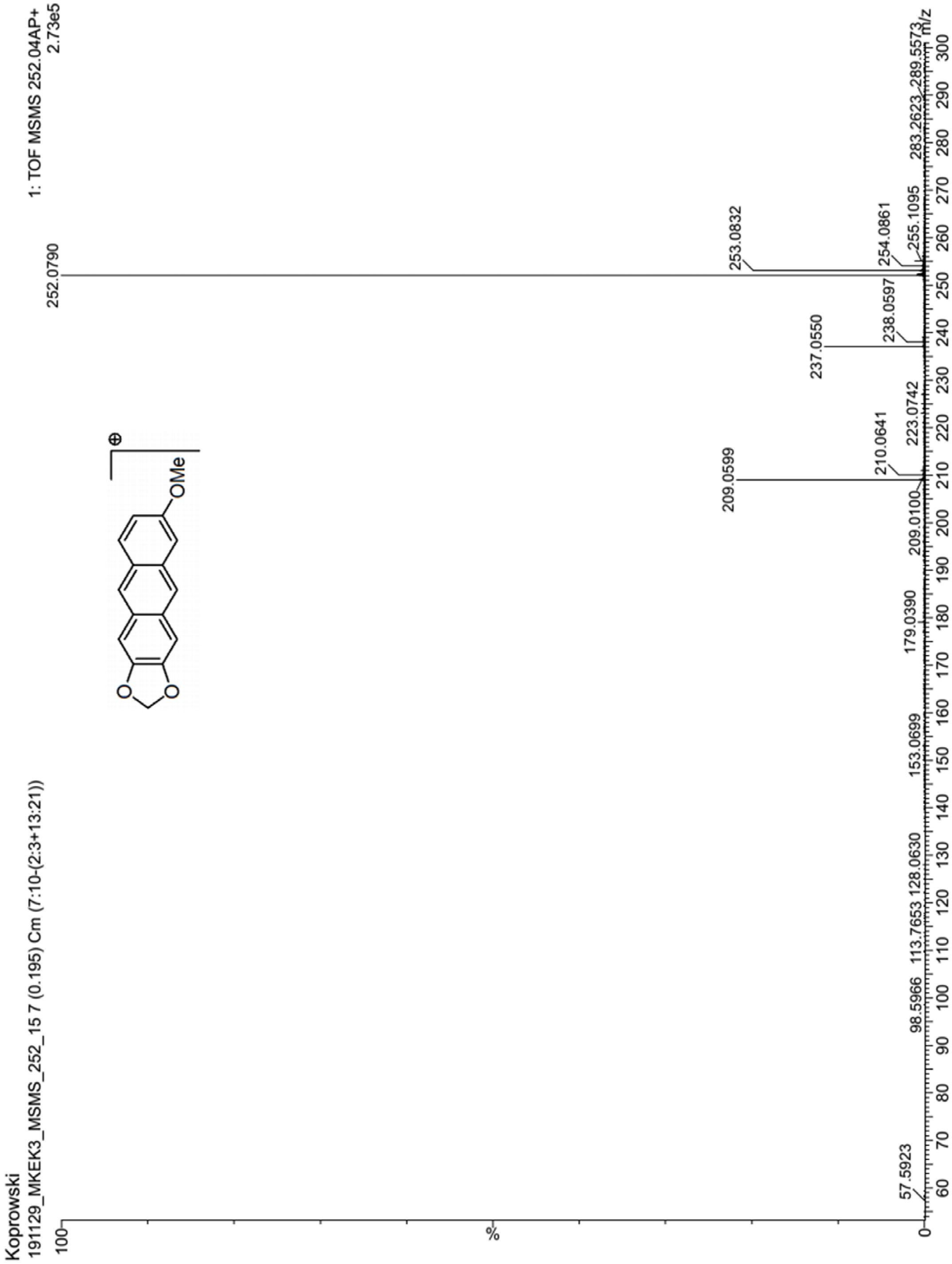

The HR(MS_MS)-(+)-APCI fragmentation spectrum of the peak at $m/z$ = 252 (25eV) after irradiation of ethanolic solution of **3c** for 9 hours at 254 nm

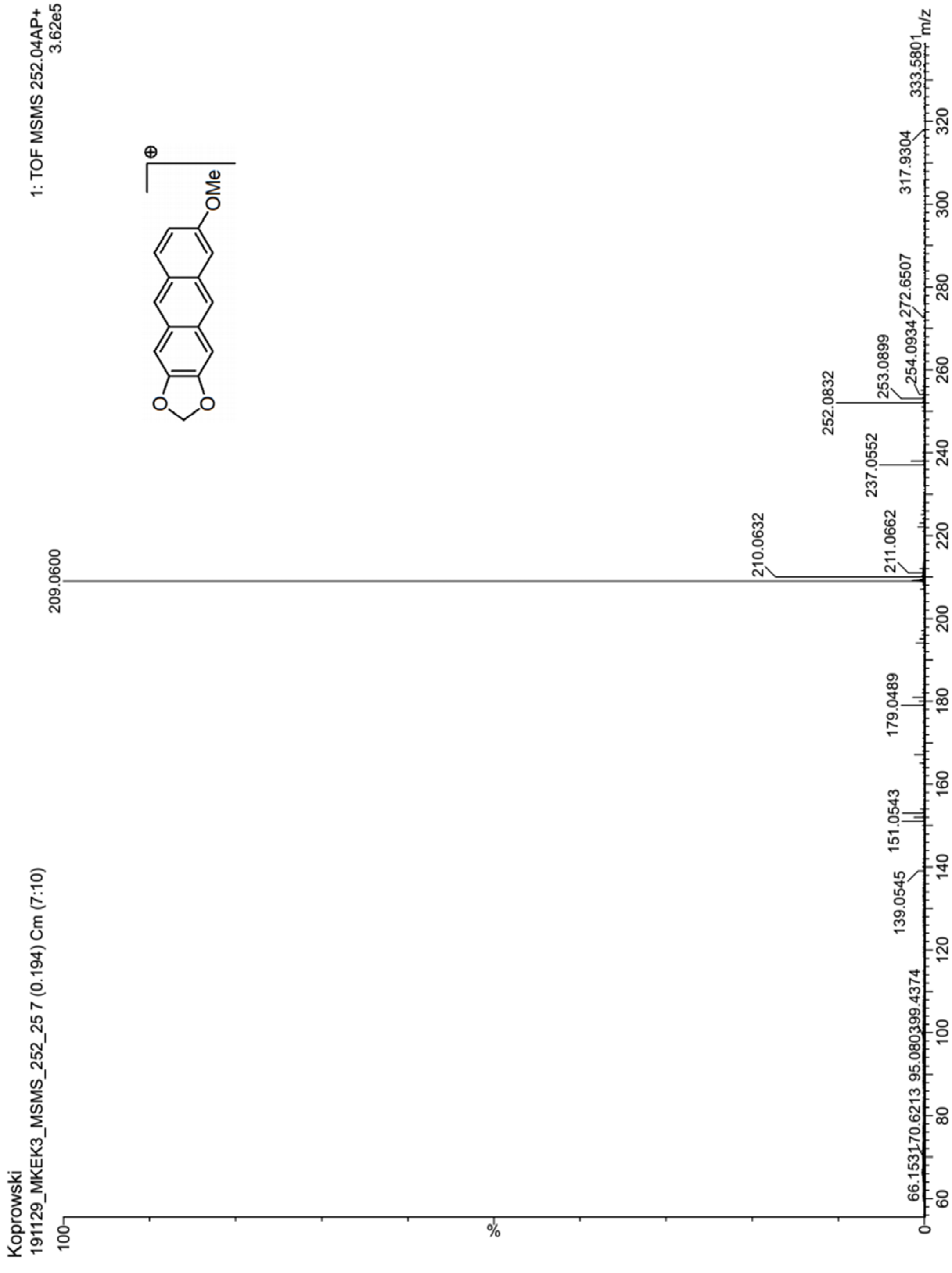

The HR(MS_MS)-(+)-APCI fragmentation spectrum of the peak at $m/z$ = 252 (35eV) after irradiation of ethanolic solution of **3c** for 9 hours at 254 nm

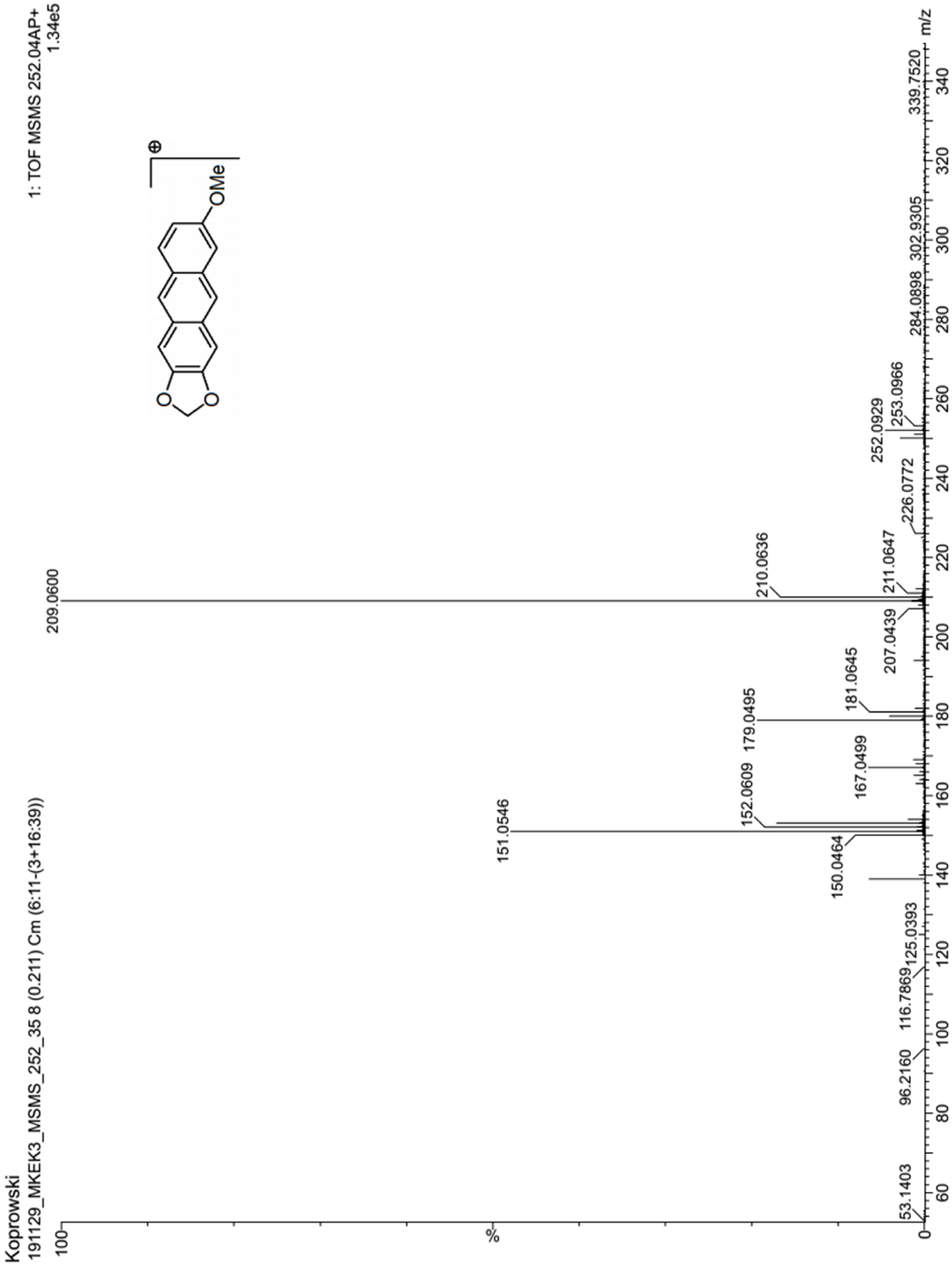

**Figure S15.** HRMS-(+)-APCI spectra of **3d**.
(The left spectrum shows fragmentation of **3d** after irradiation for 3 hours at 254 nm under air $O_2$ in EtOH and the right spectrum shows fragmentation of the starting acene **3d**)

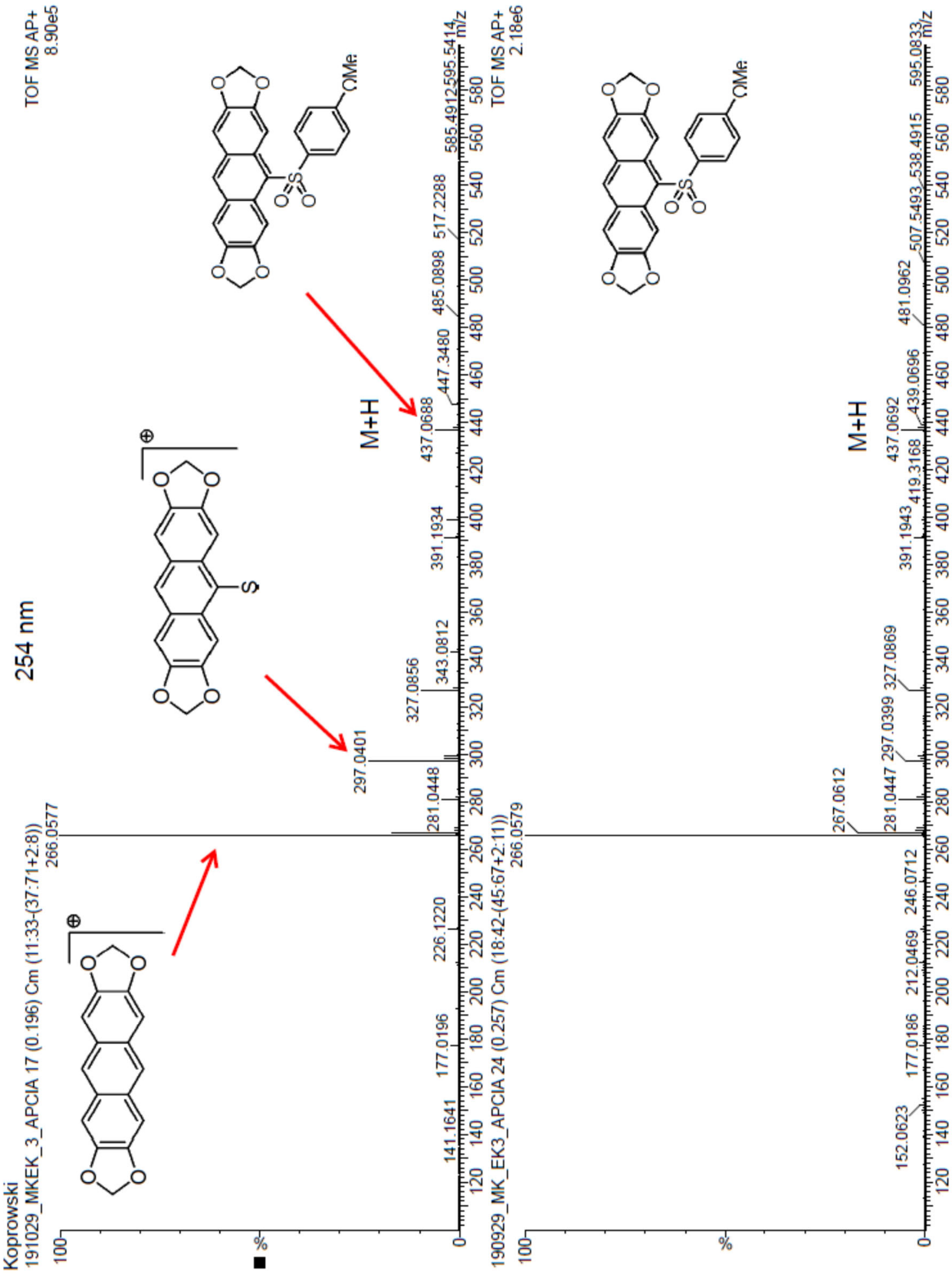

The HRMS-(+)-APCI spectrum of ethanolic solution of **3d** after irradiation for 9 hours at 254 nm

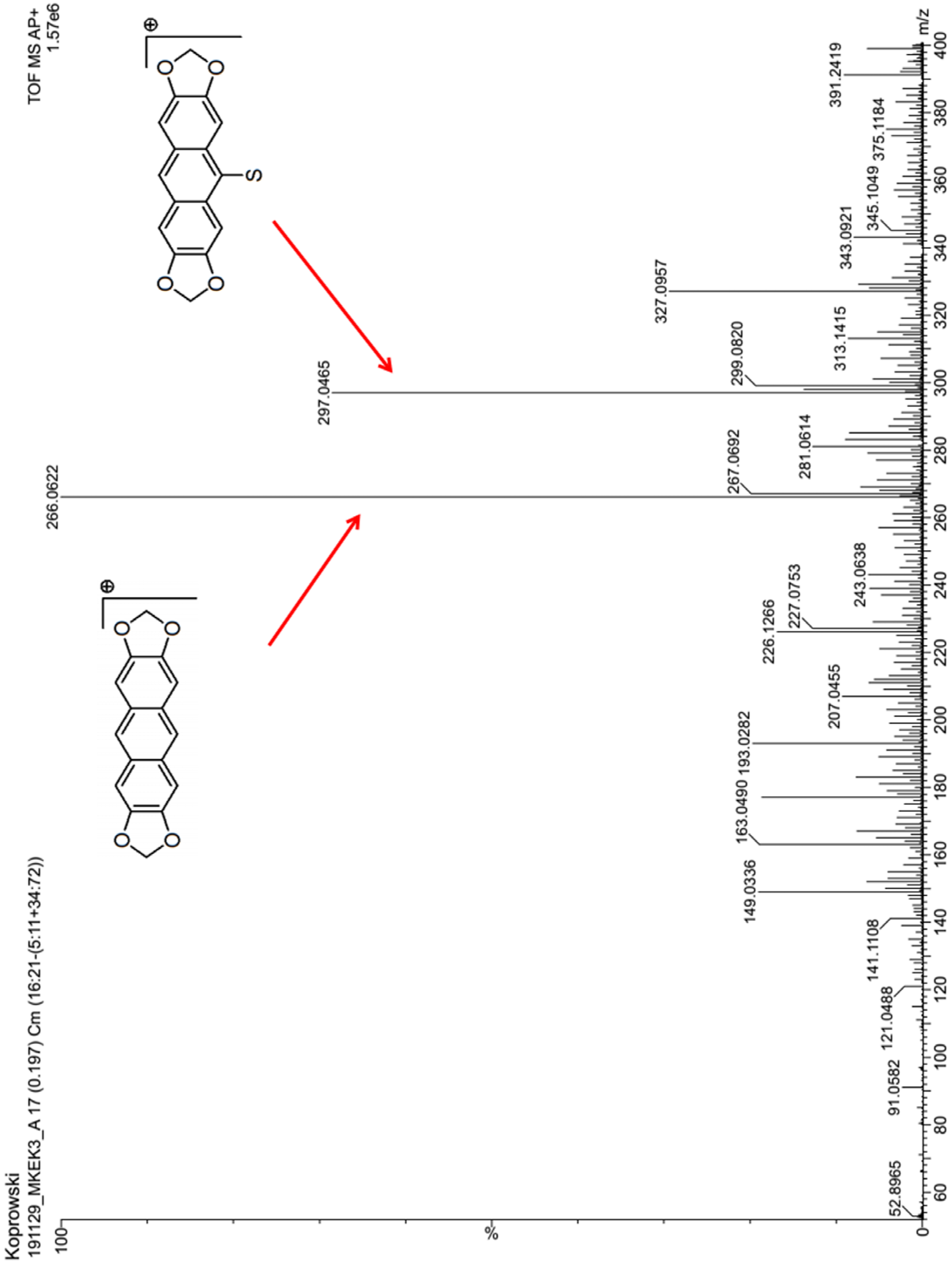

The HR(MS_MS)-(+)-APCI fragmentation spectrum of the peak at *m/z* = 266 (25eV) after irradiation of ethanolic solution of **3d** for 9 hours at 254 nm

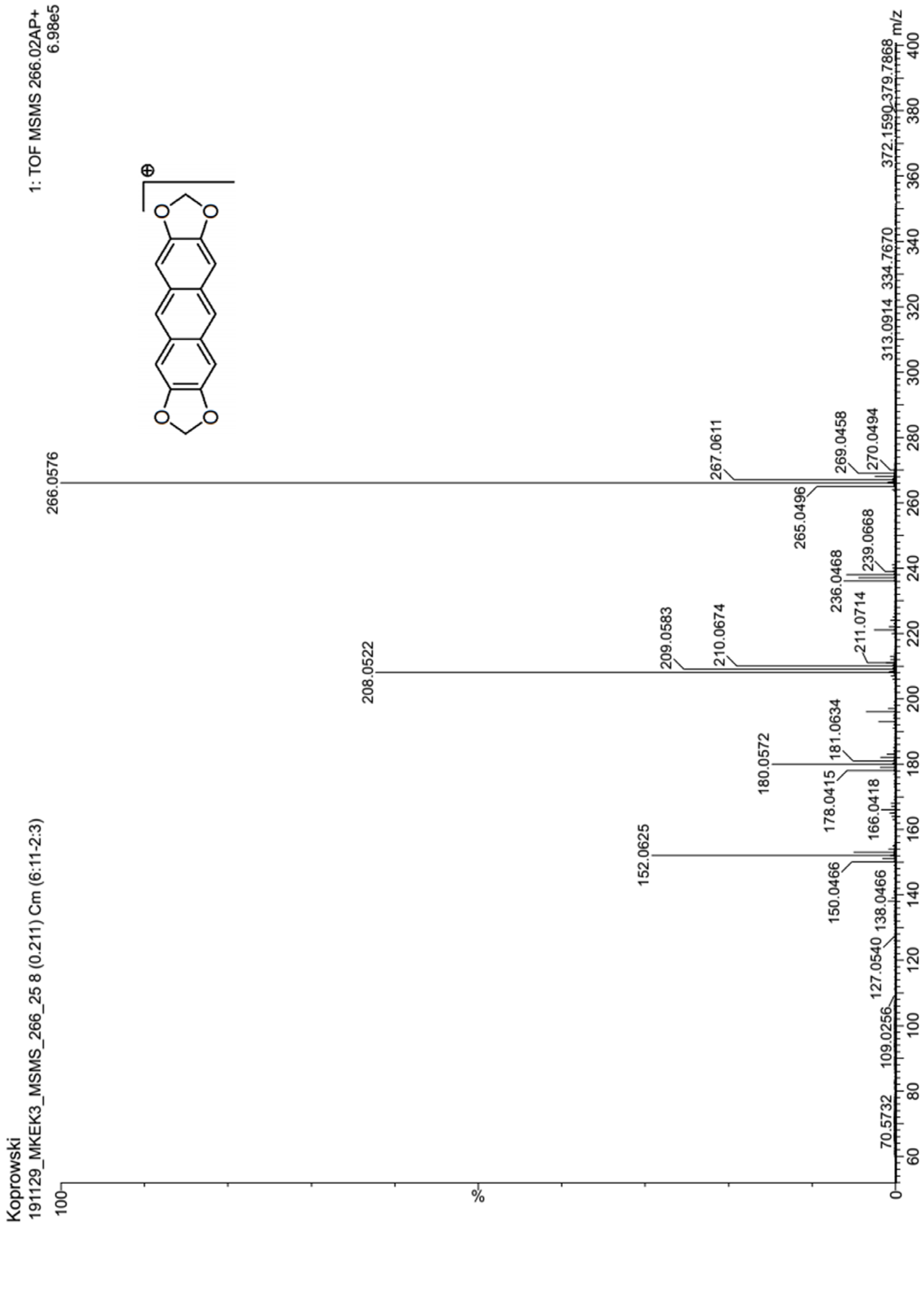

The HR(MS_MS)-(+)-APCI fragmentation spectrum of the peak at $m/z$ = 266 (35eV) after irradiation of ethanolic solution of **3d** for 9 hours at 254 nm

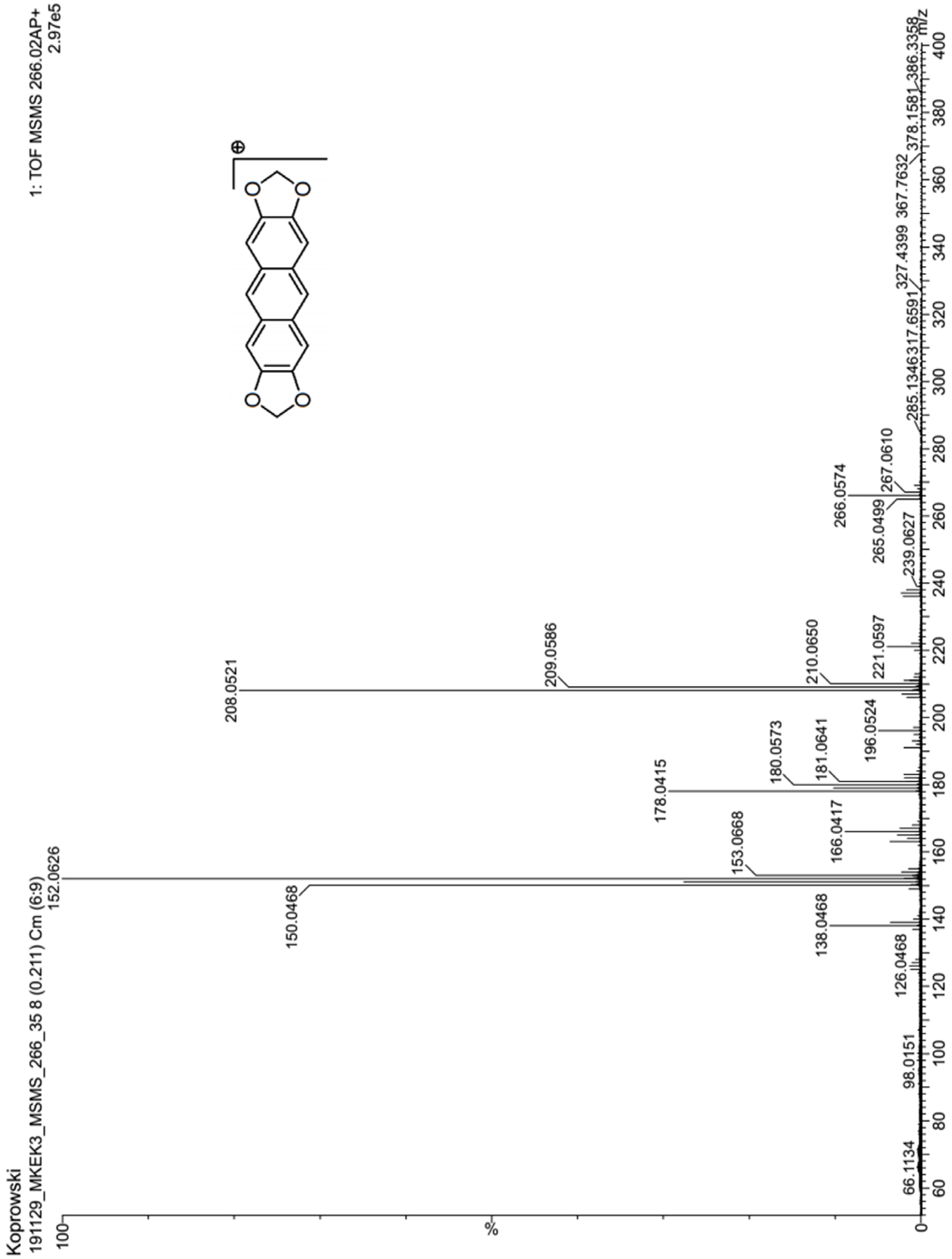

The HR(MS_MS)-(+)-APCI fragmentation spectrum of the peak at $m/z$ = 297 (25eV) after irradiation of ethanolic solution of **3d** for 9 hours at 254 nm

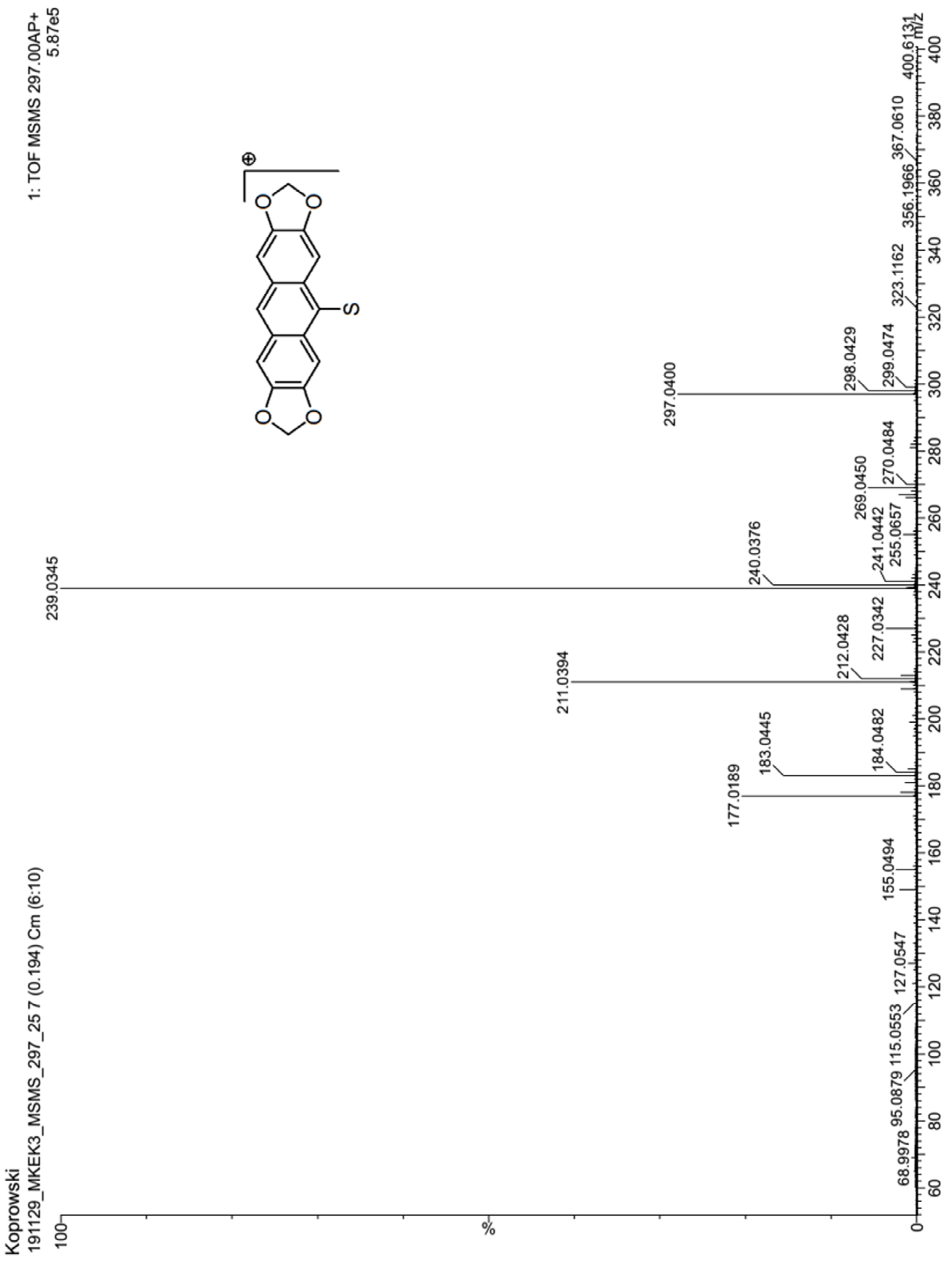

The HR(MS_MS)-(+)-APCI fragmentation spectrum of the peak at $m/z$ = 297 (35eV) after irradiation of ethanolic solution of **3d** for 9 hours at 254 nm

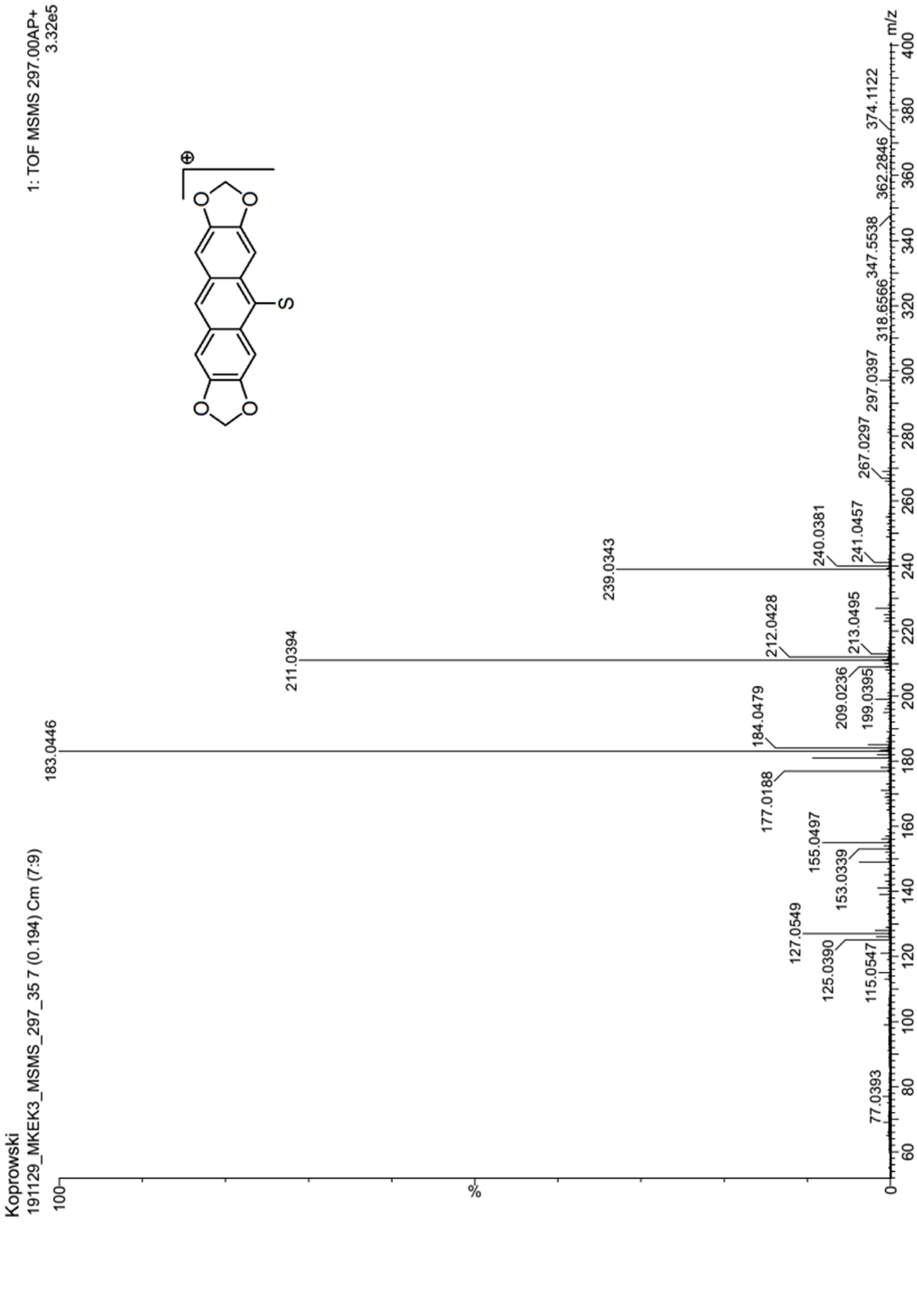

## Cartesian coordinates and total energies for the geometries of 1a, 1b, 1c, 1d, 2a, 2b, 2c, 2d, 3a, 3b, 3c and 3d optimized using Gaussian 09

**Table S1.** Atom coordinates (Å), total energy (Hartree) and the number of imaginary vibrational frequencies for the geometry of **1a** optimized at the B3LYP/6-311++(d,p) level in the gas phase using Gaussian 09.

| Atom | X | Y | Z |
|---|---|---|---|
| O | 4.950273 | -2.929777 | 0.359849 |
| O | 5.339227 | -0.830094 | -0.511165 |
| O | -4.35161 | 0.882437 | -0.779757 |
| O | -4.700165 | -1.577657 | 0.250672 |
| O | -2.504677 | -3.172552 | 0.845163 |
| C | 5.956015 | -1.930571 | 0.163999 |
| H | 6.330879 | -1.595390 | 1.138111 |
| H | 6.753032 | -2.336269 | -0.458107 |
| C | 3.749845 | -2.292425 | 0.177310 |
| C | 2.496487 | -2.749743 | 0.406222 |
| H | 2.311241 | -3.736081 | 0.812216 |
| C | 1.396954 | -1.880345 | 0.087874 |
| C | 0.086205 | -2.297989 | 0.315439 |
| H | -0.094990 | -3.278175 | 0.738436 |
| C | -1.009386 | -1.483571 | 0.027776 |
| C | -2.349710 | -1.931700 | 0.283119 |
| C | -3.428061 | -1.120715 | 0.027716 |
| C | -3.212014 | 0.181697 | -0.539458 |
| C | -1.944266 | 0.637267 | -0.798150 |
| H | -1.785047 | 1.614639 | -1.225856 |
| C | -0.803354 | -0.169394 | -0.519026 |
| C | 0.526066 | 0.264426 | -0.751435 |
| C | 1.636795 | -0.561688 | -0.463020 |
| C | 2.992779 | -0.139824 | -0.681309 |
| H | 3.203138 | 0.835584 | -1.094728 |
| C | 3.988175 | -1.002473 | -0.359082 |
| C | -4.236920 | 2.177529 | -1.361481 |
| H | -3.670636 | 2.854311 | -0.713786 |
| H | -5.256559 | 2.542714 | -1.471174 |
| H | -3.757941 | 2.126879 | -2.344334 |
| C | -5.366834 | -1.016190 | 1.390958 |

| | | | |
|---|---|---|---|
| H | -4.802391 | -1.227277 | 2.304648 |
| H | -6.339838 | -1.504002 | 1.441180 |
| H | -5.502626 | 0.060925 | 1.271969 |
| C | -3.161368 | -4.159412 | 0.033651 |
| H | -4.186701 | -3.862602 | -0.190809 |
| H | -3.157855 | -5.077874 | 0.620105 |
| H | -2.604746 | -4.318074 | -0.896064 |
| S | 0.802986 | 1.869460 | -1.509632 |
| C | 0.832725 | 3.016070 | -0.126098 |
| C | 0.914852 | 4.377664 | -0.445384 |
| C | 0.793759 | 2.623082 | 1.213270 |
| C | 0.957578 | 5.331082 | 0.567304 |
| H | 0.945059 | 4.689153 | -1.484244 |
| C | 0.834030 | 3.587067 | 2.220199 |
| H | 0.731457 | 1.573110 | 1.469753 |
| C | 0.916265 | 4.941813 | 1.906376 |
| H | 1.021871 | 6.381973 | 0.307041 |
| H | 0.802605 | 3.270551 | 3.257149 |
| H | 0.948174 | 5.685743 | 2.693870 |

| | |
|---|---|
| Total energy | -1701.218192 |
| Number of imaginary vibrational frequencies | 0 |

**Table S2.** Atom coordinates (Å), total energy (Hartree) and the number of imaginary vibrational frequencies for the geometry of **1b** optimized at the B3LYP/6-311++(d,p) level in the gas phase using Gaussian 09.

| | | | |
|---|---|---|---|
| O | 3.549354 | 5.093585 | 0.483647 |
| O | 1.560795 | 5.419780 | -0.637928 |
| C | 2.567945 | 6.076201 | 0.137975 |
| H | 2.117329 | 6.478468 | 1.052877 |
| H | 3.033771 | 6.858181 | -0.460659 |
| C | 2.950063 | 3.877694 | 0.273478 |
| C | 3.389112 | 2.640084 | 0.602638 |
| H | 4.321834 | 2.483825 | 1.129582 |
| C | 2.574282 | 1.517239 | 0.226860 |
| C | 2.974692 | 0.222394 | 0.555757 |
| H | 3.901775 | 0.075052 | 1.094148 |
| C | 2.214876 | -0.896732 | 0.215007 |
| C | 2.658766 | -2.222550 | 0.564623 |
| C | 1.906693 | -3.327504 | 0.230654 |
| C | 0.666446 | -3.139941 | -0.474440 |
| C | 0.230584 | -1.892401 | -0.837594 |
| H | -0.697374 | -1.768128 | -1.372154 |
| C | 0.982104 | -0.730744 | -0.502771 |
| C | 0.561162 | 0.581879 | -0.833402 |

| | | | |
|---|---|---|---|
| C | 1.329001 | 1.717060 | -0.485368 |
| C | 0.920325 | 3.058519 | -0.800068 |
| H | -0.003293 | 3.240028 | -1.329634 |
| C | 1.729291 | 4.077454 | -0.417404 |
| O | 3.811457 | -2.261519 | 1.291129 |
| C | 4.770209 | -3.304549 | 1.089418 |
| H | 5.703199 | -2.925582 | 1.507303 |
| H | 4.476423 | -4.220246 | 1.600646 |
| H | 4.907676 | -3.502459 | 0.021806 |
| S | -0.939875 | 0.810692 | -1.795092 |
| C | -2.265840 | 0.630532 | -0.596099 |
| C | -3.539443 | 0.424080 | -1.084247 |
| C | -2.052905 | 0.734081 | 0.802180 |
| C | -3.110944 | 0.625242 | 1.670158 |
| C | -4.434688 | 0.418489 | 1.202431 |
| C | -4.649350 | 0.320318 | -0.208426 |
| C | -5.971842 | 0.112207 | -0.684723 |
| C | -7.026726 | 0.007049 | 0.190158 |
| C | -6.813116 | 0.104031 | 1.585850 |
| C | -5.545113 | 0.305080 | 2.077940 |
| H | -6.137736 | 0.039539 | -1.754700 |
| H | -8.030865 | -0.150173 | -0.187796 |
| H | -7.654164 | 0.019492 | 2.264686 |
| H | -5.377001 | 0.380048 | 3.147474 |
| H | -2.938602 | 0.701240 | 2.739037 |
| H | -1.051517 | 0.894675 | 1.180720 |
| H | -3.707701 | 0.336574 | -2.153226 |
| O | 2.267145 | -4.569934 | 0.686655 |
| O | -0.022971 | -4.289622 | -0.708986 |
| C | 2.581755 | -5.568134 | -0.294821 |
| H | 1.715399 | -5.801620 | -0.913319 |
| H | 3.412188 | -5.236988 | -0.928521 |
| H | 2.883961 | -6.452192 | 0.266229 |
| C | -1.307098 | -4.196691 | -1.319382 |
| H | -1.682915 | -5.217131 | -1.371154 |
| H | -1.985054 | -3.584683 | -0.717208 |
| H | -1.237035 | -3.779650 | -2.329101 |
| Total energy | | -1854.894256 | |
| Number of imaginary vibrational frequencies | | 0 | |

**Table S3.** Atom coordinates (Å), total energy (Hartree) and the number of imaginary vibrational frequencies for the geometry of **1c** optimized at the B3LYP/6-311++(d,p) level in the gas phase using Gaussian 09.

| | | | |
|---|---|---|---|
| O | -5.579647 | -2.259367 | 0.473754 |

| | | | |
|---|---|---|---|
| O | -3.964316 | -3.288999 | -0.811478 |
| C | -5.145077 | -3.523752 | -0.038045 |
| H | -4.905878 | -4.191925 | 0.797235 |
| H | -5.920978 | -3.940374 | -0.679435 |
| C | -4.513169 | -1.410318 | 0.331353 |
| C | -4.356067 | -0.150426 | 0.800411 |
| H | -5.114011 | 0.335279 | 1.401877 |
| C | -3.139871 | 0.541199 | 0.470546 |
| C | -2.917446 | 1.834822 | 0.938760 |
| H | -3.677703 | 2.308757 | 1.552273 |
| C | -1.750845 | 2.542980 | 0.646134 |
| C | -1.543011 | 3.868543 | 1.138319 |
| C | -0.407844 | 4.562589 | 0.849148 |
| C | 0.599033 | 3.965332 | 0.034743 |
| C | 0.442824 | 2.693594 | -0.460603 |
| H | 1.198536 | 2.240953 | -1.083730 |
| C | -0.732650 | 1.937312 | -0.171154 |
| C | -0.941321 | 0.617009 | -0.643732 |
| C | -2.124100 | -0.098412 | -0.343387 |
| C | -2.356048 | -1.439546 | -0.805197 |
| H | -1.620280 | -1.943542 | -1.414390 |
| C | -3.520879 | -2.041590 | -0.459564 |
| S | 0.280387 | -0.136334 | -1.725972 |
| C | 1.549821 | -0.745113 | -0.609398 |
| C | 2.750953 | -1.130376 | -1.168138 |
| C | 1.345509 | -0.877941 | 0.787326 |
| C | 2.340561 | -1.388187 | 1.584060 |
| C | 3.585447 | -1.800068 | 1.042225 |
| C | 3.789489 | -1.668373 | -0.367508 |
| C | 5.034012 | -2.077575 | -0.918063 |
| C | 6.023656 | -2.591965 | -0.114945 |
| C | 5.820453 | -2.722290 | 1.279708 |
| C | 4.627861 | -2.334018 | 1.842794 |
| H | 5.191229 | -1.979860 | -1.987314 |
| H | 6.967588 | -2.902086 | -0.549306 |
| H | 6.609628 | -3.130077 | 1.901157 |
| H | 4.467860 | -2.432132 | 2.911686 |
| H | 2.176386 | -1.484319 | 2.652602 |
| H | 0.402469 | -0.572604 | 1.222108 |
| H | 2.916144 | -1.025948 | -2.236058 |
| O | 1.680696 | 4.760495 | -0.188043 |
| C | 2.741761 | 4.252325 | -0.989655 |
| H | 3.487543 | 5.044238 | -1.030609 |
| H | 3.182649 | 3.356062 | -0.541665 |
| H | 2.396410 | 4.021587 | -2.002733 |
| H | -2.314955 | 4.318306 | 1.753759 |
| H | -0.241003 | 5.567470 | 1.217390 |

| | |
|---|---|
| Total energy | -1625.796378 |
| Number of imaginary vibrational frequencies | 0 |

**Table S4.** Cartesian coordinates (Å), total energy (Hartree) and the number of imaginary vibrational frequencies for the geometry of **1d** optimized at the B3LYP/6-311++(d,p) level in the gas phase using Gaussian 09.

| Atom | X | Y | Z |
|---|---|---|---|
| O | 5.805040 | -0.645214 | -0.736548 |
| O | 4.752023 | -2.206873 | 0.594489 |
| O | -3.340698 | 3.4337340 | 0.591547 |
| O | -2.256826 | 4.936991 | -0.781623 |
| C | 5.874540 | -1.995981 | -0.266062 |
| H | 5.813538 | -2.681210 | -1.119606 |
| H | 6.795633 | -2.134970 | 0.298825 |
| C | 4.521956 | -0.230195 | -0.491500 |
| C | 3.900861 | 0.904290 | -0.892731 |
| H | 4.396452 | 1.638153 | -1.515617 |
| C | 2.546735 | 1.115745 | -0.460710 |
| C | 1.852941 | 2.258682 | -0.857261 |
| H | 2.356506 | 2.980575 | -1.492710 |
| C | 0.536511 | 2.510713 | -0.468496 |
| C | -0.130380 | 3.706345 | -0.903843 |
| C | -1.402738 | 3.906789 | -0.485114 |
| C | -2.067449 | 2.981814 | 0.356444 |
| C | -1.489165 | 1.836927 | 0.796772 |
| H | -2.018948 | 1.149838 | 1.439379 |
| C | -0.141595 | 1.560465 | 0.385840 |
| C | 0.548749 | 0.390523 | 0.783191 |
| C | 1.882582 | 0.141983 | 0.381088 |
| C | 2.599901 | -1.042151 | 0.766926 |
| H | 2.129578 | -1.784703 | 1.394583 |
| C | 3.873721 | -1.188296 | 0.326363 |
| S | -0.253857 | -0.775823 | 1.893756 |
| C | -1.348079 | -1.709455 | 0.811988 |
| C | -2.418453 | -2.377697 | 1.404389 |
| C | -1.148241 | -1.838754 | -0.567256 |
| C | -3.278216 | -3.172596 | 0.643812 |
| C | -2.007786 | -2.614828 | -1.330729 |
| H | -0.323261 | -1.327042 | -1.047217 |
| C | -3.076945 | -3.291613 | -0.732752 |
| H | -1.862922 | -2.713951 | -2.399979 |
| H | -2.594924 | -2.280538 | 2.470225 |
| H | -4.098418 | -3.677083 | 1.136619 |
| H | 0.378582 | 4.413270 | -1.546777 |

| | | | |
|---|---|---|---|
| C | -3.384578 | 4.770378 | 0.084878 |
| H | -3.309748 | 5.478755 | 0.918170 |
| H | -4.302226 | 4.912234 | -0.484977 |
| O | -3.861604 | -4.031682 | -1.572426 |
| C | -4.964576 | -4.736187 | -1.021359 |
| H | -5.685038 | -4.053034 | -0.557820 |
| H | -5.439864 | -5.247140 | -1.857223 |
| H | -4.638484 | -5.477036 | -0.282750 |
| Total energy | | -1660.699187 | |
| Number of imaginary vibrational frequencies | | 0 | |

**Table S5.** Atom coordinates (Å), total energy (Hartree) and the number of imaginary vibrational frequencies for the geometry of **2a** optimized at the B3LYP/6-311++(d,p) level in the gas phase using Gaussian 09.

| | | | |
|---|---|---|---|
| O | 4.138971 | -3.661782 | 0.288922 |
| O | 4.857729 | -1.668911 | -0.623094 |
| O | -5.149018 | -0.726687 | 0.324218 |
| C | 5.298863 | -2.862534 | 0.029124 |
| H | 5.780771 | -2.602582 | 0.978605 |
| H | 5.973258 | -3.407994 | -0.630284 |
| C | 3.065039 | -2.824904 | 0.139157 |
| C | 1.757415 | -3.062082 | 0.399070 |
| H | 1.414803 | -4.004213 | 0.807644 |
| C | 0.818733 | -2.015788 | 0.104580 |
| C | -0.540790 | -2.205404 | 0.354865 |
| H | -0.877631 | -3.142979 | 0.779040 |
| C | -1.490473 | -1.221665 | 0.082419 |
| C | -2.884500 | -1.450529 | 0.344687 |
| C | -3.821553 | -0.478199 | 0.097265 |
| C | -3.403543 | 0.772761 | -0.472592 |
| C | -2.079953 | 1.027258 | -0.728574 |
| H | -1.789810 | 1.971292 | -1.161098 |
| C | -1.074670 | 0.047289 | -0.455572 |
| C | 0.307950 | 0.248804 | -0.680086 |
| C | 1.270745 | -0.754942 | -0.445448 |
| C | 2.668833 | -0.574902 | -0.720540 |
| H | 3.029253 | 0.319789 | -1.205128 |
| C | 3.505832 | -1.598375 | -0.416461 |
| C | -5.714434 | -0.064064 | 1.465073 |
| H | -5.190460 | -0.363640 | 2.378154 |
| H | -6.753505 | -0.387552 | 1.516789 |
| H | -5.674026 | 1.020796 | 1.345380 |
| C | 1.652318 | 2.543138 | 0.155645 |
| C | 2.881521 | 3.160444 | -0.048364 |

| | | | |
|---|---|---|---|
| C | 1.046917 | 2.534493 | 1.411857 |
| C | 3.528815 | 3.760283 | 1.032598 |
| C | 1.700316 | 3.138648 | 2.483121 |
| H | 0.088737 | 2.049636 | 1.560590 |
| C | 2.940811 | 3.750953 | 2.295443 |
| H | 1.242475 | 3.127067 | 3.465901 |
| H | 3.445296 | 4.219161 | 3.133045 |
| O | 1.833919 | 1.777187 | -2.416010 |
| S | 0.782351 | 1.896401 | -1.325882 |
| O | -3.231034 | -2.652588 | 0.902396 |
| O | -4.420272 | 1.639138 | -0.720822 |
| C | -4.047123 | -3.517625 | 0.094859 |
| H | -4.197659 | -4.420941 | 0.685231 |
| H | -5.007599 | -3.052193 | -0.129195 |
| H | -3.526040 | -3.770951 | -0.834285 |
| C | -4.113742 | 2.886522 | -1.336378 |
| H | -5.067699 | 3.395352 | -1.462174 |
| H | -3.456363 | 3.491399 | -0.703318 |
| H | -3.645522 | 2.738548 | -2.314372 |
| H | 3.319552 | 3.154232 | -1.039753 |
| H | 4.492242 | 4.235021 | 0.883960 |
| Total energy | | -1776.410044 | |
| Number of imaginary vibrational frequencies | | 0 | |

**Table S6.** Atom coordinates (Å), total energy (Hartree) and the number of imaginary vibrational frequencies for the geometry of **2b** optimized at the B3LYP/6-311++(d,p) level in the gas phase using Gaussian 09.

| | | | |
|---|---|---|---|
| O | 1.934074 | 5.132201 | -0.498589 |
| O | 3.177942 | 3.696176 | 0.809865 |
| O | -5.647505 | -0.981351 | -0.649786 |
| C | 3.253762 | 4.865998 | -0.009274 |
| H | 3.923562 | 4.675887 | -0.855686 |
| H | 3.592484 | 5.708637 | 0.592786 |
| C | 1.213883 | 3.987784 | -0.280506 |
| C | -0.046520 | 3.686509 | -0.673488 |
| H | -0.641219 | 4.368687 | -1.267531 |
| C | -0.587658 | 2.420533 | -0.264931 |
| C | -1.881996 | 2.059012 | -0.640010 |
| H | -2.468580 | 2.736803 | -1.247392 |
| C | -2.453367 | 0.844921 | -0.261399 |
| C | -3.793882 | 0.507746 | -0.653594 |
| C | -4.356088 | -0.692817 | -0.297351 |
| C | -3.603210 | -1.608656 | 0.514380 |
| C | -2.317172 | -1.323375 | 0.897248 |

| | | | |
|---|---|---|---|
| H | -1.773098 | -2.024026 | 1.509866 |
| C | -1.691507 | -0.093536 | 0.520630 |
| C | -0.366938 | 0.260425 | 0.871232 |
| C | 0.202419 | 1.505664 | 0.532116 |
| C | 1.523223 | 1.895798 | 0.938502 |
| H | 2.105422 | 1.274870 | 1.602150 |
| C | 1.979028 | 3.102530 | 0.518410 |
| C | -5.800531 | -1.962171 | -1.686487 |
| H | -5.311162 | -1.624701 | -2.605520 |
| H | -6.872627 | -2.055961 | -1.856229 |
| H | -5.393569 | -2.926444 | -1.374268 |
| C | 1.792904 | -1.457169 | 0.524687 |
| C | 1.363275 | -1.876683 | -0.757290 |
| C | 4.092962 | -1.939988 | -0.041509 |
| C | 2.292350 | -2.311324 | -1.668888 |
| H | 0.312748 | -1.838770 | -1.021120 |
| C | 3.675848 | -2.356291 | -1.344931 |
| H | 1.975729 | -2.624400 | -2.658418 |
| O | 1.369318 | -0.362628 | 2.941595 |
| S | 0.575313 | -0.998893 | 1.813501 |
| O | -4.464176 | 1.406743 | -1.440100 |
| O | -4.278125 | -2.739217 | 0.849156 |
| C | -5.624711 | 2.022951 | -0.857042 |
| H | -5.999577 | 2.719367 | -1.606487 |
| H | -6.386932 | 1.279042 | -0.622380 |
| H | -5.346988 | 2.574242 | 0.047369 |
| C | -3.639836 | -3.683970 | 1.702916 |
| H | -4.367139 | -4.478613 | 1.858975 |
| H | -2.739846 | -4.097589 | 1.236279 |
| H | -3.378732 | -3.232193 | 2.664935 |
| C | 4.656311 | -2.800215 | -2.268817 |
| C | 5.986689 | -2.831422 | -1.921943 |
| H | 6.726587 | -3.172857 | -2.637128 |
| C | 6.398634 | -2.420617 | -0.632736 |
| H | 7.450463 | -2.450509 | -0.371622 |
| H | 4.339920 | -3.115608 | -3.257671 |
| C | 3.113490 | -1.496945 | 0.887181 |
| H | 3.403680 | -1.178558 | 1.882820 |
| C | 5.472962 | -1.984963 | 0.285871 |
| H | 5.785704 | -1.668786 | 1.275264 |

| | |
|---|---|
| Total energy | -1930.088365 |
| Number of imaginary vibrational frequencies | 0 |

**Table S7.** Atom coordinates (Å), total energy (Hartree) and the number of imaginary vibrational frequencies for the geometry of **2c**

optimized at the B3LYP/6-311++(d,p) level in the gas phase using Gaussian 09.

| | | | |
|---|---|---|---|
| O | 6,040244 | -1,298135 | -0,646512 |
| O | 4,796726 | -2,502426 | 0,878753 |
| C | 5,951376 | -2,554636 | 0,032960 |
| H | 5,826660 | -3,358410 | -0,701407 |
| H | 6,841062 | -2,699656 | 0,644705 |
| C | 4,816899 | -0,699746 | -0,493816 |
| C | 4,341910 | 0,433344 | -1,060985 |
| H | 4,926927 | 1,008707 | -1,767296 |
| C | 3,024091 | 0,870316 | -0,689142 |
| C | 2,487364 | 2,031195 | -1,242332 |
| H | 3,079453 | 2,589947 | -1,960807 |
| C | 1,219958 | 2,506769 | -0,903064 |
| C | 0,698459 | 3,704978 | -1,480638 |
| C | -0,535751 | 4,171795 | -1,145521 |
| C | -1,325682 | 3,464007 | -0,192537 |
| C | -0,870270 | 2,303275 | 0,385835 |
| H | -1,444832 | 1,801553 | 1,147499 |
| C | 0,411463 | 1,780370 | 0,041088 |
| C | 0,937340 | 0,581882 | 0,577527 |
| C | 2,233199 | 0,114388 | 0,264235 |
| C | 2,804903 | -1,080383 | 0,831911 |
| H | 2,269710 | -1,671583 | 1,559786 |
| C | 4,053114 | -1,440128 | 0,442160 |
| C | -1,286104 | -1,094173 | 0,591006 |
| C | -2,592011 | -1,073662 | 1,006252 |
| C | -0,906552 | -1,710436 | -0,625314 |
| C | -1,869536 | -2,277640 | -1,421668 |
| C | -3,239105 | -2,266863 | -1,041717 |
| C | -3,605744 | -1,653673 | 0,197637 |
| C | -4,971666 | -1,646669 | 0,582939 |
| C | -5,931172 | -2,215536 | -0,220858 |
| C | -5,569207 | -2,818599 | -1,448028 |
| C | -4,253690 | -2,844105 | -1,847601 |
| H | -5,246944 | -1,186158 | 1,525886 |
| H | -6,971543 | -2,204444 | 0,084024 |
| H | -6,335568 | -3,263121 | -2,072902 |
| H | -3,974987 | -3,307583 | -2,788246 |
| H | -1,590400 | -2,740979 | -2,362277 |
| H | 0,132364 | -1,718529 | -0,933017 |
| H | -2,844430 | -0,604641 | 1,951354 |
| O | -2,522991 | 4,045817 | 0,080689 |
| C | -3,375425 | 3,421496 | 1,038167 |
| H | -4,258267 | 4,054846 | 1,104276 |
| H | -3,665166 | 2,418279 | 0,710253 |

| | | | |
|---|---|---|---|
| H | -2,890654 | 3,358435 | 2,016997 |
| H | 1,310465 | 4,243341 | -2,196508 |
| H | -0,939820 | 5,080865 | -1,574004 |
| S | -0,025340 | -0,446030 | 1,751359 |
| O | -0,777421 | 0,416022 | 2,751940 |
| Total energy | | -1700.989221 | |
| Number of imaginary vibrational frequencies | | 0 | |

**Table S8.** Atom coordinates (Å), total energy (Hartree) and the number of imaginary vibrational frequencies for the geometry of **2d** optimized at the B3LYP/6-311++(d,p) level in the gas phase using Gaussian 09.

| | | | |
|---|---|---|---|
| O | -5.684540 | -0.431187 | -0.939139 |
| O | -4.985005 | 1.222510 | 0.508859 |
| O | 4.010643 | -2.857000 | 0.531235 |
| O | 3.239885 | -4.474851 | -0.920747 |
| C | -6.024452 | 0.852225 | -0.397960 |
| H | -6.078925 | 1.584294 | -1.212070 |
| H | -6.968130 | 0.777615 | 0.141577 |
| C | -4.359361 | -0.618718 | -0.652694 |
| C | -3.529559 | -1.605780 | -1.065122 |
| H | -3.858963 | -2.388846 | -1.736289 |
| C | -2.177215 | -1.589387 | -0.581066 |
| C | -1.280631 | -2.571954 | -0.997972 |
| H | -1.630532 | -3.340792 | -1.679715 |
| C | 0.048980 | -2.604036 | -0.578874 |
| C | 0.927035 | -3.641785 | -1.042953 |
| C | 2.208155 | -3.629248 | -0.605112 |
| C | 2.680991 | -2.634796 | 0.284095 |
| C | 1.899036 | -1.631519 | 0.754564 |
| H | 2.309171 | -0.901314 | 1.435713 |
| C | 0.524849 | -1.583092 | 0.328062 |
| C | -0.378025 | -0.576305 | 0.742887 |
| C | -1.727687 | -0.549302 | 0.325463 |
| C | -2.665502 | 0.466458 | 0.730522 |
| H | -2.376353 | 1.227316 | 1.440764 |
| C | -3.924756 | 0.397359 | 0.232955 |
| C | 0.997840 | 1.850077 | 0.753226 |
| C | 2.195925 | 2.454584 | 1.118973 |
| C | 0.348089 | 2.249825 | -0.420155 |
| C | 2.765547 | 3.448679 | 0.321886 |
| C | 0.904659 | 3.234667 | -1.216944 |
| H | -0.586093 | 1.788886 | -0.713688 |
| C | 2.116620 | 3.841212 | -0.851698 |
| H | 0.420575 | 3.555494 | -2.131423 |

| | | | |
|---|---|---|---|
| H | 2.698078 | 2.154185 | 2.032616 |
| H | 3.699528 | 3.901551 | 0.625271 |
| H | 0.562615 | -4.400134 | -1.724355 |
| C | 4.302732 | -4.145324 | -0.020357 |
| H | 4.339056 | -4.886323 | 0.786521 |
| H | 5.241832 | -4.099146 | -0.570394 |
| O | 2.576118 | 4.794898 | -1.704697 |
| C | 3.796821 | 5.459046 | -1.397527 |
| H | 4.634286 | 4.754635 | -1.359743 |
| H | 3.958611 | 6.169492 | -2.206093 |
| H | 3.727360 | 5.999280 | -0.447722 |
| S | 0.317368 | 0.644804 | 1.941652 |
| O | -0.790921 | 1.314333 | 2.732912 |
| Total energy | | -1735.888995 | |
| Number of imaginary vibrational frequencies | | 0 | |

**Table S9.** Atom coordinates (Å), total energy (Hartree) and the number of imaginary vibrational frequencies for the geometry of **3a** optimized at the B3LYP/6-311++(d,p) level in the gas phase using Gaussian 09.

| | | | |
|---|---|---|---|
| O | -3.965899 | 3.826175 | 0.132703 |
| O | -4.720013 | 1.848936 | -0.787968 |
| O | 5.240895 | 0.745342 | 0.396867 |
| C | -5.143350 | 3.069642 | -0.171641 |
| H | -5.679098 | 2.841404 | 0.756610 |
| H | -5.763875 | 3.631081 | -0.869402 |
| C | -2.919538 | 2.948257 | 0.031378 |
| C | -1.611879 | 3.131488 | 0.325638 |
| H | -1.237005 | 4.063704 | 0.728198 |
| C | -0.708153 | 2.044169 | 0.071452 |
| C | 0.646576 | 2.208236 | 0.348263 |
| H | 0.992869 | 3.145486 | 0.765010 |
| C | 1.582778 | 1.209443 | 0.102409 |
| C | 2.972780 | 1.451459 | 0.379277 |
| C | 3.918627 | 0.483135 | 0.161079 |
| C | 3.503672 | -0.773799 | -0.391847 |
| C | 2.184277 | -1.040259 | -0.660410 |
| H | 1.908221 | -1.992415 | -1.073390 |
| C | 1.163984 | -0.074143 | -0.409546 |
| C | -0.228205 | -0.257621 | -0.655160 |
| C | -1.176665 | 0.786748 | -0.473189 |
| C | -2.574860 | 0.675512 | -0.791914 |
| H | -2.976939 | -0.195163 | -1.281534 |
| C | -3.381553 | 1.734395 | -0.526956 |
| C | 5.813776 | 0.075770 | 1.530328 |

| | | | |
|---|---|---|---|
| H | 5.284536 | 0.357999 | 2.445953 |
| H | 6.848015 | 0.413397 | 1.587235 |
| H | 5.787749 | -1.007602 | 1.397365 |
| C | -1.939629 | -2.342645 | 0.314452 |
| C | -3.241442 | -2.772938 | 0.081191 |
| C | -1.390011 | -2.341702 | 1.594733 |
| C | -4.012892 | -3.205208 | 1.159647 |
| C | -2.171516 | -2.767698 | 2.664630 |
| H | -0.369785 | -2.013449 | 1.754600 |
| C | -3.481210 | -3.199168 | 2.447448 |
| H | -1.757722 | -2.766089 | 3.666440 |
| H | -4.085290 | -3.532162 | 3.283828 |
| O | 3.304636 | 2.667821 | 0.916634 |
| O | 4.518384 | -1.645176 | -0.620859 |
| C | 4.121564 | 3.521780 | 0.098438 |
| H | 4.258922 | 4.439304 | 0.669946 |
| H | 5.088042 | 3.059484 | -0.105376 |
| H | 3.608508 | 3.749999 | -0.841759 |
| C | 4.209199 | -2.908322 | -1.210324 |
| H | 5.161960 | -3.425309 | -1.308750 |
| H | 3.535123 | -3.488874 | -0.573864 |
| H | 3.755011 | -2.779776 | -2.196853 |
| S | -0.904840 | -1.895878 | -1.108697 |
| O | 0.159206 | -2.908445 | -1.169587 |
| O | -1.791499 | -1.759097 | -2.273386 |
| H | -3.636693 | -2.767332 | -0.927078 |
| H | -5.028762 | -3.543261 | 0.990492 |
| Total energy | | -1851.635463 | |
| Number of imaginary vibrational frequencies | | 0 | |

**Table S10.** Atom coordinates (Å), total energy (Hartree) and the number of imaginary vibrational frequencies for the geometry of **3b** optimized at the B3LYP/6-311++(d,p) level in the gas phase using Gaussian 09.

| | | | |
|---|---|---|---|
| O | -2.125453 | 4.992187 | 0.306522 |
| O | -3.251973 | 3.480356 | -1.024523 |
| O | 5.782054 | -0.622815 | 0.814117 |
| C | -3.409469 | 4.665984 | -0.237968 |
| H | -4.112046 | 4.469199 | 0.579540 |
| H | -3.749191 | 5.481024 | -0.876319 |
| C | -1.352684 | 3.873692 | 0.141371 |
| C | -0.097182 | 3.624301 | 0.578792 |
| H | 0.457314 | 4.337185 | 1.175419 |
| C | 0.504645 | 2.372236 | 0.212987 |
| C | 1.802604 | 2.094556 | 0.633225 |

| | | | |
|---|---|---|---|
| H | 2.329677 | 2.819271 | 1.240712 |
| C | 2.458459 | 0.915665 | 0.294423 |
| C | 3.811665 | 0.701858 | 0.729815 |
| C | 4.481069 | -0.455154 | 0.424322 |
| C | 3.819822 | -1.442621 | -0.379121 |
| C | 2.525821 | -1.273551 | -0.804348 |
| H | 2.058068 | -2.032110 | -1.403774 |
| C | 1.780885 | -0.101864 | -0.473812 |
| C | 0.437490 | 0.163383 | -0.870244 |
| C | -0.208519 | 1.399438 | -0.589052 |
| C | -1.526302 | 1.748382 | -1.048151 |
| H | -2.075318 | 1.114547 | -1.723582 |
| C | -2.044231 | 2.943911 | -0.668280 |
| C | 5.999221 | -1.588029 | 1.854417 |
| H | 5.458540 | -1.298113 | 2.760896 |
| H | 7.070864 | -1.582053 | 2.050546 |
| H | 5.690580 | -2.584651 | 1.532477 |
| C | -1.854400 | -1.474052 | -0.397156 |
| C | -1.449723 | -1.875119 | 0.898515 |
| C | -4.180583 | -1.761138 | 0.185082 |
| C | -2.405225 | -2.205171 | 1.825017 |
| H | -0.397013 | -1.919250 | 1.149652 |
| C | -3.789261 | -2.159742 | 1.501821 |
| H | -2.110539 | -2.511368 | 2.823117 |
| O | -1.274312 | -0.579706 | -2.831713 |
| S | -0.593299 | -1.131540 | -1.651473 |
| O | 4.386536 | 1.677848 | 1.501599 |
| O | 4.582626 | -2.525629 | -0.673435 |
| C | 5.500577 | 2.373144 | 0.917085 |
| H | 5.805860 | 3.117850 | 1.651642 |
| H | 6.324860 | 1.688775 | 0.713057 |
| H | 5.192467 | 2.875021 | -0.006208 |
| C | 4.028765 | -3.537784 | -1.514302 |
| H | 4.813573 | -4.283105 | -1.629769 |
| H | 3.146473 | -3.995154 | -1.057063 |
| H | 3.759122 | -3.130041 | -2.492640 |
| C | -4.797111 | -2.497065 | 2.440795 |
| C | -6.126880 | -2.443906 | 2.094140 |
| H | -6.887468 | -2.705528 | 2.821110 |
| C | -6.512656 | -2.050670 | 0.791513 |
| H | -7.564506 | -2.014543 | 0.531965 |
| H | -4.502650 | -2.799844 | 3.440117 |
| C | -3.175356 | -1.419242 | -0.757506 |
| H | -3.451219 | -1.116445 | -1.760958 |
| C | -5.560456 | -1.716623 | -0.141972 |
| H | -5.851538 | -1.414918 | -1.142364 |
| O | 0.165642 | -2.379742 | -1.817872 |

| | |
|---|---|
| Total energy | -2005.313792 |
| Number of imaginary vibrational frequencies | 0 |

**Table S11.** Atom coordinates (Å), total energy (Hartree) and the number of imaginary vibrational frequencies for the geometry of **3c** optimized at the B3LYP/6-311++(d,p) level in the gas phase using Gaussian 09.

| | | | |
|---|---|---|---|
| O | 6,211543 | -0,913240 | -0,872759 |
| O | 5,146462 | -2,226017 | 0,699522 |
| C | 6,280447 | -2,160832 | -0,169666 |
| H | 6,235882 | -2,985681 | -0,889274 |
| H | 7,194123 | -2,191035 | 0,423537 |
| C | 4,946126 | -0,435650 | -0,668694 |
| C | 4,338393 | 0,645583 | -1,206885 |
| H | 4,831734 | 1,283430 | -1,929040 |
| C | 3,002257 | 0,950224 | -0,774103 |
| C | 2,360710 | 2,066083 | -1,300719 |
| H | 2,880212 | 2,663933 | -2,043360 |
| C | 1,088809 | 2,461738 | -0,896384 |
| C | 0,491427 | 3,640068 | -1,440009 |
| C | -0,737034 | 4,063339 | -1,038574 |
| C | -1,434375 | 3,323216 | -0,042129 |
| C | -0,903595 | 2,175615 | 0,499391 |
| H | -1,428663 | 1,668529 | 1,289177 |
| C | 0,373254 | 1,685719 | 0,083301 |
| C | 0,990147 | 0,495270 | 0,569790 |
| C | 2,311477 | 0,118211 | 0,198745 |
| C | 3,029090 | -1,008121 | 0,736451 |
| H | 2,584664 | -1,649776 | 1,477410 |
| C | 4,289584 | -1,240012 | 0,291479 |
| C | -1,379500 | -1,097284 | 0,454885 |
| C | -2,674758 | -1,040220 | 0,900091 |
| C | -1,049909 | -1,630802 | -0,812717 |
| C | -2,054185 | -2,081946 | -1,630593 |
| C | -3,413996 | -2,035726 | -1,219026 |
| C | -3,728809 | -1,510266 | 0,073695 |
| C | -5,084571 | -1,473255 | 0,491972 |
| C | -6,085151 | -1,929483 | -0,332586 |
| C | -5,775093 | -2,443823 | -1,613256 |
| C | -4,470867 | -2,496344 | -2,045369 |
| H | -5,318748 | -1,082449 | 1,476541 |
| H | -7,117530 | -1,898152 | -0,003435 |
| H | -6,573677 | -2,800040 | -2,254101 |
| H | -4,233802 | -2,893216 | -3,026807 |
| H | -1,816078 | -2,485921 | -2,608756 |

| | | | |
|---|---|---|---|
| H | -0,015524 | -1,676899 | -1,130561 |
| H | -2,891775 | -0,637544 | 1,882875 |
| O | -2,628905 | 3,862105 | 0,316396 |
| C | -3,397393 | 3,211802 | 1,325298 |
| H | -4,291696 | 3,819337 | 1,451464 |
| H | -3,680486 | 2,201675 | 1,013357 |
| H | -2,848456 | 3,163083 | 2,270568 |
| H | 1,047943 | 4,200892 | -2,183175 |
| H | -1,198411 | 4,958007 | -1,437814 |
| S | -0,057796 | -0,602333 | 1,591709 |
| O | -0,675717 | 0,169991 | 2,680448 |
| O | 0,657881 | -1,835274 | 1,944860 |
| Total energy | | -1776.213135 | |
| Number of imaginary vibrational frequencies | | 0 | |

**Table S12.** Atom coordinates (Å), total energy (Hartree) and the number of imaginary vibrational frequencies for the geometry of **3d** optimized at the B3LYP/6-311++(d,p) level in the gas phase using Gaussian 09.

| | | | |
|---|---|---|---|
| O | 6.013494 | -0.097729 | -1.094753 |
| O | 5.264964 | -1.727096 | 0.358736 |
| O | -3.419785 | 3.008559 | 0.755448 |
| O | -2.586324 | 4.540939 | -0.755090 |
| C | 6.317156 | -1.388185 | -0.547374 |
| H | 6.353548 | -2.125041 | -1.357495 |
| H | 7.260947 | -1.336922 | -0.005074 |
| C | 4.702018 | 0.140135 | -0.788587 |
| C | 3.899379 | 1.156125 | -1.179370 |
| H | 4.242451 | 1.937117 | -1.845774 |
| C | 2.557807 | 1.188114 | -0.665808 |
| C | 1.715953 | 2.227285 | -1.048972 |
| H | 2.095504 | 2.974087 | -1.739272 |
| C | 0.414596 | 2.362224 | -0.573154 |
| C | -0.376789 | 3.483100 | -0.997628 |
| C | -1.629969 | 3.595156 | -0.500007 |
| C | -2.140565 | 2.651457 | 0.419895 |
| C | -1.438142 | 1.576590 | 0.857997 |
| H | -1.860705 | 0.928748 | 1.606807 |
| C | -0.107744 | 1.376018 | 0.348232 |
| C | 0.724986 | 0.274749 | 0.694110 |
| C | 2.066923 | 0.158739 | 0.234708 |
| C | 2.983908 | -0.885510 | 0.613517 |
| H | 2.688257 | -1.667004 | 1.291688 |

| | | | |
|---|---|---|---|
| C | 4.238108 | -0.858421 | 0.098299 |
| C | -1.343979 | -1.694153 | 0.561870 |
| C | -2.640447 | -1.829101 | 1.037386 |
| C | -1.003962 | -2.137056 | -0.719737 |
| C | -3.622781 | -2.401870 | 0.228933 |
| C | -1.976110 | -2.697002 | -1.528569 |
| H | 0.012634 | -2.042412 | -1.082618 |
| C | -3.292350 | -2.833511 | -1.058946 |
| H | -1.743642 | -3.042630 | -2.528272 |
| H | -2.883359 | -1.490268 | 2.036946 |
| H | -4.628688 | -2.501507 | 0.612662 |
| H | 0.033788 | 4.206197 | -1.690677 |
| C | -3.625528 | 4.313305 | 0.204919 |
| H | -3.552966 | 5.061071 | 1.002645 |
| H | -4.592887 | 4.347517 | -0.295116 |
| O | -4.169511 | -3.396079 | -1.930625 |
| C | -5.523286 | -3.571770 | -1.525832 |
| H | -5.997248 | -2.611777 | -1.296799 |
| H | -6.027703 | -4.030881 | -2.373973 |
| H | -5.595620 | -4.234614 | -0.657531 |
| S | -0.067125 | -1.064339 | 1.670734 |
| O | 0.873554 | -2.168387 | 1.899971 |
| O | -0.742070 | -0.482802 | 2.840666 |

| | |
|---|---|
| Total energy | -1811.117786 |
| Number of imaginary vibrational frequencies | 0 |

## References


1. Bałczewski, P.; Kowalska, E.; Różycka-Sokołowska, E.; Skalik, J.; Owsianik, K.; Koprowski, M.; Marciniak, B.; Guziejewski, D.; Ciesielski, W. Mono-Aryl/Alkylthio-Substituted (Hetero)acenes of Exceptional Thermal and Photochemical Stability by the Thio-Friedel–Crafts/Bradsher Cyclization Reaction. *Chem. Eur. J.* **2019**, *25*, 14148-14161. DOI: 10.1002/chem.201903027
